# Triflate-Enabled Rhodium-Catalyzed Cross-Dehydrogenative Si–N Bond Formation from Hydrosiloxanes

**DOI:** 10.1021/acs.inorgchem.6c01798

**Published:** 2026-07-07

**Authors:** Marina Padilla, María Batuecas, Pilar García-Orduña, Luis A. Oro, Francisco J. Fernández-Álvarez

**Affiliations:** Departamento de Química Inorgánica–Instituto de Síntesis Química y Catálisis Homogénea (ISQCH), 16765 Universidad de Zaragoza, CSIC, Facultad de Ciencias, Zaragoza 50009, Spain

## Abstract

Hydrosiloxanes are an attractive yet underexplored class of silicon reagents for Si–N bond formation, largely due to challenges associated with their activation. Herein, we demonstrate that the triflate anion plays a non-inonnocent role in catalysis by promoting substrate organization and N–H activation. Rhodium complexes of the type [Rh­(H)­(X)­(κ^2^-NSi^DMQ^)­(PCy_3_)] (NSi^DMQ^ = {4,8-dimethylquinolin-2-yloxy}­dimethylsilyl; X = Cl, OTf), featuring an organosilyl ligand, display marked anion-dependent reactivity in the cross-dehydrogenative coupling (CDC) of secondary amines with hydrosiloxanes. Whereas the chloride complex is catalytically inactive, the triflate derivative efficiently promotes Si–N bond formation. Among the hydrosiloxanes investigated, HSiMe­(OSiMe_3_)_2_ proved to be the most versatile reagent. Combined experimental and computational studies reveal an unconventional pathway in which hydrosiloxanes coordinate to the metal center through the oxygen atom rather than via the Si–H bond. This reactivity is enabled by the hemilability of the NSi^DMQ^ ligand, which undergoes hemidissociation to generate the active species. The triflate ligand facilitates N–H bond activation by organizing the amine through hydrogen-bonding interactions, thereby promoting Si–N bond formation. The interplay between anion effects and ligand hemilability provides valuable insight for the design of catalytic systems for Si–N bond formation from challenging substrates such as hydrosiloxanes.

## Graphical abstract

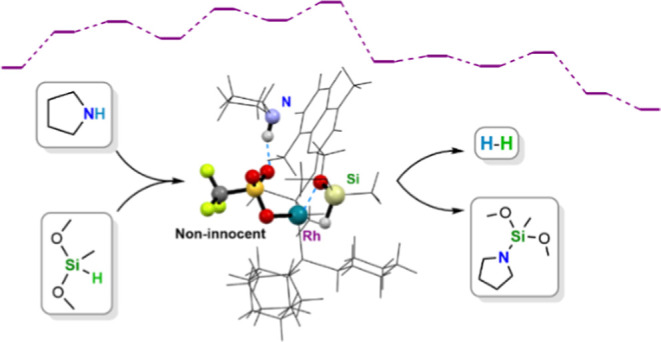

## Introduction

Siloxazanes are organosilicon compounds containing both Si–O and Si–N linkages within the same molecular framework. These motifs are of considerable interest because they constitute key structural units in a variety of silicon-based materials, including polysiloxazanes and related precursors to silicon-based ceramics. Owing to the unique combination of Si–O and Si–N linkages, such compounds have attracted attention as versatile building blocks for the preparation of functional materials and hybrid inorganic–organic architectures.^1^

Several synthetic approaches have been developed for the preparation of siloxazanes. Traditional methodologies typically rely on: (i) the reaction of the Si–Cl bonds in silanes with amines or lithium amides;^2^ (ii) condensation of organosilanes bearing Si–Cl or Si–OH functionalities;^3^ and (iii) ring-opening of cyclosiloxazanes.^4^ However, these methods often suffer from limited substrate scope, poor selectivity, or the generation of stoichiometric amounts of byproducts, which can complicate the preparation of structurally well-defined siloxazanes.

In this context, catalytic methodologies enabling the selective formation of Si–N and Si–O bonds represent an attractive alternative. Nevertheless, efficient catalytic strategies for the synthesis of siloxazanes remain relatively underdeveloped. To the best of our knowledge, only a limited number of catalytic methods have been reported, including the cross-dehydrogenative coupling (CDC) of amines with hydrosiloxanes (Scheme 1A)^5^ and the B­(C_6_F_5_)_3_-catalyzed reaction of methoxysilazanes with hydrosilanes (Scheme 1B).^6^

**Scheme 1:**
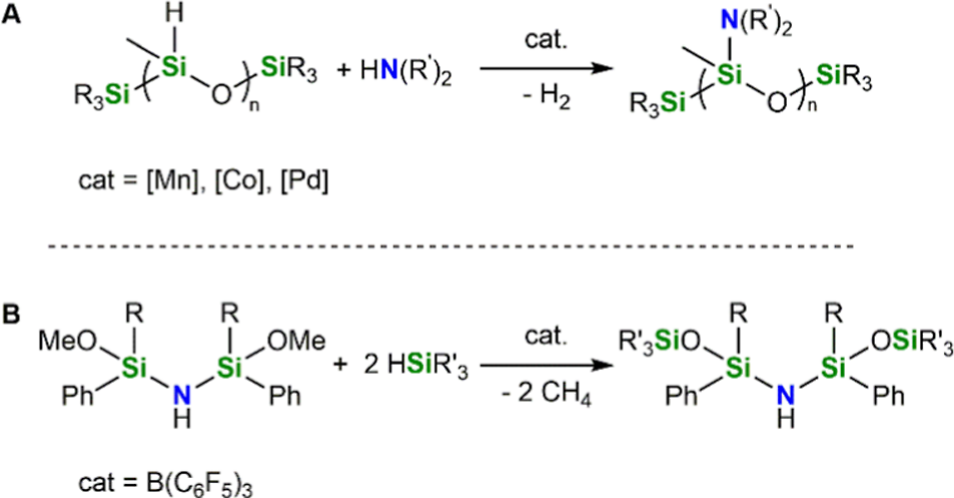
Reported Catalytic Methodologies for the Synthesis of Siloxazanes; (A) TM-Catalyzed CDC of Polyhydrosiloxanes with Secondary Amines; (B) B­(C_6_F_5_)_3_-Catalyzed Reaction of Methoxysilazanes with Hydrosilanes

The catalytic CDC of amines with hydrosilanes, first reported in 1966,^7^ constitutes a direct and highly efficient strategy for the formation of Si–N bonds and, thus provides a straightforward and atom-economical route to siloxazanes. These reactions are generally highly selective and generate molecular hydrogen (H_2_) as the sole byproduct, making them an alternative to traditional stoichiometric approaches.^8–10^

Homogeneous catalysts for the CDC of amines with hydrosilanes have been developed based on main group elements,^11–14^ lanthanides and actinides,^15^ and various transition metals.^16–27^ Nevertheless, most reported catalytic systems have been applied almost exclusively to hydrosilanes, whereas examples involving hydrosiloxanes remain scarce (Scheme 1A).^5^ This distinction is important, since hydrosiloxanes are often available as industrial byproducts of the silicone industry, making their use particularly attractive.^28–30^

Building on our previous studies of transition metal (TM) complexes with monoanionic organosilyl ligands,^31,32^ we recently demonstrated that iridium–(κ^2^-NSi) species with the ligand (4,8-dimethylquinolin-2-yloxy)­dimethylsilyl (NSi^DMQ^) are among the most efficient catalysts for the CDC of aniline derivatives with HSiMe_2_Ph.^8^ However, their catalytic activity strongly depends on the silane employed. Bulkier hydrosilanes like HSiEt_3_ or HSiMe­(OSiMe_3_)_2_ exhibit markedly reduced reactivity, most likely due to steric hindrance that hampers coordination and/or activation at the metal center.

Motivated by the need for efficient and selective methodologies for the synthesis of siloxazanes, as well as by our experience in the CDC of amines with hydrosilanes,^8,25^ we herein report the development and mechanistic investigation of Rh–(κ^2^-NSi) catalyzed CDC of secondary amines with hydrosiloxanes, providing a straightforward and atom-economical entry to siloxazanes.

## Results and Discussion

### Synthesis and Characterization of Rh–(κ^2^-NSi^DMQ^) Precatalysts

The reaction of the rhodium­(I) dimer [{Rh­(coe)_2_}_2_(μ-Cl)_2_][Bibr ref33] with two equivalents of the proligand (4,8-dimethylquinolin-2-yloxy)­dimethylsilane (**1**)^8^ and PCy_3_ in toluene at room temperature (r.t.) affords, after 20 h, the rhodium­(III) complex [Rh­(H)­(Cl)­(κ^2^-NSi^DMQ^)­(PCy_3_)] (NSi^DMQ^ = {4,8-dimethylquinolin-2-yloxy}­dimethylsilyl; **2**), which was isolated as a white solid in 70% yield (Scheme 2). Complex **2** reacts with one equivalent of silver triflate (AgOTf) in CH_2_Cl_2_ at r.t. in the dark, affording after 15 h the complex [Rh­(H)­(OTf)­(κ^2^-NSi^DMQ^)­(PCy_3_)] (**3**), which was isolated as a light-brown solid in 73% yield (Scheme 2).

**Scheme 2:**
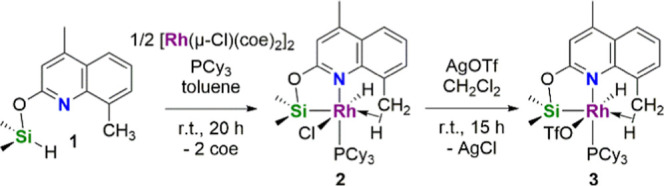
Synthesis of the Rhodium Complexes [Rh­(H)­(X)­(κ^2^-NSi^DMQ^)­(PCy_3_)] (NSi^DMQ^ = {4,8-Dimethylquinolin-2-yloxy}­dimethylsilyl; X = Cl, **2**; OTf, **3**)

Complexes **2** and **3** have been characterized by multinuclear NMR spectroscopy, high-resolution mass spectrometry (HR-MS), and elemental analysis (Figures S16–S32). The ^1^H NMR spectra (CD_2_Cl_2_) of complexes **2** and **3** exhibit a characteristic doublet of doublets integrating for one proton at δ – 17.09 ppm (^1^ *J*_H–Rh_ = 25.8 Hz, ^2^ *J*_H–P_ = 22.3 Hz) and δ – 21.77 ppm (^1^ *J*_H–Rh_ = 33.6 Hz, ^2^ *J*_H–P_ = 23.7 Hz), respectively. These signals confirm the presence of a Rh–H bond in both species and are consistent with values reported for related Rh–(κ^2^-NSi^tBu^) hydride complexes (NSi^tBu^ = {4-methylpyridin-2-yloxy}­ditertbutylsilyl).^34^ The ^29^Si­{^1^H} NMR spectra of complexes **2** and **3** in CD_2_Cl_2_ show doublet of doublets resonances at δ 76.9 ppm (^1^ *J*_Si–Rh_ = 40.4 Hz, ^2^ *J*_Si–P_ = 14.4 Hz) and δ 77.7 ppm (^1^ *J*_Si–Rh_ = 40.7 Hz, ^2^ *J*_Si–P_ = 13.1 Hz), respectively. These values are high-field shifted relative to the ^29^Si chemical shift observed for related Rh–(κ^2^-NSi^tBu^) complexes (δ 78.7 and 87.9 ppm).^34^ Furthermore, the ^31^P­{^1^H} NMR spectra (CD_2_Cl_2_) show doublet resonances at δ 51.8 ppm (^1^ *J*_P–Rh_ = 142.1 Hz; **2**) and δ 49.2 ppm (^1^ *J*_P–Rh_ = 139.0 Hz; **3**).

The solid-state structure of **3** has been determined by single-crystal X-ray diffraction analysis. As illustrated in Figure 1, considering the five well-defined metal–ligand bonds, complex **3** exhibits a slightly distorted square pyramidal geometry with a τ value of 0.06 (τ ranges from 0 for an ideal square pyramidal geometry to 1 for an ideal trigonal bipyramid).^35^ The silicon atom occupies the apical position, while the nitrogen atom is located *trans* to the phosphorus atom, as summarized in Table 1. In addition to these five primary interactions, and consistently with observations in analogous systems, the metal center engages in an agostic interaction with a hydrogen atom from a methyl substituent, occupying the coordination site located *trans* to the silicon atom. The observed Rh···H and Rh···C distances and the Rh···H–C angle (2.24(3) Å, 2.7257(17) Å, and 111.2(17)°, respectively) compare well with values reported in the literature for agostic Rh···H–C interactions.^36^ When this weak agostic interaction is taken into account as a donor, the coordination environment may alternatively be viewed as a distorted octahedral Rh­(III) system. The Rh–Si bond distance of 2.2548(5) Å in **3** is shorter than the value of 2.2689(3) Å reported for the related species [Rh­(H)­(OTf)­(κ^2^-NSi^tBu^)­(PCy_3_)].^34^

**Figure 1:**
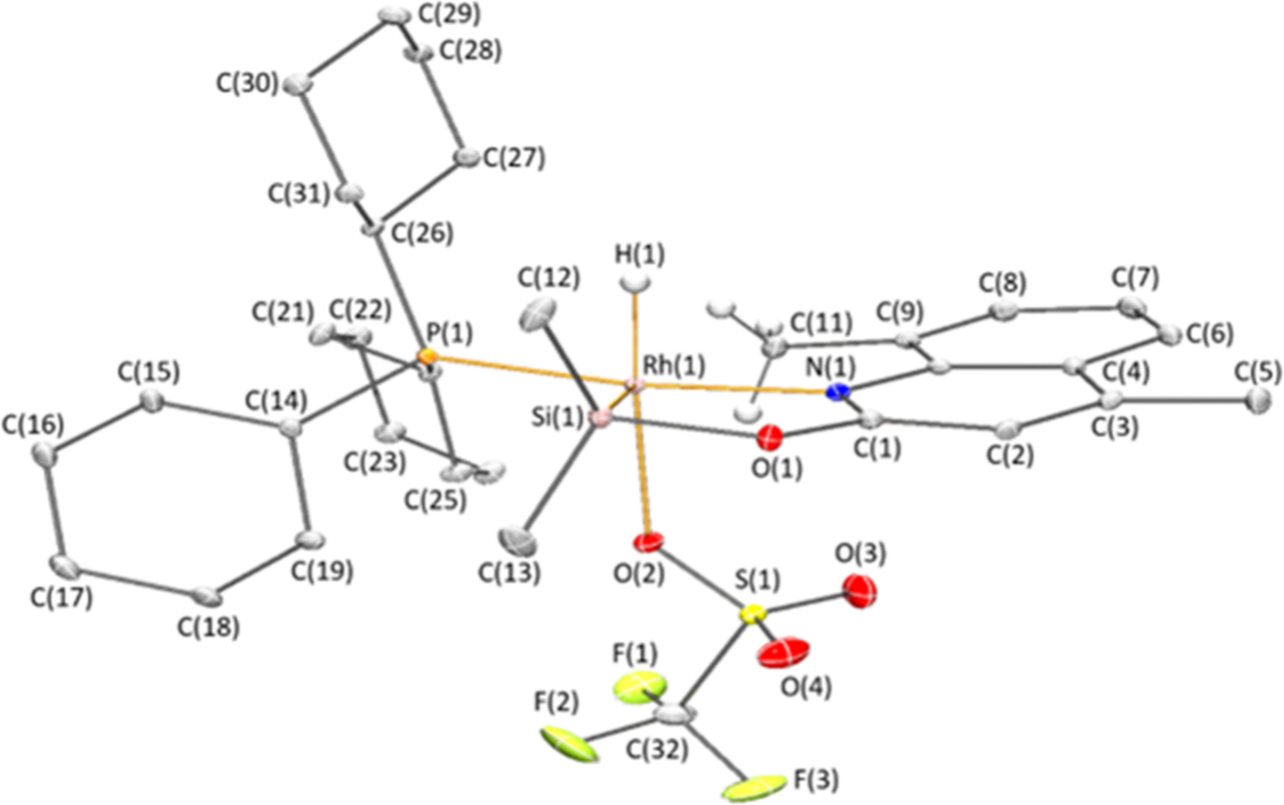
Molecular structure of complex **3**. Hydrogen atoms (except hydride) have been omitted for clarity.

**Table 1:** Selected Bond Lengths (Å) and Angles (°) for Complex **3**

Rh(1)–P(1)	2.2859(4)	P(1)–Rh(1)–H(1)	85.8(10)
Rh(1)–Si(1)	2.2548(5)	Si(1)–Rh(1)–O(2)	95.69(4)
Rh(1)–O(2)	2.2528(12)	Si(1)–Rh(1)–N(1)	83.50(4)
Rh(1)–N(1)	2.1136(13)	Si(1)–Rh(1)–H(1)	83.1(10)
Rh(1)–H(1)	1.45(3)	O(2)–Rh(1)–N(1)	88.07(5)
P(1)–Rh(1)–Si(1)	100.609(15)	O(2)–Rh(1)–H(1)	176.1(10)
P(1)–Rh(1)–O(2)	98.08(3)	N(1)–Rh(1)–H(1)	88.1(10)
P(1)–Rh(1)–N(1)	172.17(4)		

^1^H–^29^Si HMQC NMR experiments of CD_2_Cl_2_ solutions of complexes **2** and **3** show a clear correlation between the silicon atom and the 8-Me protons of the NSi^DMQ^ ligand (Figures S22 and S30). This observation, along with lower ^1^ *J*_C–H_ values observed in the ^13^C NMR spectra for the 8-Me fragment (124.4 Hz in **2**; 120.7 Hz in **3**) compared to 4-Me substituent (127.9 Hz in **2**; 128.2 Hz in **3**), supports the presence of a weak Rh···H–C agostic interaction (Figures S31 and S32).^8,37^

Density Functional Theory (DFT) calculations (B3LYP^38^-GD3 ^39^/def2-TZVP^40^) further confirm the presence of a weak Rh···H–C agostic interaction, with calculated Rh···H distances of 2.159 and 2.147 Å for **2** and **3**, respectively. Quantum Theory of Atom in Molecules (QTAIM)^41^ analysis reveals a (3,–1) bond critical point (BCP) and bond paths (BP) between the Rh and H atoms, confirming the agostic interaction in **2** and **3** (Figure 2 and Table 2). The agostic interaction in these complexes was also examinated by Natural Bond Orbital (NBO)^42^ analysis. The second order stabilization energy (Δ*E* ^(2)^) obtained for the donation of electron density from an occupied σ­(C–H) orbital to an empty Rh d-orbital is similar in **3** (−9.51 kcal·mol^–1^) and **2** (−9.45 kcal·mol^–1^), inidicating comparable donor–acceptor interactions. Therefore, the presence of the Rh···H–C agostic interaction in **2** and **3** is supported by both experimental observations and theoretical calculations.

**Figure 2:**
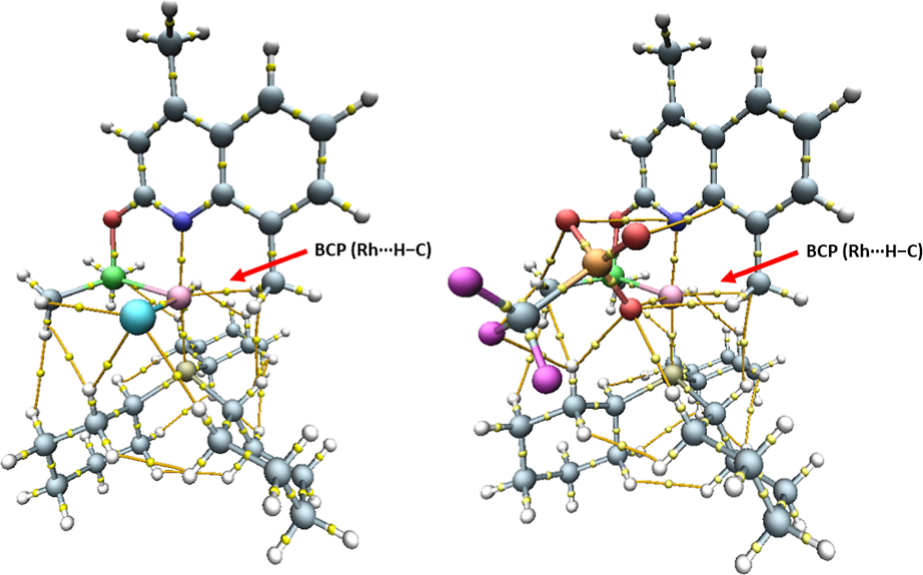
Contour line diagrams ∇^2^ρ­(r) for complexes **2** (left) and **3** (right) in the Rh···H–*C* plane. Yellow spheres denote the located bond critical points (BCP).

**Table 2:** Results of the QTAIM and NBO Analysis for **2** and **3**

	**2**	**3**
r(Rh···H)/Å	2.159	2.147
ρ(r)/e·Å^–3^	0.033	0.034
∇^2^ ρ(r)/e·Å^–5^	0.108	0.113
ε	0.341	0.371
Δ*E* ^(2)^/σ(C–H) → δ(Rh) and δ(Rh) → σ*(C–H) kcal·mol^–1^	–9.45	–9.51
Δ*E* ^(2)^/LP (O) → LV (Si) kcal·mol^–1^	–137.59	–139.23

Beyond the characterization of the Rh···H–C agostic interaction, the electronic structure of complexes **2** and **3** was further analyzed to gain insight into the nature of the Rh–Si bond. In this context, it has recently been reported that the TM–Si bond in related Ir–(κ^2^-NSi) complexes with 2-quinolone based ligands is better described as a base-stabilized silylene bond than as a silyl bond (Figure 3).^43^ It was found that, to elucidate the nature of the TM–Si bond in complexes of this kind, it is important to analyze the interaction between the Si and the O atoms of the κ^2^-NSi ligand. The NBO studies of complexes **2** and **3** revealed a dative O→Si interaction with second-order stabilization energies, Δ*E* ^(2)^, of −137.59 kcal·mol^–1^ (**2**) and −139.23 kcal·mol^–1^ (**3**) (Table 2). These data along with the values of the Wiberg Bond Index (WBI),^44^ electron density ρ­(*r*), Laplacian of electron density ∇^2^ρ­(*r*), and the ratio between electronic potential and kinetic energies |*V*(*r*)|/*G*(*r*) at the BCP obtained from the QTAIM analysis for the Rh–Si and Si–O bonds (Tables S7 and S8) support the description of both species as base-stabilized Rh–silylene complexes.^43^

**Figure 3:**
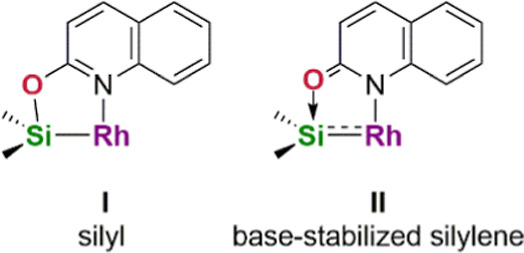
Representation of the Rh–silyl and base-stabilized TM–silylene tautomers.

### Rh–(κ^2^-NSi) Catalyzed CDC of Secondary Amines with Hydrosiloxanes

With complexes **2** and **3** fully synthesized and characterized, we next evaluated their catalytic activity using the reaction of pyrrolidine with hydrosilanes as a benchmark. NMR (C_6_D_6_) scale reactions of pyrrolidine with HSiMe_2_Ph catalyzed by **2** and **3** (1.0 mol %) at r.t., indicate that while complex **3** is active, no product formation was observed when the related chloride derivative **2** was used as catalyst (Table 3, entries 1 to 5). For comparison purposes, data for the related iridium species [Ir­(H)­(OTf)­(κ^2^-NSi^DMQ^)­(PCy_3_)] (**4**), which is known to be highly active in catalyzing this reaction under neat conditions,^8^ were also included in Table 3. These data show that **4** affords 92% conversion to the corresponding silylpyrrolidine derivative after 3 h at r.t. under the same reaction conditions (Table 3, entry 6). Complex **3** exhibits lower activity than its Ir counterpart. To achieve comparable reactivity, the reaction temperature must be increased to 333 K, affording 90% conversion to the corresponding silazane after 3 h (Table 3, entry 10). Reactions of pyrrolidine with related hydrosilanes, HSiMePh_2_ (Table 3, entry 11) and HSiEt_3_ (Table 3, entry 12), in C_6_D_6_ at 333 K in the presence of 1.0 mol % of **3** confirm its reduced activity relative to its iridium analogue **4** (Table 3 entries 8 and 9).

To our surprise, rhodium complex **3** displays unique reactivity with the hydrosiloxane HSiMe­(OSiMe_3_)_2_ (HMTS), achieving 99% conversion in 3 h at 298 K (Table 3, entry 5). Notably, the iridium counterpart **4** is significantly less active under the same conditions (Table 3, entry 7). To the best of our knowledge no previous examples of Ir– or Rh–catalyzed CDC of amines with hydrosiloxanes have been reported until now, highlighting the potential of **3** as catalyst for the synthesis of siloxazanes by CDC.

Therefore, the results summarized in Table 3 demonstrate that complex **3** is active toward both hydrosilanes and the hydrosiloxane HMTS. Notably, it exhibits markedly higher activity with the hydrosiloxane HMTS than its iridium analogue **4**. Considering the greater steric hindrance around the Si–H bond in the hydrosiloxane, this enhanced reactivity of complex **3** is most plausibly governed by electronic rather than steric effects.

Data shown in Table 3 reveal a positive effect of temperature on the reaction rate (Table 3, entries 10 to 13). To gain insight on the reaction performance, we studied the solventless **3**-catalyzed (1.0 mol %) CDC of pyrrolidine with HSiMe­(OSiMe_3_)_2_ in a microreactor equipped with a pressure sensor (*Man on the Moon* microreactor),^45^ evaluating the process at different temperatures. These studies revealed a clear positive correlation between the temperature and the reaction rate in the range of 298 to 333 K; however, upon heating at 343 K catalyst decomposition was observed (Figure S1).

**Table 3:**
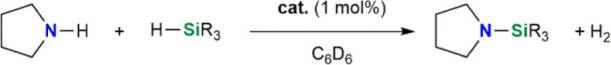
Comparison of the Activity of **2**, **3** and **4** (1.0 mol %) as Catalysts for the CDC of Pyrrolidine (0.3 mmol) with Different Hydrosilanes (0.3 mmol) and HSiMe­(OSiMe_3_)_2_ (0.3 mmol) in C_6_D_6_ (0.4 mL)

entry	cat.	HSiR_3_	time (h)	T (K)	product	conver. (%)^a^
1	**2**	HSiMe_2_Ph	3	298	**5a**	n.d
2	**2**	HMTS^b^	3	298	**8a**	n.d
3	**3**	HSiMe_2_Ph	3	298	**5a**	10.0
4	**3**	HMTS^b^	0.5	298	**8a**	47.0
5	**3**	HMTS^b^	3	298	**8a**	>99.0
6^7^	**4**	HSiMe_2_Ph	3	298	**5a**	92.0
7^7^	**4**	HMTS^b^	3	298	**8a**	10.0
8^7^	**4**	HSiMePh_2_	3	298	**6a**	38.0
9^7^	**4**	HSiEt_3_	3	298	**7a**	1.0
10	**3**	HSiMe_2_Ph	3	333	**5a**	90.0
11	**3**	HSiMePh_2_	3	333	**6a**	54.0
12	**3**	HSiEt_3_	3	333	**7a**	n.d
13	**3**	HMTS^b^	0.5	333	**8a**	>99.0

a Conversion based on ^1^H NMR integration using hexamethylbenzene as internal standard (IS).

b HMTS = HSiMe­(OSiMe_3_)_2;_ n.d. = not detected.

Once 333 K was stablished as the optimal reaction temperature under neat conditions, a comparative study was performed to evaluate the reactivity of different hydrosilanes. The **3**-catalyzed (1.0 mol %) solventless reactions of pyrrolidine with HSiMe_2_Ph, HSiMePh_2_, HSiEt_3_, and HSiMe­(OSiMe_3_)_2_, were conducted. Results obtained from these experiments confirmed the trends observed in the ^1^H NMR (C_6_D_6_) studies. The silazanes C_4_H_8_N–SiMe_2_Ph (**5a**)^8^ and C_4_H_8_N-SiMePh_2_, (**6a**)^8^ were identified by comparison of their ^1^H and ^29^Si­{^1^H} NMR spectra with the reported data.^8^ The system showed higher turnover frequency (TOF) for HSiMe_2_Ph (TOF_10 min_ = 370 h^–1^, **5a**) and HSiMe­(OSiMe_3_)_2_ (TOF_10 min_ = 260 h^–1^, **8a**) than for HSiMePh_2_ (TOF_10 min_ = 50 h^–1^, **6a**), and no detectable activity for HSiEt_3_ (Figure 4). Thus, the higher reactivity observed for HSiMe­(OSiMe_3_)_2_ with rhodium complex **3** may be related to the greater polarization of its Si–H bond, although steric accessibility around the Si center may also contribute.

**Figure 4:**
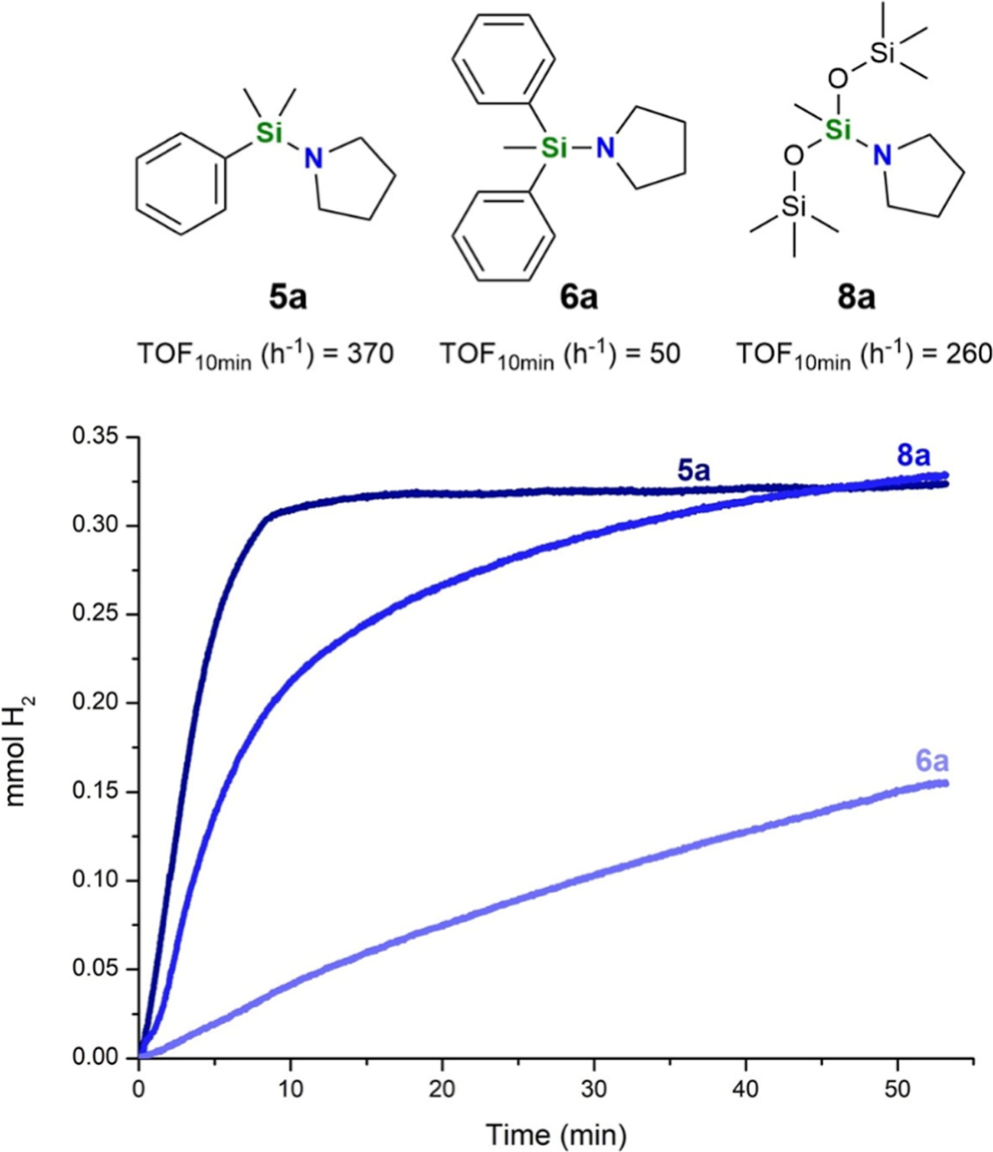
Time profile of H_2_ (mmol) generation from the **3**-catalyzed (1.0 mol %) solventless reaction of pyrrolidine with HSiMe_2_Ph, HSiMePh_2_ and HSiMe­(OSiMe_3_)_2_ (HMTS) at 333 K.

To investigate the hydrosiloxanes scope, the solvent-free catalytic reactions of pyrrolidine with pentamethyldisiloxane (HSiMe_2_(OSiMe_3_); PMDS), heptamethyltrisiloxane (HSiMe­(OSiMe_3_)_2_; HMTS) and tris­(trimethylsiloxy)­silane (HSi­(OSiMe_3_)_3_; TTMS) were performed at 333 K in the presence of catalytic amounts of **3** (1.0 mol %), and the generated hydrogen pressure was measured (Figure 5).

**Figure 5:**
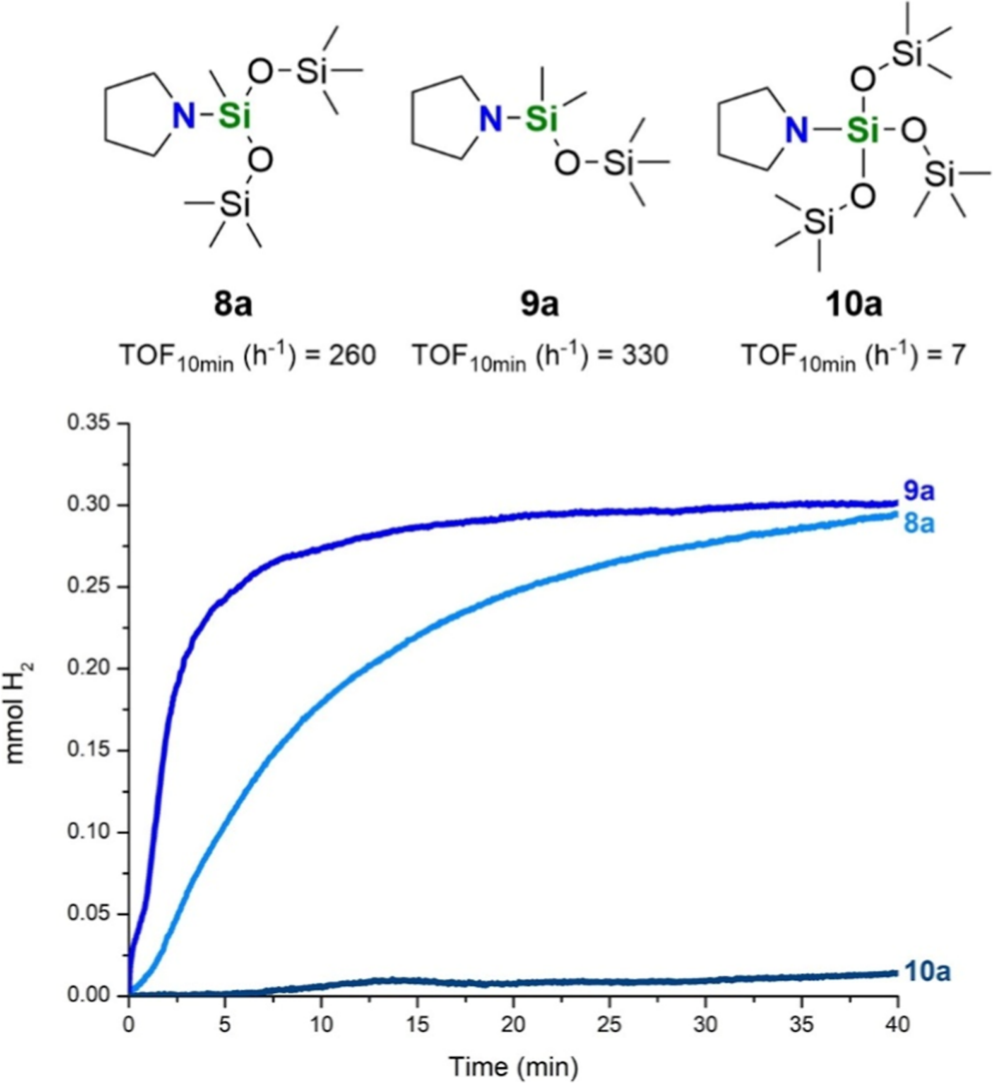
Time profile of H_2_ (mmol) generation from the **3**-catalyzed (1.0 mol %) reaction of pyrrolidine (0.5 mmol) with different hydrosiloxanes (0.5 mmol) at 333 K under neat conditions.

Among the hydrosiloxanes examined, PMDS exhibited the highest reactivity, followed by HMTS. In contrast, TTMS, despite its highly polarized Si–H bonds, displayed markedly lower reactivity, likely due to steric congestion around the silicon atom. These results indicate that in this system, steric effects prevail over electronic factors in determining reactivity.

Following this methodology, the siloxazane C_4_H_8_N–SiMe­(OSiMe_3_)_2_ (**8a**) was isolated as a yellow oil in 72% yield and characterized by HR-MS and multinuclear NMR spectroscopy. The ^1^H–^29^Si HMQC NMR spectrum (60 MHz, 298 K, C_6_D_6_) of **8a** shows two singlet resonances at δ 6.0 ppm and −47.8 ppm, assigned to the O*Si*Me_3_ and the N*Si*Me groups, respectively.

Analogously, the siloxazane C_4_H_8_N–SiMe_2_(OSiMe_3_) (**9a**) was successfully isolated as a yellow oil in 78% yield and characterized by HR-MS and multinuclear NMR spectroscopy. The ^1^H–^29^Si HMQC NMR spectrum (60 MHz, 298 K, C_6_D_6_) of **9a** shows two silicon resonances at δ 5.0 (O*Si*Me_3_) and −13.1 (N*Si*Me_2_), consistent with the proposed formulation. Unfortunately, due to the low efficiency of the reaction under these conditions, the corresponding siloxazane C_4_H_8_N–Si­(OSiMe_3_)_3_ (**10a**) could not be isolated.

Although PMDS proved to be the most reactive hydrosiloxane, the gain in reactivity over HMTS was relatively modest (Figure 5). Considering its significantly higher volatility (bp ≈ 85 °C vs 142 °C for HMTS) and cost, HMTS was selected as the optimal compromise between reactivity and practicality. Therefore, we decided to investigate the scope of the reaction with various secondary amines using HMTS as hydrosiloxane in the presence of catalytic amounts of **3** (1.0 mol %). We explored the performance of **3** as catalyst for the CDC of various amines and aniline derivatives with HSiMe­(OSiMe_3_)_2_ at 333 K under neat conditions. In any case, reactions with aliphatic amines showed higher activity compared to those with aniline derivatives (Figure 6 and Table 4). This implies that the nucleophilic character of the amine is relevant and suggests that coordination of the amine to the metal center may be a key step in the mechanism. The observed catalytic performance is also influenced by steric hindrance around the N–H bond as evidenced by the different formation rate of siloxazanes **8d** and **8e**.

**Figure 6:**
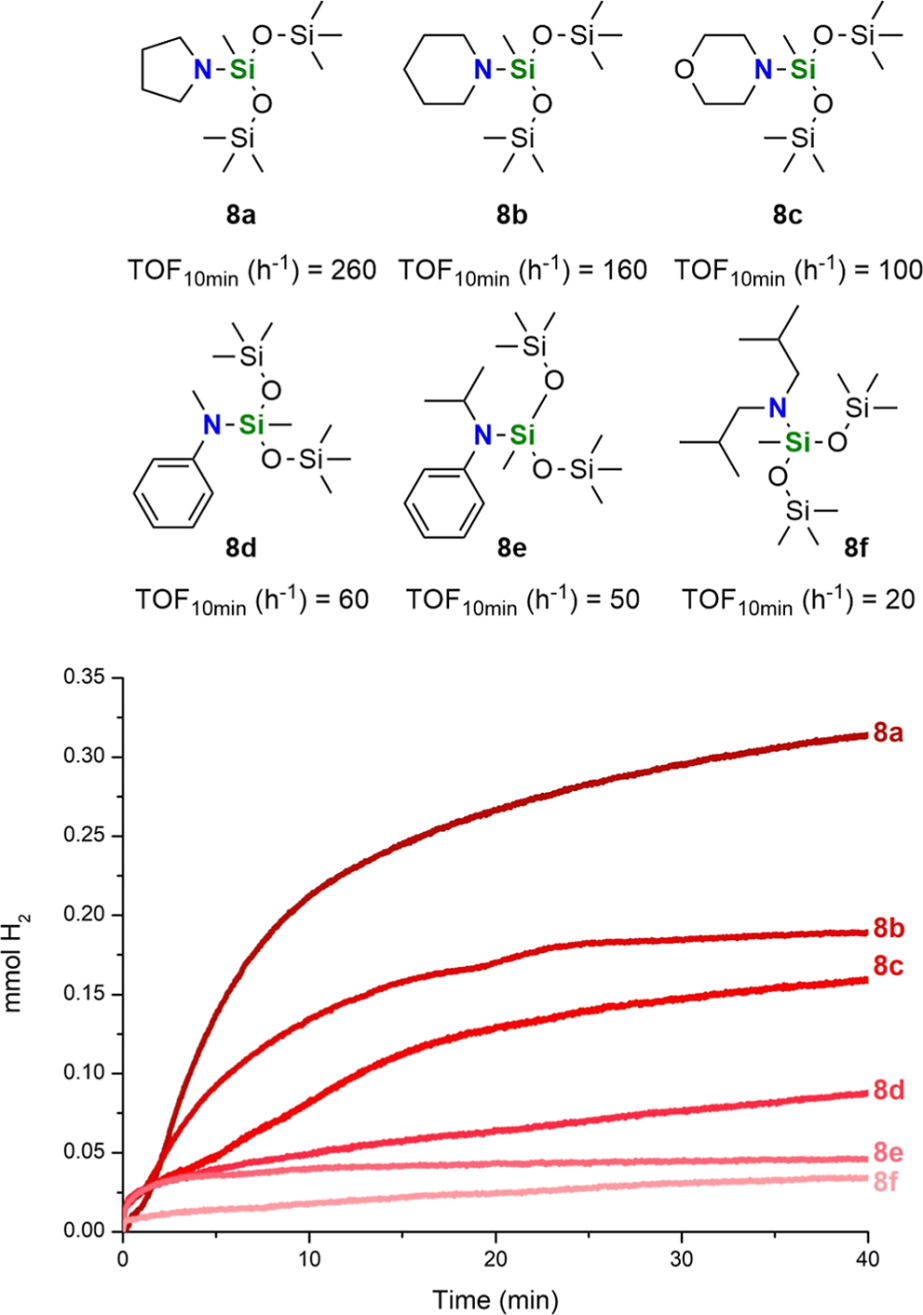
Time profile of H_2_ (mmol) generation from the **3**-catalyzed (1.0 mol %) solventless reaction of secondary amines and aniline derivatives (0.5 mmol) with HSiMe­(OSiMe_3_)_2_ (0.5 mmol) at 333 K.

**Table 4:**
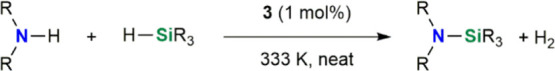
Comparison of the Performance of the **3**-Catalyzed (1.0 mol %) Reaction of Different Secondary Amines (0.5 mmol) with Different Hydrosiloxanes (0.5 mmol) at 333 K under Neat Conditions after 24 h

entry	siloxane	amine	product	conv. (%)	yield (%)^c^
1	HMTS	pyrrolidine	**8a**	99^a^	92
2	PMDS	pyrrolidine	**9a**	98^a^	78
3	TTMS	pyrrolidine	**10a**	<1^b^	-
4	HMTS	piperidine	**8b**	94^a^	80
5	HMTS	morpholine	**8c**	91^a^	80
6	HMTS	*N*–Me-aniline	**8d**	83^a^	76
7	HMTS	*N*–^i^Pr-aniline	**8e**	<1^b^	-
8	HMTS	*N*,*N*-diisobutylamine	**8f**	<1^b^	-

a Conversion calculated by ^1^H NMR using hexamethylbenzene as IS.

b Conversion calculated based on the hydrogen generation.

c Isolated yield.

The ^1^H NMR spectrum (C_6_D_6_) of the crude reaction mixtures, recorded using hexamethylbenzene as internal standard, revealed the formation of siloxazanes **8b**, **8c** and **8d** in 94%, 91% and 83% respectively (Table 4). Compounds **8b**, **8c** and **8d** have been characterized by means of HR-MS and multinuclear NMR spectroscopy. The ^1^H–^29^Si HMQC NMR spectrum (60 MHz, 298 K, C_6_D_6_) of **8b**, **8c** and **8d** shows two singlet resonances at δ 6.0 ppm and −49.2 ppm (**8b**), δ 7.1 ppm and −49.1 ppm (**8c**) and δ 8.0 ppm and −48.8 ppm (**8d**) which are assigned to the O*Si*Me_3_ and N*Si*Me groups, respectively, and are consistent with those observed for compound **8a**.

### Theoretical Studies

To elucidate the mechanism operating in the **3**-catalyzed CDC of secondary amines with HSiMe­(OSiMe_3_)_2_, DFT calculations were performed using the reaction of complex **3** (denoted as **INT1** in Figure 7) with pyrrolidine and HSiMe­(OMe)_2_ as a model system. The corresponding Gibbs free energy (kcal·mol^–1^) profiles are shown in Figures 7 and 8. The overall process can be divided into two steps: (i) activation of the catalytic precursor (Figure 7) and (ii) the catalytic cycle itself (Figure 8).

**Figure 7:**
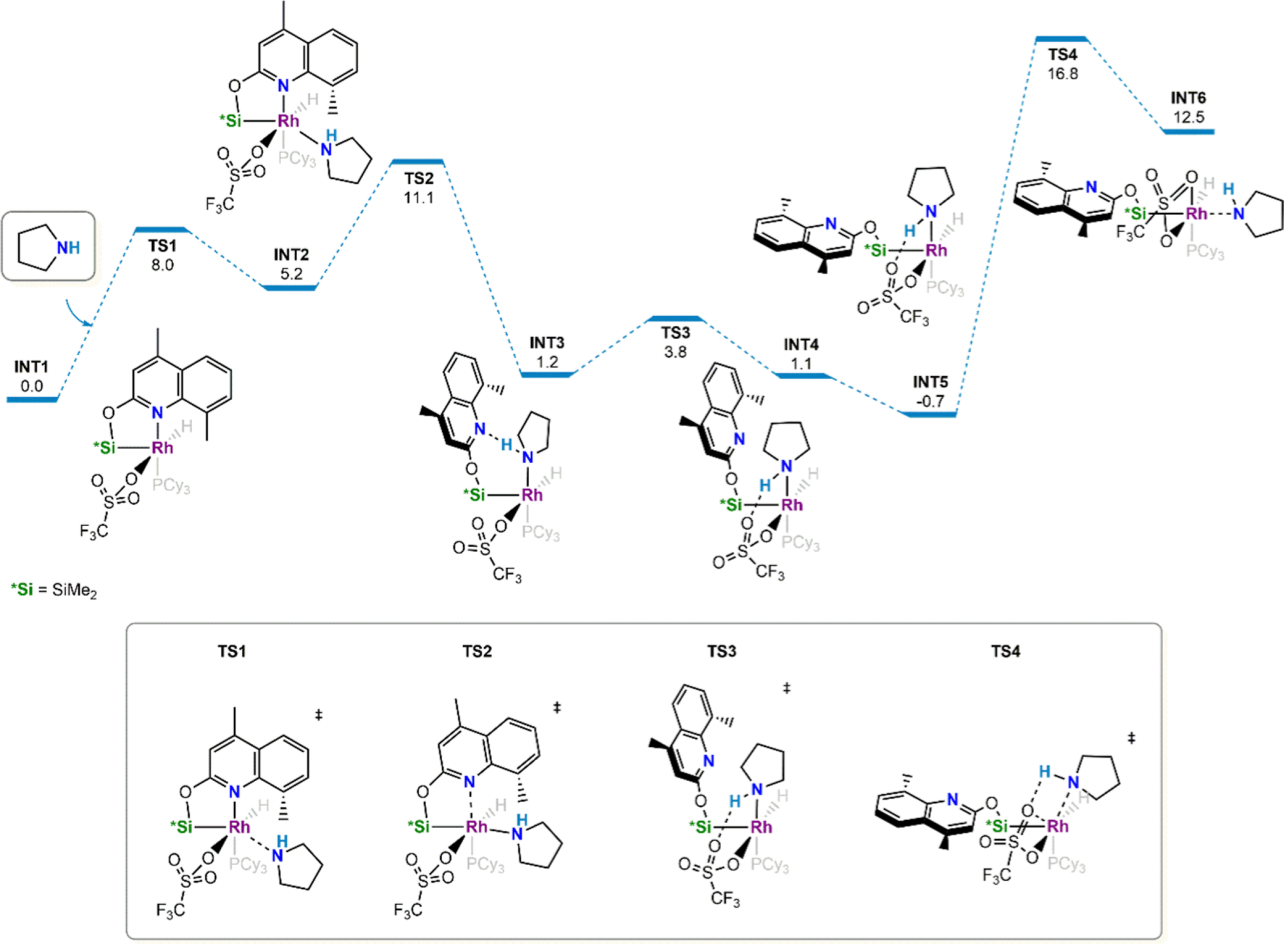
Gibbs free energy profile for the catalyst activation (in kcal·mol^–1^) relative to **INT1**.

**Figure 8:**
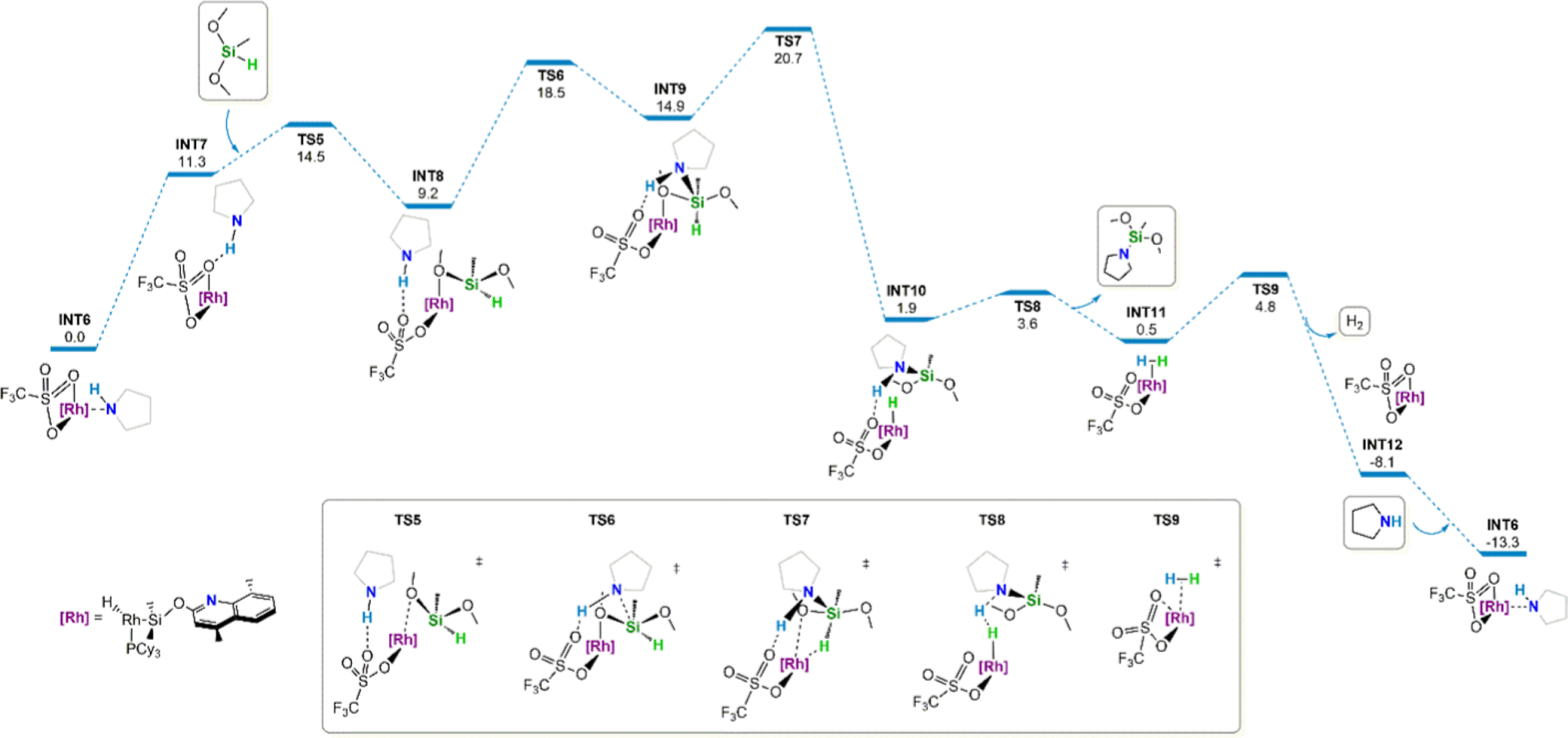
Gibbs free energy profile for the catalyst cycle (in kcal mol^–1^) relative to **INT6**.

Activation of the catalytic precursor (Figure 7) is initiated by *N*-coordination of pyrrolidine to the metal center (**INT1 INT2**). This step is slightly endergonic (Δ*G*_298_ = 5.2 kcal·mol^–1^) and proceeds via a low barrier (Δ*G* ^‡^_298_ = 8.0 kcal·mol^–1^, **TS1**), consistent with a rapid and reversible equilibrium at room temperature. Subsequent hemidissociation of the quinolone *N* donor atom of the bidentate κ^2^-NSi ligand affords **INT3** (Δ*G*_298_ = 1.2 kcal·mol^–1^), stabilized by an intramolecular N···H–N hydrogen bond, as supported by NBO analysis (Δ*E* ^(2)^ = 6.6 kcal·mol^–1^). This transformation is accompanied by a change in coordination geometry from octahedral to square-based pyramid via **TS2** (Δ*G* ^‡^_298_ = 11.1 kcal·mol^–1^). From **INT3**, rotation about the Rh–Si bond via **TS3** (Δ*G* ^‡^_298_ = 3.8 kcal·mol^–1^) gives intermediate **INT4**, in which a triflate oxygen atom engages in a stabilizing O···H–N interaction with the coordinated amine (Δ*E* ^(2)^ = 9.7 kcal·mol^–1^). A subsequent reorganization furnishes **INT5** (Δ*G*_298_ = – 0.7 kcal·mol^–1^).^46^ Further rearrangement enables a chelating interaction of the triflate ligand through two oxygen atoms, leading to the formation of the catalytic active species **INT6** (Δ*G*_298_ = 12.5 kcal·mol^–1^) via **TS4** (Δ*G* ^‡^_298_ = 16.8 kcal·mol^–1^). **INT6** can be described as a Rh­(III) species featuring a square-pyramidal environment, in which the triflate ligand adopts a κ^2^-*O*,*O*′ coordination mode and the coordination site *trans* to the silyl fragment is occupied by the amine supported by a weak Rh–N interaction (Δ*E* ^(2)^ = 12.2 kcal·mol^–1^). The overall activation barrier is therefore determined by **TS4** (Δ*G* ^‡^_298_ = 16.8 kcal·mol^–1^).

This proposed activation mechanism is consistent with the experimental observations. While ^1^H NMR (C_6_D_6_) experiments show that complex **3** does not react with HSiMe­(OSiMe_3_)_2_, the ^1^H NMR spectra of solutions of **3** in C_6_D_6_ change instantaneously upon addition of pyrrolidine to give a mixture of unidentified Rh–H species. These observations suggest that the initial catalyst activation is promoted by the amine.

Once the activation process has been established, the catalytic cycle was investigated starting from the active catalytic species **INT6** (Figure 8). Cleavage of the Rh–N interaction in **INT6** leads to an unstable 14e intermediate **INT7** (Δ*G*_298_ = 11.3 kcal·mol^–1^), in which pyrrolidine is supported by the interaction with the triflate anion. Then, coordination of the siloxane to the metal center *trans* to the phosphine ligand through one of the oxygen atoms leads to the formation of **INT8** (Δ*G*_298_ = 9.2 kcal·mol^–1^) via **TS5** (Δ*G* ^‡^_298_ = 14.5 kcal·mol^–1^). Then, formation of the Si–N bond occurs through **TS6** (Δ*G* ^‡^_298_ = 18.5 kcal·mol^–1^) to give **INT9**, an unstable intermediate (Δ*G*_298_ = 14.9 kcal·mol^–1^), which evolves to **INT10** (Δ*G*_298_ = 1.9 kcal·mol^–1^) via **TS7**. This transformation corresponds to the highest energy barrier of the overall process (Δ*G* ^‡^_298_ = 20.7 kcal·mol^–1^) and implies cleavage of the interaction between the rhodium center and the oxygen atom of the silane, together with hydride migration from silicon to rhodium. **INT10** is preorganized for 1,2 proton shift from the N atom of the pyrrolidine to Rh and formation of a Rh–dihydrogen species **INT11** and siloxazane via **TS8** (Δ*G* ^‡^_298_ = 3.6 kcal·mol^–1^). The change of the coordination mode of the triflate ligand from κ^1^-*O* to κ^2^-*O*,*O*′ through **TS9** (Δ*G* ^‡^_298_ = 4.8 kcal·mol^–1^) favors the hydrogen release to give **INT12**. Finally, approaching of pyrrolidine to **INT12** regenerates the active species **INT6**.

Overall, the catalytic cycle is thermodynamically favorable by 13.3 kcal·mol^–1^. The kinetic of the catalytic process is governed by two closely related energy barriers: **TS6** (Δ*G* ^‡^_298_ = 18.5 kcal·mol^–1^) and **TS7** (Δ*G* ^‡^_298_ = 20.7 kcal·mol^–1^), corresponding to Si–N bond formation and hydride migration from silicon to rhodium, respectively (Figure 8). These barriers are consistent with the corresponding Arrhenius analysis, which yields an apparent activation energy of 15.6 ± 1.8 kcal·mol^–1^ (Figure S3), as well as with the Eyring analysis of the kinetic data, which provides activation parameters of Δ*H* ^‡^ = 15.1 ± 1.9 kcal·mol^–1^ and Δ*S* ^‡^ = – 15.4 ± 5.9 cal·K^–1^·mol^–1^. These values correspond to an activation free energy of Δ*G* ^‡^ = 19.6 ± 0.1 kcal·mol^–1^ at 298 K (Figure S4).

Prompted by the observed effect of the triflate ligand on the catalytic activity (Table 3, entries 1–5), we investigated the effect of replacing it with a less electron-withdrawing ligand. Calculations using methylsulfonate instead of triflate revealed higher activation barriers relative to the corresponding **INT6-H** intermediate (Δ*G* ^‡^_298_ = 24.9 and 28.1 kcal·mol^–1^ for **TS6-H** and **TS7-H**, respectively). These values are higher than those found for the triflate system, where the corresponding barriers from **INT6** are 18.5 and 20.7 kcal·mol^–1^, for **TS6** and **TS7**, respectively (Figure S12).

Although stoichiometric experiments were performed, direct experimental evidence for the proposed mechanism remains limited. The observation of multiple intermediate species, which could not be fully characterized, together with hydrogen evolution during the catalytic reaction complicate intermediate determination by NMR. Therefore, several plausible pathways of evolution were investigated by theoretical calculations, including: (a) elimination of the proligand **1** (Figure S13); (b) elimination of Si–OTf (Figure S14); (c) elimination of the PCy_3_H^+^ fragment (Figure S15). All these alternative pathways were found to involve higher activation barriers (21.8 **TS-I**; 26.0 **TS-II** and 30.4 **TS-III** kcal·mol^–1^, respectively) than that calculated for **TS4** (16.4 kcal·mol^–1^) (Figure 9). Therefore, while alternative mechanistic scenarios cannot be completely ruled out, the mechanism depicted in Figure 8 represent a plausible pathway consistent with the available experimental (Δ*G* ^‡^ = 19.6 ± 0.1 kcal·mol^–1^ at 298 K) and computational data. However, these mechanisms (Figures S13–S15) could operate at high temperature or when reagents are consumed and the main proposed catalytic cycle cannot operate.

**Figure 9:**
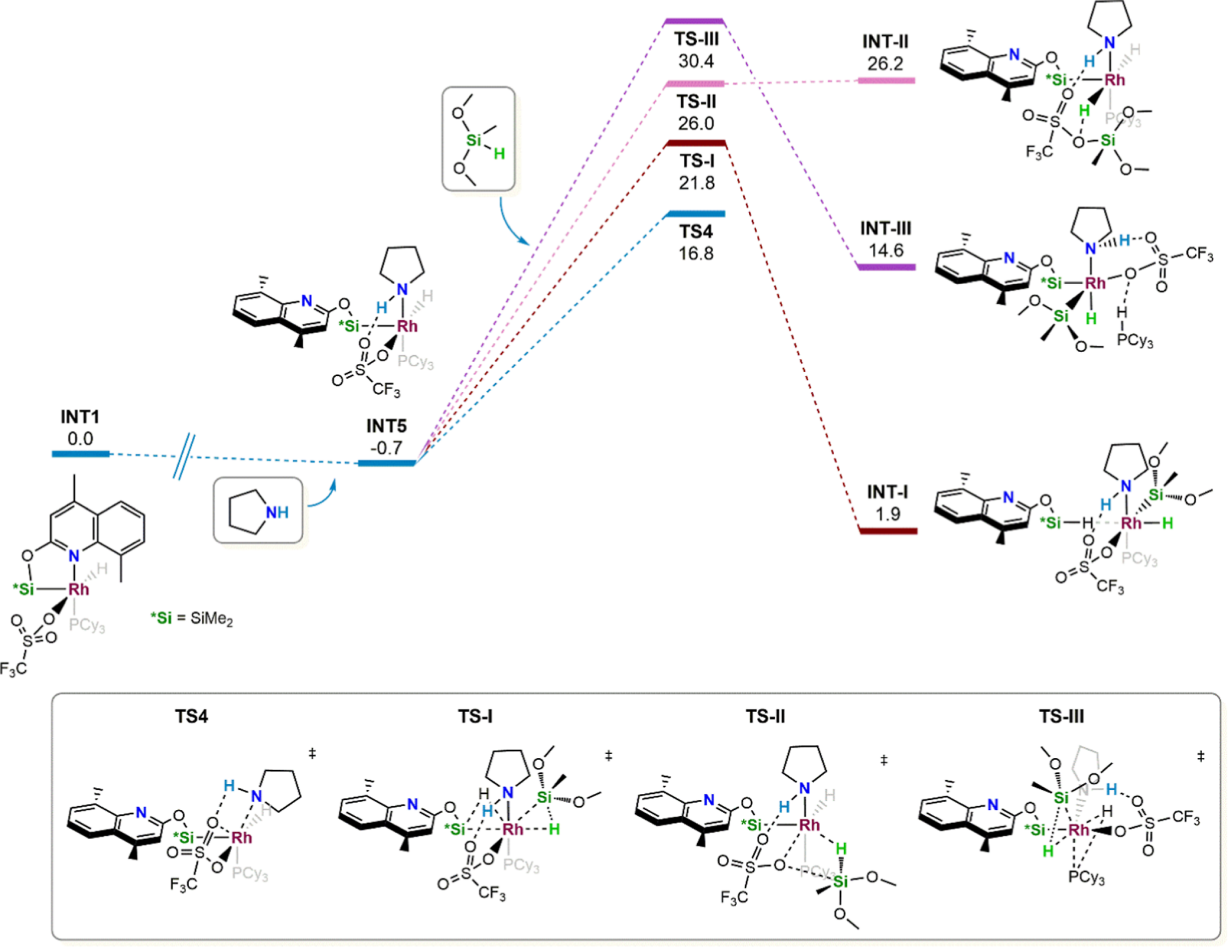
Gibbs free energy (in kcal·mol^–1^ relative to **INT1**) comparison for different reaction pathways from **INT5**.

On the basis of these DFT calculations, together with the kinetic and reactivity studies described above, a simplified catalytic cycle is proposed (Scheme 3). The mechanism is initiated by coordination of the amine to complex **3**, followed by hemidissociation of the κ^2^-NSi^DMQ^ ligand to generate the catalytically active species (**INT6**). Then, κ^1^-*O* coordination of the siloxane to the metal center anchors the substrate, bringing the Si atom into proximity with the N atom and facilitating hydride migration from the silicon to the metal. The triflate-supported pyrrolidine plays a dual and crucial role: it directs the amine toward the silicon center, promoting Si–N bond, and facilitates N–H bond activation to afford **INT10**. Release of the siloxazane product leads to a rhodium–dihydrogen intermediate (**INT11**), which upon H_2_ evolution and coordination of a new amine molecule, regenerates the active catalytic species **INT6**.

**Scheme 3:**
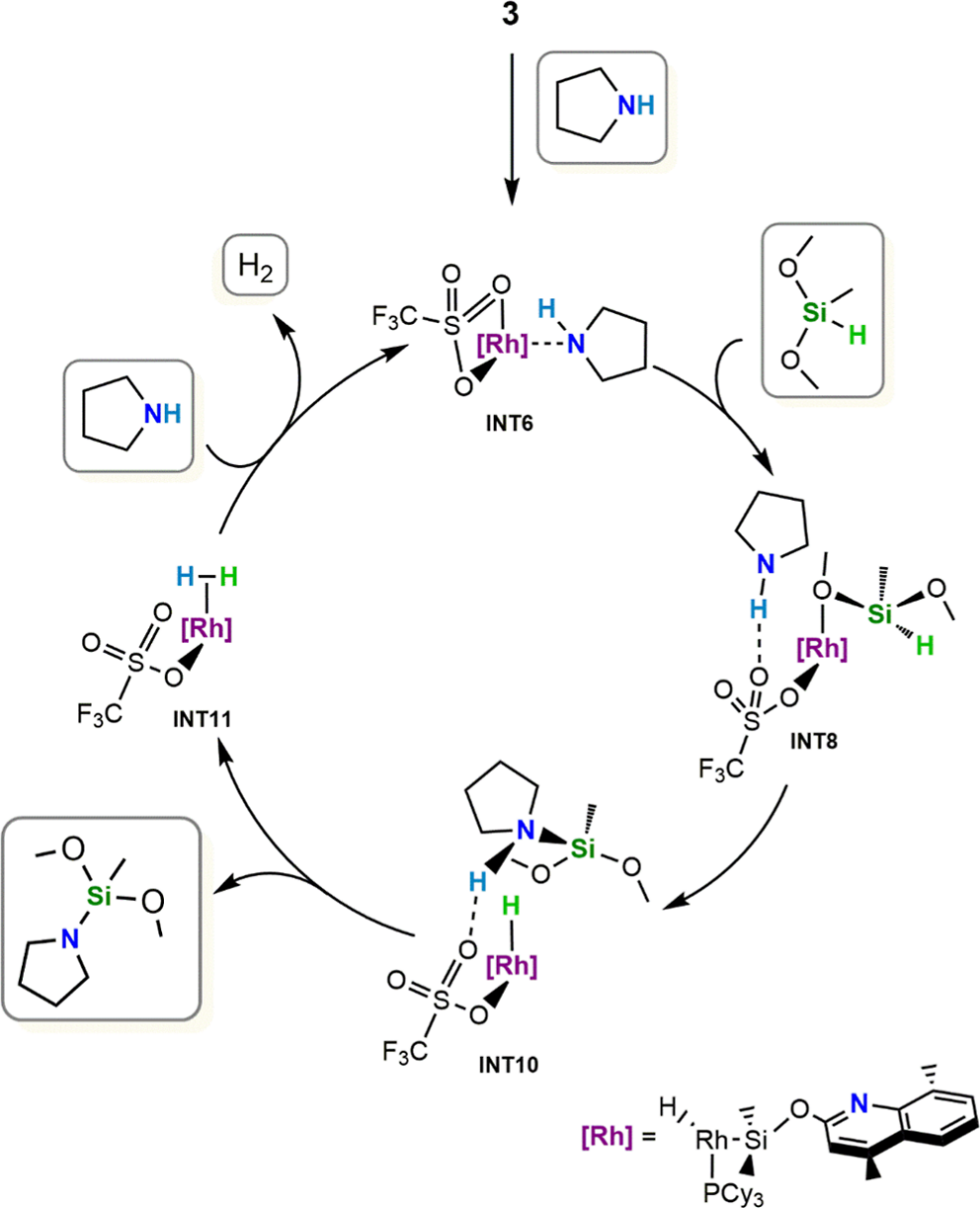
Proposed Simplified Catalytic Cycle for the **3**-Catalyzed Synthesis of Siloxazanes

## Conclusions

To the best of our knowledge, this work represents the first example of a rhodium-based system capable of catalyzing the CDC of secondary amines with hydrosiloxanes.

The complexes [Rh­(H)­(Cl)­(κ^2^-NSi^DMQ^)­(PCy_3_)] (**2**) and [Rh­(H)­(OTf)­(κ^2^-NSi^DMQ^)­(PCy_3_)] (**3**) were synthesized and fully characterized, with single-crystal X-ray diffraction analysis of complex **3**. Both complexes display weak but discernible Rh···H–C agostic interactions, confirmed by both experimental and theoretical results. This interaction is slightly more pronounced in the triflate derivative **3**.

Catalytic investigations demonstrate a marked anion effect, with the triflate derivative **3** showing high activity in the Si–N bond-forming CDC of secondary amines with hydrosiloxanes, whereas the chloride analogue **2** is inactive. Complex **3** displays excellent performance, particularly with HSiMe­(OSiMe_3_)_2_ (HMTS), enabling efficient and selective transformations under mild and solvent-free conditions, and outperforming the corresponding iridium system.

DFT calculations suggest that the process comprises two stages: (i) activation of the catalytic precursor, involving coordination of the amine and hemidissociation of the κ^2^-NSi^DMQ^ ligand in complex **3** to generate the active species, and (ii) the catalytic cycle. In the latter, κ^1^-*O* coordination of the siloxane anchors the substrate to the metal center, while the triflate-supported pyrrolidine plays a dual key role by directing the nucleophile toward the silicon center to promote Si–N bond formation and by facilitating subsequent N–H bond activation. Release of the siloxazane affords a rhodium–dihydrogen intermediate, and subsequent H_2_ evolution, followed by amine coordination, regenerates the active catalyst. The catalytic process is thermodynamically favorable and proceeds with moderate energy barrier, in agreement with experimental observations.

Overall, this work establishes a new platform for rhodium-catalyzed Si–N bond formation using hydrosiloxanes and provides mechanistic insights into the role of coordinating anions and ligand hemilability. These findings offer a basis for the development of improved catalytic systems for dehydrogenative Si–N bond-forming processes.

## Experimental Section

### General Information

All manipulations were performed under an argon atmosphere using Schlenk line or glovebox techniques, with rigorous exclusion of air. Solvents were dried by standard procedures and distilled under argon prior to use or obtained oxygen- and moisture-free from a Solvent Purification System (Innovative Technologies). ^1^H, ^13^C­{^1^H}, ^13^C, ^1^H–^13^C HSQC, ^1^H–^13^C HMBC, ^31^P­{^1^H}, ^1^H–^29^Si HMQC, ^29^Si­{^1^H} and ^19^F NMR spectra were recorded on Bruker Avance 300 MHz or Bruker Avance 400 MHz spectrometers. Coupling constants (*J*) are given in hertz (Hz) with multiplicity defined as: s = singlet, d = doublet, dd = double of doublets, ddd = double doublet of doublets, psd = pseudo doublet, m = multiplet, bs = broad signal. Compound **1** ^8^ and [{Rh­(coe)_2_}_2_(μ-Cl)_2_][Bibr ref33] were prepared following published procedures. Secondary amines and hydrosilanes were purchased from commercial sources and dried over 4 Å molecular sieves prior to use.

#### Synthesis of [Rh­(H)­(Cl)­(κ^2^-NSi^DMQ^)­(PCy_3_)] (2)

To a solution of [{Rh­(coe)_2_}_2_(μ-Cl)_2_] (311 mg, 0.43 mmol) in toluene (12 mL) was added a solution of (4,8-dimethylquinolin-2-yloxy)­dimethylsilane (200 mg, 0.86 mmol) in toluene (5 mL), followed by a solution of PCy_3_ (255 mg, 0.91 mmol) in toluene (5 mL). After 20 h of stirring at r.t., the reaction mixture was evaporated to dryness, and the residue was extracted with a 1:5 mixture of dichloromethane and hexane (5 mL). The resulting solution was cooled to 253 K and maintained at this temperature for 3 h, during which a white solid precipitated. The suspension was then filtered through Celite at low temperature, and the solid was collected to afford compound **2** as a white solid in 70% yield (391 mg, 0.60 mmol). ^1^H NMR (300 MHz, 298 K, CD_2_Cl_2_): δ 7.77 (m, 1H, *H* ^5^), 7.49 (d, ^3^ *J*_HH_ = 7.1 Hz, 1H, *H* ^7^), 7.32 (dd, ^3^ *J*_HH_ = 8.2 Hz, ^3^ *J*_HH_ = 7.1 Hz, 1H, *H* ^6^), 6.92 (br s, 1H, *H* ^3^), 3.23 (s, 3H, 8-C*H*_3_), 2.63 (d, 3H, ^4^ *J*_HH_ = 1.0 Hz, 4-C*H*_3_), 2.23–1.29 (overlapping signals, 33H, C*H*-PCy_3_ and C*H*_2_-PCy_3_), 0.84 (s, 3H, Si–C*H*_3_), 0.63 (s, 3H, Si–C*H*_3_), −17.09 (dd, ^2^ *J*_HP_ = 22.3 Hz, ^1^ *J*_HRh_ = 25.8 Hz, 1H, *H*–Rh). ^13^C­{^1^H} NMR (75 MHz, 298 K, CD_2_Cl_2_): δ 162.9 (d, ^2^ *J*_CRh_ = 2.6 Hz, *C* ^ipso–2^), 149.9 (s, *C* ^ipso–8a^), 145.8 (s, *C* ^ipso–4^), 133.5 (s, *C* ^ipso–8^), 132.8 (s, *C* ^7^), 126.5 (s, *C* ^ipso–4a^), 124.1 (s, *C* ^6^), 123.4 (s, *C* ^5^), 114.3 (s, *C* ^3^), 37.1 (d, ^1^ *J*_CP_ = 23.5 Hz, 3C, *C*H-PCy_3_), 31.2 (br s, 3C, *C*H_2_–PCy_3_), 30.3 (br s, 3C, *C*H_2_–PCy_3_), 30.2 (br s, 3C, *C*H_2_–PCy_3_), 28.3 (d, ^3^ *J*_CP_ = 10.3 Hz, 3C, *C*H_2_–PCy_3_), 27.1 (br s, 3C, *C*H_2_–PCy_3_), 19.7 (s, 4-*C*H_3_), 18.0 (d, *J*_CRh_ = 2.0 Hz, 8-*C*H_3_), 11.7 (d, ^2^ *J*_CRh_ = 5.5 Hz, Si–*C*H_3_), 8.2 (d, ^2^ *J*_CRh_ = 1.1 Hz, Si–*C*H_3_). ^29^Si NMR (60 MHz, 298 K, CD_2_Cl_2_): δ 76.9 (dd, ^1^ *J*_SiRh_ = 40.4 Hz, ^2^ *J*_SiP_ = 14.4 Hz, *Si*(CH_3_)_2_). ^31^P­{^1^H} NMR (121 MHz, 298 K, CD_2_Cl_2_): δ 51.8 (d, ^1^ *J*_RhP_ = 142.1 Hz, *P*Cy_3_). Anal. Calcd for C_31_H_50_ClNOPRhSi·0.2CH_2_Cl_2_: C, 56.17; H, 7.61; N, 2.10. Found: C, 56.54; H, 7.89; N, 2.27. HR-MS (ESI^+^, *m*/*z*): calcd for C_31_H_50_NOPRhSi, [M–Cl]^+^ = 614.2454; found, 614.2437.

#### Synthesis of [Rh­(H)­(OTf)­(κ^2^-NSi^DMQ^)­(PCy_3_)] (3)

CH_2_Cl_2_ (10 mL) was added to a mixture of compound **2** (300 mg, 0.46 mmol) and AgTfO (119 mg, 0.46 mmol) in the dark. After stirring for 15 h at r.t., the resulting suspension was filtered through Celite using a cannula. The solution was evaporated to dryness to afford compound **3** as a light brown solid in 73% yield (256 mg, 0.32 mmol). ^1^H NMR (300 MHz, 298 K, CD_2_Cl_2_): δ 7.83 (d, ^3^ *J*_HH_ = 8.2 Hz, 1H, *H* ^5^), 7.56 (d, ^3^ *J*_HH_ = 7.2 Hz, 1H, *H* ^7^), 7.37 (dd, ^3^ *J*_HH_ = 8.2 Hz, ^3^ *J*_HH_ = 7.2 Hz, 1H, *H* ^6^), 6.97 (br s, 1H, *H* ^3^), 3.26 (s, 3H, 8-C*H*_3_), 2.67 (br s, 3H, 4-C*H*_3_), 2.02–1.27 (overlapping signals, 33H, C*H*-PCy_3_ and C*H*_2_-PCy_3_), 0.92 (s, 3H, Si–C*H*_3_), 0.66 (s, 3H, Si–C*H*_3_), −21.77 (dd, ^2^ *J*_HP_ = 23.7 Hz, ^1^ *J*_HRh_ = 33.6 Hz, 1H, *H*–Rh). ^13^C­{^1^H} NMR (75 MHz, 298 K, CD_2_Cl_2_): δ 163.5 (d, ^2^ *J*_CRh_ = 2.4 Hz, *C* ^ipso–2^), 151.1 (s, *C* ^ipso–8a^), 146.3 (s, *C* ^ipso–4^), 133.0 (s, *C* ^ipso–8^), 132.5 (s, *C* ^7^), 126.3 (s, *C* ^ipso–4a^), 124.3 (s, *C* ^6^), 123.7 (s, *C* ^5^), 114.1 (s, *C* ^3^), 37.3 (d, ^1^ *J*_CP_ = 23.6 Hz, 3C, *C*H-PCy_3_), 32.2 (s, 3C, *C*H_2_–PCy_3_), 30.3 (br s, 3C, *C*H_2_–PCy_3_), 29.9 (br s, 3C, *C*H_2_–PCy_3_), 28.3 (d, ^3^ *J*_CP_ = 10.7 Hz, 3C, *C*H_2_–PCy_3_), 26.9 (br s, 3C, *C*H_2_–PCy_3_), 19.8 (s, 4-*C*H_3_), 16.8 (br s, 8-*C*H_3_), 10.7 (d, ^2^ *J*_CRh_ = 5.4 Hz, Si–*C*H_3_), 6.4 (s, Si–*C*H_3_). ^29^Si NMR (60 MHz, 298 K, CD_2_Cl_2_): δ 77.7 (dd, ^1^ *J*_SiRh_ = 40.7 Hz, ^2^ *J*_SiP_ = 13.1 Hz, *Si*(CH_3_)_2_). ^19^F NMR (282 MHz, 298 K, CD_2_Cl_2_): δ – 79.2 (s, OTf). ^31^P­{^1^H} NMR (121 MHz, 298 K, CD_2_Cl_2_): δ 49.2 (d, ^1^ *J*_RhP_ = 139.0 Hz, *P*Cy_3_). Anal. Calcd for C_32_H_50_F_3_NO_4_PRhSSi: C, 50.32; H, 6.60; N, 1.83; S, 4.20, found C, 50.14; H, 7.01; N, 1.78; S, 4.63. HR-MS (ESI^+^, *m*/*z*): calcd for C_31_H_50_NOPRhSi, [M–OTf]^+^ = 614.2454; found, 614.2471.

### Catalytic Reactions at NMR Scale

Under an argon atmosphere, an NMR tube was charged with **3** (2.3 mg, 0.003 mmol, 1.0 mol %) and hexamethylbenzene (4.0 mg, 0.025 mmol) as internal standard (IS), and the mixture was dissolved in 0.4 mL of benzene-*d*_6_. Then, pyrrolidine (25 μL, 0.3 mmol) and 0.3 mmol of the corresponding hydrosilane (HSiMe_2_Ph, 46 μL; HSiMePh_2_, 58 μL; HSiEt_3_, 47 μL; and HSiMe­(SiOMe_3_)_2_, 82 μL) were added at r.t. and the resulting mixture was heated at 333 K and monitored by ^1^H NMR spectroscopy.

### Catalytic Reactions in a *Man on the Moon* Microreactor

Catalytic reactions were carried out in a microreactor (*Man on the moon* series X102 Kit)^45^ with a total volume of 16.2 mL. Under an argon atmosphere, the reactor was filled with the corresponding amine (pyrrolidine, 82 μL, 1.0 mmol; piperidine, 45 μL; morpholine, 43 μL; diisobutylamine, 88 μL; *N*-methylaniline, 54 μL; *N*-isopropylaniline, 72 μL; 0.5 mmol) and **3** (7.6 mg, 0.01 mmol or 3.8 mg, 0.005 mmol). Then the reactor was closed and placed in an external oil bath preheated at the desired temperature. Once the temperature and pressure of the system were stabilized, the corresponding hydrosilane (HSiMe­(SiOMe_3_)_2_, 272 μL, 1.0 mmol; HSiMe_2_Ph, 76 μL; HSiMePh_2_, 99 μL; HSiMe­(SiOMe_3_)_2_, 136 μL; (HSiMe_2_(SiOMe_3_), 98 μL; HSi­(SiOMe_3_)_3_, 174 μL; 0.5 mmol) was injected with a microsyringe, and the pressure variation was recorded until a constant value was reached.

### NMR and HR-MS Data for the Siloxazanes 8a-8d and 9a

#### 1-(1,1,1,3,5,5,5-Heptamethyltrisiloxan-3-yl)­pyrrolidine (8a)

^1^H NMR (300 MHz, 298 K, C_6_D_6_): δ 2.98 (m, 4H, C*H*_2_-2 and C*H*_2_-5), 1.57 (m, 4H, C*H*_2_-3 and C*H*_2_-4), 0.17 (s, 3H, Si–C*H*_3_), 0.14 (s, 18H, 2x Si-(C*H*_3_)_3_). ^13^C­{^1^H} NMR (75 MHz, 298 K, C_6_D_6_): δ 46.3 (s, 2C, *C* ^2^ and *C* ^5^), 27.0 (s, 2C, *C* ^3^ and *C* ^4^), 1.8 (s, Si–*C*H_3_), 1.7 (s, 6C, 2x Si-(*C*H_3_)_3_). ^29^Si from the ^1^H–^29^Si HMQC NMR (60 MHz, 298 K, C_6_D_6_): δ 6.0 (s, 2x *Si*-(CH_3_)_3_), −47.8 (s, *Si*–CH_3_). HRMS (ESI^+^, *m*/*z*): calcd for C_11_H_30_NO_2_Si_3_, [M + H]^+^ = 292.1584; found, 292.1567. **8a** was isolated as a yellow oil in a 92% yield (134.2 mg, 0.46 mmol).

#### 1-(1,1,1,3,5,5,5-Heptamethyltrisiloxan-3-yl)­piperidine (8b)

^1^H NMR (300 MHz, 298 K, C_6_D_6_): δ 2.92 (m, 4H, C*H*_2_-2 and C*H*_2_-6), 1.51 (m, 2H, C*H*_2_-4), 1.40 (m, 4H, C*H*_2_-3 and C*H*_2_-5), 0.22 (s, 18H, 2x Si-(C*H*_3_)_3_), 0.19 (s, 3H, Si–C*H*_3_). ^13^C­{^1^H} NMR (75 MHz, 298 K, C_6_D_6_): δ 45.6 (s, 2C, *C* ^2^ and *C* ^6^), 28.0 (s, 2C, *C* ^3^ and *C* ^5^), 26.0 (s, *C* ^4^), 1.8 (s, Si–*C*H_3_), 1.7 (s, 6C, 2x Si-(*C*H_3_)_3_). ^29^Si from the ^1^H–^29^Si HMQC NMR (60 MHz, 298 K, C_6_D_6_): δ 6.0 (s, 2x *Si*-(CH_3_)_3_), −49.2­(s, *Si*–CH_3_). HRMS (ESI^+^, *m*/*z*): calcd for C_12_H_34_NO_2_Si_3_, [M + H]^+^ = 306.1741; found, 306.1736. **8b** was isolated as an orange oil in an 80% yield (123.0 mg, 0.40 mmol).

#### 4-(1,1,1,3,5,5,5-Heptamethyltrisiloxan-3-yl)­morpholine (8c)

^1^H NMR (300 MHz, 298 K, C_6_D_6_): δ 3.50 (m, 4H, C*H*_2_-3 and C*H*_2_-5), 2.85 (m, 4H, C*H*_2_-2 and C*H*_2_-6), 0.21 (s, 18H, 2x Si-(C*H*_3_)_3_), 0.14 (s, 3H, Si–C*H*_3_). ^13^C­{^1^H} NMR (75 MHz, 298 K, C_6_D_6_): δ 68.5 (s, 2C, *C* ^3^ and *C* ^5^), 45.0 (s, 2C, *C* ^2^ and *C* ^6^), 1.9 (s, Si–*C*H_3_), 1.8 (s, 6C, 2x Si-(*C*H_3_)_3_). ^29^Si from the ^1^H–^29^Si HMQC NMR (60 MHz, 298 K, C_6_D_6_): δ 7.1 (s, 2x *Si*-(CH_3_)_3_), −49.1 (s, *Si*–CH_3_). HRMS (ESI^+^, *m*/*z*): calcd for C_11_H_30_NO_3_Si_3_, [M + H]^+^ = 308.1533; found, 308.1516. **8c** was isolated as a yellow oil in an 80% yield (123.9 mg, 0.40 mmol).

#### *N*-(1,1,1,3,5,5-Heptamethyltrisiloxan)-*N*-methylaniline (8d)

^1^H NMR (300 MHz, 298 K, C_6_D_6_): δ 7.61 (m, 2H, *m*-(C_6_ *H*_5_)), 7.18 (m, 2H, *o*-(C_6_ *H*_5_)), 6.85 (m, 1H, *p*-(C_6_ *H*_5_)), 2.80 (s, 3H, N–C*H*_3_), 0.67 (s, 3H, Si–C*H*_3_), 0.66 (s, 18H, 2x Si-(C*H*_3_)_3_). ^13^C­{^1^H} NMR (75 MHz, 298 K, C_6_D_6_): δ 149.9 (s, *C* ^ipso^), 129.0 (s, 2C, *m*-*C*H–Ar), 119.5 (s, *p*-*C*H–Ar), 118.2 (s, 2C, *o*-*C*H–Ar), 34.0 (s, N–*C*H_3_), 1.9 (s, Si–*C*H_3_), 1.8 (s, 6C, 2x Si-(*C*H_3_)_3_). ^29^Si from the ^1^H–^29^Si HMQC NMR (60 MHz, 298 K, C_6_D_6_): δ 8.0 (s, 2x *Si*-(CH_3_)_3_), −48.8 (s, N–*Si*–CH_3_). HRMS (ESI^+^, *m*/*z*): calcd for C_12_H_23_NO_2_Si_3_, [M-2CH_3_]^+^ = 297.1037; found, 297.2330. **8d** was isolated as a yellow oil in a 76% yield (124.5 mg, 0.38 mmol).

#### 1-(1,1,3,3,3-Pentamethyldisiloxan-3-yl)­pyrrolidine (9a)

^1^H NMR (300 MHz, 298 K, C_6_D_6_): δ 2.98 (m, 4H, C*H*_2_-2 and C*H*_2_-5), 1.58 (m, 4H, C*H*_2_-3 and C*H*_2_-4), 0.18 (s, 6H, Si-(C*H*_3_)_2_), 0.15 (s, 9H, Si-(C*H*_3_)_3_). ^13^C­{^1^H} NMR (75 MHz, 298 K, C_6_D_6_): δ 46.2 (s, 2C, *C* ^2^ and *C* ^5^), 27.2 (s, 2C, *C* ^3^ and *C* ^4^), 2.1 (s, 2C, Si-(*C*H_3_)_2_), 0.5 (s, 3C, Si-(*C*H_3_)_3_). ^29^Si from the ^1^H–^29^Si HMQC NMR (60 MHz, 298 K, C_6_D_6_): δ 5.0 (s, *Si*-(CH_3_)_3_), −13.1 (s, *Si*–CH_3_). HRMS (ESI^+^, *m*/*z*): calcd for C_8_H_20_DNNaOSi_2_, [M-CH_3_+D + Na]^+^ = 227.1122; found, 226.9515. **9a** was isolated as a yellow oil in a 78% yield (85.7 mg, 0.39 mmol).

### Single-Crystal Structure Determination

X-ray diffraction data of compound **3** were collected on a D8 VENTURE Bruker diffractometer, using Mo κα (λ = 0.71073 Å). Single crystal was mounted on a MiTeGen support and cooled to 100(2) K with open-flow nitrogen gas. Data were collected using ω and φ scans with narrow frames strategies. Diffracted intensities were integrated and corrected from absorption effects with APEX4 package.^47^ Crystal structure was solved and refined using SHELXS^48^ and SHELXL^49^ included in Olex2 program^50^ Most of the hydrogen atoms were included in the model in calculated positions and refined with a riding model. Hydrogen atoms of C(11) methyl group (involved in agostic interactions) and hydride ligand were included in the model in observed positions and freely refined.

CCDC 2513622 contains the supplementary crystallographic data for this paper. These data can be obtained free of charge from the Cambridge Crystallographic Data Center via www.ccdc.cam.ac.uk/data_request/cif.

Crystal data of **3**: C_32_H_50_F_3_NO_4_PRhSSi; *M*_r_ = 763.76; colorless prism 0.050 × 0.060 × 0.120 mm^3^; Monoclinic *P*2_1_/*c*; *a* = 9.3579(7) Å, *b* = 21.0525(15) Å; *c* = 17.4963(12) Å, β = 90.553(3)°; *V* = 3446.7(4) Å^3^; *Z* = 4; Dc = 1.472 g/cm^3^; μ = 0.690 mm^–1^; min and max. absorption correction factors: 0.7182 and 0.7428; 2θ_max_ = 56.668°; 275202 reflections measured, 8603 unique; *R*_int_ = 0.0653; number of data/restraint/parameters: 8603/0/416; *R*_1_ = 0.0263 [7752 reflections, I > 2σ­(I)], w*R*(F^2^) = 0.0669 (all data); largest difference peak: 0.745 e Å^–3^.

### Computational Details

Optimization of geometries was performed using Gaussian 16 (revision C.01) using an ultrafine integration grid (int = ultrafine).^51^ Geometry optimizations and frequency calculations were performed using the B3LYP^38^ functional with def2-TZVP^40^ (Rh) and 6–31G**^52^ (all other atoms) basis sets (unless specified) with solvent corrections (PCM, benzene, ε = 2.2706)^53^ and an empirical dispersion correction (Grimme, GD3)^39^ in all calculations. Frequency analyses for all geometries were performed using the enhanced criteria to confirm the nature of the structures either minima (no imaginary frequency) or transition states (only one imaginary frequency). Natural Bond Orbital analysis was carried out using NBO 7.0 ^42^ using same level of theory. QTAIM analyses was performed using Multiwfn program^54^ and graphics of the results generated with VMD 1.9.1.^55^

### Safety Statement

No uncommon hazards are noted.

## Supplementary Material





## Notes

coordinatesfinal.xyz (XYZ)
Experimental details, NMR spectra and theoretical calculations data (PDF) (PDF)

## Abbreviation

TOF: turnover frequency
