# Supplementary material for: Triflate-Enabled Rhodium-Catalyzed Cross-Dehydrogenative Si–N Bond Formation from Hydrosiloxanes
Source: Inorg Chem. 2026 Jul 7;65(28):16283–95. doi: 10.1021/acs.inorgchem.6c01798 (PMC13390040; doi:10.1021/acs.inorgchem.6c01798)
Supplement: Supplementary file 2 [file ic6c01798_si_002.pdf]

Supporting Information

Triflate-Enabled Rhodium-Catalyzed Cross-Dehydrogenative Si–N Bond Formation from Hydrosiloxanes

Marina Padilla, María Batuecas*, Pilar García-Orduña, Luis A. Oro and Francisco J. Fernández-Álvarez*

Departamento de Química Inorgánica, Facultad de Ciencias – Instituto de Síntesis Química y Catálisis Homogénea (ISQCH), Universidad de Zaragoza–CSIC, Campus Plaza San Francisco, 50009, Zaragoza, Spain.

Corresponding Author:

F.J.F.-A.: paco@unizar.es

M. B.: mbatuecas@unizar.es

Table of contents

1. General information S3

2. Synthesis of 2 and 3 .... S4

3. Reaction conditions optimization S6

    3.1. Screening of catalyst precursors S6

    3.2. Screening of hydrosilanes S7

    3.3. Study of reaction temperature influence S8

4. $HSiR_3$ scope S12

5. Amine scope S15

6. Characterization of siloxazanes S17

7. Miscellaneous reactions S20

8. DFT calculations S21

    8.1. Computational methods S21

    8.2. Agostic interaction S21

    8.3. Mechanistic studies S23

9. References S33

10. NMR spectra S35

    10.1. NMR spectra of Rh–complexes S35

    10.2. ^{13}C NMR spectra of **2** and **3** S43

    10.3. NMR spectra of reaction conditions optimization S44

    10.4. NMR spectra of siloxazanes S51

    10.5. NMR spectra of miscellaneous reactions S61

11. Cartesian coordinates S65

1. General information

All manipulations were performed with rigorous exclusion of air at an argon/vacuo manifold using standard Schlenk-tube or glovebox techniques. Solvents were dried by the usual procedures and distilled under argon prior to use or obtained oxygen- and water-free from a Solvent Purification System (Innovative Technologies). ^{1}H, $^{13}C\{^{1}H\}$, ^{13}C, ^{1}H-^{13}C HSQC, ^{1}H-^{13}C HMBC, $^{31}P\{^{1}H\}$, ^{1}H-^{29}Si HMQC, $^{29}Si\{^{1}H\}$ and ^{19}F NMR spectra were recorded on a Bruker Avance 300 MHz and Bruker Avance 400 MHz instrument. Coupling constants *J* are given in hertz (Hz) (multiplicity: s = singlet, d = doublet, dd = double doublet, ddd= doublet of doublets of doublets, m = multiplet, bs = broad signal). The "Brief Guide to the Nomenclature of Organic Chemistry" was followed for signal assignment.[S1] $[\{Rh(coe)_2\}_2(\mu\text{-}Cl)_2]$[S2] was prepared following the reported methodology. The secondary amines and hydrosilanes were purchased from commercial sources and dried on 4Å molecular sieves prior to use.

Turnover Number (TON) and Turnover Frequency (TOF) determination.[S3]

H_2 pressure is the pressure measured by the microreactor. $P_{H2} = P_{measured}$

Amount of H_2 formed was calculated with the Ideal Gas Law. $n_{H2} = \frac{P_{H2} \cdot V}{R \cdot T}$

Total volume (V) = 0.0162 L; R constant = 0.082 atm·L·mol^{-1}·K^{-1}

$$TON = \frac{n_{H2}}{n_{cat}};\ TOF = \frac{TON}{t}$$

2. Synthesis of 2 and 3

Scheme S1. Synthesis of [Rh(H)(Cl)(κ^2-NSi^{DMQ})(PCy$_3$)] (**2**).

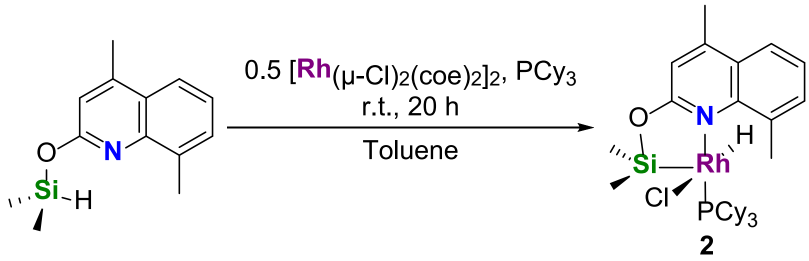

To a solution of [{Rh(coe)$_2$}$_2$(μ-Cl)$_2$] (311 mg, 0.43 mmol) in toluene (12 mL) was added a solution of (4,8-dimethylquinolin-2-yloxy)dimethylsilane (200 mg, 0.86 mmol) in toluene (5 mL), followed by a solution of PCy$_3$ (255 mg, 0.91 mmol) in toluene (5 mL) (Scheme S1). After 20 hours of stirring at r.t., the reaction mixture was evaporated to dryness, and the residue was extracted with a 1:5 mixture of dichloromethane and hexane (5 mL). The resulting solution was cooled to 253 K and maintained at this temperature for 3 h, during which a white solid precipitated. The suspension was then filtered through Celite at low temperature, and the solid was collected to afford compound **2** as a white solid in 70% yield (391 mg, 0.60 mmol). ^{1}H NMR (300 MHz, 298 K, CD$_2$Cl$_2$): δ 7.77 (m, 1H, H^5), 7.49 (d, $^3J_{HH}$ = 7.1 Hz, 1H, H^7), 7.32 (dd, $^3J_{HH}$ = 8.2 Hz, $^3J_{HH}$ = 7.1 Hz, 1H, H^6), 6.92 (bs, 1H, H^3), 3.23 (s, 3H, 8-CH_3), 2.63 (d, 3H, $^4J_{HH}$ = 1.0 Hz, 4-CH_3), 2.23 – 1.29 (overlapping signals, 33H, CH-PCy$_3$ and CH_2-PCy$_3$), 0.84 (s, 3H, Si-CH_3), 0.63 (s, 3H, Si-CH_3), –17.09 (dd, $^2J_{HP}$ = 22.3 Hz, $^1J_{HRh}$ = 25.8 Hz, 1H, H–Rh). ^{13}C{^{1}H} NMR (75 MHz, 298 K, CD$_2$Cl$_2$): δ 162.9 (d, $^2J_{CRh}$ = 2.6 Hz, C^{ipso-2}), 149.9 (s, $C^{ipso-8a}$), 145.8 (s, C^{ipso-4}), 133.5 (s, C^{ipso-8}), 132.8 (s, C^7), 126.5 (s, $C^{ipso-4a}$), 124.1 (s, C^6), 123.4 (s, C^5), 114.3 (s, C^3), 37.1 (d, $^1J_{CP}$ = 23.5 Hz, 3C, CH-PCy$_3$), 31.2 (bs, 3C, CH$_2$-PCy$_3$), 30.3 (bs, 3C, CH$_2$-PCy$_3$), 30.2 (bs, 3C, CH$_2$-PCy$_3$), 28.3 (d, $^3J_{CP}$ = 10.3 Hz, 3C, CH$_2$-PCy$_3$), 27.1 (bs, 3C, CH$_2$-PCy$_3$), 19.7 (s, 4-CH$_3$), 18.0 (d, J_{CRh} = 2.0 Hz, 8-CH$_3$), 11.7 (d, $^2J_{CRh}$ = 5.5 Hz, Si-CH$_3$), 8.2 (d, $^2J_{CRh}$ = 1.1 Hz, Si-CH$_3$). ^{29}Si NMR (60 MHz, 298 K, CD$_2$Cl$_2$): δ 76.9 (dd, $^1J_{SiRh}$ = 40.4 Hz, $^2J_{SiP}$ = 14.4 Hz, Si(CH$_3$)$_2$). ^{31}P{^{1}H} NMR (121 MHz, 298 K, CD$_2$Cl$_2$): δ 51.8 (d, $^1J_{RhP}$ = 142.1 Hz, PCy$_3$). Anal. calcd. for $C_{31}H_{50}ClNOPRhSi\cdot0.2CH_2Cl_2$: C, 56.17; H, 7.61; N, 2.10, found C, 56.54; H, 7.89; N, 2.27. HR-MS (ESI$^+$, m/z): calcd. for $C_{31}H_{50}NOPRhSi$, [M–Cl]$^+$ = 614.2454; found = 614.2437

Scheme S2. Synthesis of [Rh(H)(OTf)(κ^2-NSi^{DMQ})(PCy$_3$)] (**3**).

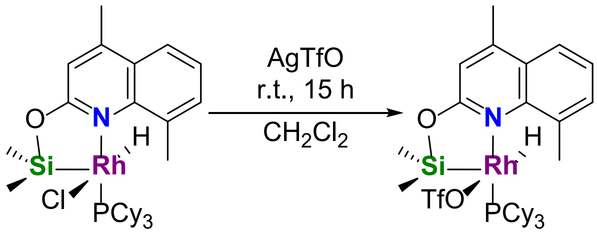

2 **3**

CH_2Cl_2 (10 mL) was added to a mixture of **2** (300 mg, 0.46 mmol) and AgTfO (119 mg, 0.46 mmol) in the dark (Scheme S2). After stirring for 15 hours at r.t., the resulting suspension was filtered through Celite using a cannula. The solution was evaporated to dryness to afford compound **3** as a light brown solid in 73% yield (256 mg, 0.32 mmol). ^{1}H NMR (300 MHz, 298 K, CD_2Cl_2): δ 7.83 (d, $^3J_{HH}$ = 8.2 Hz, 1H, H^5), 7.56 (d, $^3J_{HH}$ = 7.2 Hz, 1H, H^7), 7.37 (dd, $^3J_{HH}$ = 8.2 Hz, $^3J_{HH}$ = 7.2 Hz, 1H, H^6), 6.97 (bs, 1H, H^3), 3.26 (s, 3H, 8-CH_3), 2.67 (bs, 3H, 4-CH_3), 2.02 – 1.27 (overlapping signals, 33H, C*H*-PCy$_3$ and CH_2-PCy$_3$), 0.92 (s, 3H, Si-CH_3), 0.66 (s, 3H, Si-CH_3), –21.77 (dd, $^2J_{HP}$ = 23.7 Hz, $^1J_{HRh}$ = 33.6 Hz, 1H, *H*–Rh). ^{13}C{^{1}H} NMR (75 MHz, 298 K, CD_2Cl_2): δ 163.5 (d, $^2J_{CRh}$ = 2.4 Hz, C^{ipso-2}), 151.1 (s, $C^{ipso-8a}$), 146.3 (s, C^{ipso-4}), 133.0 (s, C^{ipso-8}), 132.5 (s, C^7), 126.3 (s, $C^{ipso-4a}$), 124.3 (s, C^6), 123.7 (s, C^5), 114.1 (s, C^3), 37.3 (d, $^1J_{CP}$ = 23.6 Hz, 3C, *C*H-PCy$_3$), 32.2 (s, 3C, *C*H_2-PCy$_3$), 30.3 (bs, 3C, *C*H_2-PCy$_3$), 29.9 (bs, 3C, *C*H_2-PCy$_3$), 28.3 (d, $^3J_{CP}$ = 10.7 Hz, 3C, *C*H_2-PCy$_3$), 26.9 (bs, 3C, *C*H_2-PCy$_3$), 19.8 (s, 4-*C*H_3), 16.8 (bs, 8-*C*H_3), 10.7 (d, $^2J_{CRh}$ = 5.4 Hz, Si-*C*H_3), 6.4 (s, Si-*C*H_3). ^{29}Si NMR (60 MHz, 298 K, CD_2Cl_2): δ 77.7 (dd, $^1J_{SiRh}$ = 40.7 Hz, $^2J_{SiP}$ = 13.1 Hz, *Si*(CH_3)$_2$). ^{19}F NMR (282 MHz, 298 K, CD_2Cl_2): δ –79.2 (s, OTf). ^{31}P{^{1}H} NMR (121 MHz, 298 K, CD_2Cl_2): δ 49.2 (d, $^1J_{RhP}$ = 139.0 Hz, *P*Cy$_3$). Anal. calcd. for $C_{32}H_{50}F_3NO_4PRhSSi$: C, 50.32; H, 6.60; N, 1.83; S, 4.20, found C, 50.14; H, 7.01; N, 1.78; S, 4.63. HR-MS (ESI$^+$, *m*/*z*): calcd. for $C_{31}H_{50}NOPRhSi$, [M–OTf]$^+$ = 614.2454; found = 614.2471.

3. Reaction conditions optimization

3.1. Screening of catalyst precursors

Under an argon atmosphere, an NMR tube was charged with 1 mol% (0.003 mmol) of the corresponding complex (**2**, 2.0 mg; **3**, 2.3 mg; **4**, 2.5 mg) and hexamethylbenzene (8.0 mg, 0.05 mmol) as internal standard (IS) and dissolved in 0.4 mL of benzene-d_6. Then, pyrrolidine (25 µL, 0.3 mmol) and $HSiMe_2Ph$ (46 µL, 0.3 mmol) or $HSiMe(SiOMe_3)_2$ (82 µL, 0.3 mmol) were added at room temperature (r.t.) and the resulting mixture was frozen by submerging the NMR tube in liquid nitrogen (Scheme S3). The reaction was allowed to warm to r.t. and monitored by ^1H NMR spectroscopy (Figures S33-S38). The results are shown in Table S1.

Scheme S3. Catalytic reaction of pyrrolidine with $HSiMe_2Ph$ or $HSiMe(SiOMe_3)_2$, using different catalyst precursors (1 mol%) in C_6D_6 at r.t.

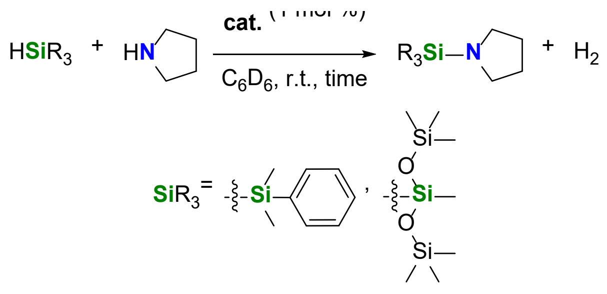

5a **8a**

Table S1. Results of the catalytic reaction of pyrrolidine with $HSiMe_2Ph$ or $HSiMe(SiOMe_3)_2$ using different catalyst precursors (1 mol%) in C_6D_6 at r.t.. Yields were calculated using hexamethylbenzene as IS.

Hydrosilane	Cat.	Time (h)	Yield (%)
$HSiMe_2Ph$ (**5a**)	**2**	0.5	<1
		3	<1
	**3**	0.5	1
		3	10
	**4**	0.5	75
		3	92
$HSiMe(SiOMe_3)_2$ (**8a**)	**2**	0.5	<1
		3	<1
	**3**	0.5	47
		3	>99
	**4**	0.5	<1
		3	10

3.2. Screening of hydrosilanes

Under an argon atmosphere, an NMR tube was charged with 1 mol% of **3** (2.3 mg, 0.003 mmol) and hexamethylbenzene (4.0 mg, 0.025 mmol) as internal standard (IS) and dissolved in 0.4 mL of benzene-d_6. Then, pyrrolidine (24 µL, 0.3 mmol) and 0.3 mmol of the corresponding hydrosilane ($HSiMe_2Ph$, 45 µL; $HSiMePh_2$, 58 µL; $HSiEt_3$, 47 µL; and $HSiMe(SiOMe_3)_2$, 82 µL) were added at r.t. and the resulting mixture was heated at 333 K and monitored by ^1H NMR spectroscopy at 0.5 and 3 hours (Scheme S4) (Figures S39-S42). The results are shown in Table S2.

Scheme S4. **3**-catalyzed (1 mol%) reaction of pyrrolidine with different hydrosilanes and $HSiMe(SiOMe_3)_2$ in C_6D_6 at 333 K.

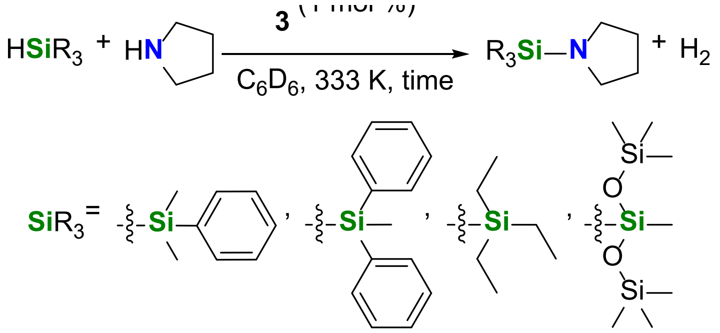

5a **6a** **7a** **8a**

Table S2. Results of the **3**-catalyzed (1 mol%) reaction of pyrrolidine with different hydrosilanes and $HSiMe(SiOMe_3)_2$ in C_6D_6 at 333 K. Yields were calculated using hexamethylbenzene as IS.

Hydrosilane	Time (h)	Yield (%)
**$HSiMe_2Ph$ (5a)**	0.5	79
	3	90
**$HSiMePh_2$ (6a)**	0.5	24
	3	54
**$HSiEt_3$ (7a)**	0.5	<1
	3	<1
**$HSiMe(SiOMe_3)_2$ (8a)**	0.5	>99
	3	>99

3.3. Study of reaction temperature influence

Catalytic reactions were carried out on a microreactor (man on the moon ™ series X102 Kit)[S4] with a total volume of 16.2 mL. Under an argon atmosphere, the reactor was filled with pyrrolidine (82 µL, 1 mmol) and **3** (7.6 mg, 0.01 mmol). The reactor was then closed and put in an external oil bath preheated at the corresponding temperature. Once the temperature and pressure of the system were stabilized, $HSiMe(SiOMe_3)_2$ (272 µL, 1 mmol) was injected with a microsyringe (Scheme S5) and the pressure variation was measured until a constant value was reached. The results are shown in Figure S1, Figure S2 and Table S3.

Scheme S5. **3**-catalyzed (1 mol%) reaction of pyrrolidine with $HSiMe(SiOMe_3)_2$ at different temperatures under neat conditions.

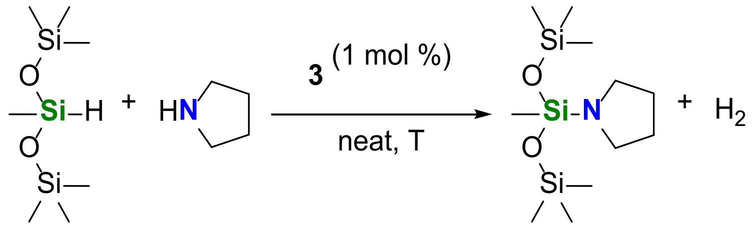

6a

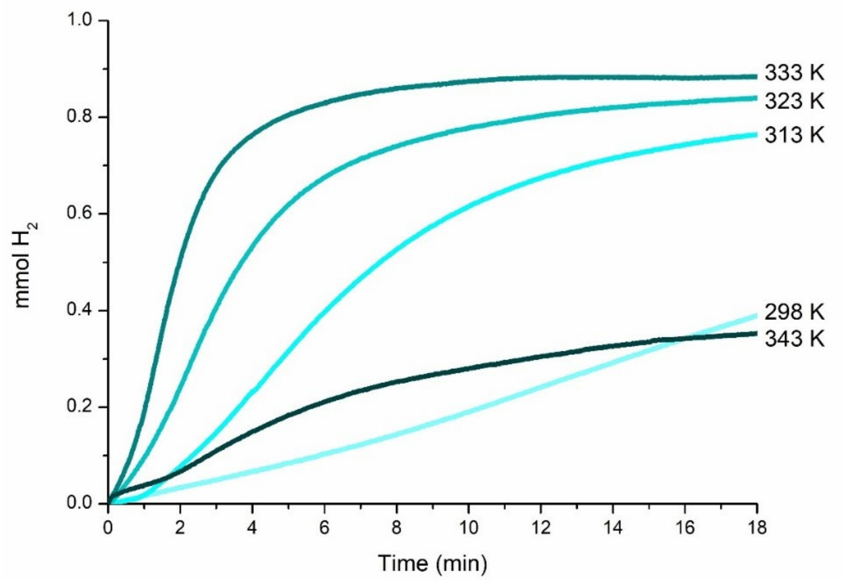

Figure S1. Time profile of H_2 (mmol) generation from the **3**-catalyzed (1 mol%) reaction of pyrrolidine (1 mmol) with $HSiMe(SiOMe_3)_2$ (1 mmol) at different temperatures under neat conditions.

Table S3. TOF values of the **3**-catalyzed (1 mol%) reaction of pyrrolidine with $HSiMe(SiOMe_3)_2$ at different temperatures under neat conditions.

Temperature (K)	TOF_{2min} (h^{-1})
**298**	100
**313**	230
**323**	730
**333**	1520
**343**	200

Calculations for activation energy from Arrhenius equation

$$k = A \cdot e^{\frac{-Ea}{RT}} \rightarrow \ln k = \ln A - \frac{Ea}{RT}$$

$$\ln k \approx Ln\ (TOF_{2min})$$

$$Ln\ (TOF_{2min}) = \left(-\frac{Ea}{R}\right) \cdot \frac{1}{T} + \ln A$$

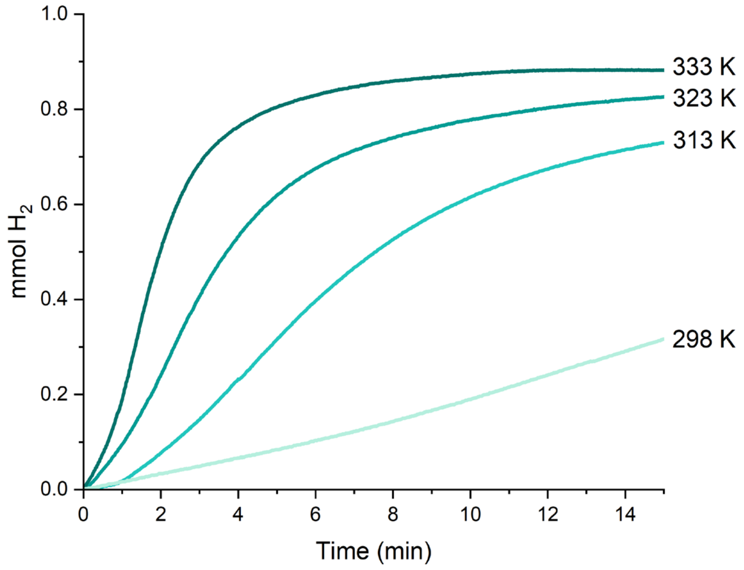

Figure S2. Time profile of H_2 (mmol) generation from the **3**-catalyzed (1 mol%) reaction of pyrrolidine (1 mmol) with $HSiMe(SiOMe_3)_2$ (1 mmol) at different temperatures under neat conditions.

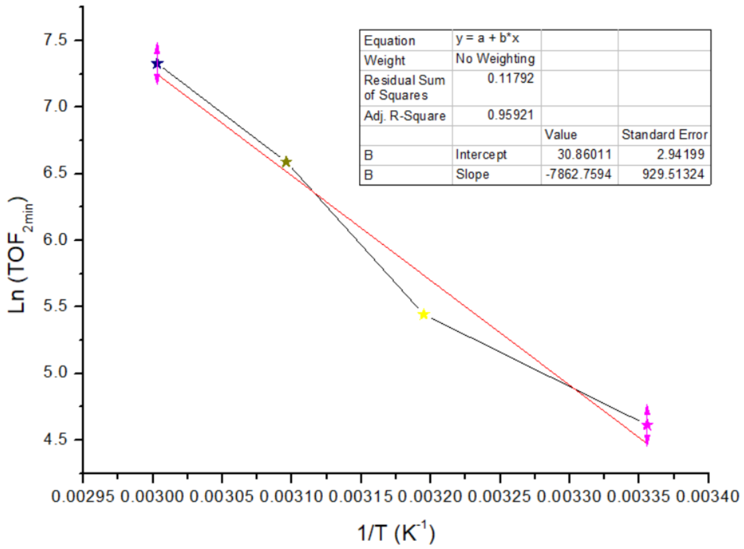

Figure S3. Representation of Ln (TOF_{2min}) vs 1/T for calculation of the activation energy.

$E_a = 15.6 \pm 1.8$ kcal·mol^{-1}

Calculations for activation energy from Eyring equation

$$k = \frac{k_B T}{h} \cdot e^{\frac{-\Delta G^{\neq}}{RT}} \rightarrow \ln\left(\frac{k}{T}\right) = \left(-\frac{\Delta H^{\neq}}{R}\right) \cdot \frac{1}{T} + \frac{\Delta S^{\neq}}{R} + \ln\left(\frac{k_B}{h}\right)$$

$$\ln\left(\frac{k}{T}\right) \approx Ln\ (TOF_{2min}/T)$$

$$Ln\left(\frac{TOF_{2min}}{T}\right) = \left(-\frac{\Delta H^{\neq}}{R}\right) \cdot \frac{1}{T} + \frac{\Delta S^{\neq}}{R} + \ln\left(\frac{k_B}{h}\right)$$

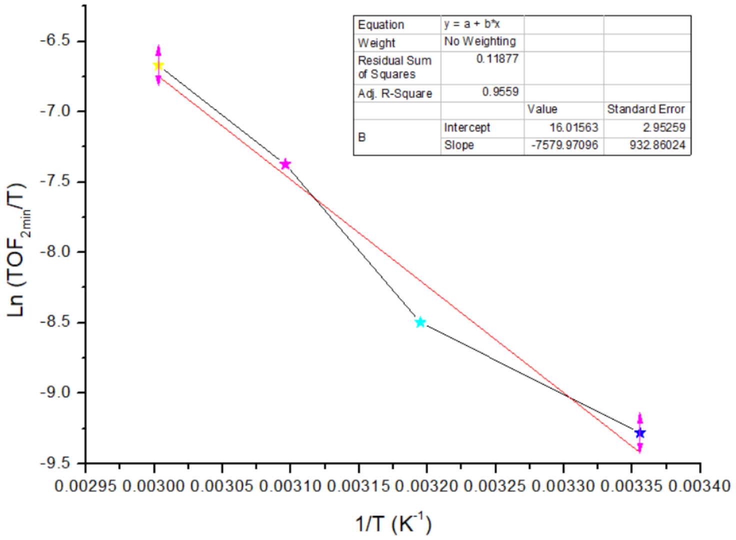

Figure S4. Representation of Ln (TOF_{2min}/T) vs 1/T for calculation of the activation energy.

$\Delta H^{\neq} = 15.1 \pm 1.9\ kcal \cdot mol^{-1}$

$\Delta S^{\neq} = -15.4 \pm 5.9\ cal \cdot mol^{-1} \cdot K^{-1}$

$\Delta G^{\neq} = 19.6 \pm 0.1\ kcal \cdot mol^{-1}$

4. $HSiR_3$ scope

Previous catalytic reactions under neat conditions on a microreactor

Catalytic reactions were carried out on a microreactor (man on the moon ™ series X102 Kit)[S4] with a total volume of 16.2 mL. Under an argon atmosphere, the reactor was filled with pyrrolidine (41 μL, 0.5 mmol) and **3** (3.8 mg, 0.005 mmol). The reactor was then closed and put in an external oil bath preheated at 333 K. Once the temperature and pressure of the system were stabilized, the corresponding amount of hydrosilane was injected with a microsyringe ($HSiMe_2Ph$, 76 μL; $HSiMePh_2$, 99 μL; $HSiMe(SiOMe_3)_2$, 136 μL; 0.5 mmol) (Scheme S6) and the pressure variation was measured until a constant value was reached. The results are shown in Figure S5 and Table S4.

Scheme S6. **3**-catalyzed (1 mol%) reaction of pyrrolidine with different hydrosilanes and $HSiMe(SiOMe_3)_2$ at 333 K under neat conditions.

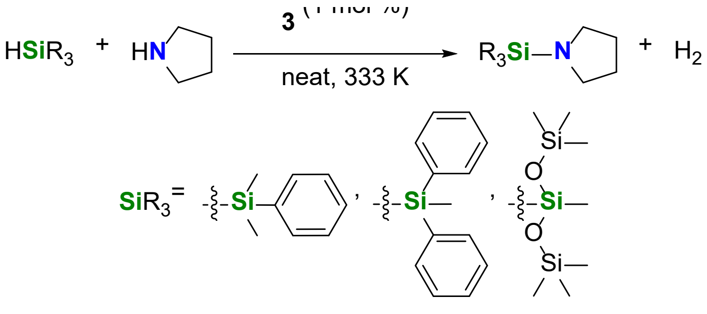

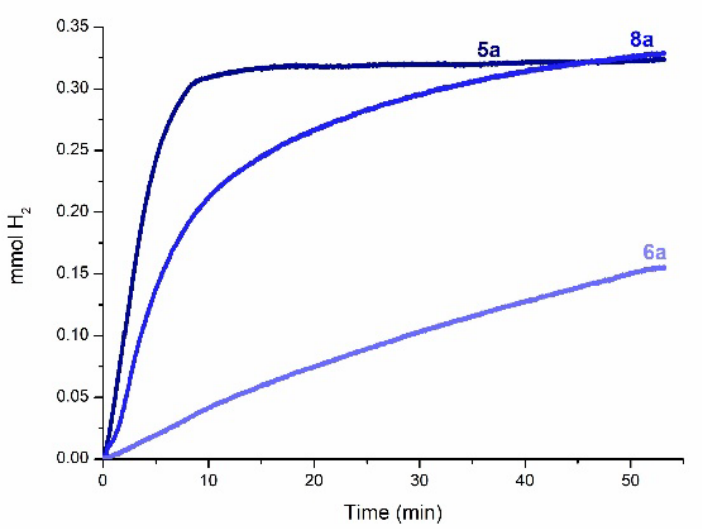

Figure S5. Time profile of H_2 (mmol) generation from the **3**-catalyzed (1 mol%) reaction of pyrrolidine (0.5 mmol) with different hydrosilanes and $HSiMe(SiOMe_3)_2$ (0.5 mmol) at 333 K under neat conditions.

Table S4. TOF values of the **3**-catalyzed (1 mol%) reaction of pyrrolidine with different hydrosilanes and $HSiMe(SiOMe_3)_2$ at 333 K under neat conditions.

Hydrosilane	TOF_{2min} (h^{-1})	TOF_{10min} (h^{-1})
**$HSiMe_2Ph$ (5a)**	610	370
**$HSiMePh_2$ (6a)**	40	50
**$HSiMe(SiOMe_3)_2$ (8a)**	260	260

Siloxane scope

Catalytic reactions were carried out on a microreactor (man on the moon ™ series X102 Kit)[S4] with a total volume of 16.2 mL. Under an argon atmosphere, the reactor was filled with pyrrolidine (41 µL, 0.5 mmol) and **3** (3.8 mg, 0.005 mmol). The reactor was then closed and put in an external oil bath preheated at 333 K. Once the temperature and pressure of the system were stabilized, the corresponding amount of siloxane was injected with a microsyringe ($HSiMe_2(SiOMe_3)$, PMDS, 98 µL; $HSiMe(SiOMe_3)_2$, HMTS, 136 µL; $HSi(SiOMe_3)_3$, TTMS, 174 µL; 0.5 mmol) (Scheme S7) and the pressure variation was measured until a constant value was reached. The results are shown in Figure S6 and Table S5.

Scheme S7. **3**-catalyzed (1 mol%) reaction of pyrrolidine with different hydrosiloxanes at 333 K under neat conditions.

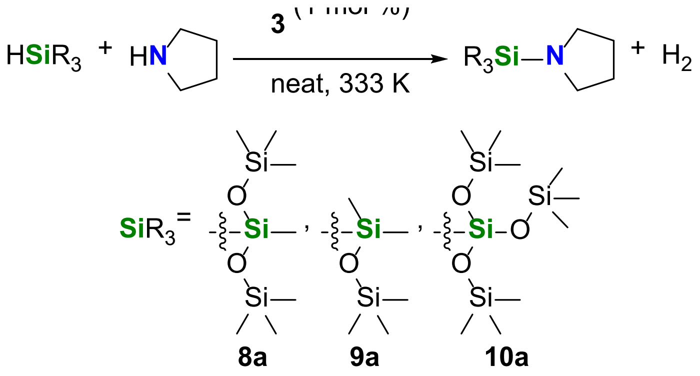

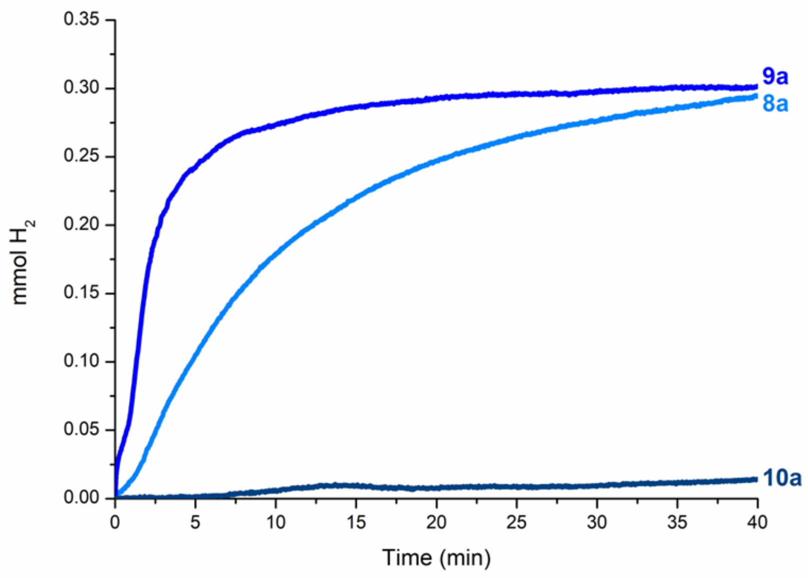

Figure S6. Time profile of H_2 (mmol) generation from the **3**-catalyzed (1 mol%) reaction of pyrrolidine (0.5 mmol) with different hydrosiloxanes (0.5 mmol) at 333 K under neat conditions.

Table S5. TOF values of the **3**-catalyzed (1 mol%) reaction of pyrrolidine with different hydrosiloxanes at 333 K under neat conditions.

Siloxane	TOF_{2min} (h^{-1})	TOF_{10min} (h^{-1})
**$HSiMe(SiOMe_3)_2$ (8a)**	260	260
**$HSiMe_2(SiOMe_3)$ (9a)**	980	330
**$HSi(SiOMe_3)_3$ (10a)**	4	7

5. Amine scope

Catalytic reactions were carried out on a microreactor (man on the moon ™ series X102 Kit)[S4] with a total volume of 16.2 mL. Under an argon atmosphere, the reactor was filled with 0.5 mmol of the corresponding amine (pyrrolidine, 41 µL; piperidine, 45 µL; morpholine, 43 µL; diisobutylamine, 88 µL; *N*-methylaniline, 54 µL; *N*-isopropylaniline, 72 µL) and **3** (3.8 mg, 0.005 mmol). The reactor was then closed and put in an external oil bath preheated at 333 K. Once the temperature and pressure of the system were stabilized, $HSiMe(SiOMe_3)_2$ (136 µL, 0.5 mmol) was injected with a microsyringe (Scheme S8) and the pressure variation was measured until a constant value was reached. The results are shown in Figure S7 and Table S6.

Scheme S8. **3**-catalyzed (1 mol%) reaction of different amines with $HSiMe(SiOMe_3)_2$ at 333 K under neat conditions.

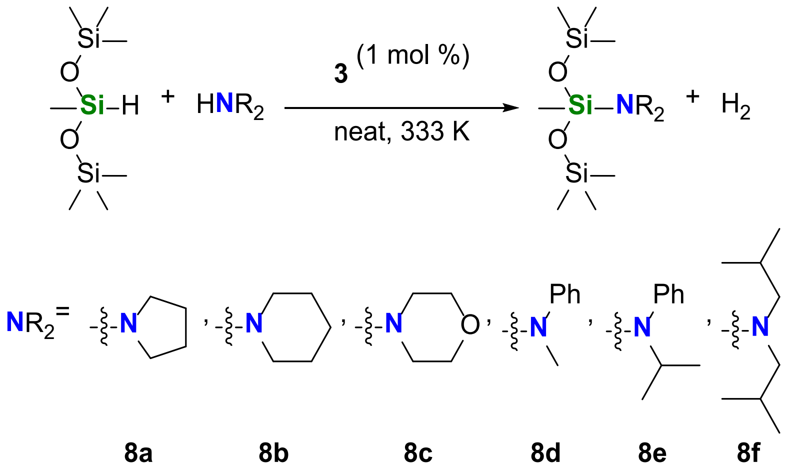

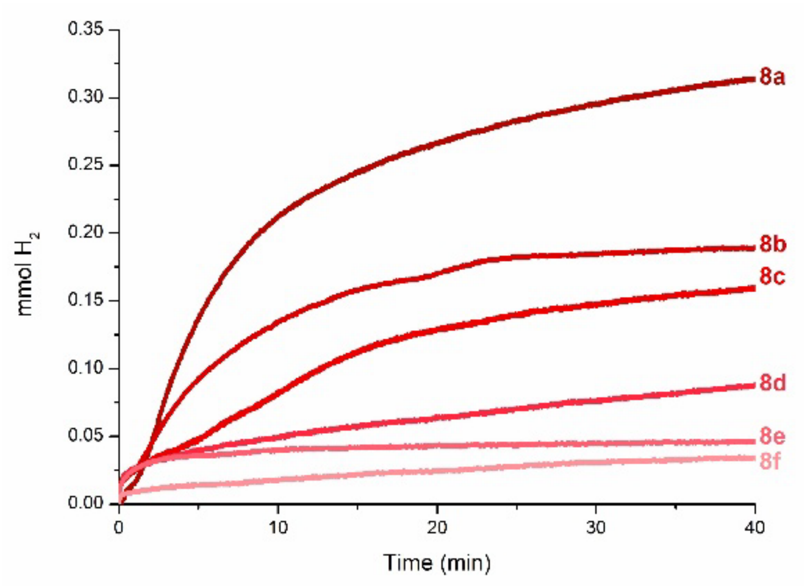

Figure S7. Time profile of H_2 (mmol) generation from the **3**-catalyzed (1 mol%) reaction of different amines (0.5 mmol) with $HSiMe(SiOMe_3)_2$ at 333 K under neat conditions.

Table S6. TOF values of the **3**-catalyzed (1 mol%) reaction of different amines with $HSiMe(SiOMe_3)_2$ at 333 K under neat conditions.

Amine	TOF_{2min} (h^{-1})	TOF_{10min} (h^{-1})
**Pyrrolidine (8a)**	260	260
**Piperidine (8b)**	260	160
**Morpholine (8c)**	200	100
***N*-methylaniline (8d)**	180	60
***N*-isopropylaniline (8e)**	180	50
**Diisobutylamine (8f)**	60	20

6. Characterization of siloxazanes

General procedure for the CDC reaction under neat conditions

Catalytic reactions were carried out on a microreactor (man on the moon ™ series X102 Kit)[S4] with a total volume of 16.2 mL. Under an argon atmosphere, the reactor was filled with the corresponding amount of each amine (0.5 mmol) and **3** (3.8 mg, 0.005 mmol). The reactor was then closed and put in an external oil bath preheated at 333 K, and when the temperature and pressure of the system were stabilized, the corresponding amount of $HSiMe(SiOMe_3)_2$ (136 µL,0.5 mmol), $HSiMe_2(SiOMe_3)$ (98 µL, 0.5 mmol) or $HSi(SiOMe_3)_3$ (174 µL, 0.5 mmol) was injected with a microsyringe (Scheme S9). Once the reaction has finished, the reactor was opened and connected to a Schlenk line. Then, under argon atmosphere, hexane (2 mL) was added and the solution was filtered through celite with a cannula to remove the residual catalyst. The solution is brought to dryness and the oily product was characterized by NMR spectroscopies and high-resolution mass spectrometry (HR-MS).

The "Brief Guide to the Nomenclature of Organic Chemistry" was followed for signal assignment,[S1] and when it was possible the siloxazanes were also characterized by comparison with reported data (**8a**[S14]).

Scheme S9. Synthesis of siloxazanes.

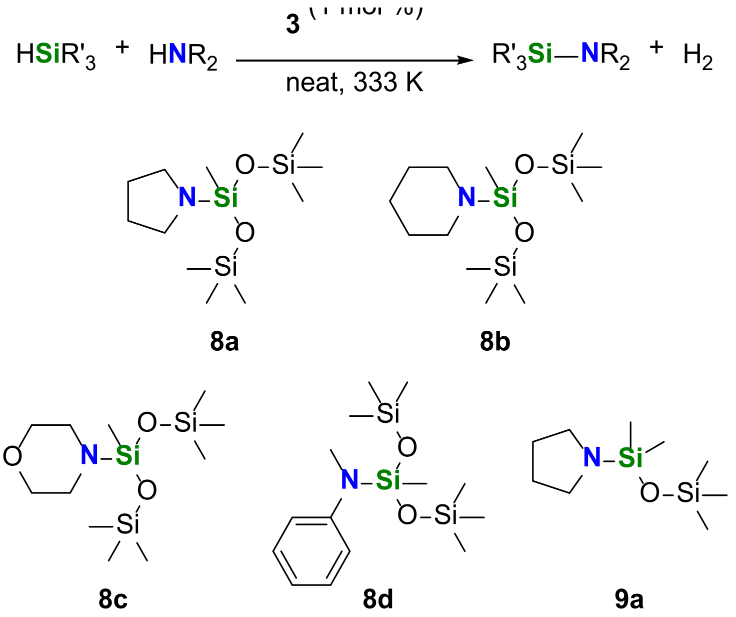

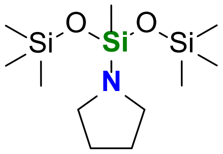

1-(1,1,1,3,5,5,5-heptamethyltrisiloxan-3-yl)pyrrolidine (8a). ^{1}H NMR (300 MHz, 298 K, C_6D_6): δ 2.98 (m, 4H, CH_2-2 and CH_2-5), 1.57 (m, 4H, CH_2-3 and CH_2-4), 0.17 (s, 3H, Si-CH_3), 0.14 (s, 18H, 2x Si-$(CH_3)_3$). ^{13}C{^{1}H} NMR (75 MHz, 298 K, C_6D_6): δ 46.3 (s, 2C, C^2 and C^5), 27.0 (s, 2C, C^3 and C^4), 1.8 (s, Si-CH_3), 1.7 (s, 6C, 2x Si-$(CH_3)_3$). ^{29}Si from the ^{1}H-^{29}Si HMQC NMR (60 MHz, 298 K, C_6D_6): δ 6.0 (s, 2x *Si*-$(CH_3)_3$), –47.8 (s, *Si*-CH_3). HRMS (ESI$^+$, *m/z*): calcd. for $C_{11}H_{30}NO_2Si_3$, $[M+H]^+$ = 292.1584; found = 292.1567. **8a** was isolated as a yellow oil in a 92% yield (134.2 mg, 0.46 mmol).

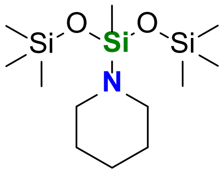

1-(1,1,1,3,5,5,5-heptamethyltrisiloxan-3-yl)piperidine (8b). ^{1}H NMR (300 MHz, 298 K, C_6D_6): δ 2.92 (m, 4H, CH_2-2 and CH_2-6), 1.51 (m, 2H, CH_2-4), 1.40 (m, 4H, CH_2-3 and CH_2-5), 0.22 (s, 18H, 2x Si-$(CH_3)_3$), 0.19 (s, 3H, Si-CH_3). ^{13}C{^{1}H} NMR (75 MHz, 298 K, C_6D_6): δ 45.6 (s, 2C, C^2 and C^6), 28.0 (s, 2C, C^3 and C^5), 26.0 (s, C^4), 1.8 (s, Si-CH_3), 1.7 (s, 6C, 2x Si-$(CH_3)_3$). ^{29}Si from the ^{1}H-^{29}Si HMQC NMR (60 MHz, 298 K, C_6D_6): δ 6.0 (s, 2x *Si*-$(CH_3)_3$), –49.2(s, *Si*-CH_3). HRMS (ESI$^+$, *m/z*): calcd. for $C_{12}H_{34}NO_2Si_3$, $[M+H]^+$ = 306.1741; found = 306.1736. **8b** was isolated as an orange oil in an 80% yield (123.0 mg, 0.40 mmol).

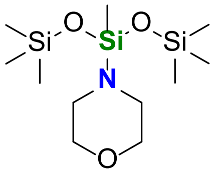

4-(1,1,1,3,5,5,5-heptamethyltrisiloxan-3-yl)morpholine (8c). ^{1}H NMR (300 MHz, 298 K, C_6D_6): δ 3.50 (m, 4H, CH_2-3 and CH_2-5), 2.85 (m, 4H, CH_2-2 and CH_2-6), 0.21 (s, 18H, 2x Si-$(CH_3)_3$), 0.14 (s, 3H, Si-CH_3). ^{13}C{^{1}H} NMR (75 MHz, 298 K, C_6D_6): δ 68.5 (s, 2C, C^3 and C^5), 45.0 (s, 2C, C^2 and C^6), 1.9 (s, Si-CH_3), 1.8 (s, 6C, 2x Si-$(CH_3)_3$). ^{29}Si from the ^{1}H-^{29}Si HMQC NMR (60 MHz, 298 K, C_6D_6): δ 7.1 (s, 2x *Si*-$(CH_3)_3$), –49.1 (s, *Si*-CH_3). HRMS (ESI$^+$, *m/z*): calcd. for $C_{11}H_{30}NO_3Si_3$, $[M+H]^+$ = 308.1533; found = 308.1516. **8c** was isolated as a yellow oil in an 80% yield (123.9 mg, 0.40 mmol).

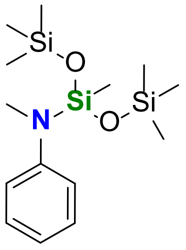

***N*-(1,1,1,3,5,5-heptamethyltrisiloxan)-*N*-methylaniline (8d)**. ^{1}H NMR (300 MHz, 298 K, C_6D_6): δ 7.61 (m, 2H, *m*-(C_6H_5)), 7.18 (m, 2H, *o*-(C_6H_5)), 6.85 (m, 1H, *p*-(C_6H_5)), 2.80 (s, 3H, N-CH_3), 0.67 (s, 3H, Si-CH_3), 0.66 (s, 18H, 2x Si-$(CH_3)_3$). ^{13}C{^{1}H} NMR (75 MHz, 298 K, C_6D_6): δ 149.9 (s, C^{ipso}), 129.0 (s, 2C, *m*-CH-Ar), 119.5 (s, *p*-CH-Ar), 118.2 (s, 2C, *o*-CH-Ar), 34.0 (s, N-CH_3), 1.9 (s, Si-CH_3), 1.8 (s,

6C, 2x Si-(CH_3)$_3$). ^{29}Si from the ^{1}H-^{29}Si HMQC NMR (60 MHz, 298 K, C_6D_6): δ 8.0 (s, 2x *Si*-(CH_3)$_3$), –48.8 (s, N-*Si*-CH_3). HRMS (ESI$^+$, *m/z*): calcd. for $C_{12}H_{23}NO_2Si_3$, [M-2CH$_3$]$^+$ = 297.1037; found = 297.2330. **8d** was isolated as a yellow oil in a 76% yield (124.5 mg, 0.38 mmol).

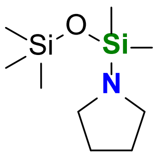

1-(1,1,3,3,3-pentamethyldisiloxan-3-yl)pyrrolidine (9a). ^{1}H NMR (300 MHz, 298 K, C_6D_6): δ 2.98 (m, 4H, C*H*$_2$-2 and C*H*$_2$-5), 1.58 (m, 4H, C*H*$_2$-3 and C*H*$_2$-4), 0.18 (s, 6H, Si-(C*H*$_3$)$_2$), 0.15 (s, 9H, Si-(C*H*$_3$)$_3$). ^{13}C{^{1}H} NMR (75 MHz, 298 K, C_6D_6): δ 46.2 (s, 2C, C^2 and C^5), 27.2 (s, 2C, C^3 and C^4), 2.1 (s, 2C, Si-(CH_3)$_2$), 0.5 (s, 3C, Si-(CH_3)$_3$). ^{29}Si from the ^{1}H-^{29}Si HMQC NMR (60 MHz, 298 K, C_6D_6): δ 5.0 (s, *Si*-(CH_3)$_3$), –13.1 (s, *Si*-CH_3). HRMS (ESI$^+$, *m/z*): calcd. for $C_8H_{20}DNNaOSi_2$, [M-CH$_3$+D+Na]$^+$ = 227.1122; found =226.9515. **9a** was isolated as a yellow oil in a 78% yield (85.7 mg, 0.39 mmol).

7. Miscellaneous reactions

*Reaction of complex **3** with differents equivalents of pyrrolidine*

Under an argon atmosphere, an NMR tube was charged with **3** (6 mg, 0.008 mmol), pyrrolidine (2.6 µL, 0.032 mmol; 0.7 µL, 0.008 mmol) and dissolved in 0.4 mL of benzene-d_6. Then, the resulting mixture was frozen by submerging the NMR tube in liquid nitrogen. The reaction was allowed to warm to r.t. and analyze by NMR spectroscopy (Figures S63-S66).

*Catalytic reaction of pyrrolidine with HSiMe(SiOMe$_3$)$_2$ using 10 mol % of **3***

Under an argon atmosphere, a Young tube was charged with 10 mol% (0.01 mmol) of **3** (7.6 mg) and hexamethylbenzene (4.0 mg, 0.025 mmol) as internal standard (IS) and dissolved in 0.4 mL of benzene-d_6. Then, pyrrolidine (8 µL, 0.1 mmol) and HSiMe(SiOMe$_3$)$_2$ (27 µL, 0.1 mmol) were added at room temperature (r.t.) and the resulting mixture was frozen by submerging the Young tube in liquid nitrogen (Scheme S10). The reaction was allowed to warm to r.t. and analyze by NMR spectroscopy and HR-MS (Figures S67-S69).

Scheme S10. Catalytic reaction of pyrrolidine with HSiMe(SiOMe$_3$)$_2$, using **3** (10 mol%) in C_6D_6 at r.t.

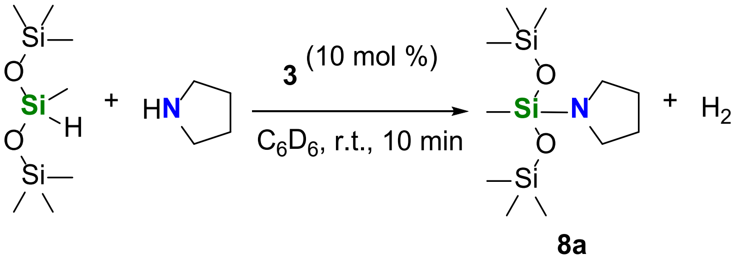

8a

8. DFT calculations

8.1. Computational methods

Optimization of geometries was performed using Gaussian 16 (revision C.01) using an ultrafine integration grid (int=ultrafine).[S5] Geometry optimizations and frequency calculations were performed using the B3LYP[S6] functional with def2tzvp[S7] (Rh) and 6-31G**[S8] (all other atoms) basis sets (unless specified) with solvent corrections (PCM, benzene, ε = 2.2706)[S9] and an empirical dispersion correction (Grimme, GD3)[S10] in all calculations. Frequency analyses for all geometries were performed using the enhanced criteria to confirm the nature of the structures either minima (no imaginary frequency) or transition states (only one imaginary frequency). Natural Bond Orbital analysis was carried out using NBO 7.0[S11] using same level of theory. QTAIM analyses was performed using Multiwfn program[S12] and graphics of the results generated with VMD 1.9.1.[S13]

8.2. Agostic interaction

Based-Stabilized Silylene

NBO and QTAIM data for **2** and **3**.

Table S7. NBO and QTAIM selected features for the Rh–Si bond in **2**, **3** and **INT5**. a.u. = hartree·Å^{-3}.

Compound	Δq (Rh–Si) (e)	WBI (Rh–Si)	$\nabla^2\rho(r)$ (e·Å^{-5})	$\rho(r)$ (e·Å^{-3})	$\varepsilon(r)$	$\|V(r)\|/G(r)$
**2**	1.98	0.66	-0.104	0.100	0.015	2.537
**3**	1.92	0.65	-0.104	0.099	0.013	2.548
**INT5**	1.90	0.68	-0.107	0.093	0.041	2.657

	q(Rh) (e)	q(Si) (e)	$E(r)$ (a.u.)	$V(r)$ (a.u.)
**2**	-0.175	1.805	-0.074	-0.122
**3**	-0.120	1.798	-0.073	-0.120
**INT5**	-0.083	1.820	-0.067	-0.108

Table S8. NBO and QTAIM selected features for the Si–O bond in **2**, **3** and **INT5**. a.u. = hartree·Å^{-3}.

Compound	Δq (Si–O) (e)	WBI (Si–O)	$\nabla^2\rho(r)$ (e·Å^{-5})	$\rho(r)$ (e·Å^{-3})	$\varepsilon(r)$	$\|V(r)\|/G(r)$
**2**	2.64	0.45	0.593	0.099	0.008	1.121
**3**	2.63	0.46	0.608	0.101	0.016	1.121
**INT5**	2.71	0.48	0.696	0.106	0.035	1.104

	q(O) (e)	q(Si) (e)	$E(r)$ (a.u.)	$V(r)$ (a.u.)	$\Delta E^{(2)}$ LP (O)→LV (Si) (kcal·mol^{-1})
**2**	-0.833	1.805	-0.020	-0.189	137.58
**3**	-0.830	1.798	-0.021	-0.194	139.23
**INT5**	-0.894	1.820	-0.020	-0.214	142.76

Agostic interaction

In complexes **2** and **3**, Quantum Theory of Atom in Molecules (QTAIM) method locates a bond critical point (BCP) between the H from the 8-Me of the ligand and Rh center and a bond path (BP) running between both atoms, which confirms the occurrence of the proposed agostic interaction (Figure S8, Table S9).

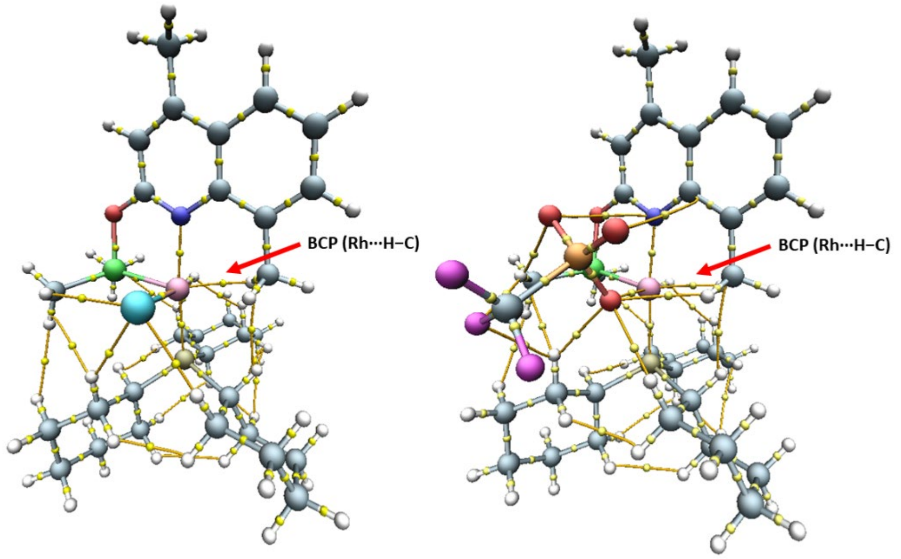

Figure S8. Contour line diagrams $\nabla^2\rho$(r) for complexes **2** (left) and **3** (right) in the Rh···H–C plane. Yellow spheres denote the located bond critical points (BCP).

Table S9. Results of the QTAIM and NBO analysis for **2** and **3**.

	**2**	**3**
r(Rh···H) / Å	2.159	2.147
ρ(r) / e·Å^{-3}	0.033	0.034
∇^2ρ(r) / e·Å^{-5}	0.108	0.113
ε	0.341	0.371
Δ$E^{(2)}$ / σ(C–H)→δ(Rh) and δ(Rh)→σ*(C–H) / kcal·mol^{-1}	–9.45	–9.51

8.3. Mechanistic studies

*Calculated transformation of **INT1** into **INT6***

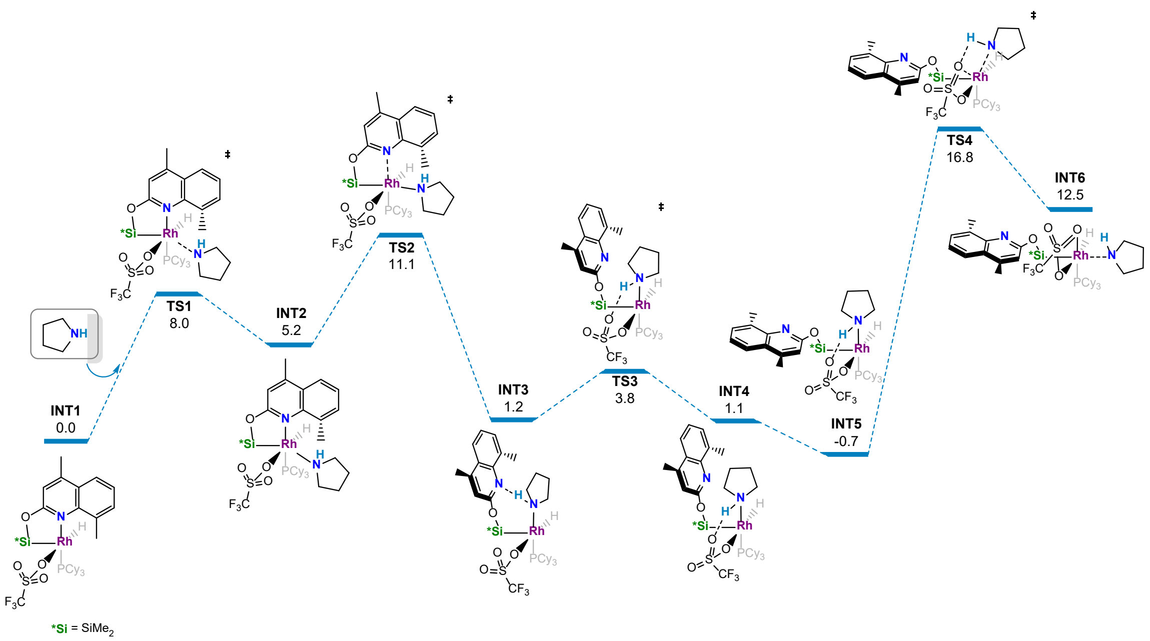

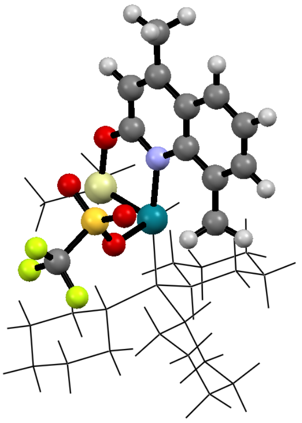

INT1

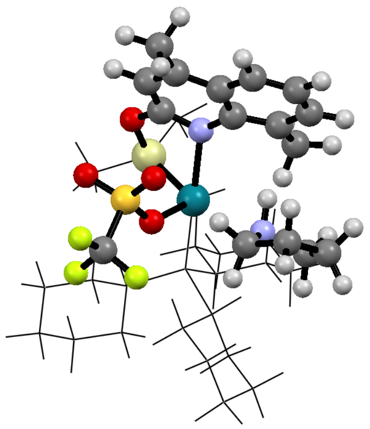

TS1

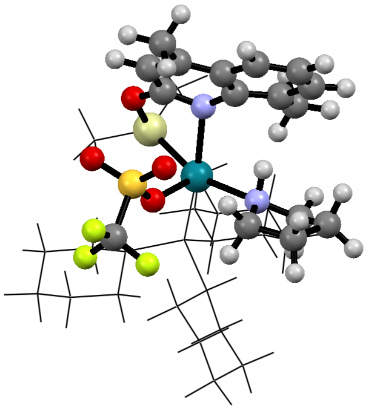

INT2

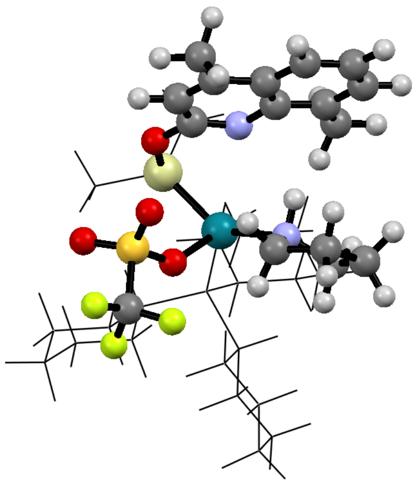

TS2

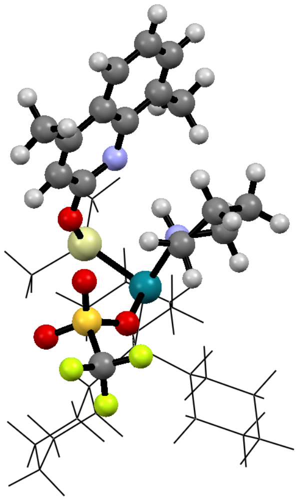

INT3

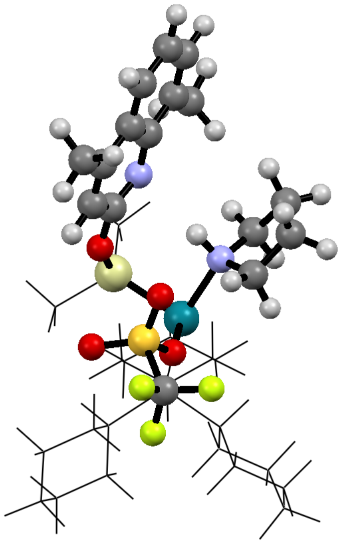

TS3

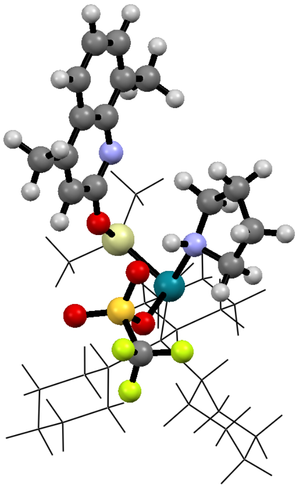

INT4

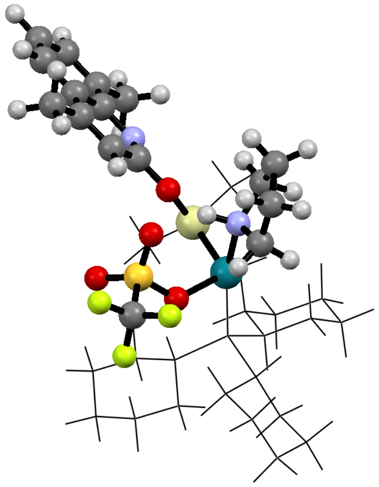

INT5

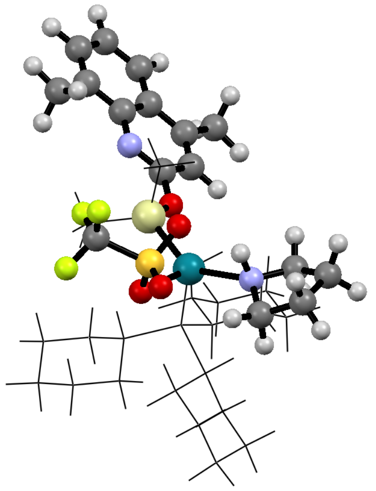

TS4

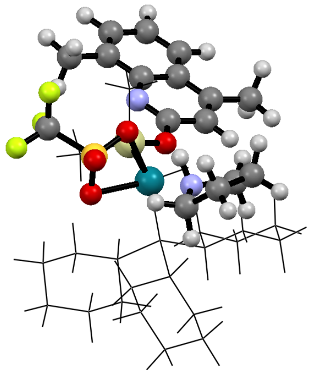

INT6

Figure S9. Calculated transformation of **INT1** into **INT6**. All energies in kcal·mol^{-1}.

Calculated mechanism for Cross-Dehydrogenative coupling of pyrrolidine with siloxane

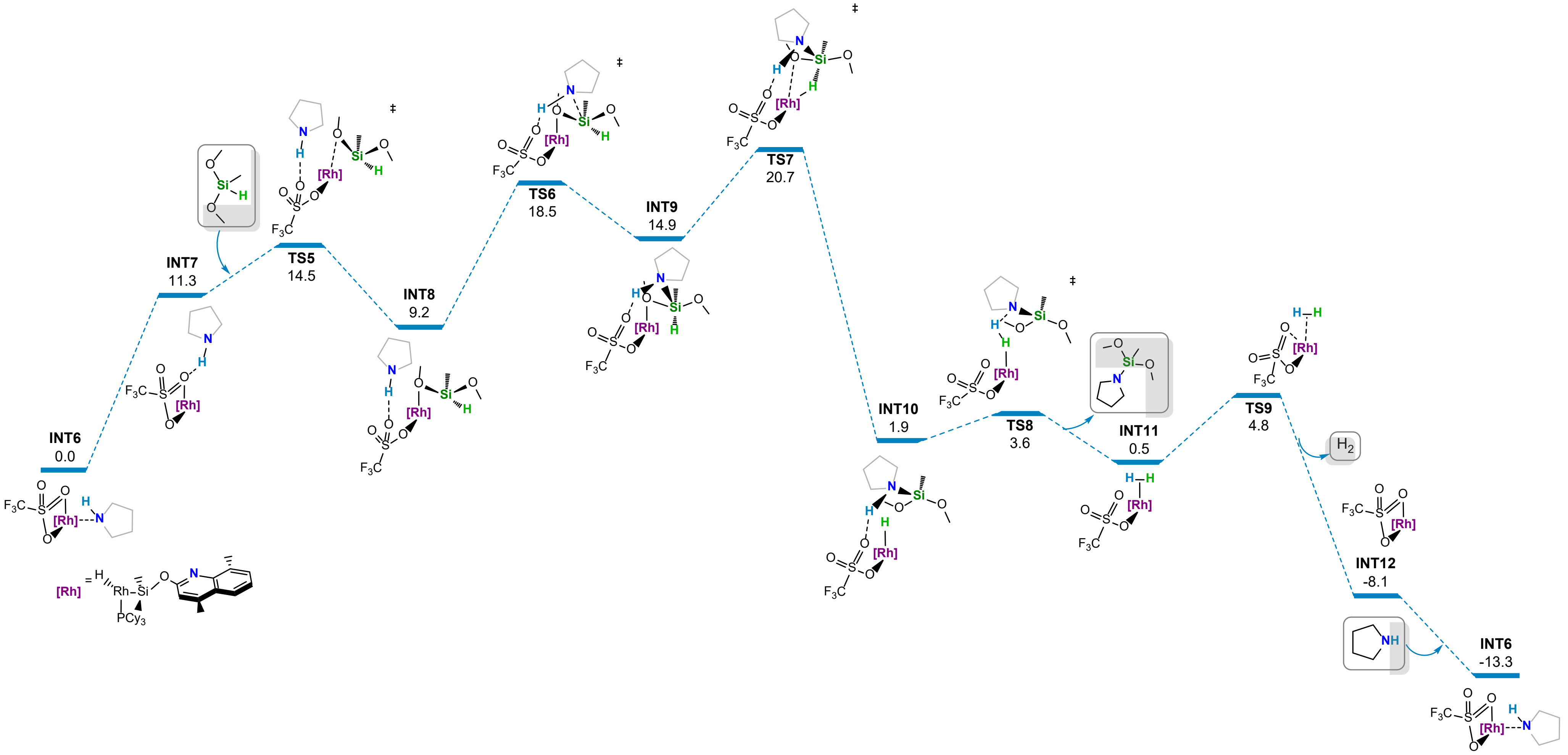

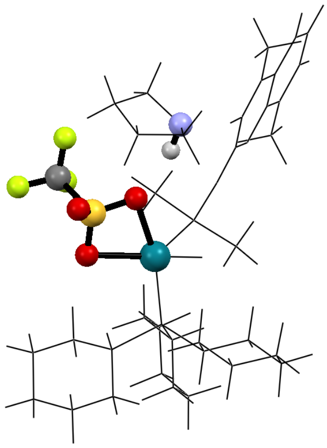

INT7

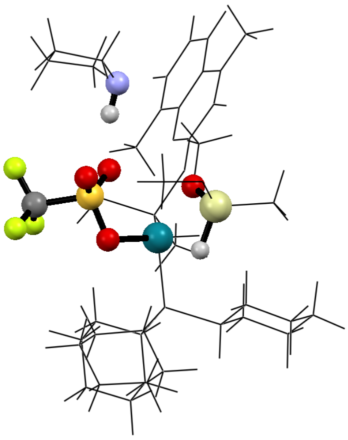

TS5

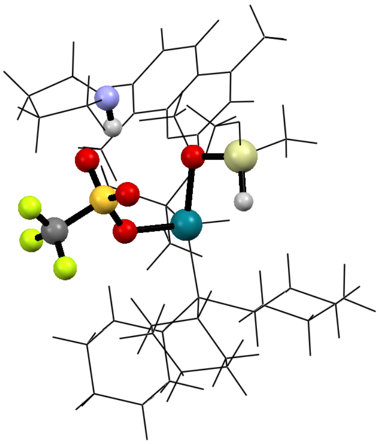

INT8

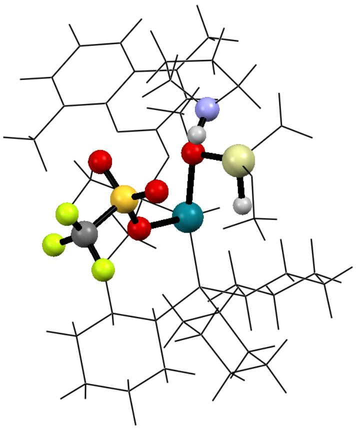

TS6

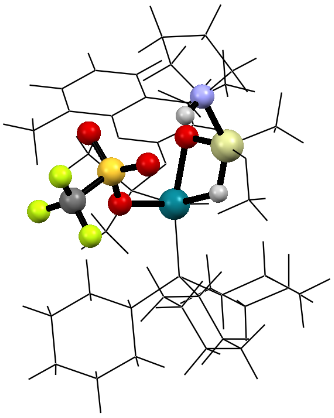

INT9

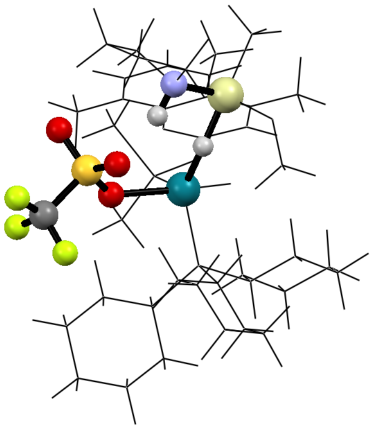

TS7

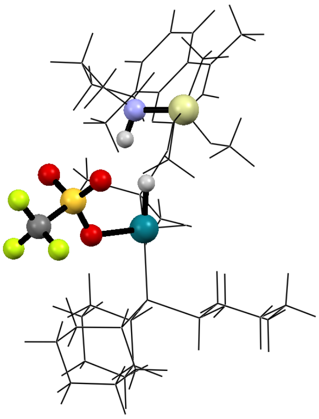

INT10

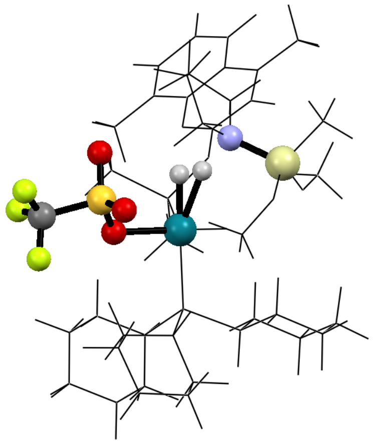

TS8

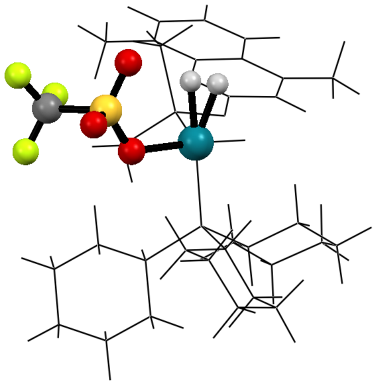

INT11

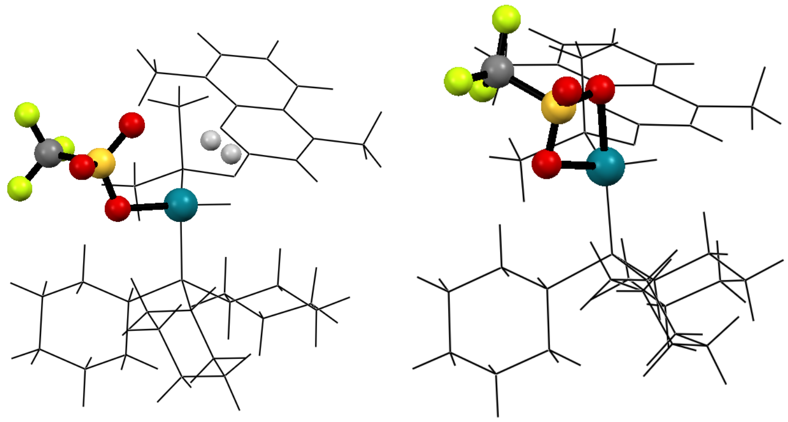

TS9 **INT12**

Figure S10. Calculated mechanism for Cross-Dehydrogenative coupling of pyrrolidine with siloxane. All energies in kcal·mol^{-1}.

Organic molecules

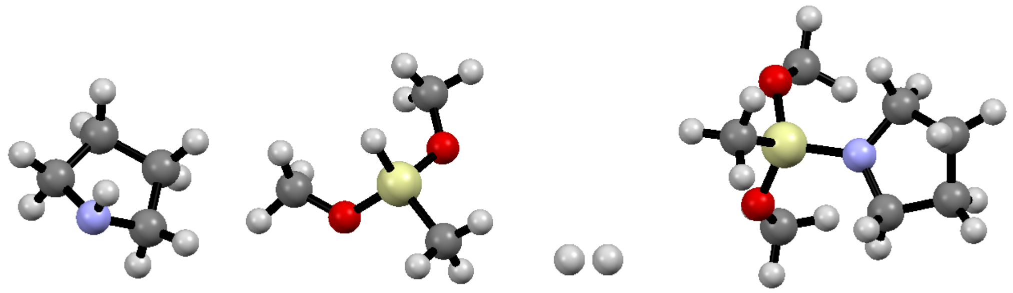

Pyrrolidine **Siloxane** **H_2** **Siloxazane**

Figure S11. Representation of the calculated organic molecules.

Other DFT calculations

- Ligand $CF_3SO_3^-$ *vs* $CH_3SO_3^-$

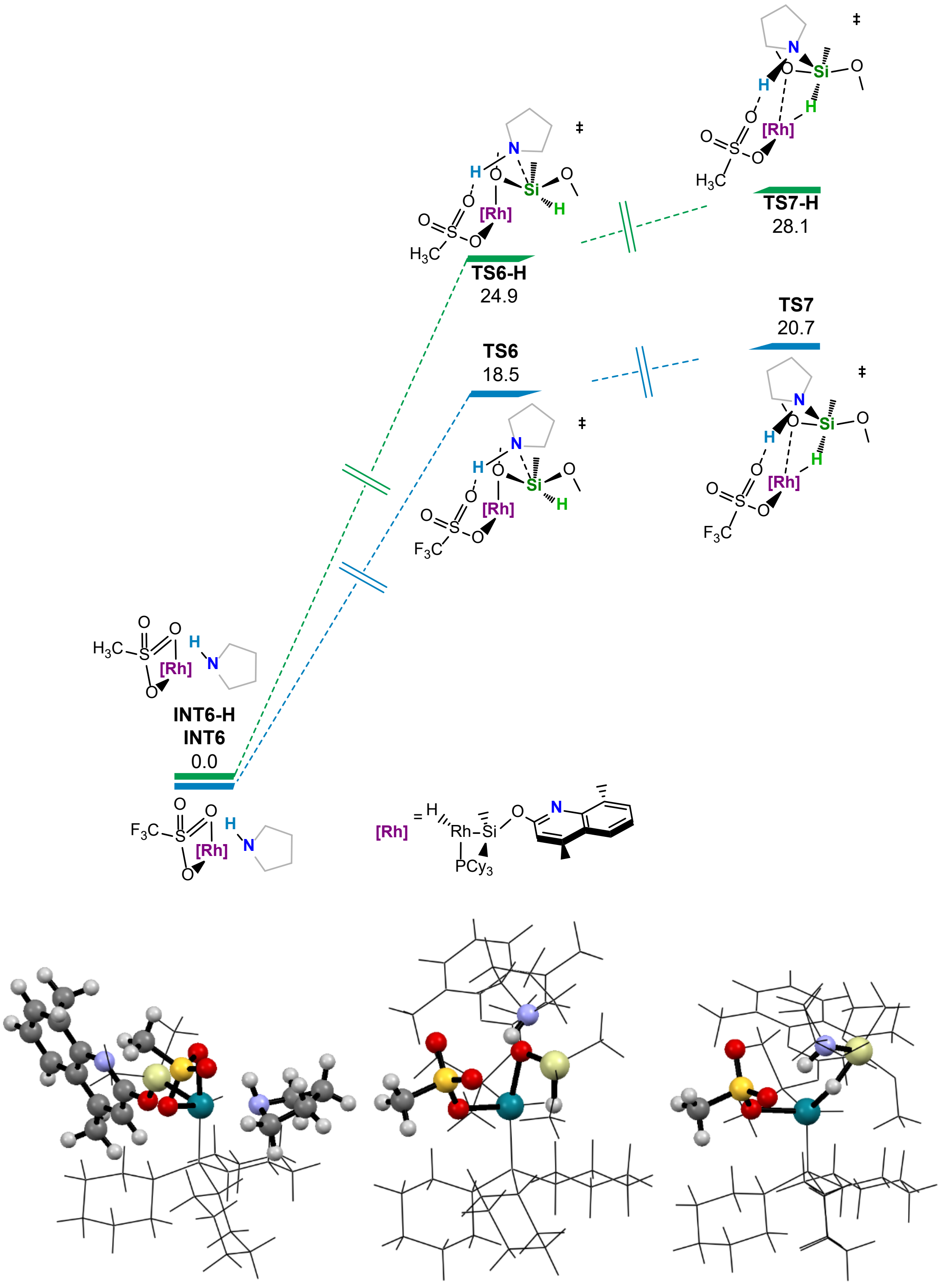

Figure S12. Calculated geometries for **INT6-H**, **TS6-H** and **TS7-H**. All energies in kcal·mol^{-1}.

- Si–H formation

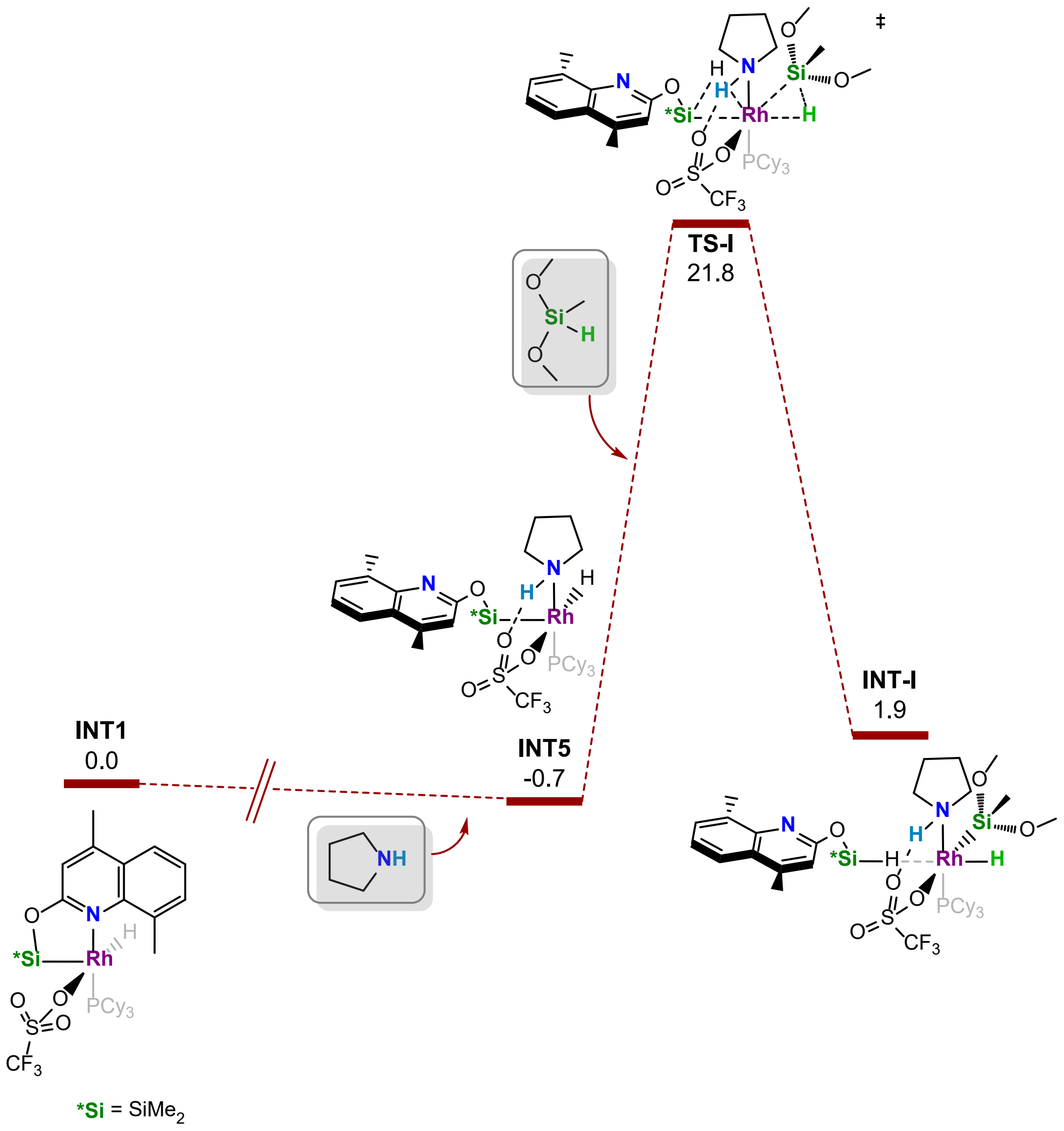

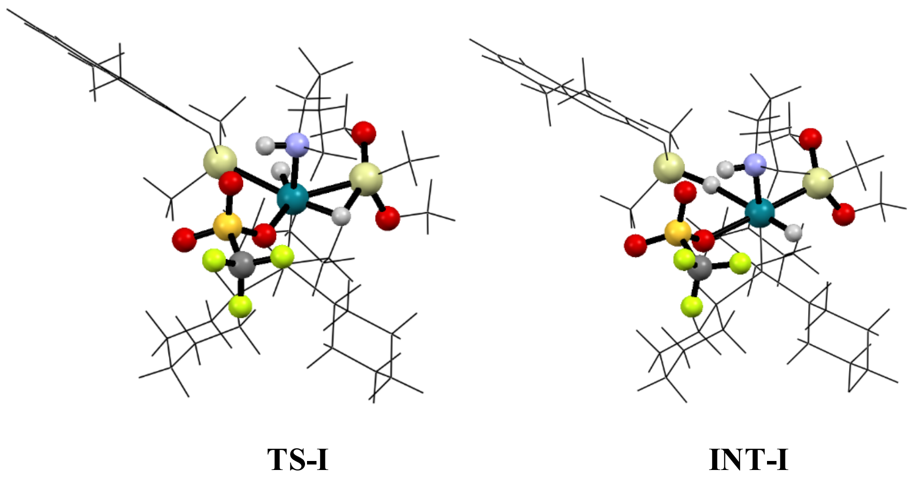

Figure S13. Calculated geometries for **TS-I** and **INT-I**. All energies in kcal·mol^{-1}.

- Si–OTf formation

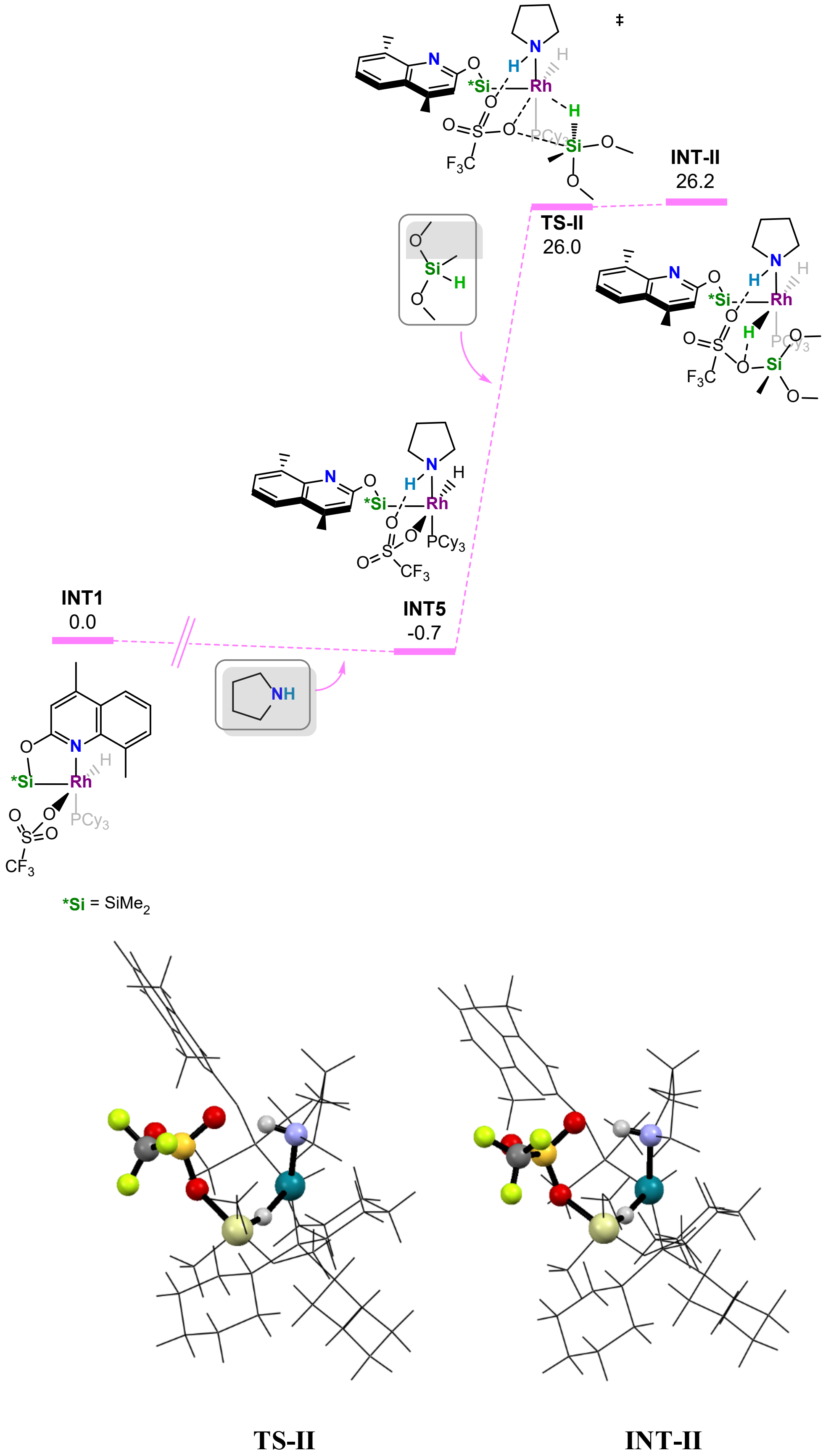

Figure S14. Calculated geometries for **TS-II** and **INT-II**. All energies in kcal·mol^{-1}.

- **P–H formation**

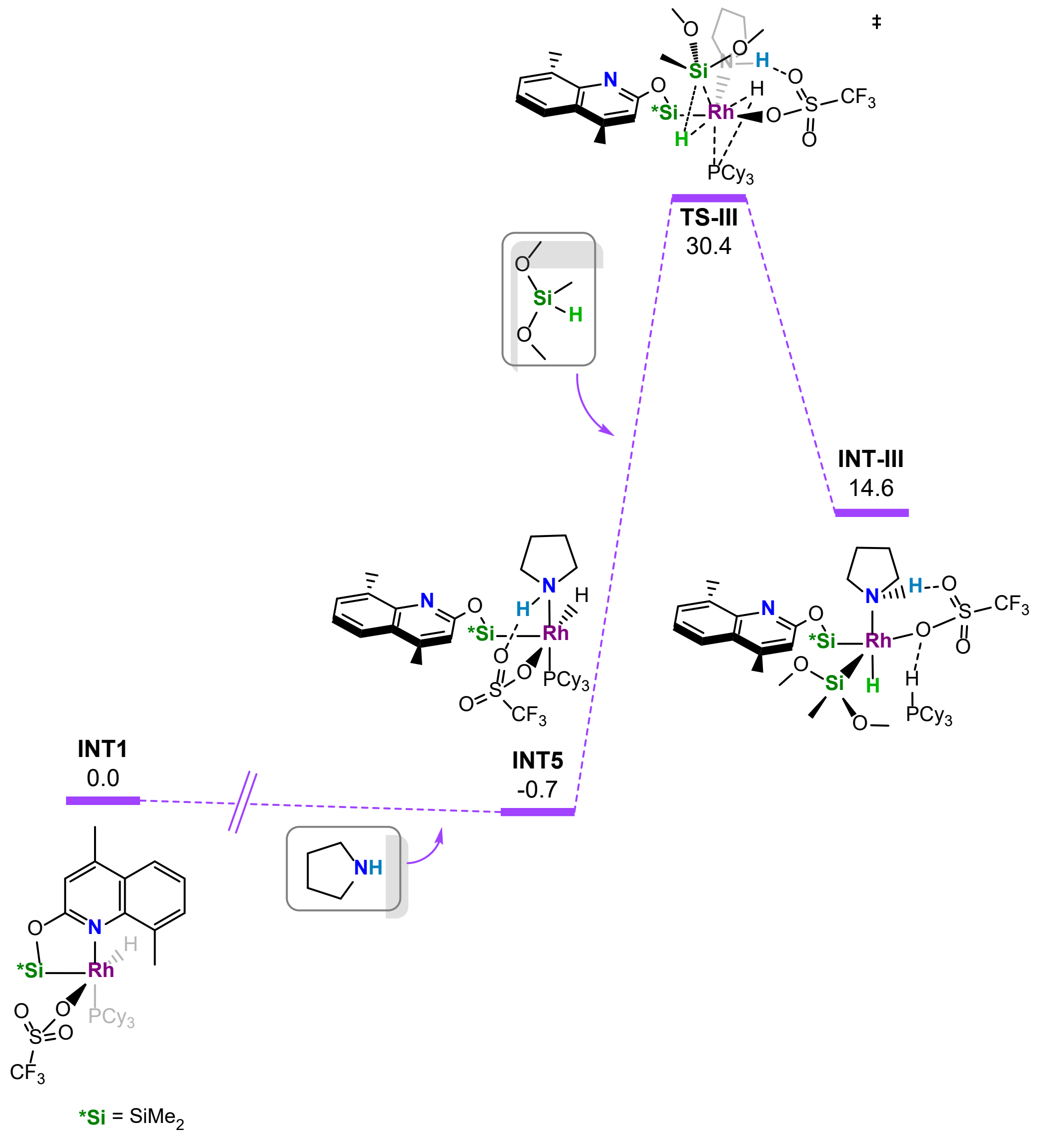

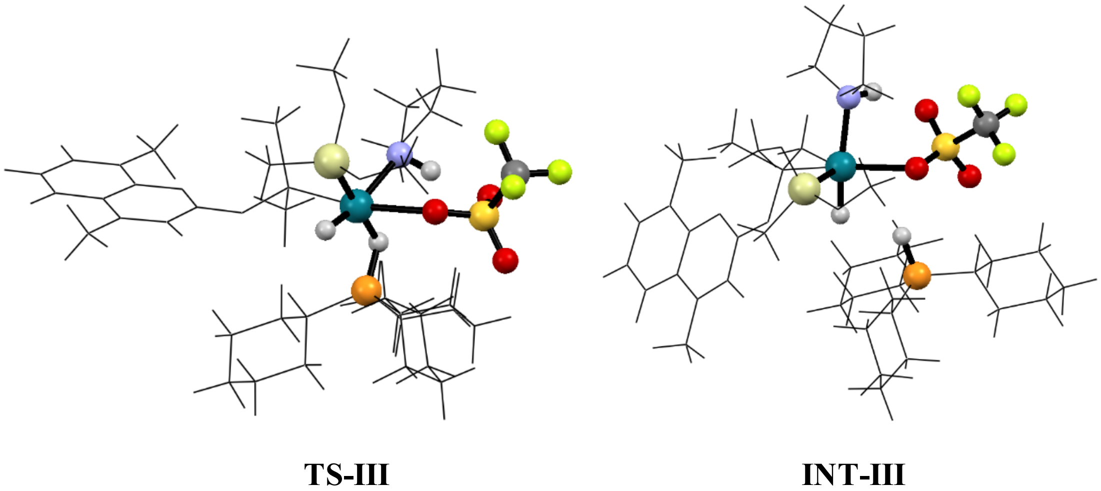

Figure S15. Calculated geometries for **TS-III** and **INT-III**. All energies in kcal·mol^{-1}.

9. References

S1. Hellwich, K.-H.; Hartshorn, R. M.; Yerin, A.; Damhus, T.; Hutton, A. T. Brief guide to the nomenclature of organic chemistry (IUPAC Technical Report). *Pure Appl. Chem.* **2020**, *92*, 527–539.

S2. Van Der Ent, A.; Onderdelinden, A. L.; Schunn, R. A. Chlorobis (cyclooctene) rhodium (I) and–iridium (I) complexes. *Inorg. Synth.* **1990**, *28*, 90–92.

S3. Kozuch, S.; Martin, J. M. L. "Turning over" definitions in catalytic cycles, *ACS Catal.* **2012**, *2*, 2787–279.

S4. The reactions were carried out in a Man on the Moon™ X102 microreactor, https://manonthemoontech.com/, and monitored by H_2 evolution during the CDC reaction.

S5. Frisch, M. J.; Trucks, G. W.; Schlegel, H. B.; Scuseria, G. E.; Robb, M. A.; Cheeseman, J. R.; Scalmani, G.; Barone, V.; Petersson, G. A.; Nakatsuji, H.; Li, X.; Caricato, M.; Marenich, A. V.; Bloino, J.; Janesko, B. G.; Gomperts, R.; Mennucci, B.; Hratchian, H. P.; Ortiz, J. V.; Izmaylov, A. F.; Sonnenberg, J. L.; Williams-Young, D.; Ding, F.; Lipparini, F.; Egidi, F.; Goings, J.; Peng, B.; Petrone, A.; Henderson, T.; Ranasinghe, D.; Zakrzewski, V. G.; Gao, J.; Rega, N.; Zheng, G.; Liang, W.; Hada, M.; Ehara, M.; Toyota, K.; Fukuda, R.; Hasegawa, J.; Ishida, M.; Nakajima, T.; Honda, Y.; Kitao, O.; Nakai, H.; Vreven, T.; Throssell, K.; Montgomery, J. A. Jr.; Peralta, J. E.; Ogliaro, F.; Bearpark, M. J.; Heyd, J. J.; Brothers, E. N.; Kudin, K. N.; Staroverov, V. N.; Keith, T. A.; Kobayashi, R.; Normand, J.; Raghavachari, K.; Rendell, A. P.; Burant, J. C.; Iyengar, S. S.; Tomasi, J.; Cossi, M.; Millam, J. M.; Klene, M.; Adamo, C.; Cammi, R.; Ochterski, J. W.; Martin, R. L.; Morokuma, K.; Farkas, O.; Foresman, J. B.; Fox, D. J. Gaussian 16 (Revision C.O1), Gaussian Inc., Wallingford CT, 2016.

S6. Stephens, P. J.; Devlin, F. J.; Chabalowski, C. F.; Frisch, M. J. Ab initio calculation of vibrational absorption and circular dichroism spectra using density functional force fields. *J. Phys. Chem.* **1994**, *98*, 11623–11627.

S7. (a) Weigend, F.; Ahlrichs, R. Balanced basis sets of split valence, triple zeta valence and quadruple zeta valence quality for H to Rn: Design and assessment of accuracy. *Phys. Chem. Chem. Phys.* **2005**, *7*, 3297–3305. (b) Weigend, F. Accurate Coulomb-fitting basis sets for H to Rn. *Phys. Chem. Chem. Phys.* **2006**, *8*, 1057–1065.

S8. (a) Petersson, G. A.; Bennett, A.; Tensfeldt, T. G.; Al-Laham, M. A.; Shirley, W. A.; Mantzaris, J. A complete basis set model chemistry. I. The total energies of closed-shell atoms and hydrides of the first-row elements. *J. Chem. Phys.* **1988**, *89*, 2193–2218. (b) Petersson, G. A.; Al-Laham, M. A. A complete basis set model chemistry. II. Open-shell systems and the total energies of the first-row atoms. *J. Chem. Phys.* **1991**, *94*, 6081–6090.

S9. Tomasi, J.; Mennucci, B.; Cammi, R. Quantum mechanical continuum solvation models. *Chem. Rev.* **2005**, *105*, 2999–3093.

S10. Grimme, S.; Antony, J.; Ehrlich, S.; Krieg, H. A consistent and accurate *ab initio* parametrization of density functional dispersion correction (DFT-D) for the 94 elements H-Pu. *J. Chem. Phys.* **2010**, *132*, 154104.

S11. Glendening, E. D.; Badenhoop, J. K.; Reed, A. E.; Carpenter, J. E.; Bohmann, J. A.; Morales, C. M.; Karafiloglou, P.; Landis, C. R.; Weinhold, F. NBO 7.0, Theoretical Chemistry Institute, University of Wisconsin, Madison (2018).

S12. (a) Lu, T.; Chen, F. Multiwfn: A multifunctional wavefunction analyzer. *J. Comput. Chem.* **2012**, *33*, 580–592. (b) Lu, T. A comprehensive electron wavefunction analysis toolbox for chemists, Multiwfn. *J. Chem. Phys.* **2024**, *161*, 082503.

S13. Humphrey, W.; Dalke, A.; Schulten, K. VMD: visual molecular dynamics. *J. Molec. Graphics* **1996**, *14*, 33–38.

S14. Julian, A.; Polo, V.; Jaseer, E. A.; Fernández-Alvarez, F. J.; Oro, L. A. Solvent-free iridium-catalyzed reactivity of CO_2 with secondary amines and hydrosilanes. *ChemCatChem* **2015**, *7*, 3895–3902.

10. NMR spectra

10.1. NMR spectra of Rh–complexes

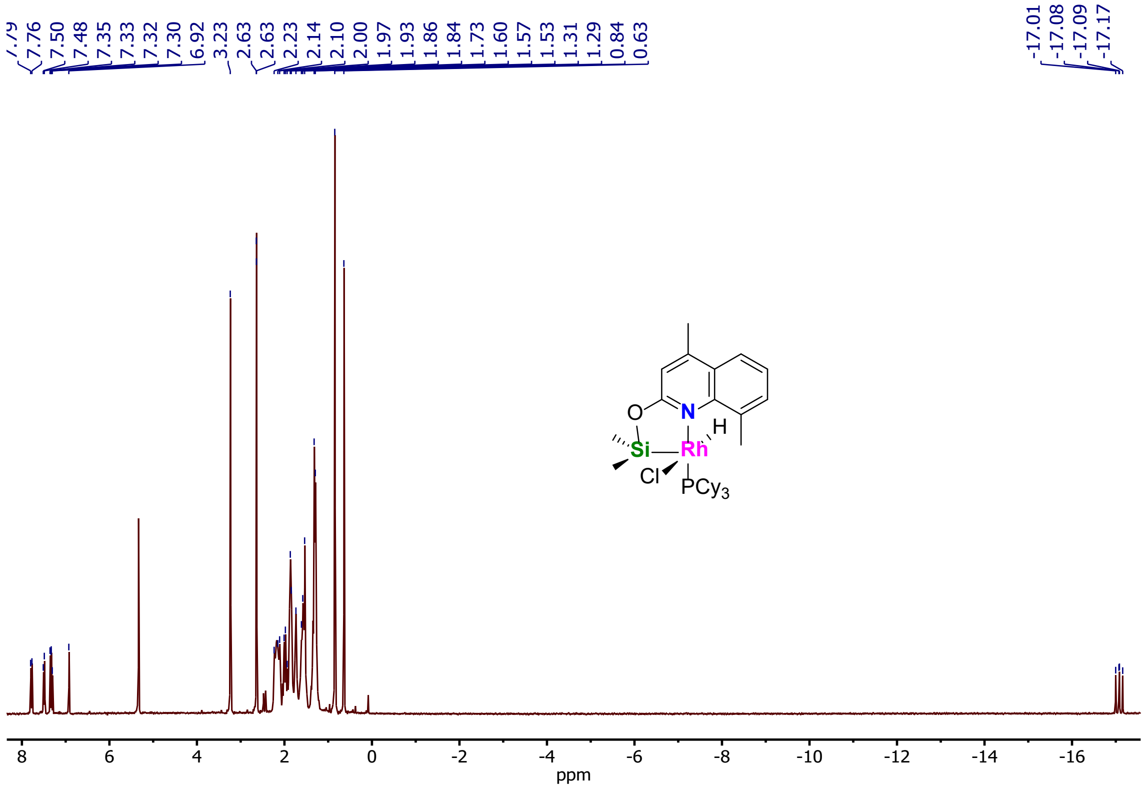

Figure S16. ^{1}H NMR spectrum of **2** in CD_2Cl_2 (300 MHz, 298K).

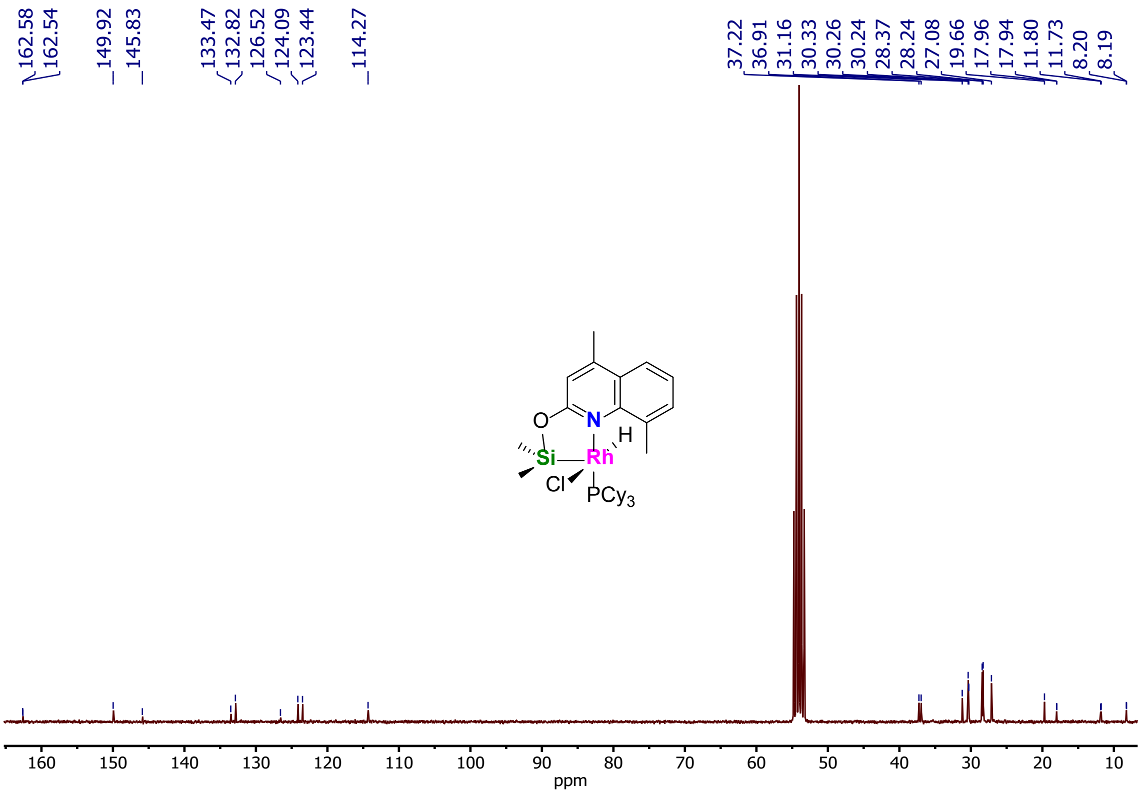

Figure S17. $^{13}C\{^{1}H\}$ NMR spectrum of **2** in CD_2Cl_2 (75 MHz, 298K).

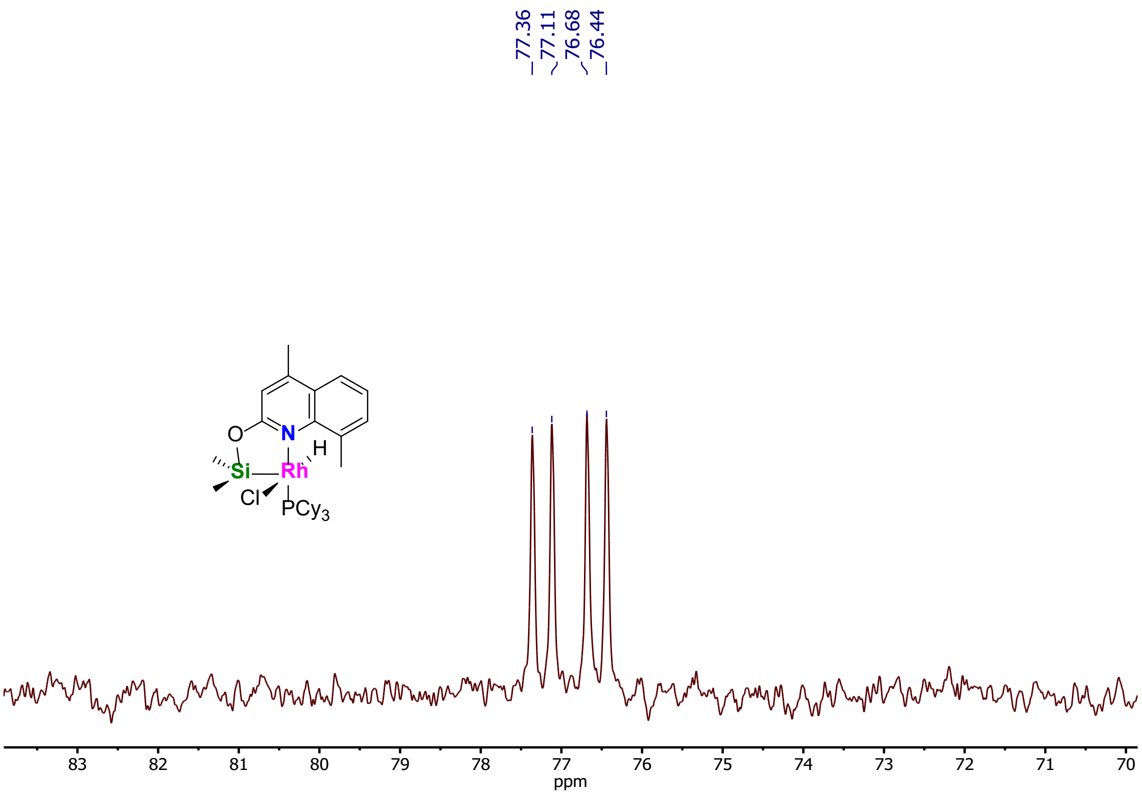

Figure S18. $^{29}Si\{^{1}H\}$ NMR spectrum of **2** in CD_2Cl_2 (60 MHz, 298K).

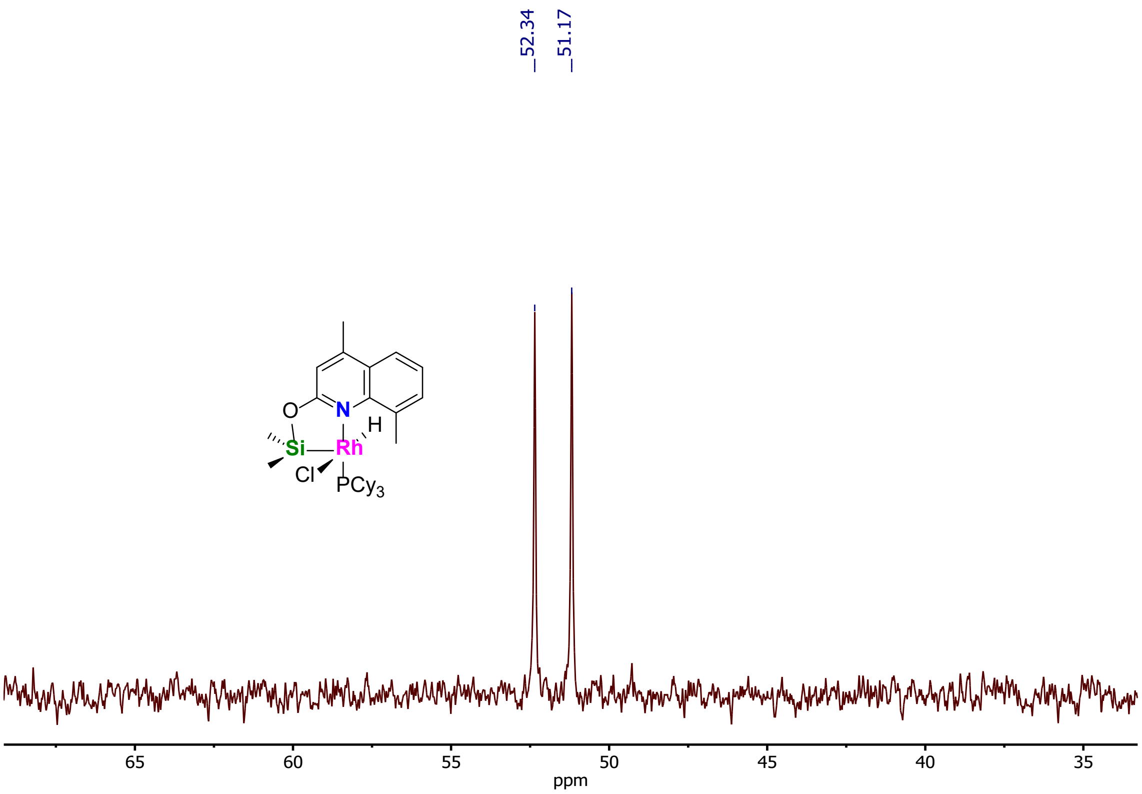

Figure S19. $^{31}P\{^{1}H\}$ NMR spectrum of **2** in CD_2Cl_2 (121 MHz, 298K).

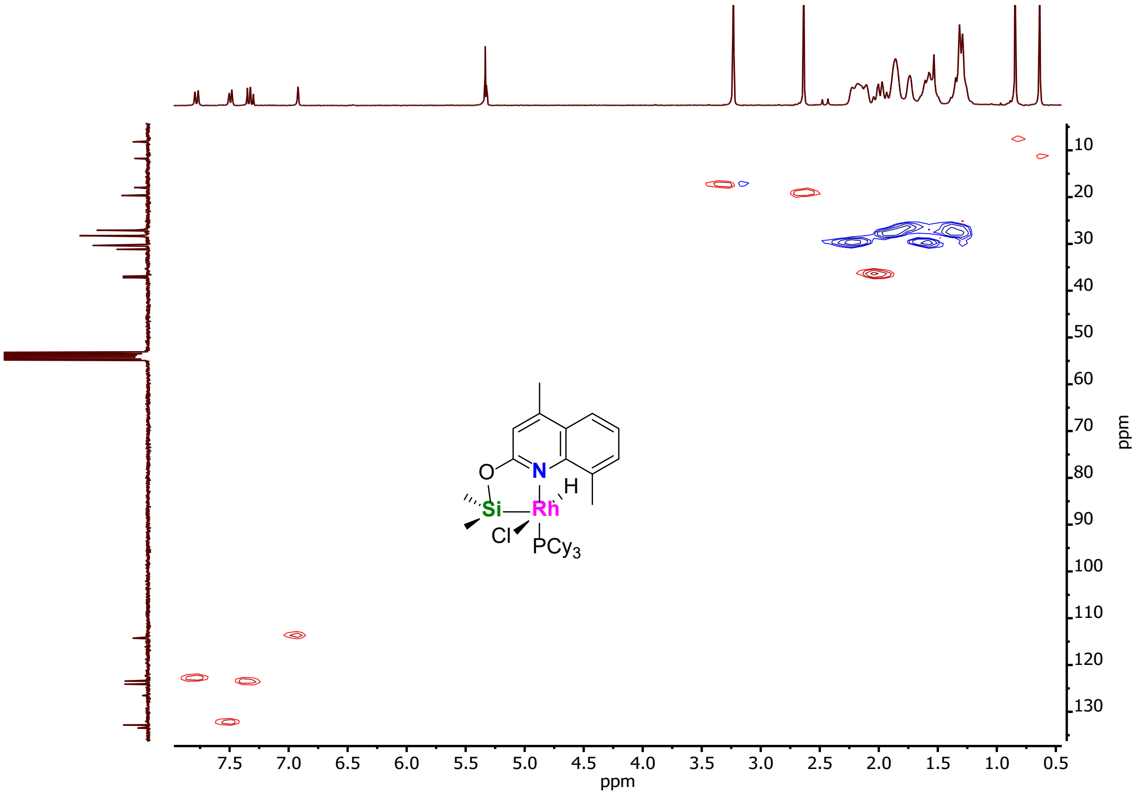

Figure S20. ^{1}H-^{13}C HSQC NMR spectrum of **2** in CD_2Cl_2 (298K).

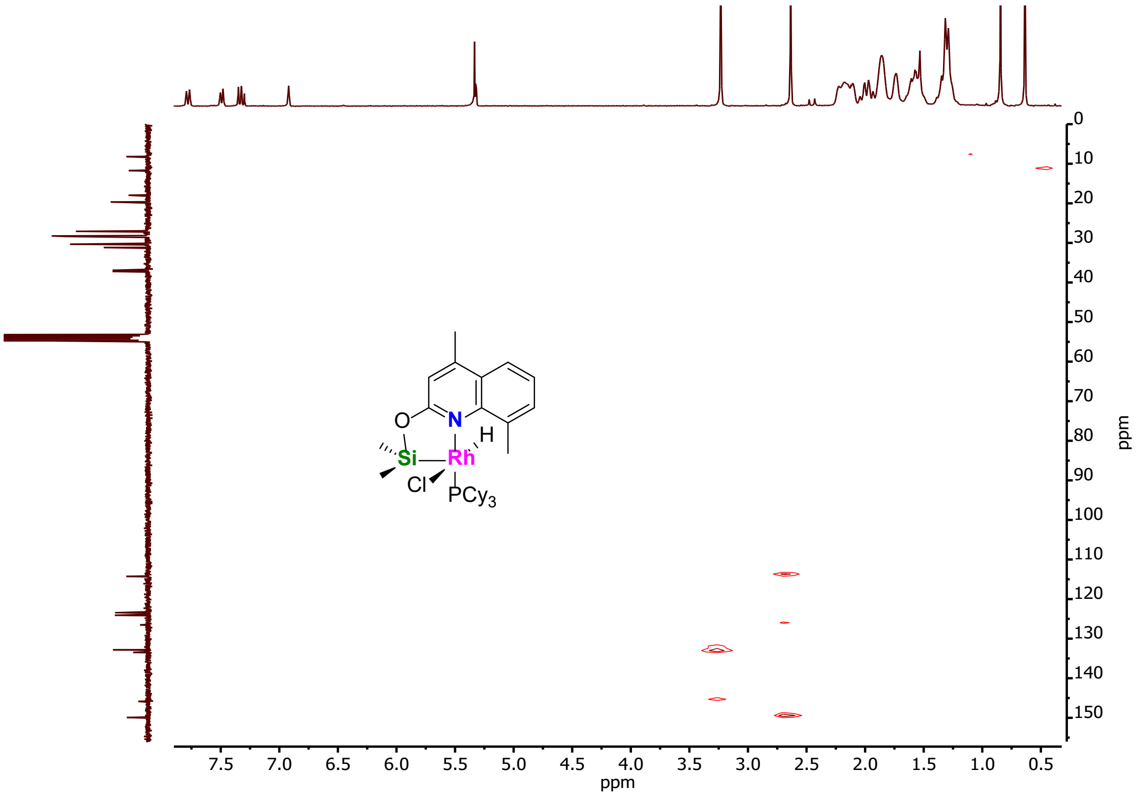

Figure S21. ^{1}H-^{13}C HMBC NMR spectrum of **2** in CD_2Cl_2 (298K).

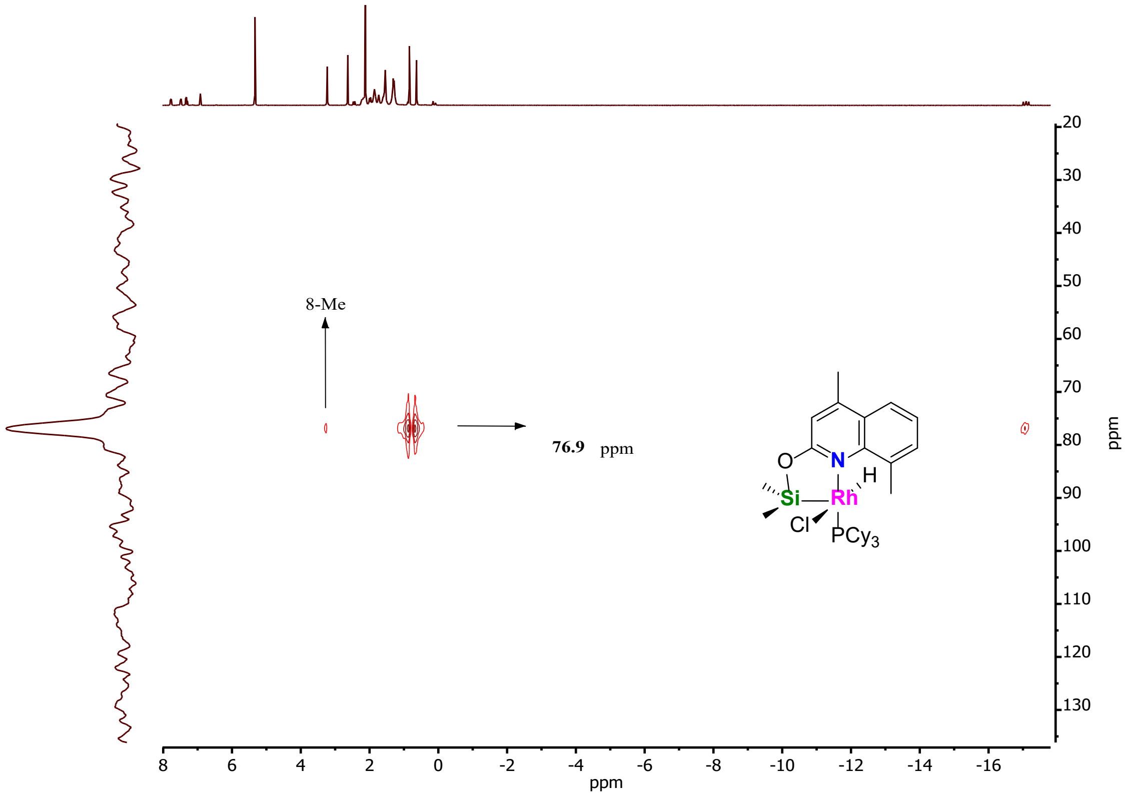

Figure S22. ^{1}H-^{29}Si HMQC NMR spectrum of **2** in CD_2Cl_2 (298K).

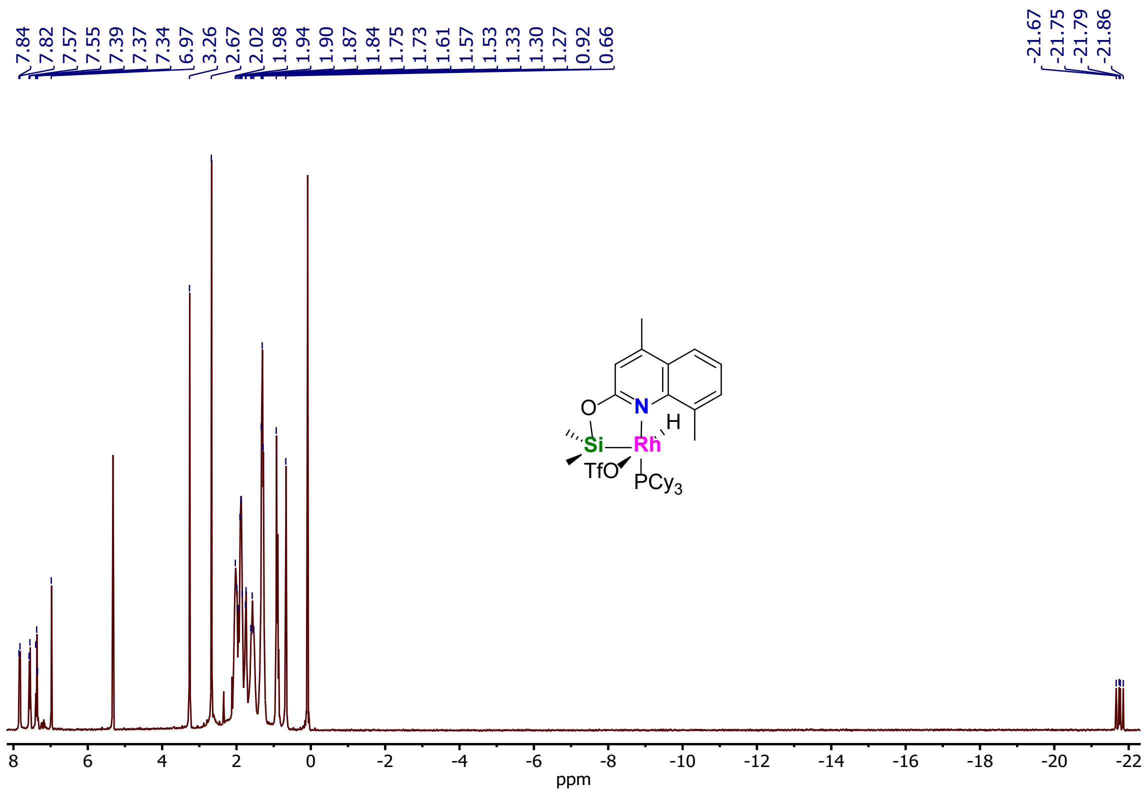

Figure S23. ^{1}H NMR spectrum of **3** in CD_2Cl_2 (300 MHz, 298K).

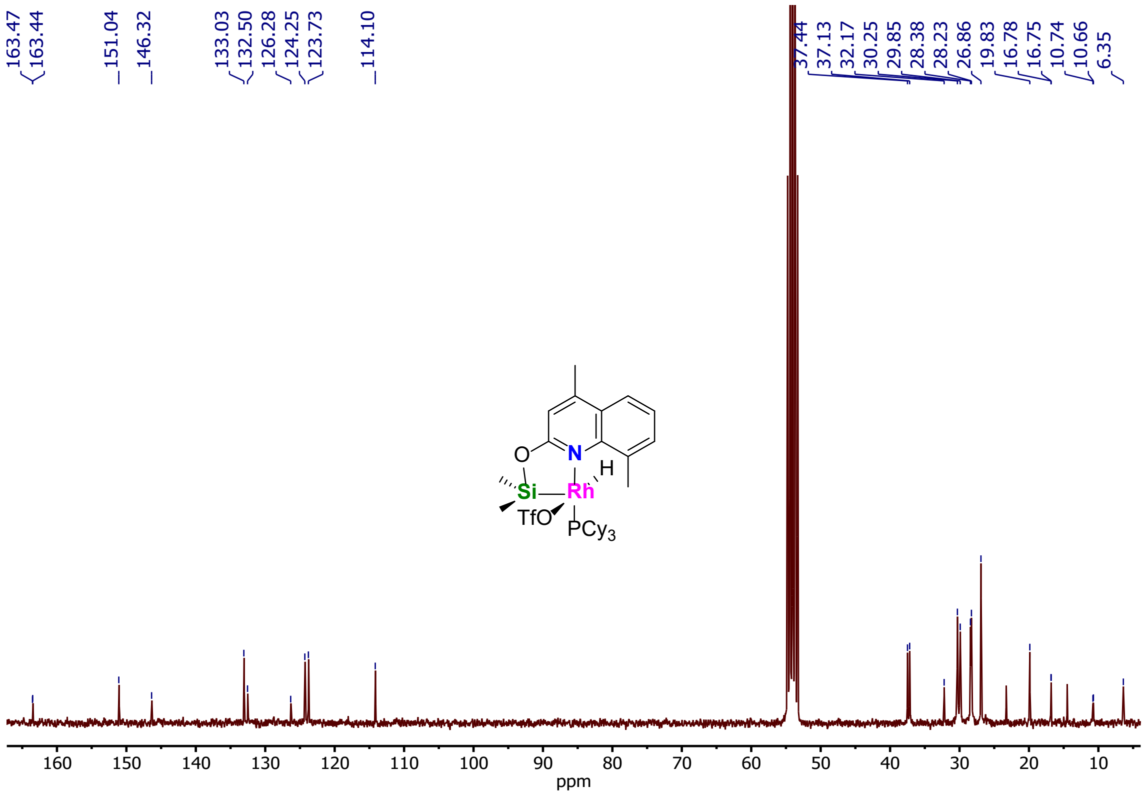

Figure S24. $^{13}C\{^{1}H\}$ NMR spectrum of **3** in CD_2Cl_2 (75 MHz, 298K).

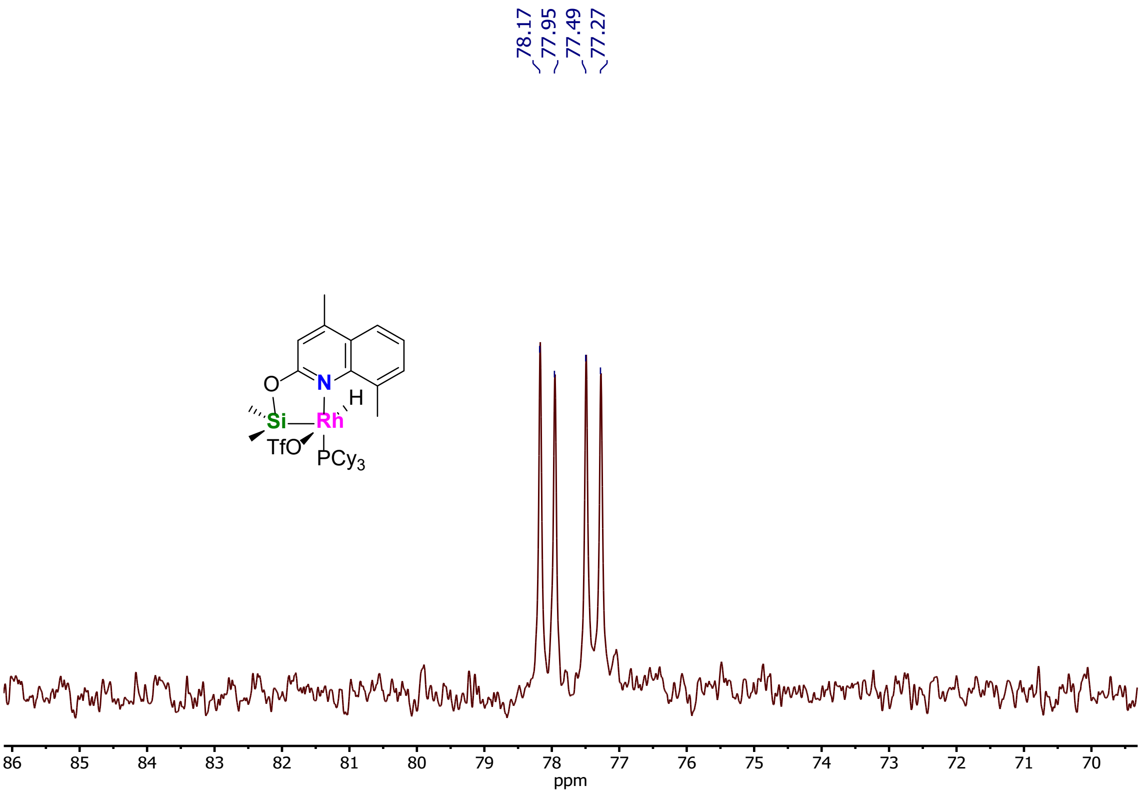

Figure S25. $^{29}Si\{^{1}H\}$ NMR spectrum of **3** in CD_2Cl_2 (60 MHz, 298K).

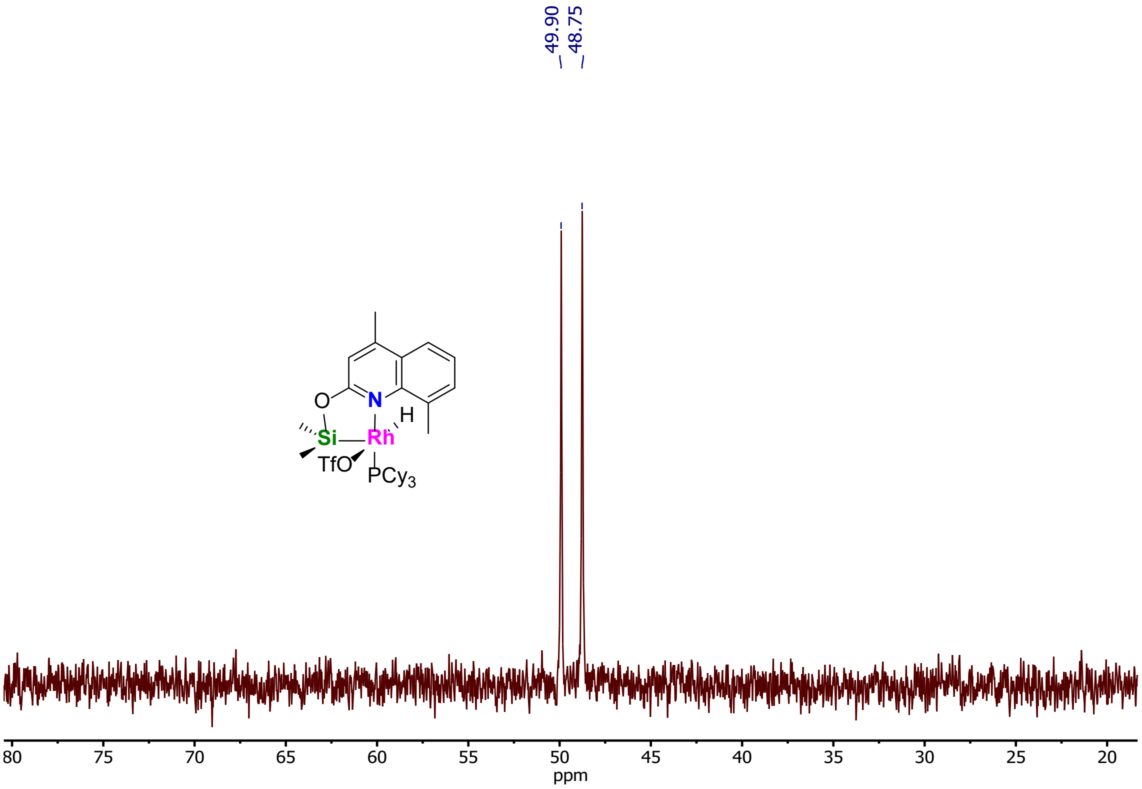

Figure S26. $^{31}P\{^{1}H\}$ NMR spectrum of **3** in CD_2Cl_2 (121 MHz, 298K).

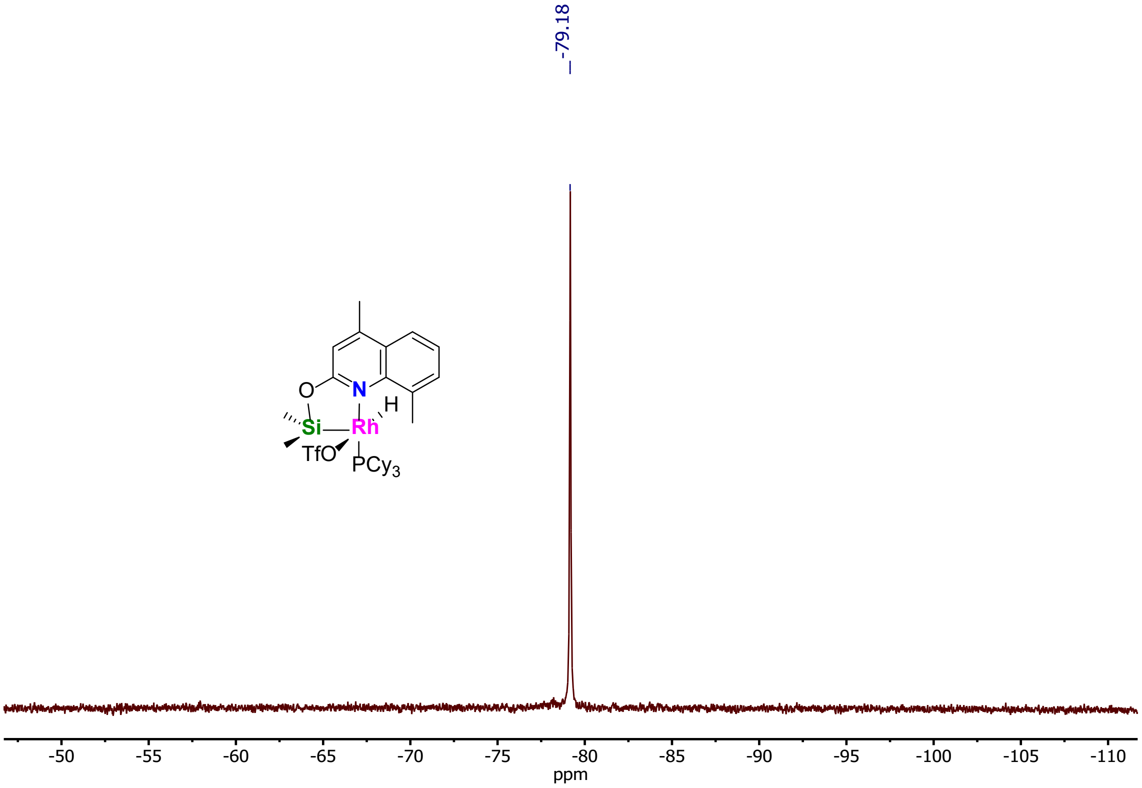

Figure S27. ^{19}F NMR spectrum of **3** in CD_2Cl_2 (282 MHz, 298K).

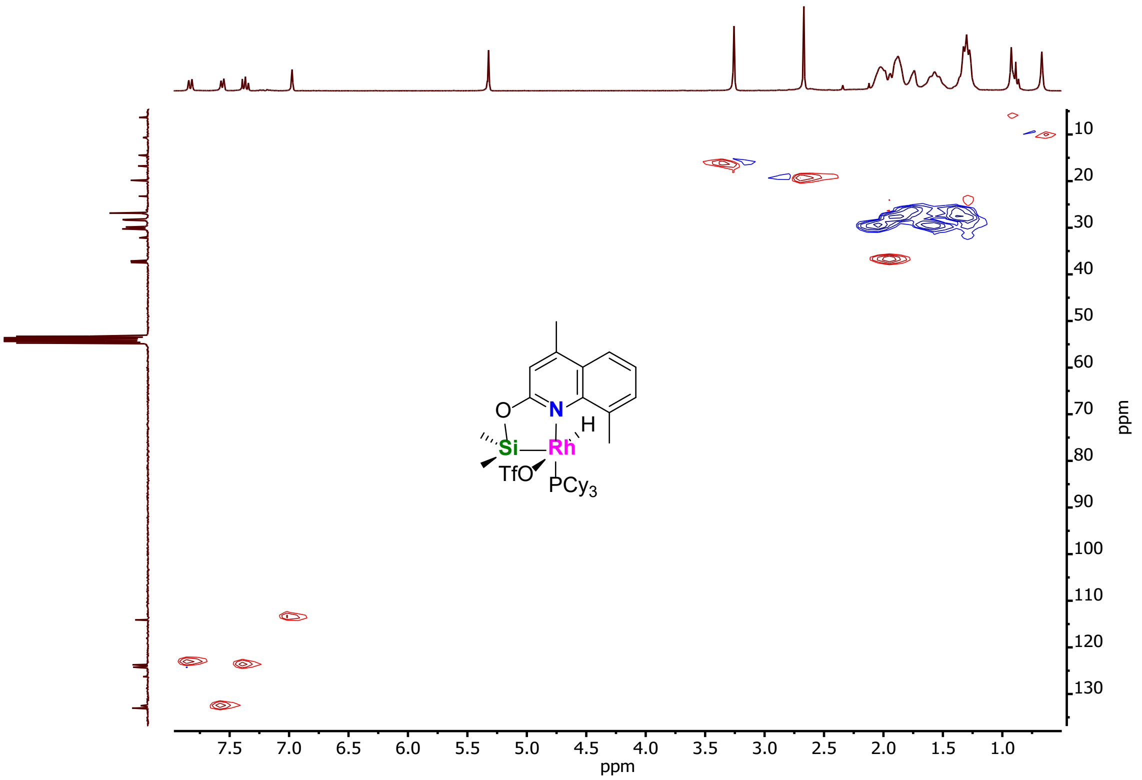

Figure S28. ^{1}H-^{13}C HSQC NMR spectrum of **3** in CD_2Cl_2 (298K).

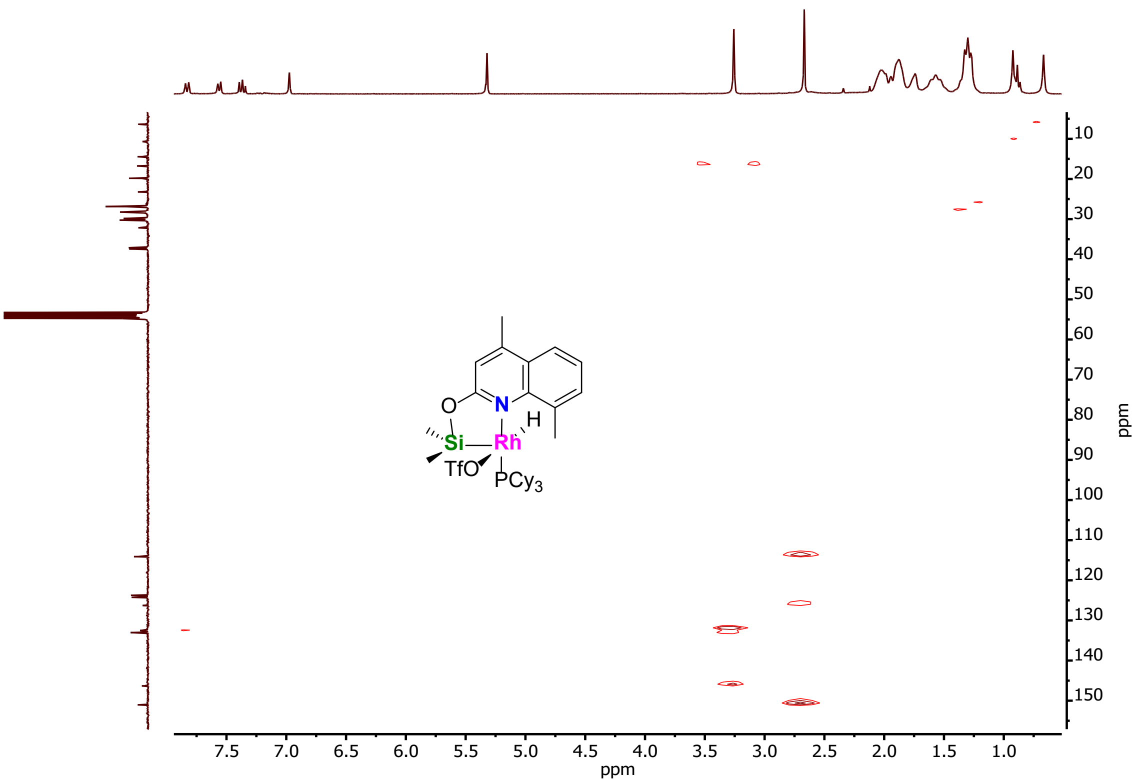

Figure S29. ^{1}H-^{13}C HMBC NMR spectrum of **3** in CD_2Cl_2 (298K).

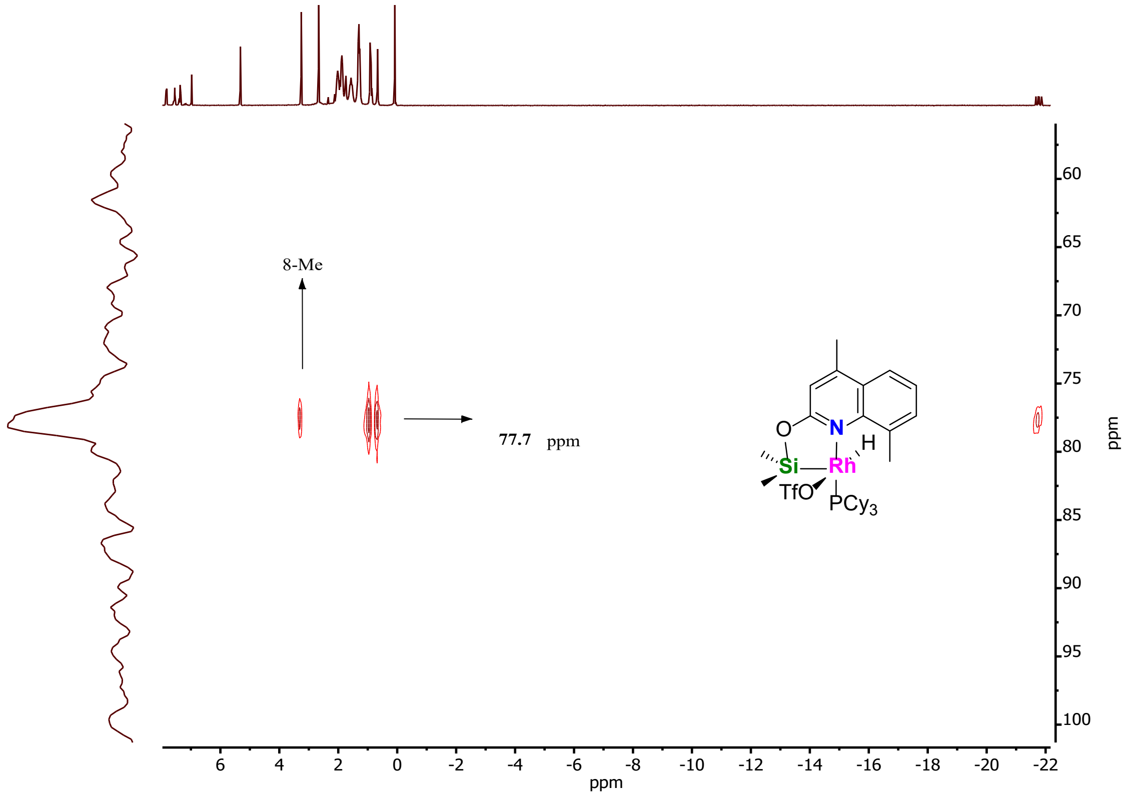

Figure S30. ^{1}H-^{29}Si HMQC NMR spectrum of **3** in CD_2Cl_2 (298K).

10.2. ^{13}C NMR spectra of **2** and **3**

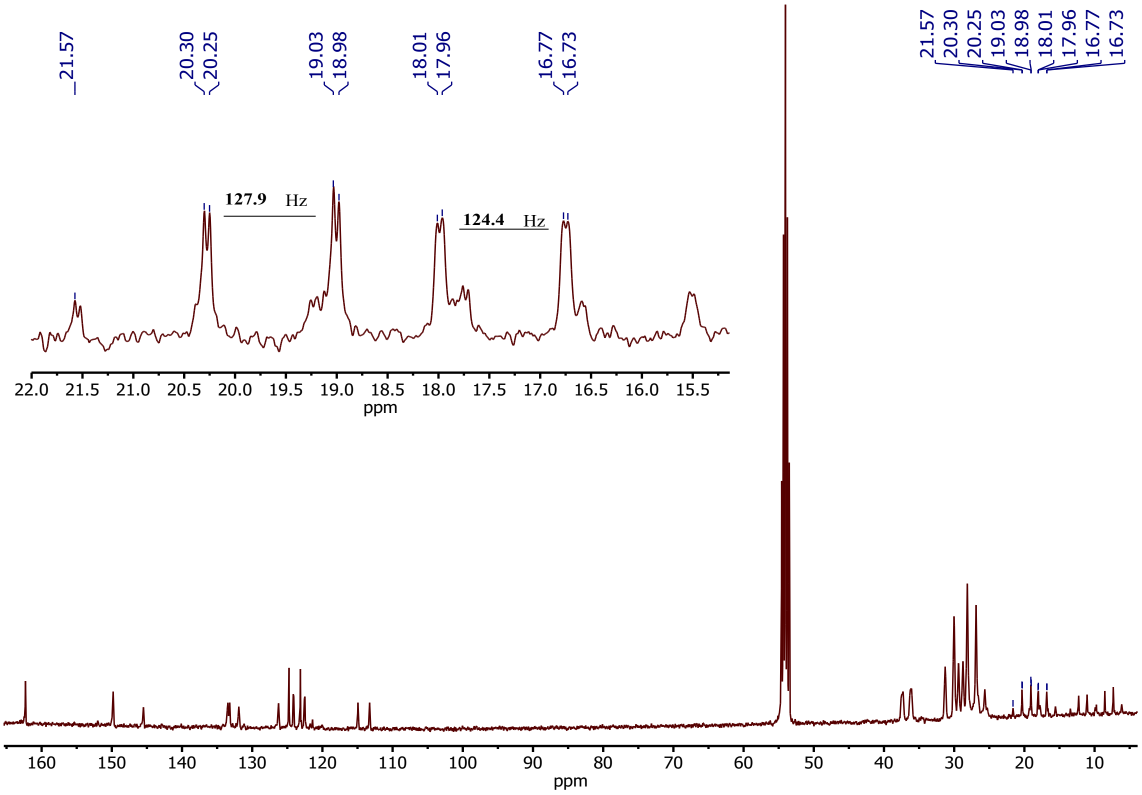

Figure S31. ^{13}C NMR spectrum of **2** in CD_2Cl_2 (101 MHz, 273K).

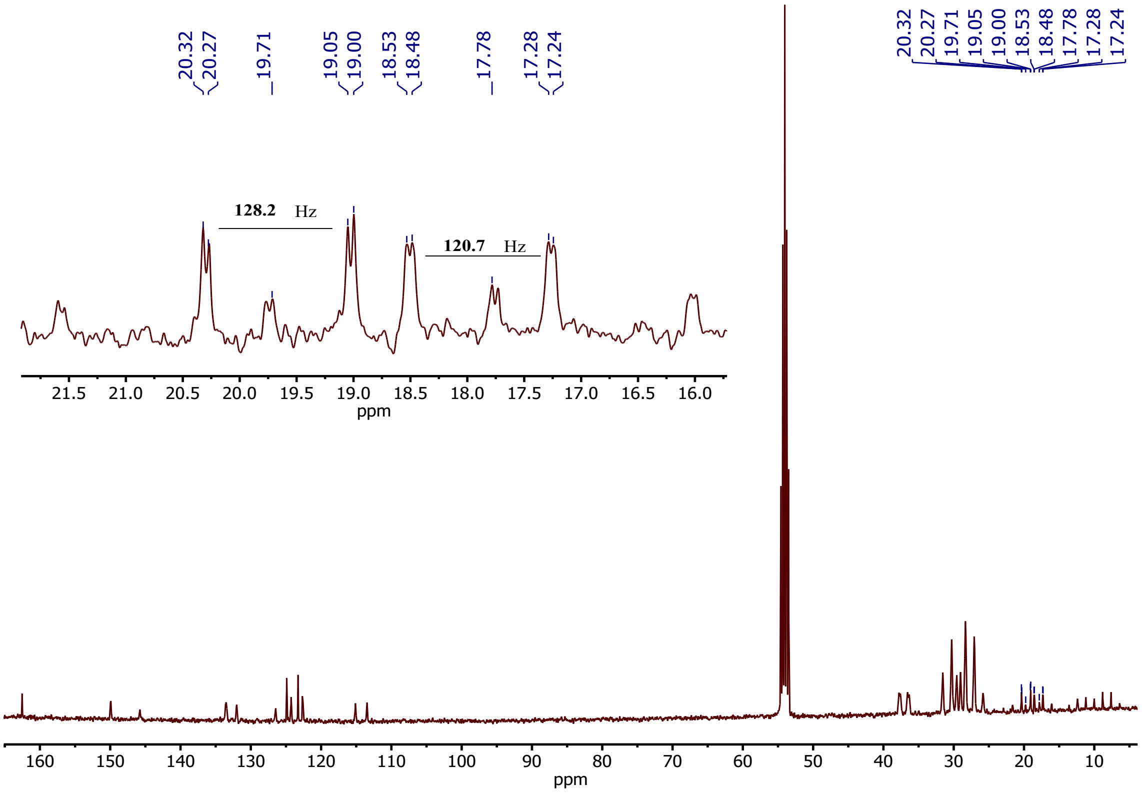

Figure S32. ^{13}C NMR spectrum of **3** in CD_2Cl_2 (101 MHz, 273K).

10.3. NMR spectra of reaction conditions optimization

NMR spectra of screening of catalyst precursor

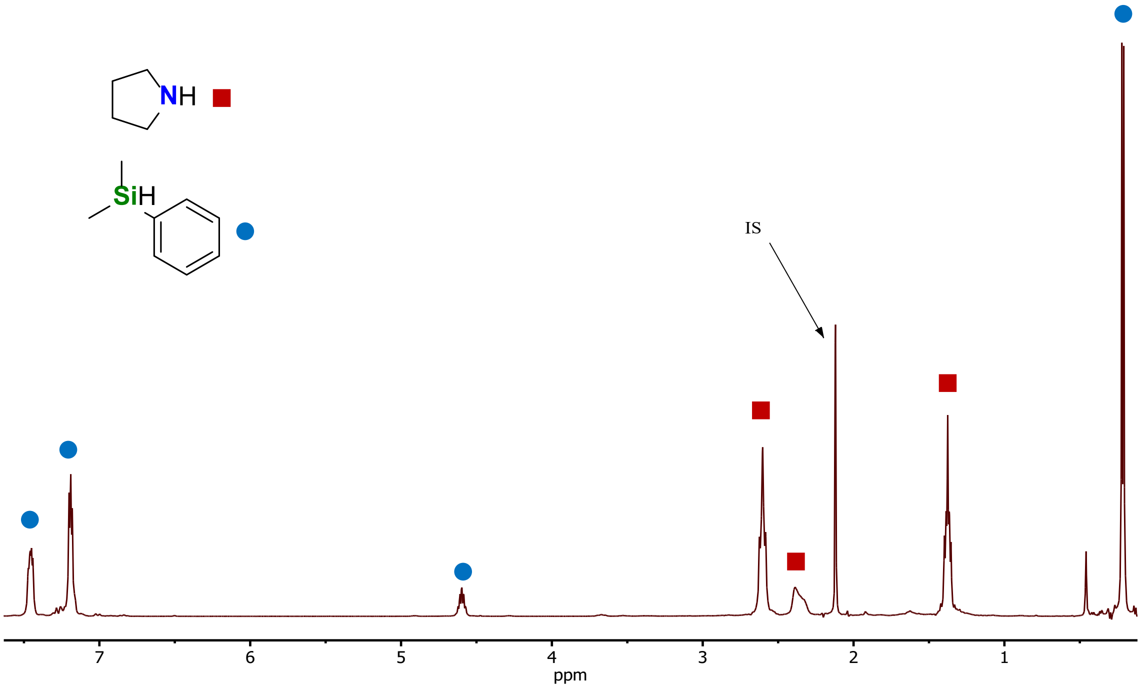

Figure S33. ^{1}H NMR spectrum of the **2**-catalyzed reaction of pyrrolidine with $HSiMe_2Ph$ in C_6D_6 after 3 h at r.t. using hexamethylbenzene as IS.

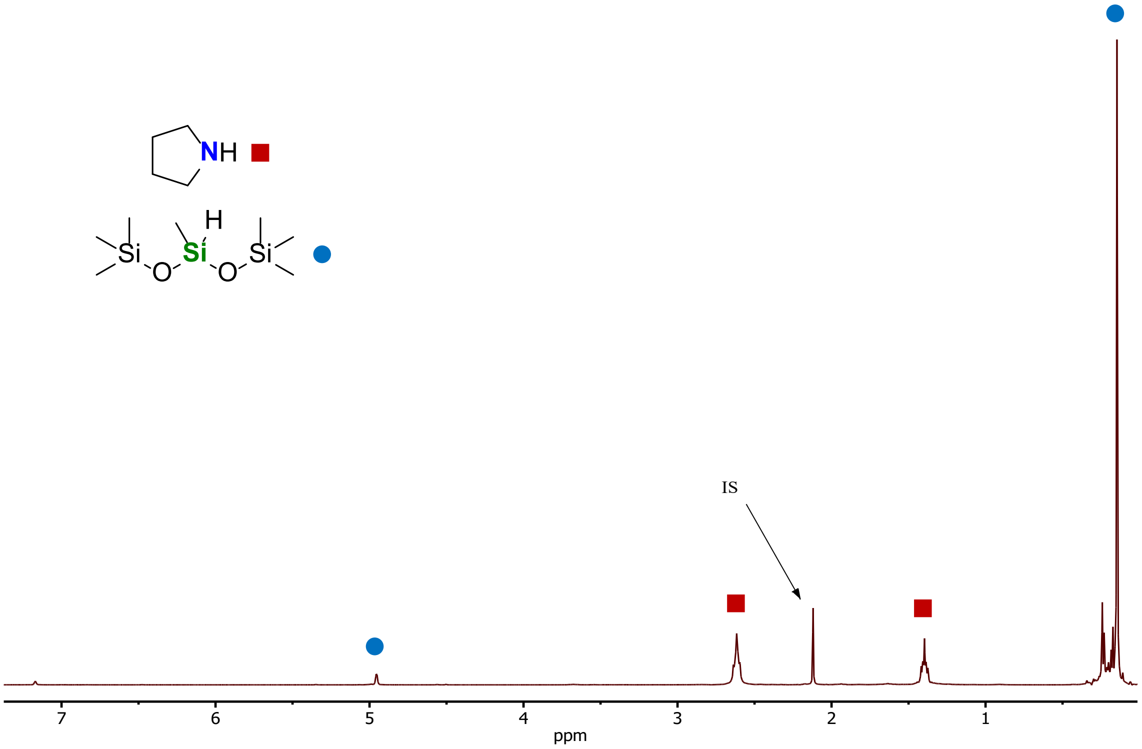

Figure S34. ^{1}H NMR spectrum of the **2**-catalyzed reaction of pyrrolidine with $HSiMe(OSiMe_3)_2$ in C_6D_6 after 3 h at r.t. using hexamethylbenzene as IS.

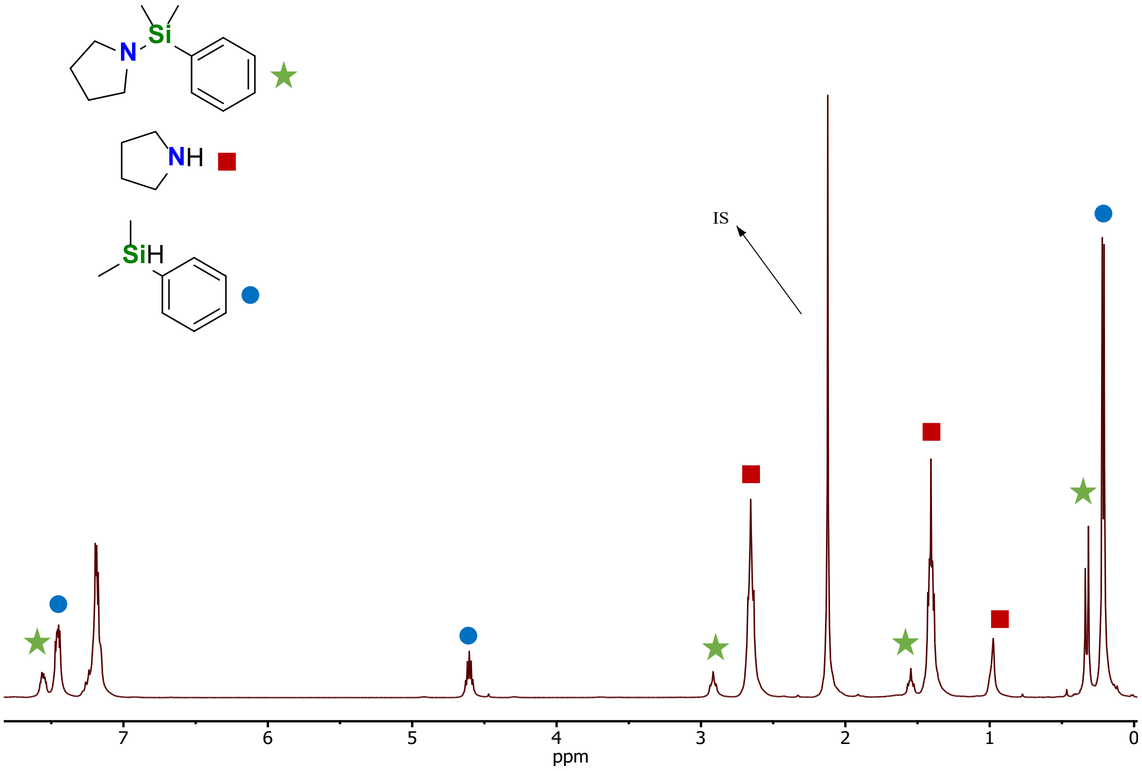

Figure S35. ^{1}H NMR spectrum of the **3**-catalyzed reaction of pyrrolidine with $HSiMe_2Ph$ in C_6D_6 after 3 h at r.t. using hexamethylbenzene as IS.

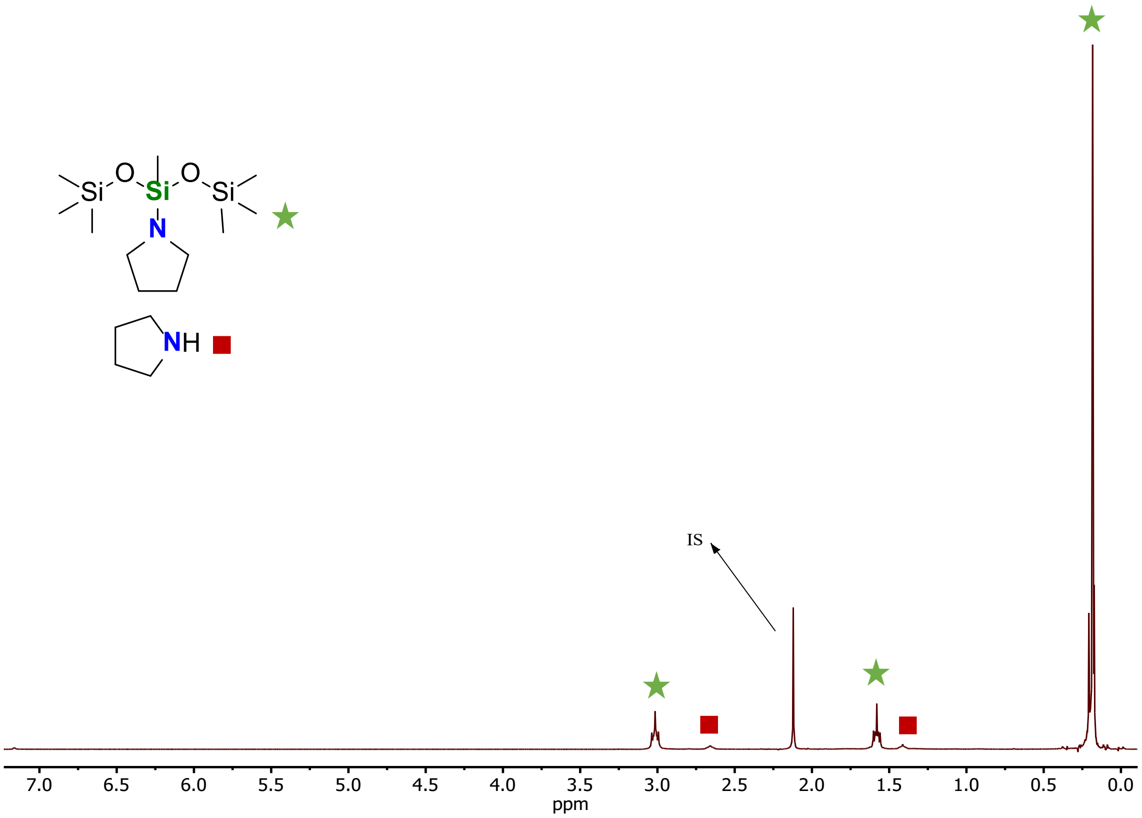

Figure S36. ^{1}H NMR spectrum of the **3**-catalyzed reaction of pyrrolidine with $HSiMe(OSiMe_3)_2$ in C_6D_6 after 3 h at r.t. using hexamethylbenzene as IS.

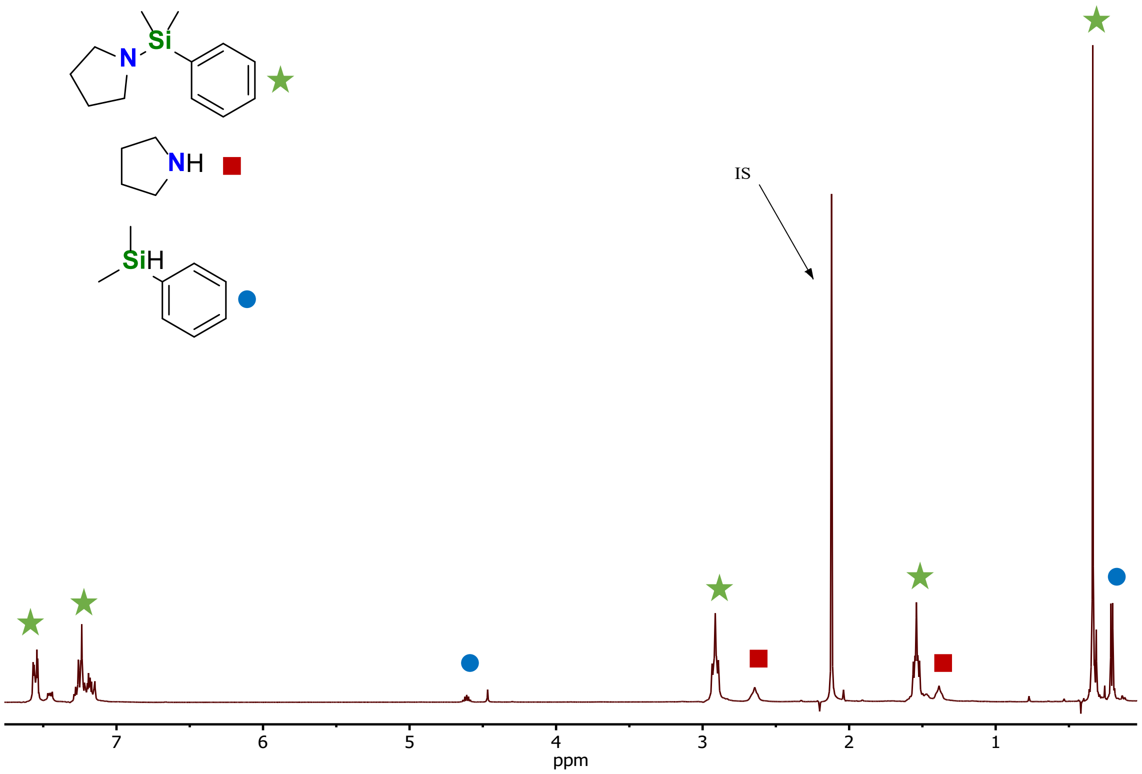

Figure S37. ^{1}H NMR spectrum of the **4**-catalyzed reaction of pyrrolidine with $HSiMe_2Ph$ in C_6D_6 after 3 h at r.t. using hexamethylbenzene as IS.

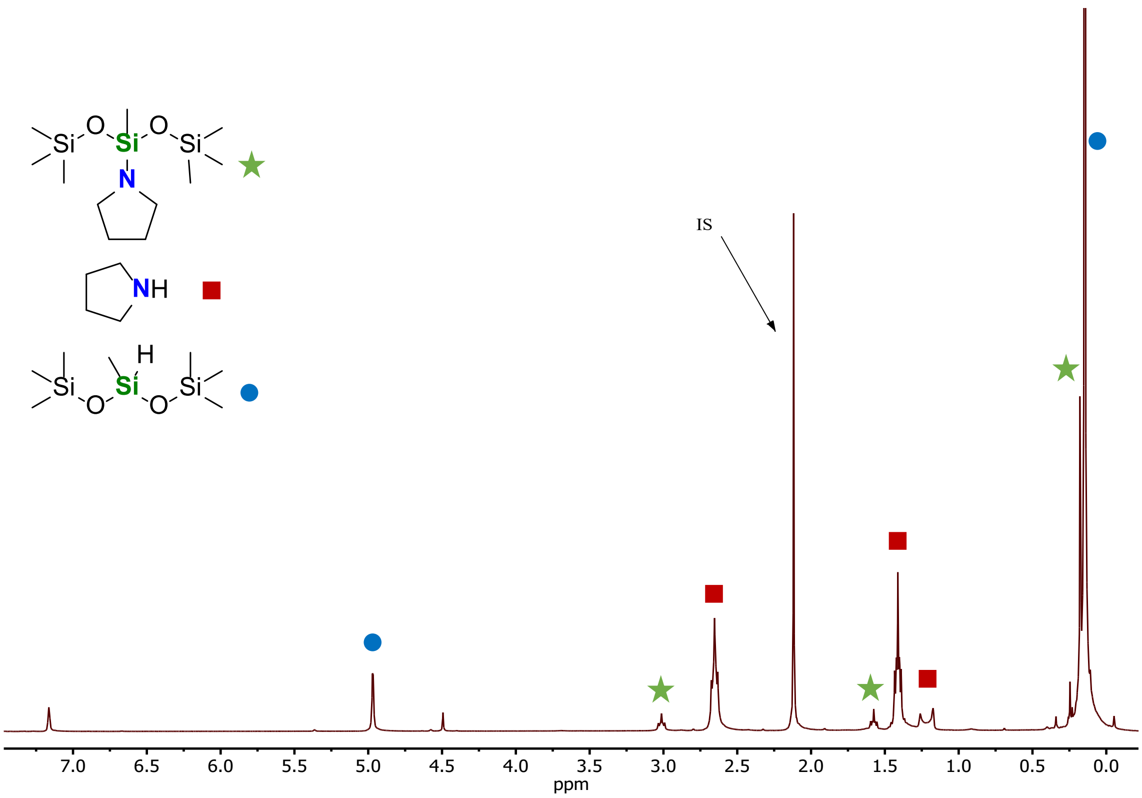

Figure S38. ^{1}H NMR spectrum of the **4**-catalyzed reaction of pyrrolidine with $HSiMe(OSiMe_3)_2$ in C_6D_6 after 3 h at r.t. using hexamethylbenzene as IS.

NMR of screening of hydrosilanes

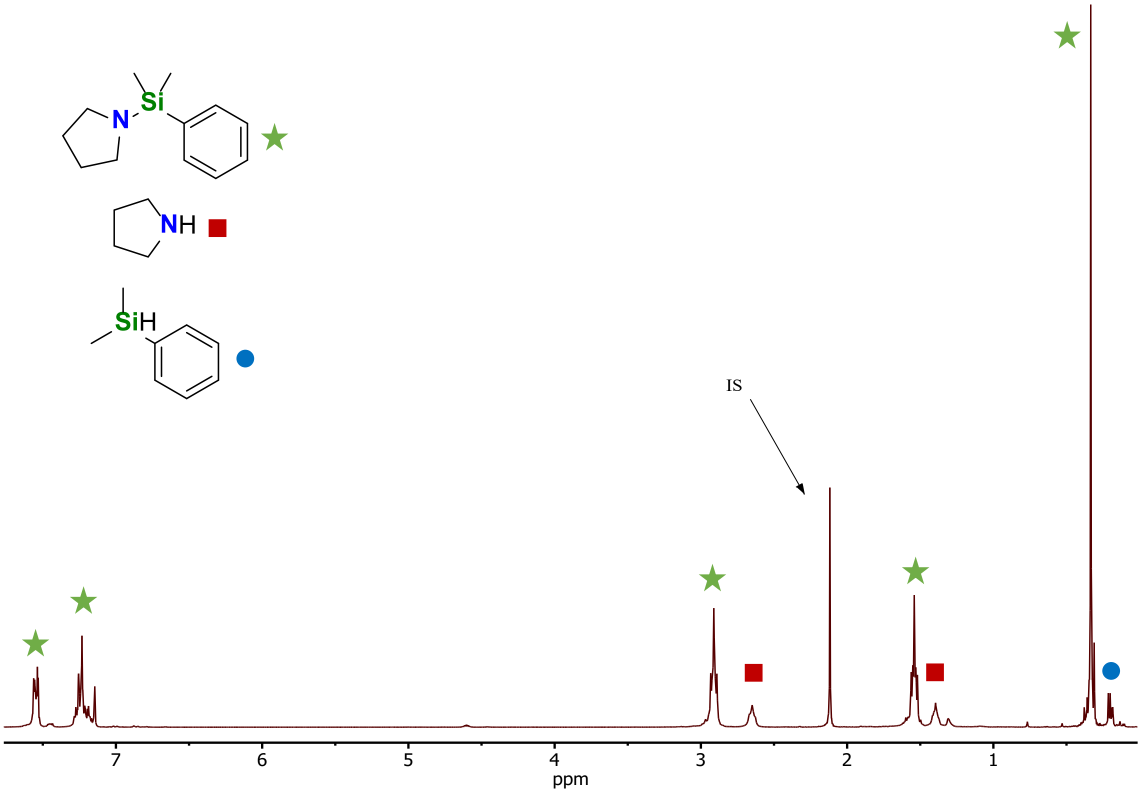

Figure S39. ^{1}H NMR spectrum of the **3**-catalyzed reaction of pyrrolidine with $HSiMe_2Ph$ in C_6D_6 after 3 h at 333 K using hexamethylbenzene as IS.

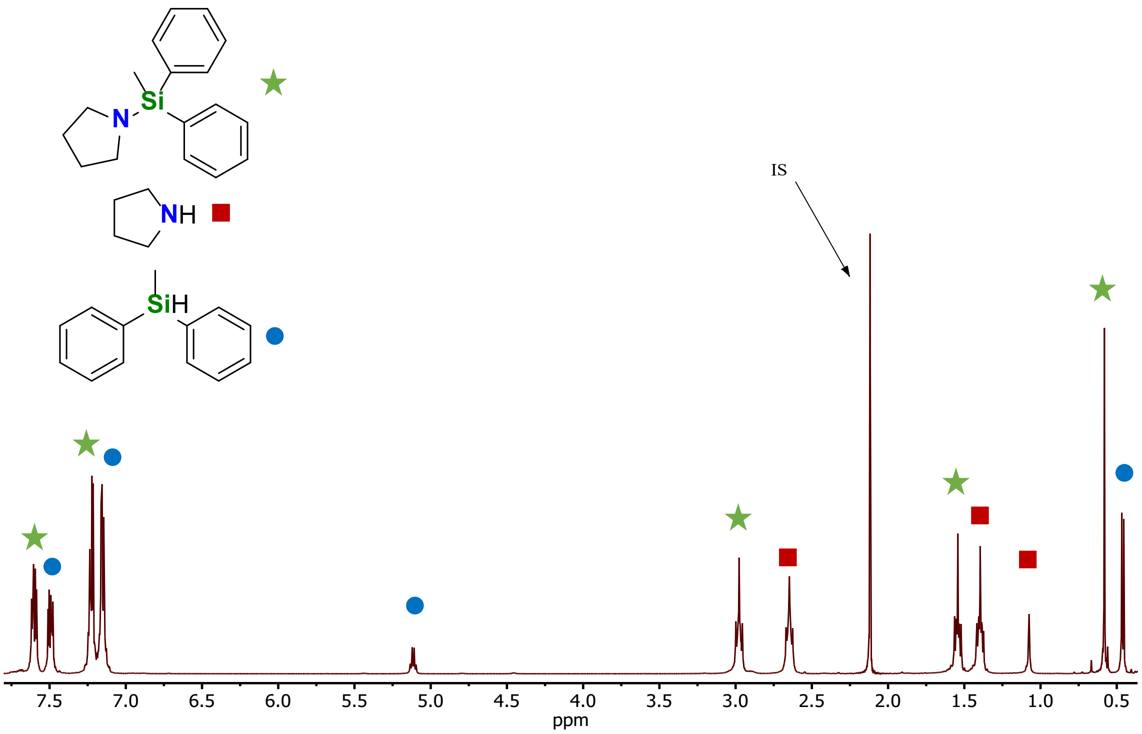

Figure S40. ^{1}H NMR spectrum of the **3**-catalyzed reaction of pyrrolidine with $HSiMePh_2$ in C_6D_6 after 3 h at r.t. using hexamethylbenzene as IS.

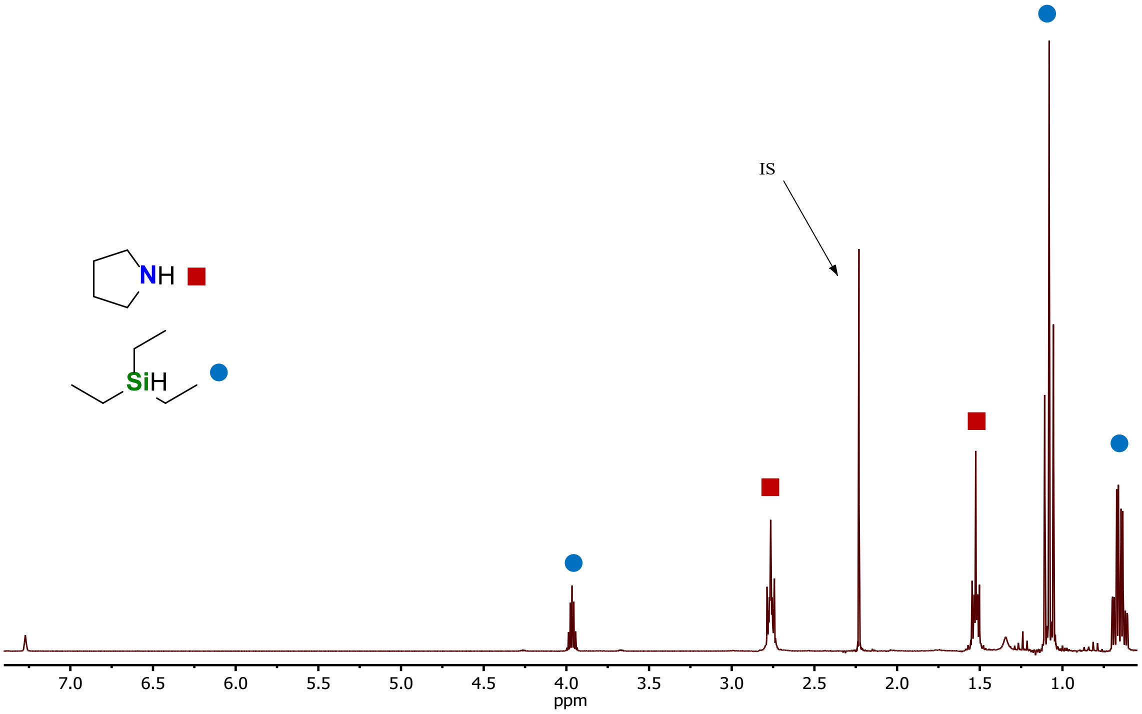

Figure S41. ^{1}H NMR spectrum of the **3**-catalyzed reaction of pyrrolidine with $HSiEt_3$ in C_6D_6 after 3 h at r.t. using hexamethylbenzene as IS.

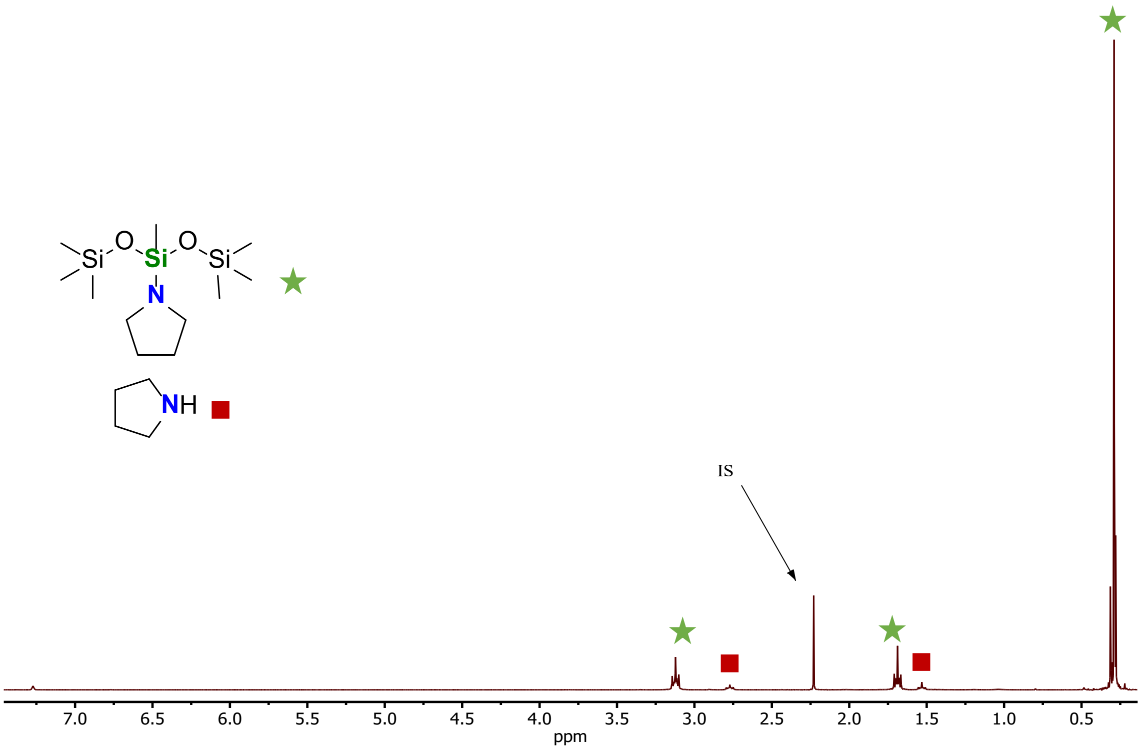

Figure S42. ^{1}H NMR spectrum of the **3**-catalyzed reaction of pyrrolidine with $HSiMe(OSiMe_3)_2$ in C_6D_6 after 3 h at r.t. using hexamethylbenzene as IS.

10.4. NMR spectra of siloxazanes

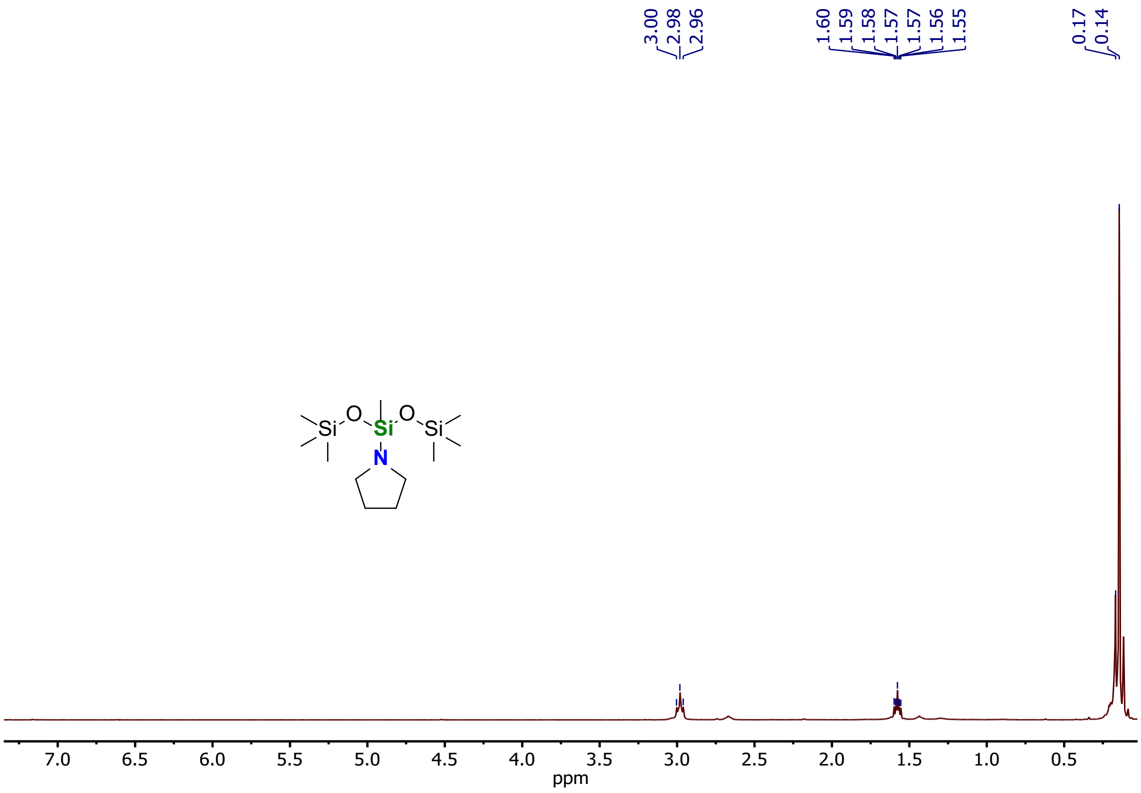

Figure S43. ^{1}H NMR spectrum of **8a** in C_6D_6 (300 MHz, 298K).

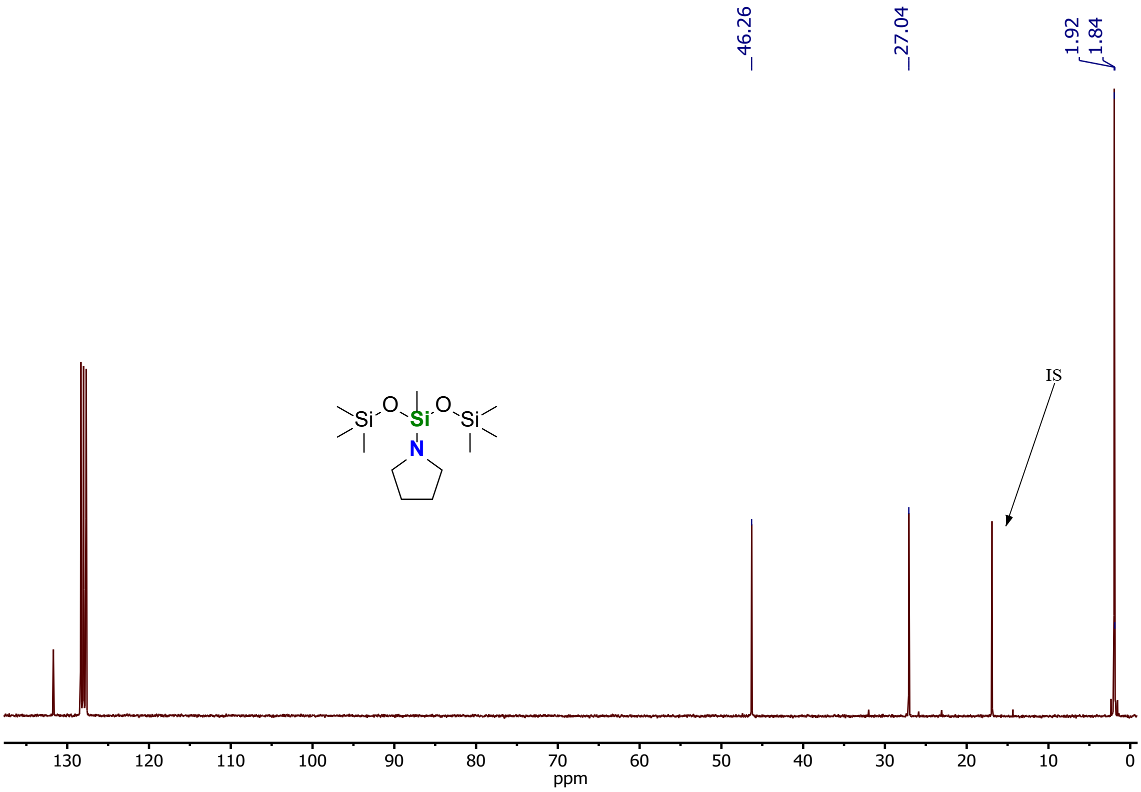

Figure S44. $^{13}C\{^{1}H\}$ NMR spectrum of **8a** in C_6D_6 (75 MHz, 298K).

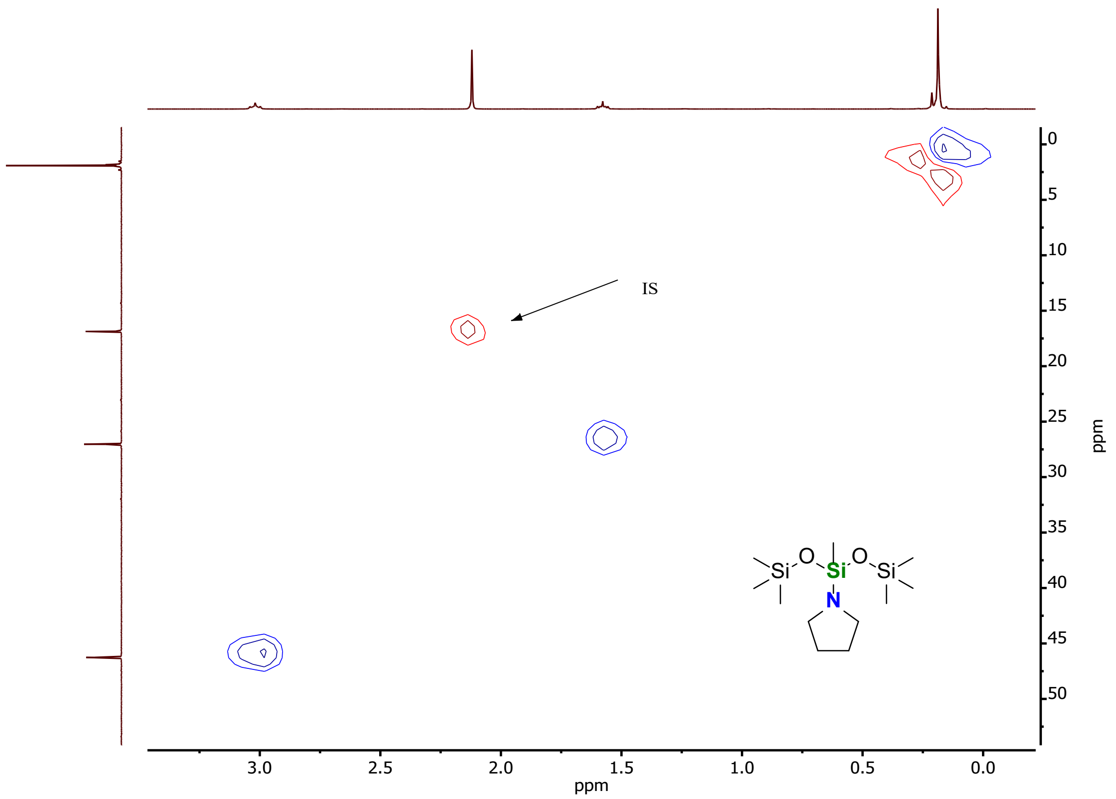

Figure S45. ^{1}H-^{13}C HSQC NMR spectrum of **8a** in C_6D_6 (298K).

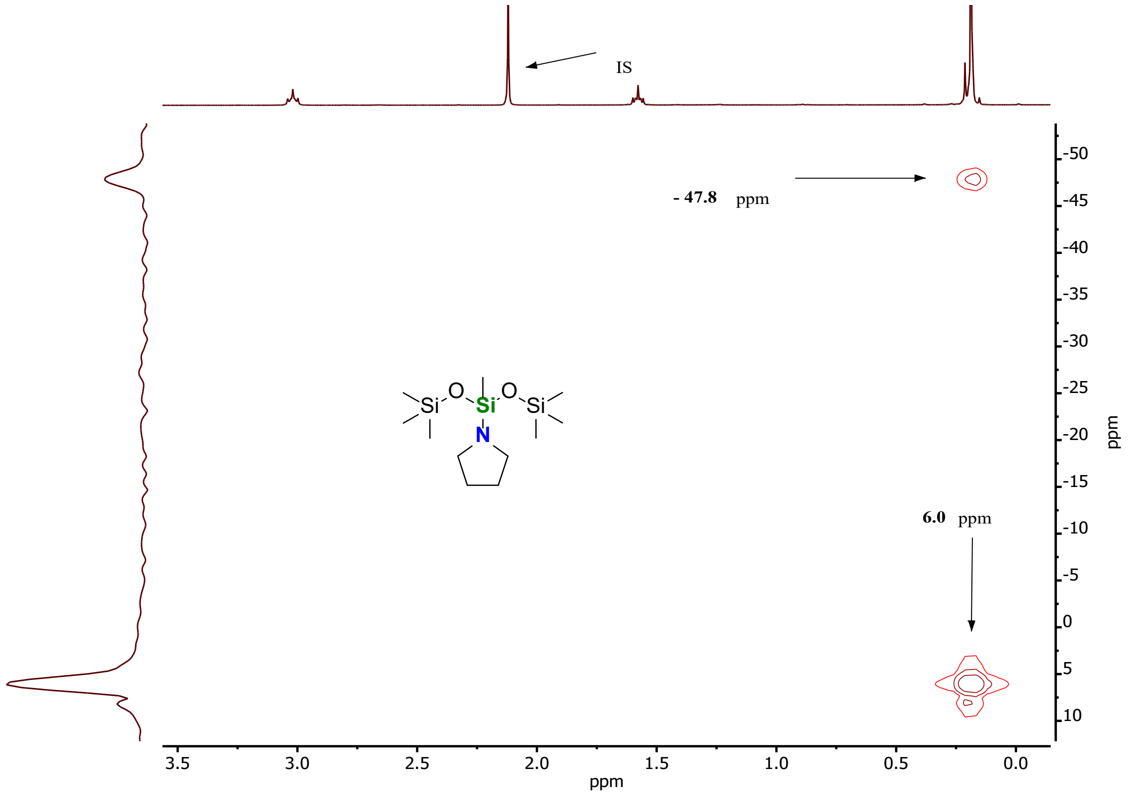

Figure S46. ^{1}H-^{29}Si HMQC NMR spectrum of **8a** in C_6D_6 (298K).

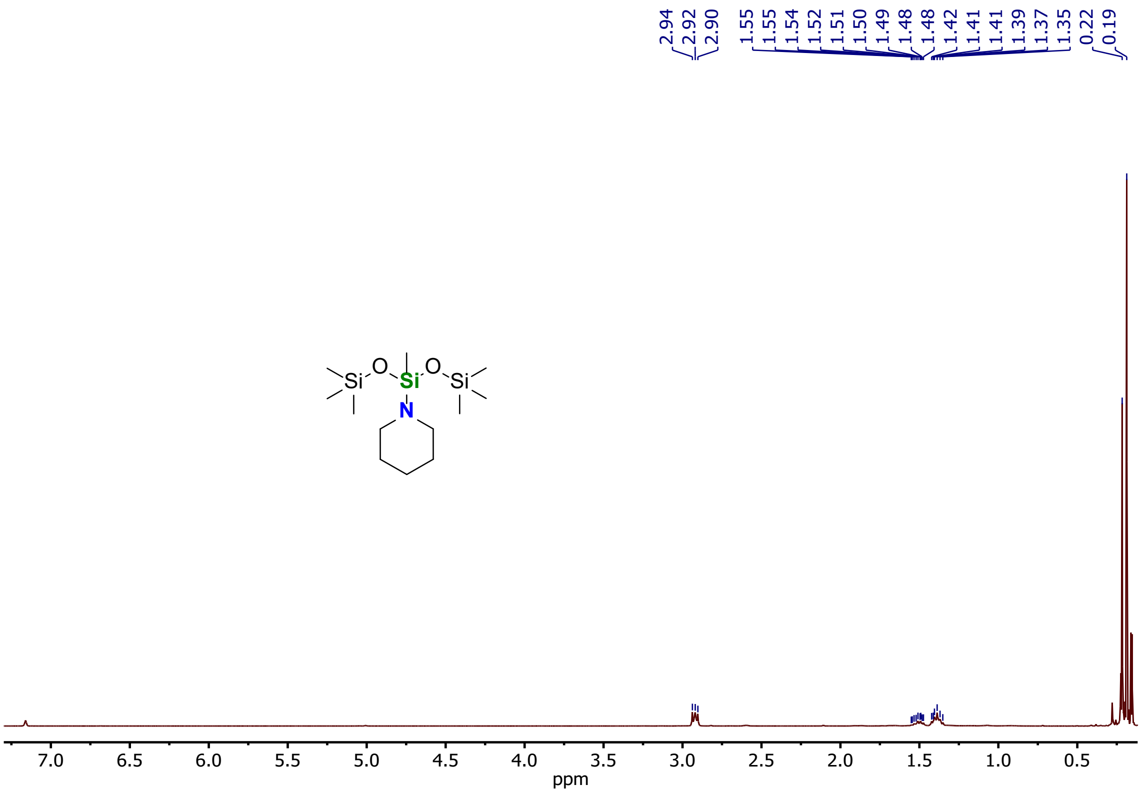

Figure S47. ^{1}H NMR spectrum of **8b** in C_6D_6 (300 MHz, 298K).

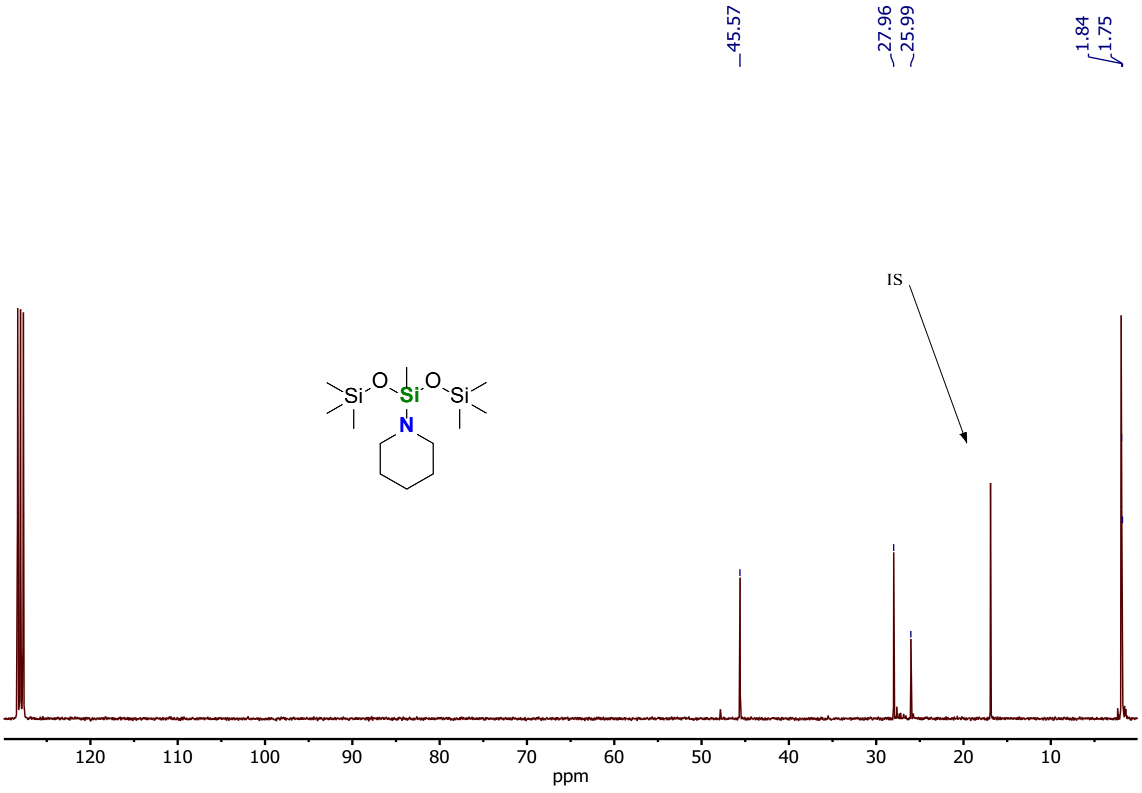

Figure S48. $^{13}C\{^{1}H\}$ NMR spectrum of **8b** in C_6D_6 (75 MHz, 298K).

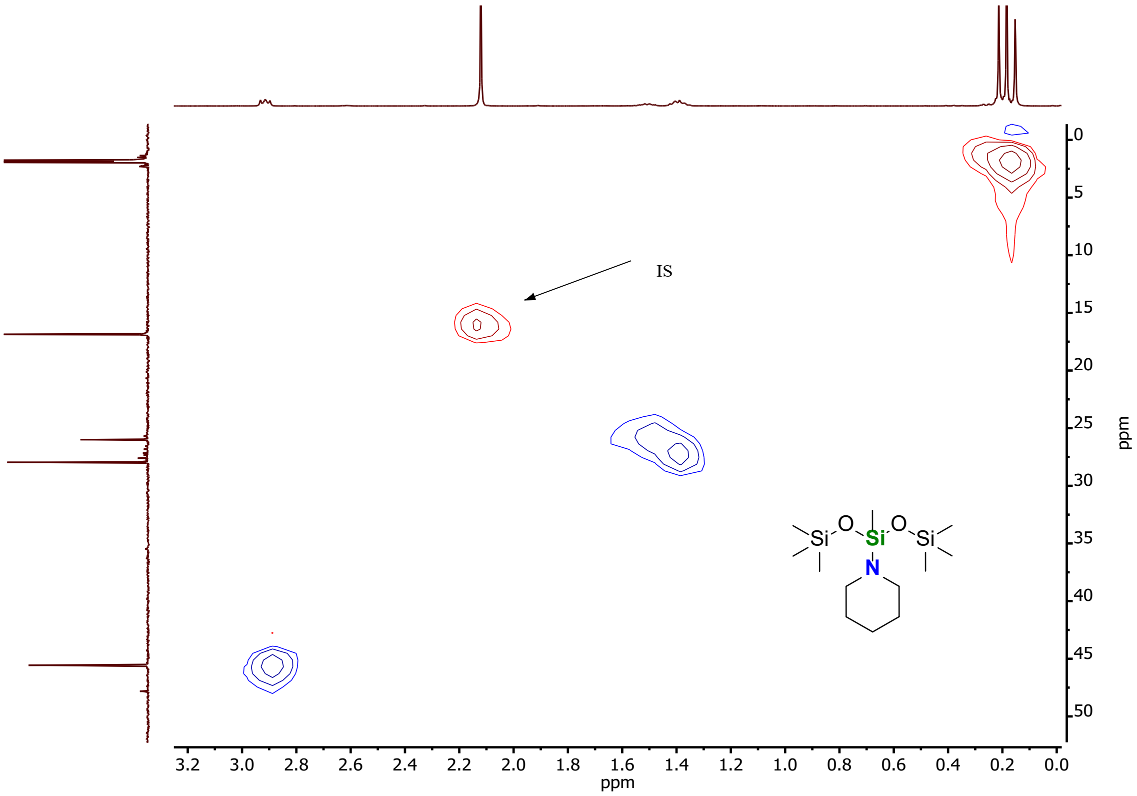

Figure S49. ^1H-^{13}C HSQC NMR spectrum of **8b** in C_6D_6 (298K).

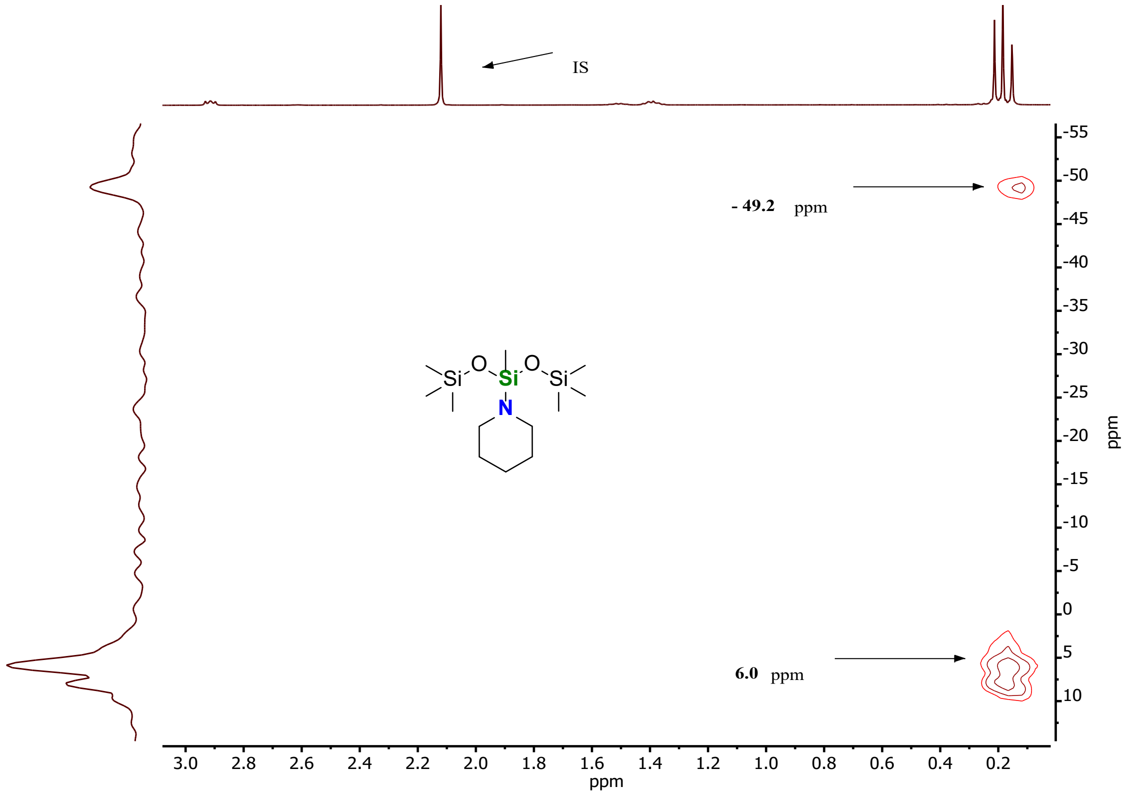

Figure S50. ^1H-^{29}Si HMQC NMR spectrum of **8b** in C_6D_6 (298K).

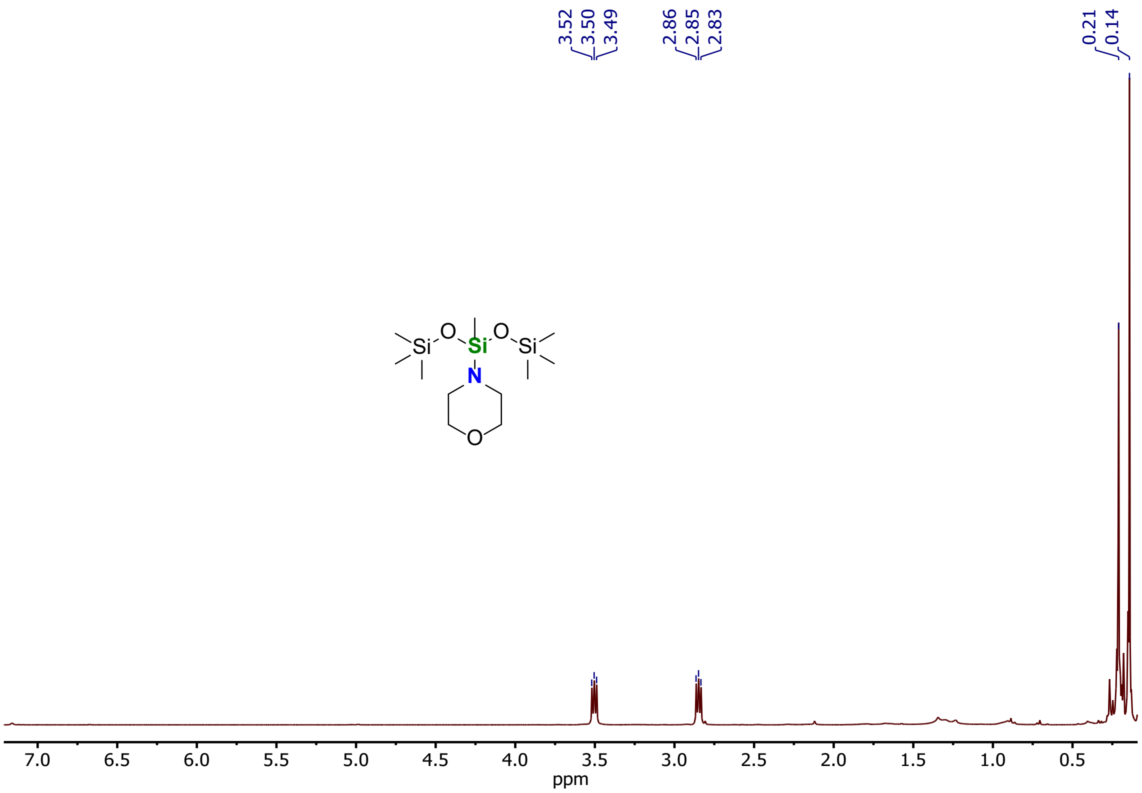

Figure S51. ^{1}H NMR spectrum of **8c** in C_6D_6 (300 MHz, 298K).

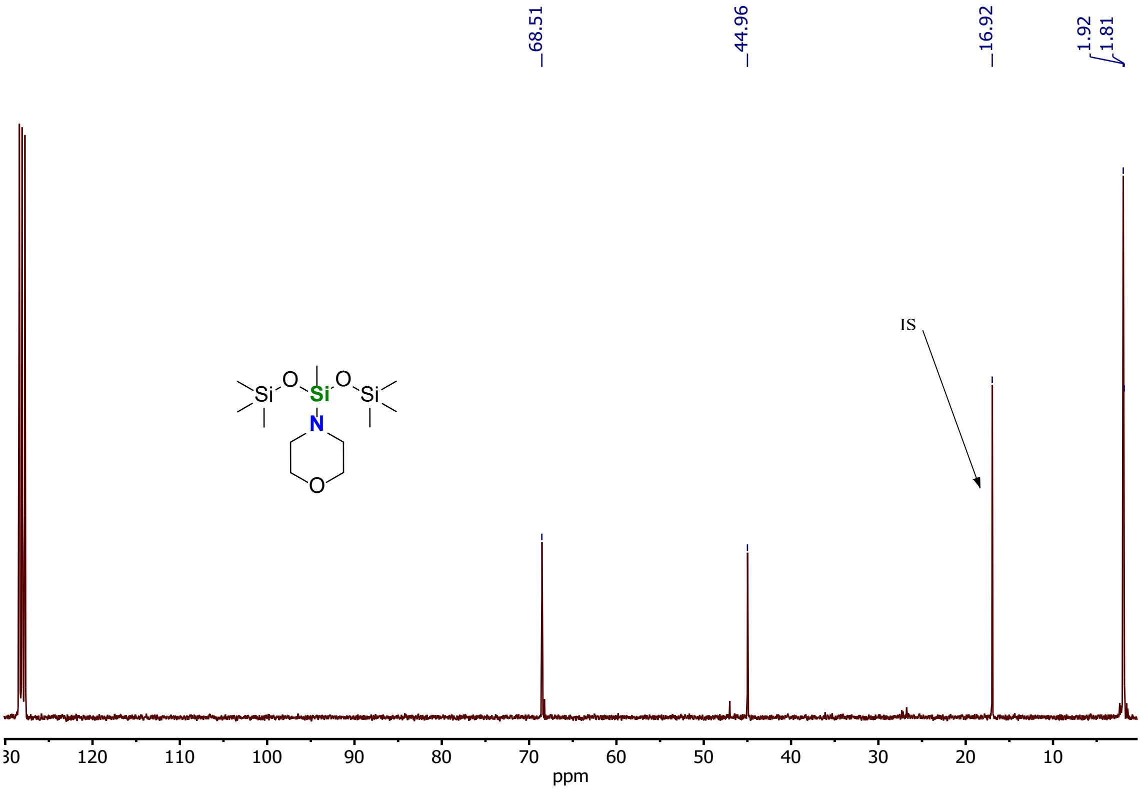

Figure S52. $^{13}C\{^{1}H\}$ NMR spectrum of **8c** in C_6D_6 (75 MHz, 298K).

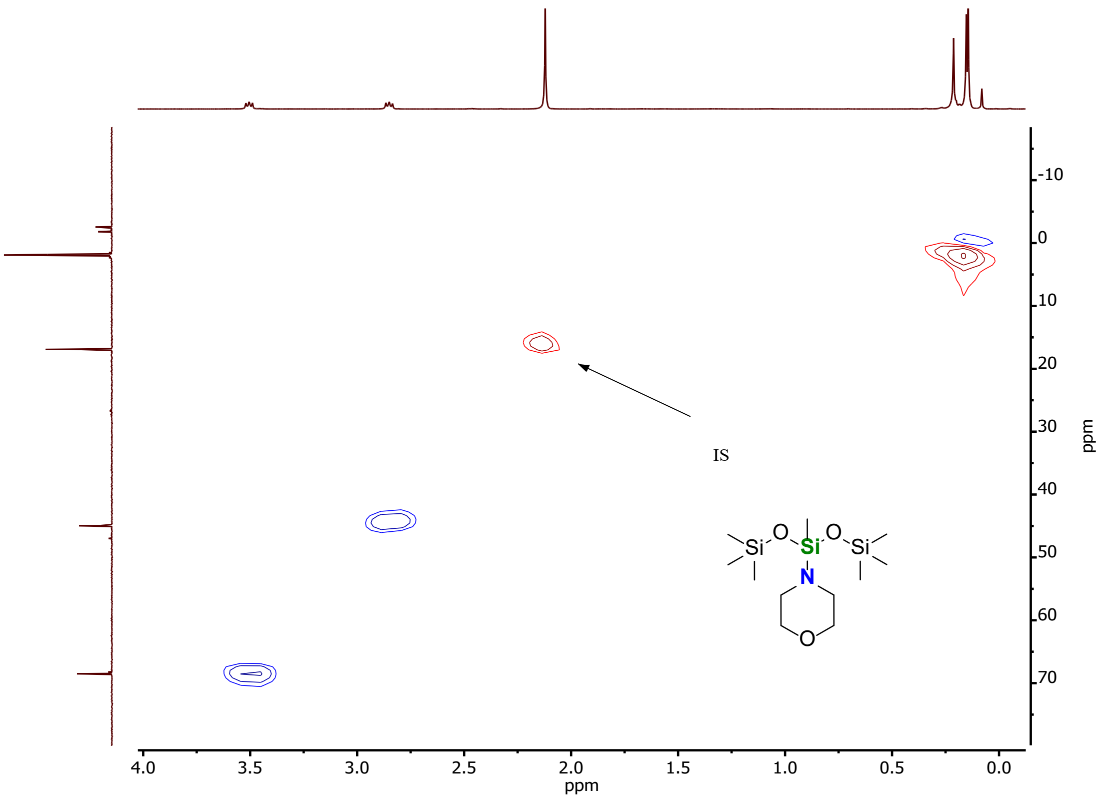

Figure S53. ^{1}H-^{13}C HSQC NMR spectrum of **8c** in C_6D_6 (298K).

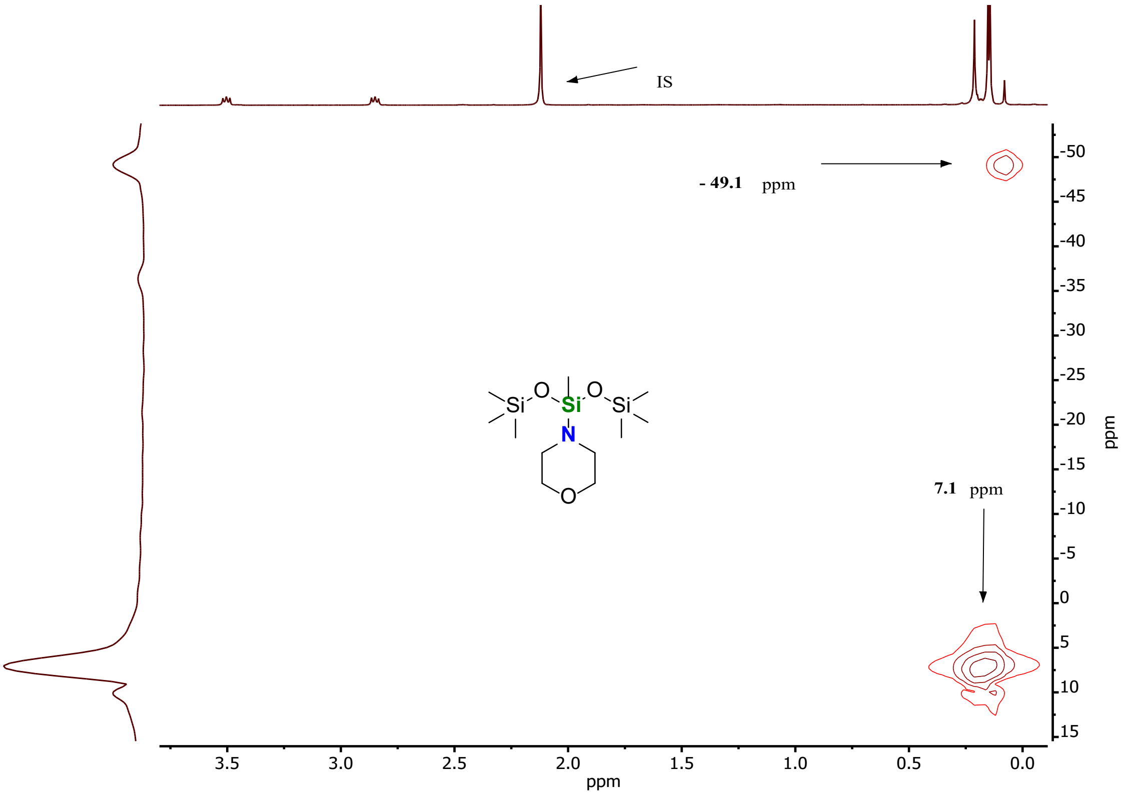

Figure S54. ^{1}H-^{29}Si HMQC NMR spectrum of **8c** in C_6D_6 (298K).

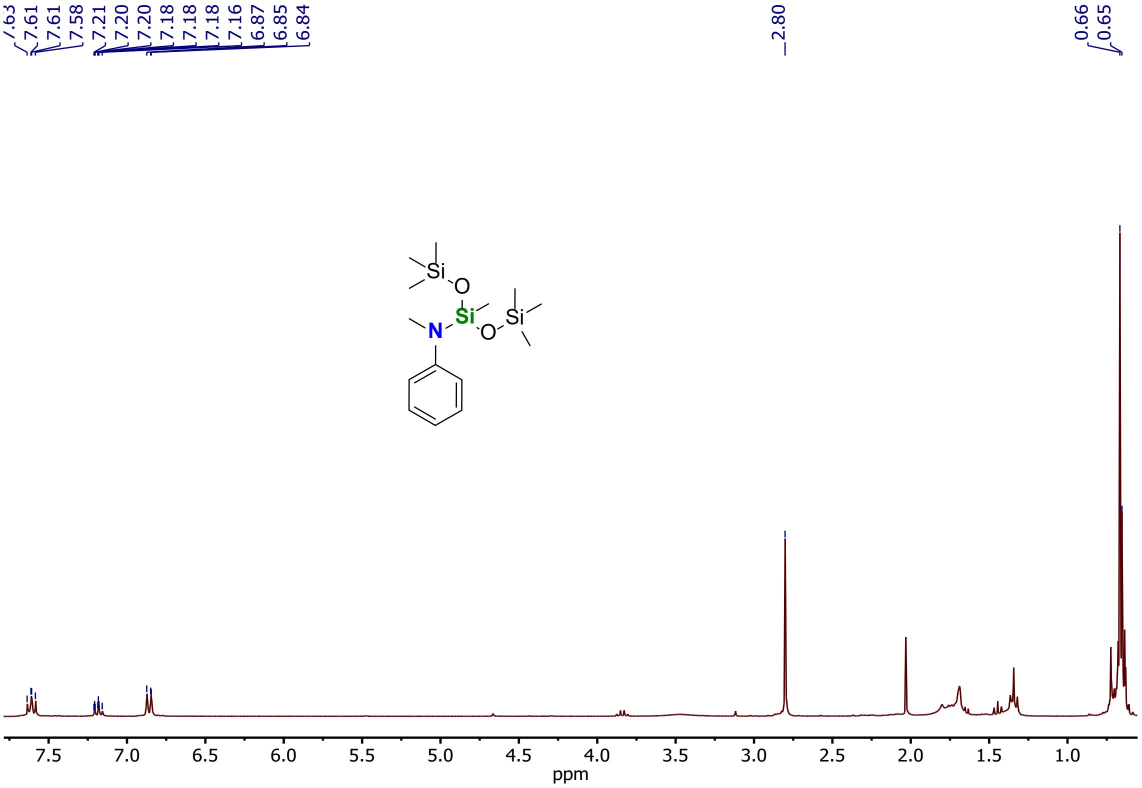

Figure S55. ^{1}H NMR spectrum of **8d** in C_6D_6 (300 MHz, 298K).

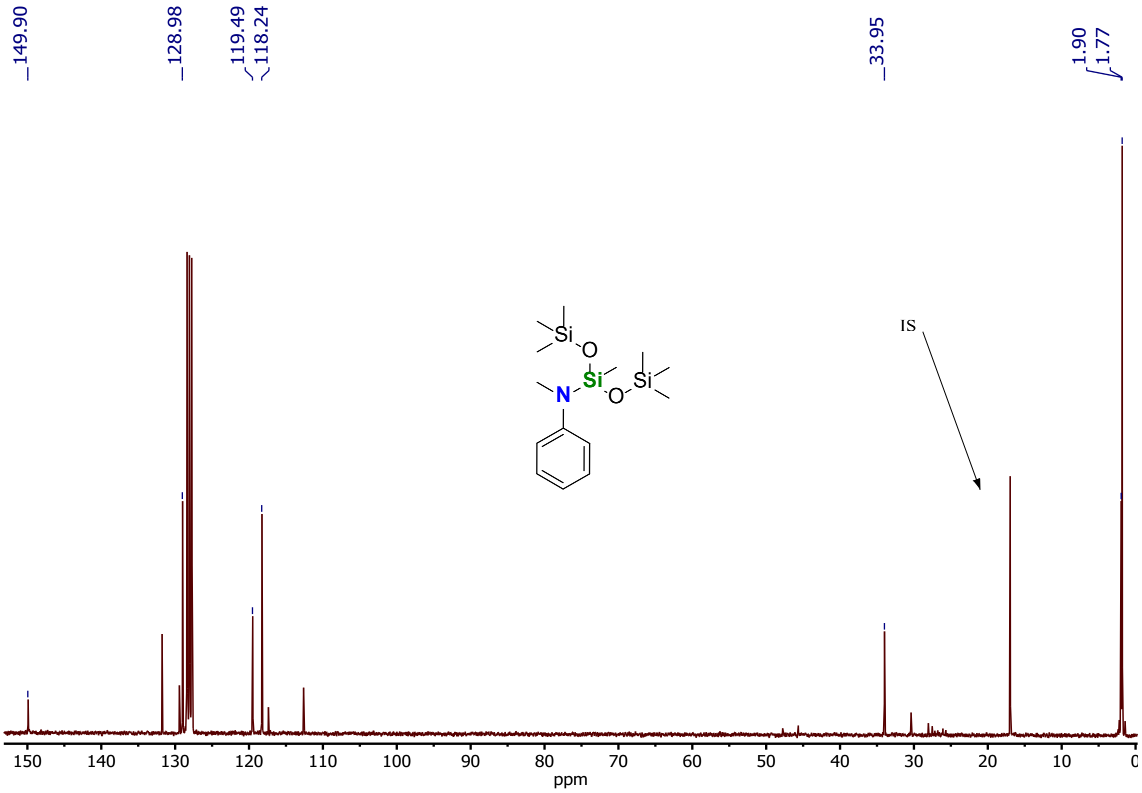

Figure S56. $^{13}C\{^{1}H\}$ NMR spectrum of **8d** in C_6D_6 (75 MHz, 298K).

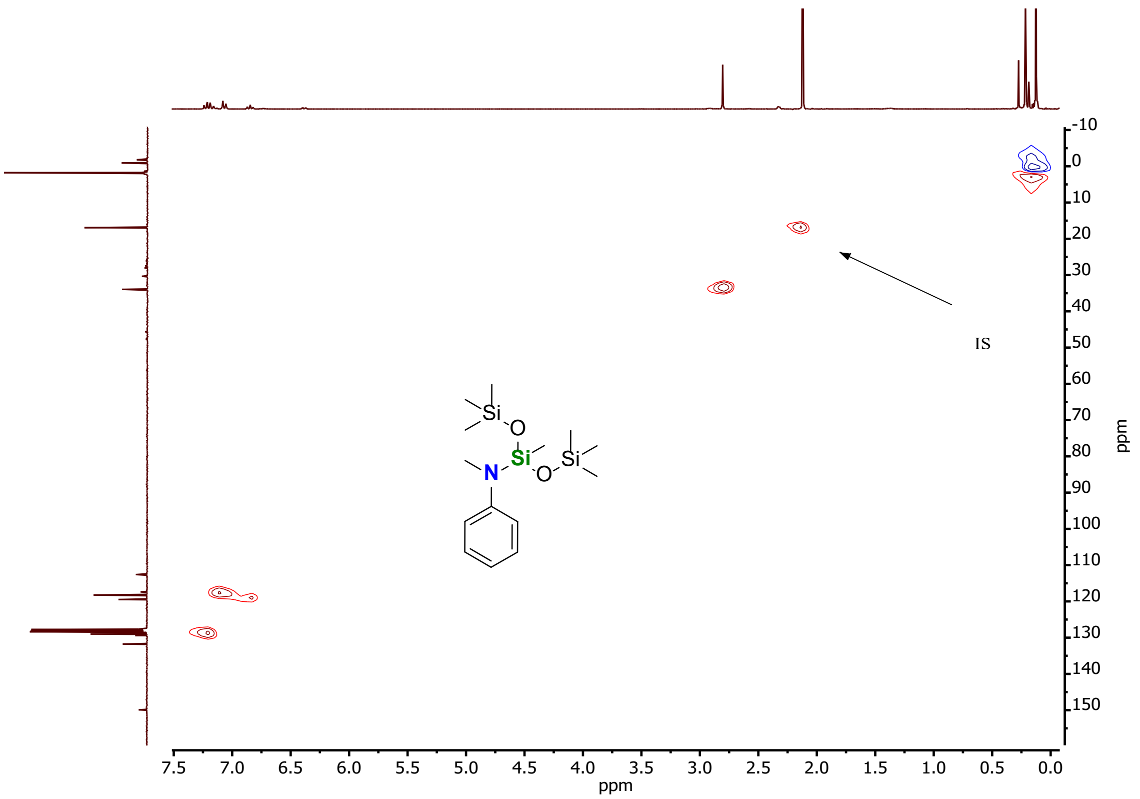

Figure S57. ^{1}H-^{13}C HSQC NMR spectrum of **8d** in C_6D_6 (298K).

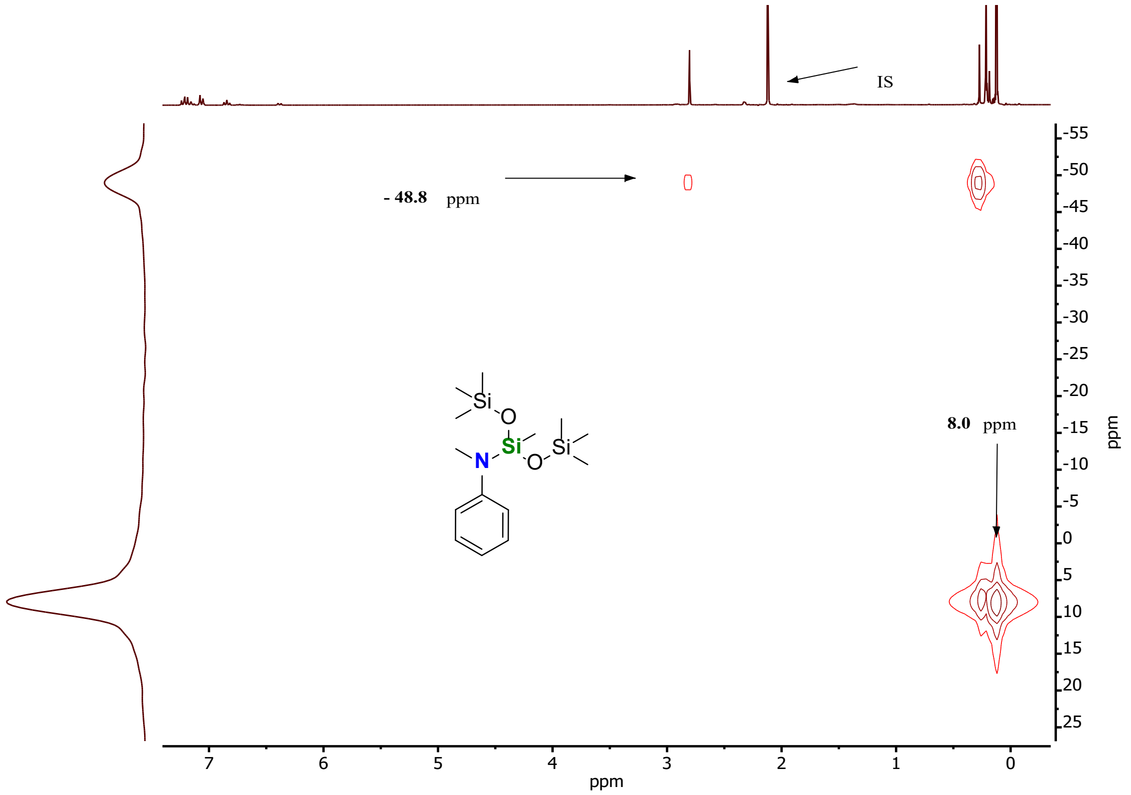

Figure S58. ^{1}H-^{29}Si HMQC NMR spectrum of **8d** in C_6D_6 (298K).

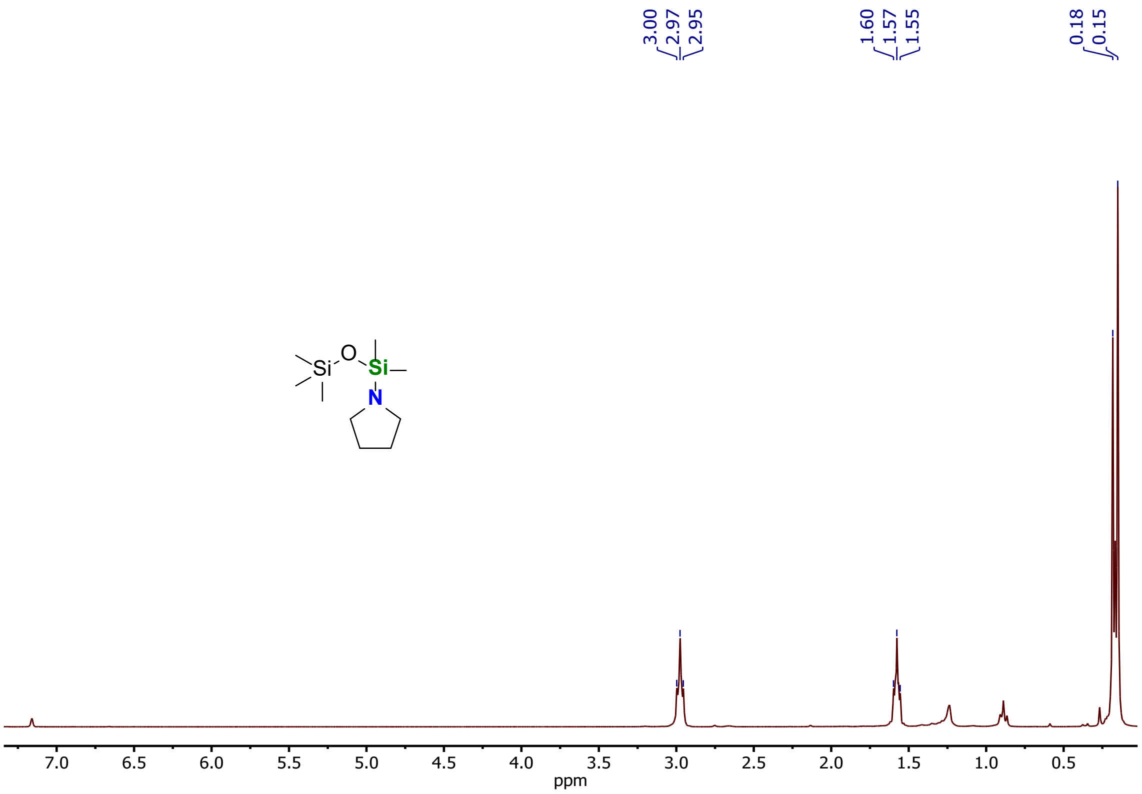

Figure S59. ^{1}H NMR spectrum of **9a** in C_6D_6 (300 MHz, 298K).

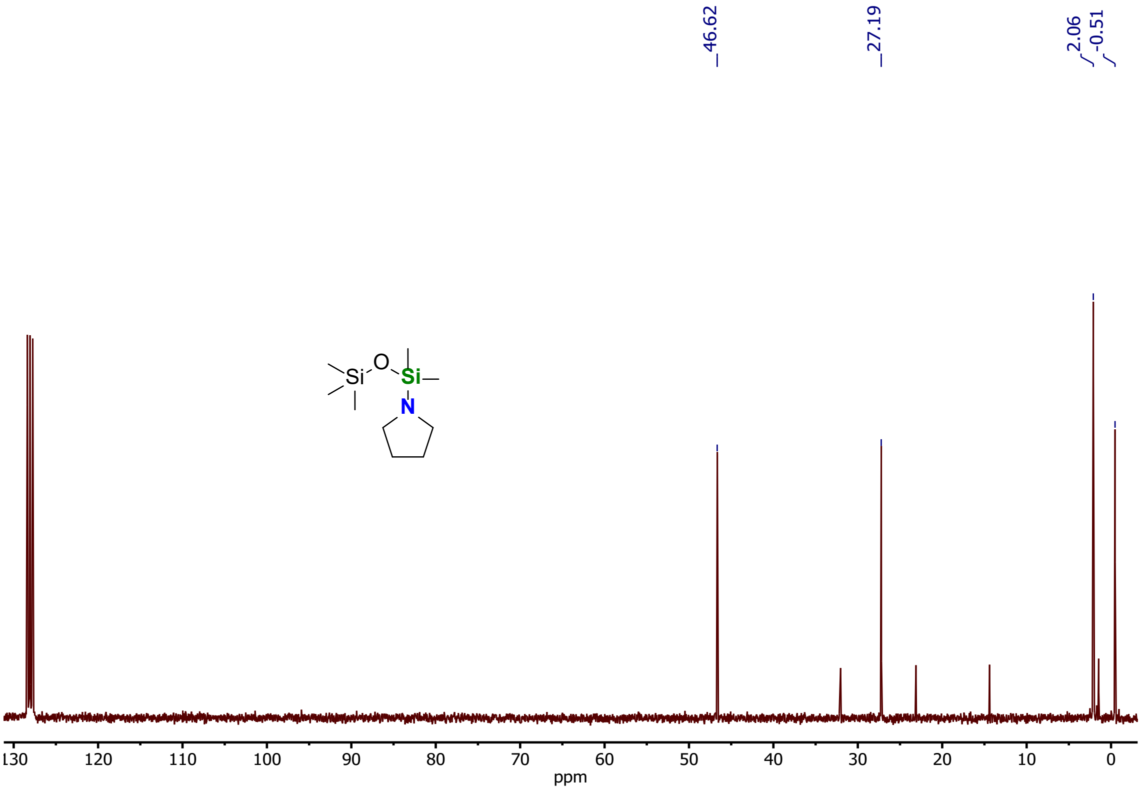

Figure S60. $^{13}C\{^{1}H\}$ NMR spectrum of **9a** in C_6D_6 (75 MHz, 298K).

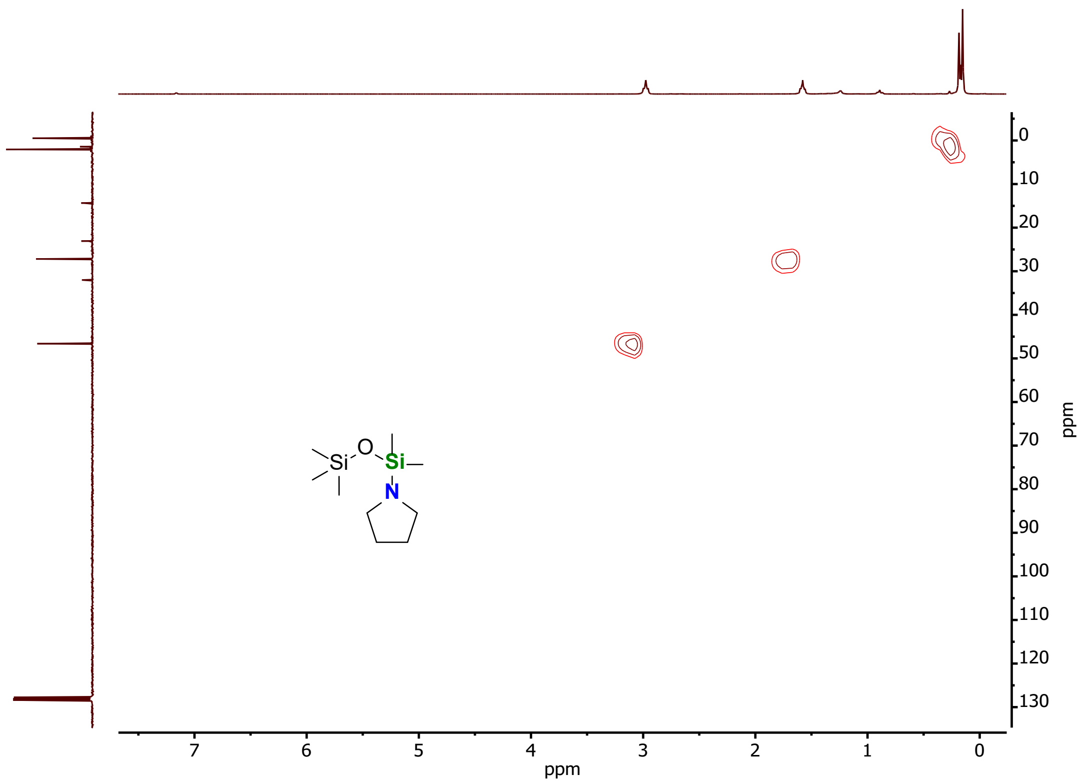

Figure S61. ^{1}H-^{13}C HSQC NMR spectrum of **9a** in C_6D_6 (298K).

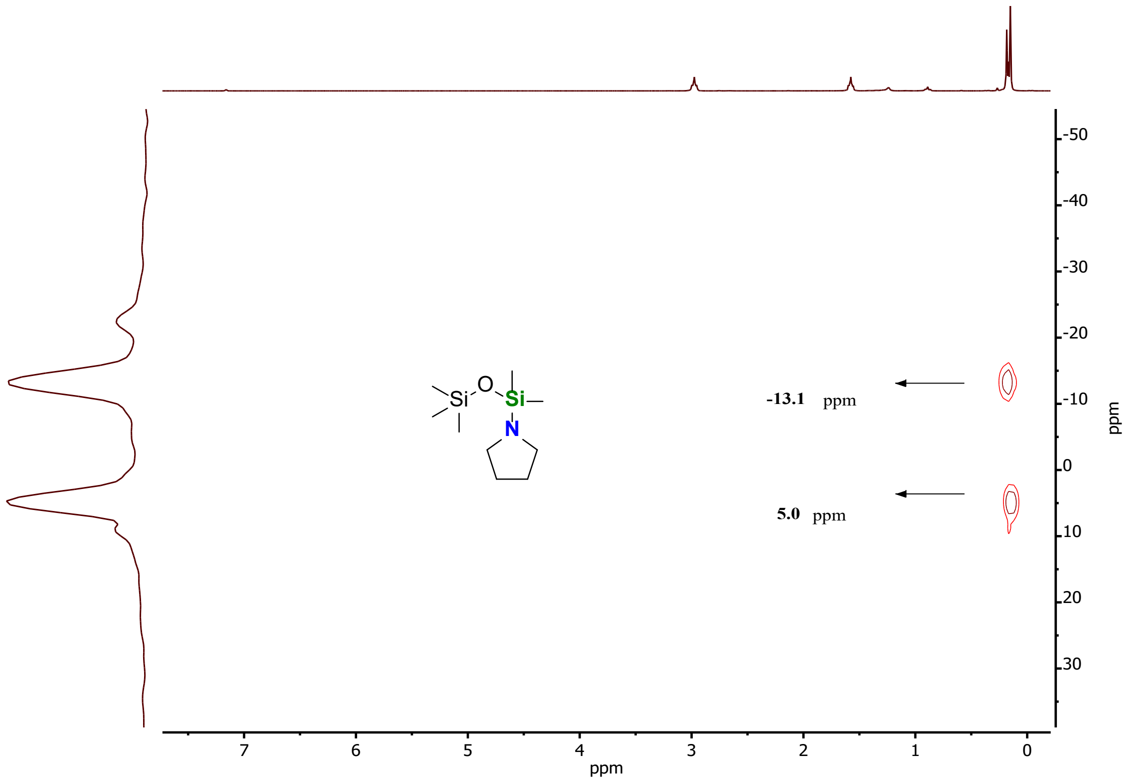

Figure S62. ^{1}H-^{29}Si HMQC NMR spectrum of **9a** in C_6D_6 (298K).

10.5. NMR spectra of miscellaneous reactions

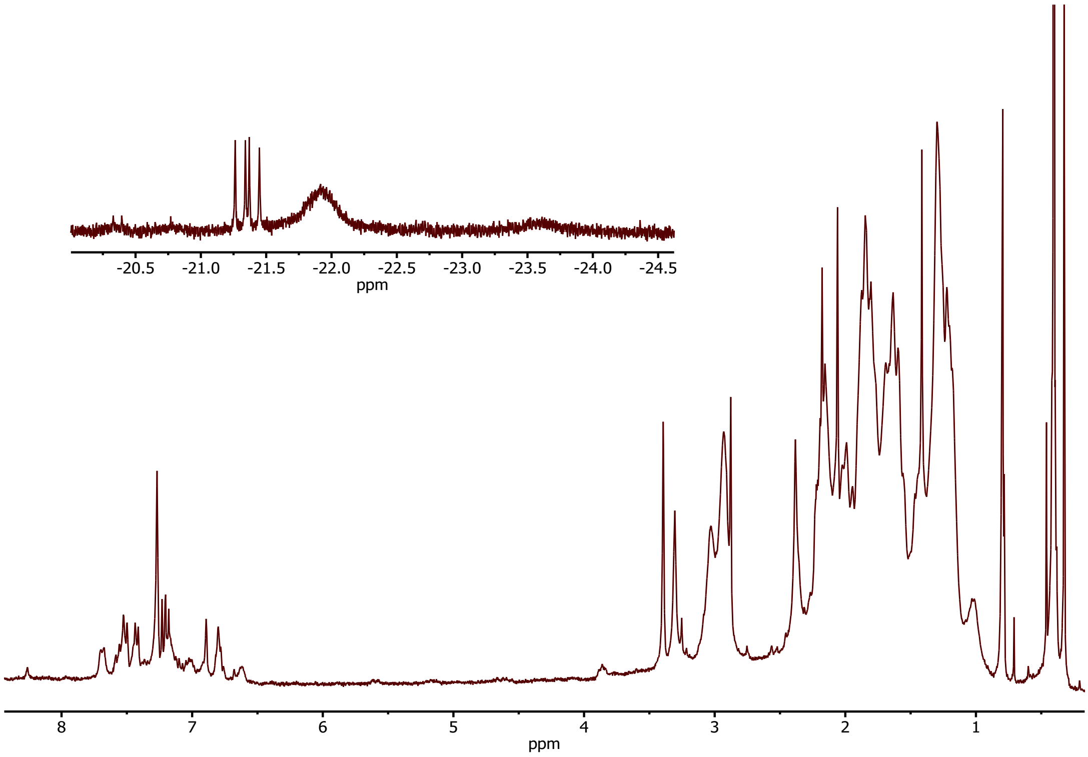

Figure S63. ^{1}H NMR spectrum of the reaction of pyrrolidine with **3** (1 equiv.) in C_6D_6 at r.t. after 10 min.

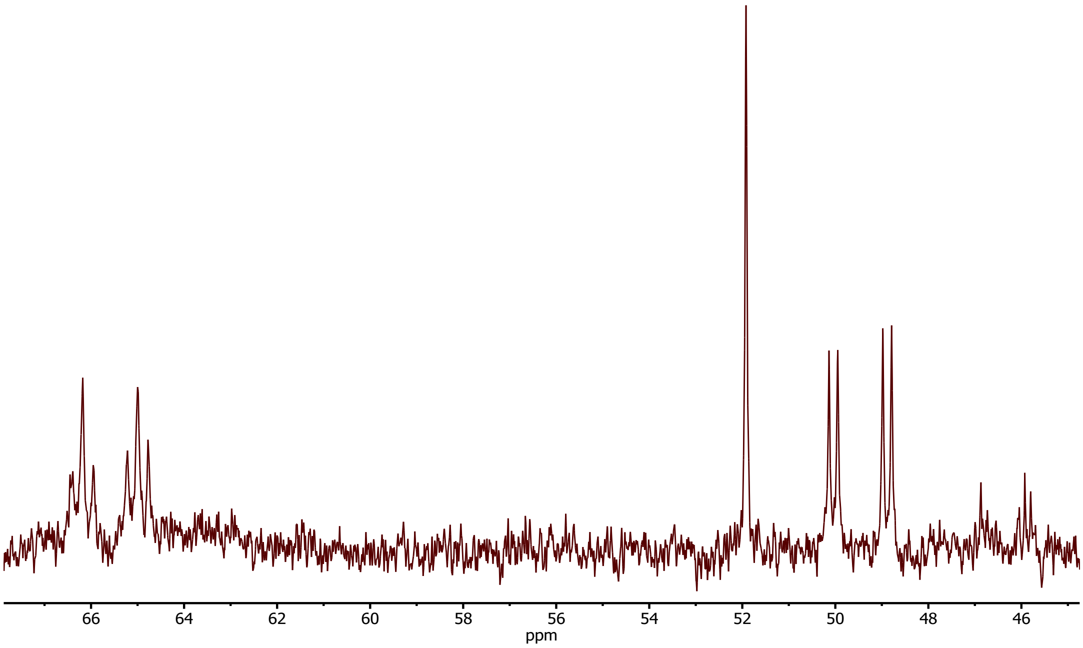

Figure S64. ^{31}P NMR spectrum of the reaction of pyrrolidine with **3** (1 equiv.) in C_6D_6 at r.t. after 10 min.

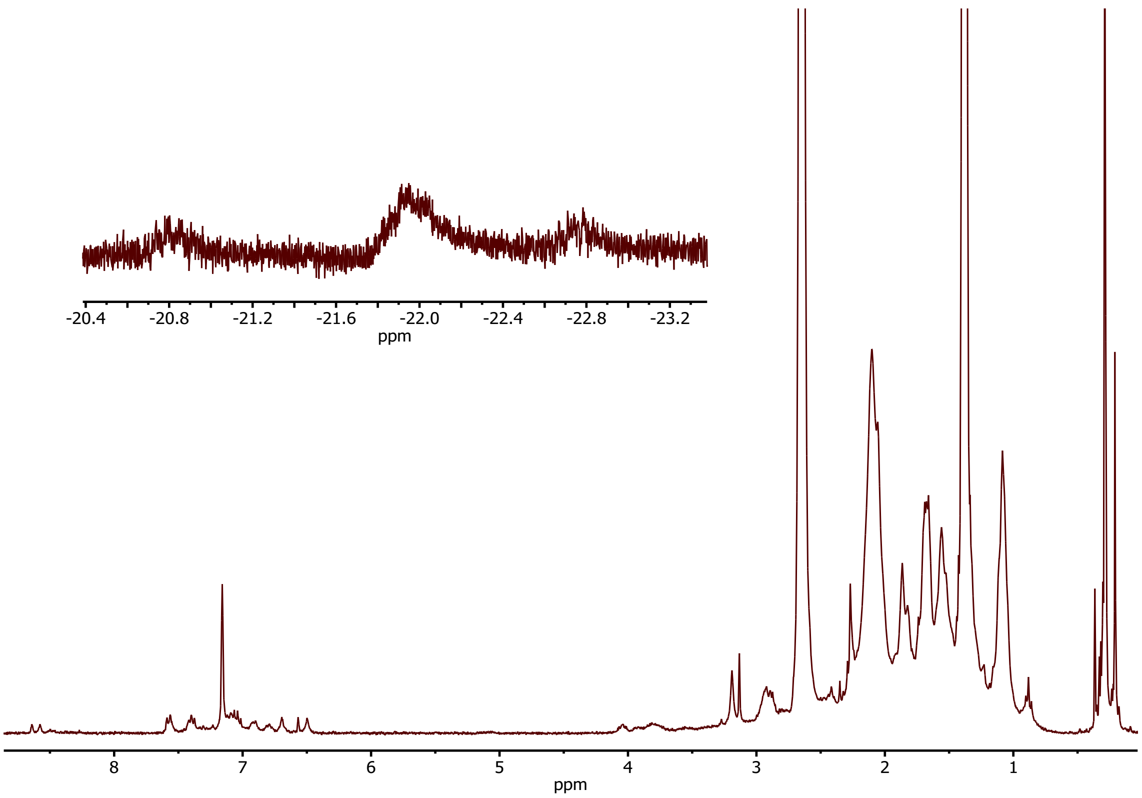

Figure S65. ^{1}H NMR spectrum of the reaction of pyrrolidine with **3** (4 equiv.) in C_6D_6 at r.t. after 3 h.

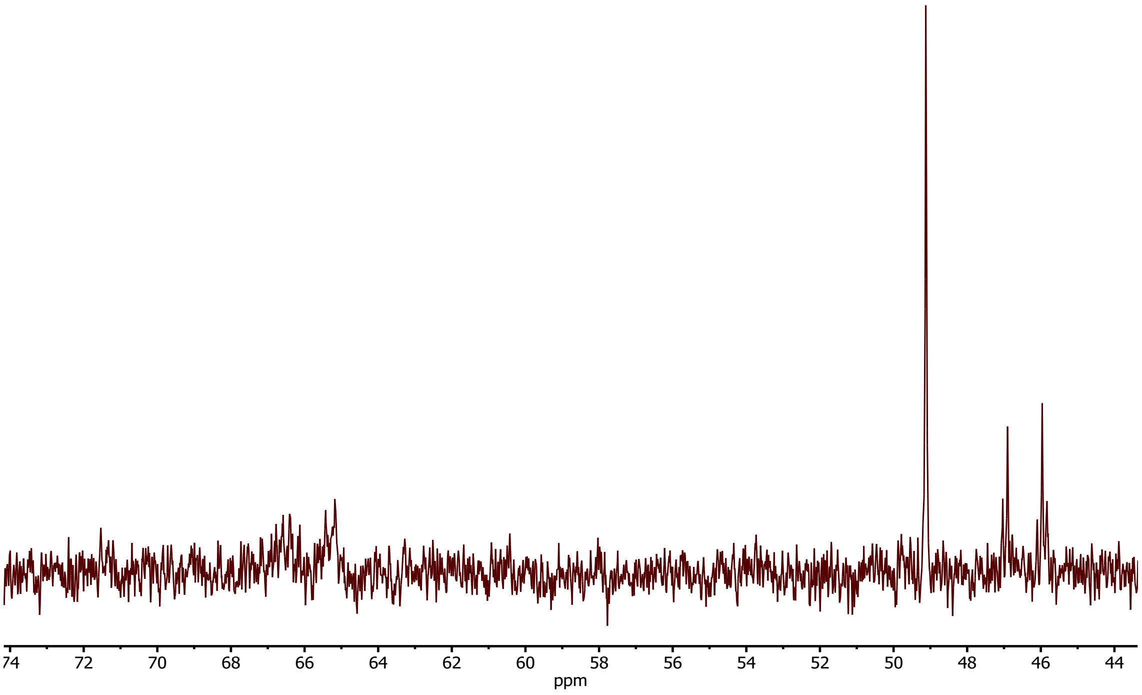

Figure S66. ^{31}P NMR spectrum of the reaction of pyrrolidine with **3** (4 equiv.) in C_6D_6 at r.t. after 3 h.

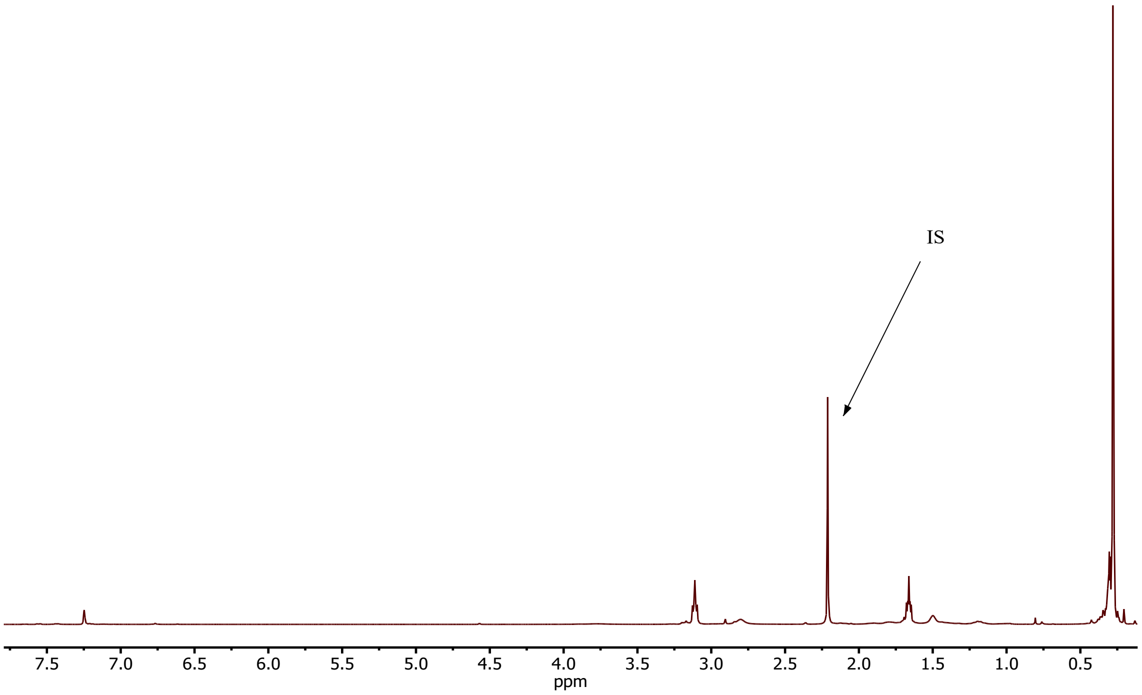

Figure S67. ^{1}H NMR spectrum of the **3**-catalyzed reaction of pyrrolidine with $HSiMe(SiOMe_3)_2$ in C_6D_6 after 10 min at r.t. using hexamethylbenzene as IS.

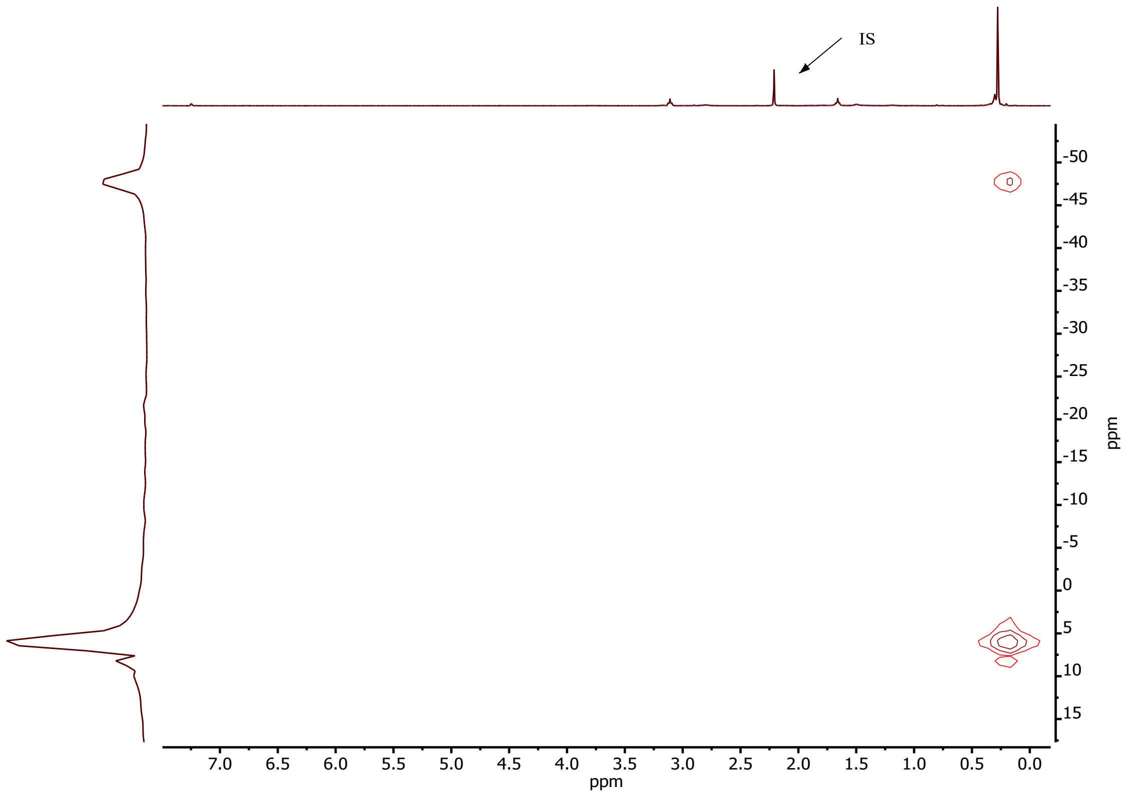

Figure S68. ^{1}H-^{29}Si HMQC NMR spectrum of the **3**-catalyzed reaction of pyrrolidine with $HSiMe(SiOMe_3)_2$ in C_6D_6 after 10 min at r.t. using hexamethylbenzene as IS.

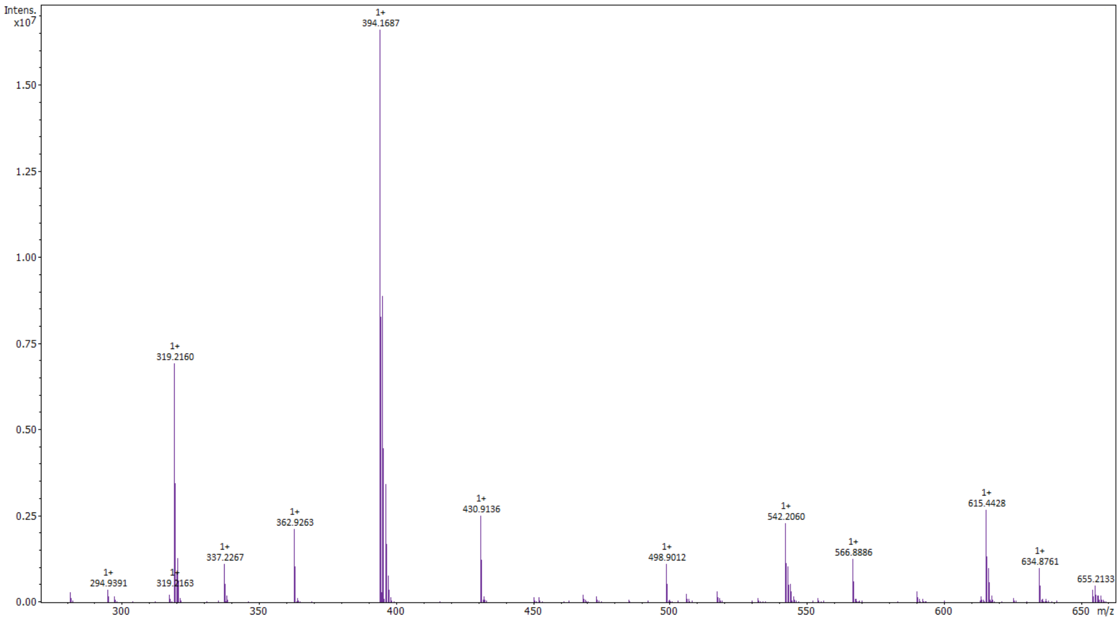

Figure S69. HR-MS spectrum of the **3**-catalyzed reaction of pyrrolidine with $HSiMe(SiOMe_3)_2$ in C_6D_6.

11. Cartesian coordinates

Cartesian coordinates (in Å) of all the stationary points discussed in the text. All calculations have been performed at the B3LYP-GD3/def2-TZVP level.

2:

Rh	6.62204500	5.60899400	10.09712300
P	6.37439800	3.39631400	10.72832800
Si	4.69949200	6.51674200	10.87843800
O	4.89826100	8.11857300	10.19046500
N	6.92048700	7.55231100	9.24120500
C	8.17063800	9.27009200	8.02544200
C	8.09594300	1.01245000	10.59590900
H	7.74605300	0.57452300	11.53813900
H	7.43294700	0.64124100	9.81043300
C	8.06737900	2.55485500	10.70198300
H	8.47766700	2.94823000	9.76044000
C	5.32020700	1.49347600	7.09523100
H	5.78173100	1.55934300	6.10290100
H	5.42905200	0.45015900	7.42468000
C	5.97837400	8.45751300	9.49934500
C	6.06634300	2.41329100	8.07529000
H	6.03220100	3.44534700	7.70575200
H	7.12270000	2.12756700	8.11268400
C	3.18699200	1.82271100	8.41224500
H	3.20001800	0.79409700	8.80017300
H	2.13438600	2.12420400	8.35518000
C	7.24112500	11.65486800	7.86785000
H	8.14199700	12.13572500	8.26429300
H	6.37462600	12.23302000	8.19553900
H	7.29683300	11.71246200	6.77520300
C	5.19814300	1.56753300	12.66403500
H	4.72299900	1.05950500	11.81895100

H	6.14407800	1.04771200	12.85818300
C	10.45722500	2.53817800	11.53054100
H	10.81108600	3.02156900	10.60845000
H	11.12589800	2.86976900	12.33329100
C	5.48821700	3.04840500	12.34300800
H	4.51981600	3.54330700	12.17407600
C	9.30720500	9.63007300	7.26218200
H	9.40941300	10.64874100	6.90611700
C	3.93582800	2.74265000	9.38989600
H	3.45919600	2.69449000	10.37527400
H	3.85590100	3.77762100	9.04528700
C	4.29718600	1.44929500	13.90586000
H	3.31840300	1.89550100	13.67848100
H	4.11526000	0.39170300	14.13122300
C	5.42536400	2.36663500	9.47935500
H	5.48500000	1.33268200	9.84017800
C	8.03280300	7.92570800	8.50155700
C	10.27755100	8.69934900	6.96544700
H	11.14614100	8.97707900	6.37654400
C	6.06325100	9.80447200	9.05940200
H	5.25159100	10.47124500	9.32543300
C	9.05107300	6.97240900	8.18996800
C	7.14208900	10.22577200	8.33235800
C	9.00835100	5.54706800	8.67960000
C	4.90934700	2.16186800	15.11999600
H	4.23201000	2.09720400	15.97972200
H	5.83636700	1.64636000	15.40885100
C	9.52821700	0.52407700	10.31078300
H	9.53807100	-0.57088800	10.25038700
H	9.83694100	0.89543300	9.32295100
C	6.13763900	3.74165300	13.55960100
H	7.09225400	3.25598600	13.78606000

H	6.37247200	4.78300500	13.33309800
C	10.52501700	1.01336100	11.37029500
H	10.28297000	0.54239300	12.33376100
H	11.54173800	0.69471700	11.11115600
C	10.13671900	7.38015000	7.42962600
H	10.90620700	6.65068100	7.19161900
C	3.82910700	1.84797900	7.01862500
H	3.72110500	2.85511200	6.59184600
H	3.30621200	1.16059700	6.34332200
C	2.98690500	6.02466300	10.26165700
H	2.95530600	5.92960600	9.17300100
H	2.27222600	6.80044500	10.56174700
H	2.65626600	5.07850400	10.70069800
C	5.23076300	3.62737800	14.79455300
H	5.71088000	4.11567200	15.65028200
H	4.29135800	4.16854500	14.61258200
C	9.02381000	3.00599400	11.82539900
H	8.98911500	4.08877100	11.95900300
H	8.69989900	2.55514200	12.77143800
C	4.56216700	6.83391000	12.72802500
H	4.21403000	5.94124000	13.25567200
H	3.83376200	7.63465300	12.90305200
H	5.53070800	7.12636700	13.13796300
H	9.70582600	4.91740600	8.11962900
H	8.00979900	5.09295100	8.52624000
H	9.27767900	5.48886600	9.73878700
H	5.70941400	5.24926400	8.89112600
Cl	8.08970600	6.42928900	11.97392200

3:

Rh	6.53776800	5.57120200	9.96784600
P	6.31004400	3.36226300	10.64275600
S	8.35268900	7.36934400	12.21131900
Si	4.58528900	6.48939500	10.67987500
O	4.80596700	8.07856500	9.98307900
O	7.86356500	6.03289600	11.69267400
O	7.33366400	8.43159600	12.17903400
O	9.71000300	7.70813600	11.76019700
N	6.90228200	7.53230200	9.20382800
F	9.27141200	5.81831300	14.15435600
F	9.10176200	7.92396800	14.69083300
C	8.28344300	9.29370000	8.21229900
C	8.08732100	1.02008900	10.49707100
H	7.74907600	0.57561400	11.44011600
H	7.43198000	0.63222300	9.71324700
C	8.02291600	2.56217000	10.59573900
H	8.41776500	2.95640800	9.64888600
C	5.14628600	1.35116300	7.11555100
H	5.56223100	1.40661500	6.10281900
H	5.29665200	0.31870900	7.46200400
C	5.94798200	8.43899200	9.41707700
C	5.91030000	2.31088200	8.04189400
H	5.83119400	3.33374200	7.65368000
H	6.97529200	2.05616600	8.03801700
F	7.31461800	6.68688500	14.55338600
C	3.06397700	1.66072700	8.51820200
H	3.11775300	0.64253400	8.92903900
H	2.00333700	1.93742300	8.50035900
C	7.40014400	11.69757800	8.10151400
H	8.25684900	12.13777400	8.62306700
H	6.50895800	12.26939000	8.36804800

H	7.57600900	11.81835600	7.02692200
C	5.36429700	1.61185200	12.75657200
H	4.89158000	1.00651100	11.97582300
H	6.35869600	1.18488700	12.93452500
C	10.41713300	2.60792800	11.41638700
H	10.75612900	3.09655400	10.49176200
H	11.07932400	2.95921300	12.21591700
C	5.51606200	3.08132000	12.31458600
H	4.50541600	3.48663500	12.15561900
C	9.49546100	9.67708600	7.59089800
H	9.64434700	10.71250000	7.30741700
C	3.83232700	2.62013600	9.44136700
H	3.39976500	2.58632500	10.44749600
H	3.71573400	3.64379200	9.07442700
C	4.54237300	1.51825900	14.05422700
H	3.51964600	1.86859800	13.85439300
H	4.46037400	0.46978900	14.36423800
C	5.33213300	2.27773200	9.47380500
H	5.43345200	1.25506100	9.85774400
C	8.08666400	7.92863500	8.59554900
C	10.48212700	8.74935000	7.34169600
H	11.40963400	9.04641700	6.86252600
C	6.08614500	9.80241200	9.05484800
H	5.26023600	10.46644000	9.27962700
C	9.12050600	6.97975700	8.33644900
C	7.23792700	10.24617700	8.46682900
C	9.01610800	5.53408100	8.74926300
C	5.15893400	2.36606900	15.17652100
H	4.53892100	2.30986200	16.07883600
H	6.14148300	1.95036800	15.44172500
C	9.53055500	0.56639800	10.20923000
H	9.56679400	-0.52813000	10.15398900

H	9.82666200	0.94036400	9.21864400
C	6.15851800	3.92008400	13.43974500
H	7.16828200	3.55401800	13.64615700
H	6.27337300	4.95554100	13.12853300
C	10.51935600	1.08448100	11.26266300
H	10.29264400	0.61259200	12.22933200
H	11.54217400	0.78918200	11.00076900
C	10.28183300	7.40929200	7.71295900
H	11.06475700	6.68206500	7.51586800
C	3.64462300	1.66749900	7.09754500
H	3.49243900	2.66083600	6.65230900
H	3.11032400	0.95139300	6.46245200
C	2.89637300	5.99627100	10.00628300
H	2.91178400	5.84625600	8.92348500
H	2.18997200	6.80429100	10.23133800
H	2.52059800	5.08430400	10.47935400
C	5.33379500	3.82432400	14.73073500
H	5.81959200	4.41795100	15.51322800
H	4.34278500	4.27143400	14.56857400
C	8.97389500	3.04095900	11.71490900
H	8.91525900	4.12094200	11.85275900
H	8.66376800	2.58099600	12.66126900
C	4.38742200	6.80853600	12.52297100
H	4.05271300	5.90950800	13.04807600
H	3.61932000	7.58041600	12.65344600
H	5.31555900	7.16456100	12.97124100
C	8.52528600	6.92526100	14.01239300
H	9.74587100	4.91808000	8.21645300
H	8.02679000	5.10362700	8.49416700
H	9.20661700	5.42074700	9.82014800
H	5.64980100	5.23485800	8.75467000

INT1:

Rh	6.59896000	5.63181600	10.09754200
P	6.33660500	3.38049400	10.71594900
S	8.50455900	7.35998000	12.31358800
Si	4.64952000	6.55728900	10.83577200
O	4.85814600	8.13490700	10.11902300
O	7.87781400	6.04165700	11.87919700
O	7.53430400	8.45912000	12.43443100
O	9.78541000	7.63689200	11.64986500
N	6.92930300	7.59174400	9.26758100
F	9.76868200	5.85024400	14.08693600
F	9.53461200	7.93176100	14.67936400
C	8.15834300	9.31008200	8.02152400
C	8.09543200	1.00807000	10.59354900
H	7.73000700	0.56237200	11.52657500
H	7.44971800	0.63561600	9.79365100
C	8.04991800	2.55119700	10.70136500
H	8.46463000	2.94732100	9.76292800
C	5.29632800	1.47471100	7.06256100
H	5.75261300	1.56444000	6.06958900
H	5.43422800	0.42959500	7.37511300
C	5.95924700	8.48382000	9.47366600
C	6.02673600	2.40164300	8.04992700
H	5.97643900	3.43517500	7.68570100
H	7.08788000	2.13274200	8.08291600
F	7.82914200	6.58128700	14.75327900
C	3.16013200	1.75168000	8.38885100
H	3.18656900	0.72186400	8.77301800
H	2.10253600	2.03610100	8.33513900
C	7.18891800	11.67722400	7.81218500
H	8.07208400	12.18291100	8.21752500
H	6.30540800	12.24032200	8.12028100

H	7.25939100	11.72552700	6.71977000
C	5.18146700	1.54280400	12.68177600
H	4.71600800	1.01731500	11.84143500
H	6.13383600	1.03708400	12.88253100
C	10.43689100	2.54514100	11.56487600
H	10.81161600	3.03140200	10.65294400
H	11.08636300	2.87987900	12.38177100
C	5.44969000	3.02588600	12.34330300
H	4.47257600	3.50106600	12.16730900
C	9.29169900	9.67467000	7.25689100
H	9.37200600	10.68767700	6.87936700
C	3.89337000	2.68073700	9.37240100
H	3.41901400	2.61170200	10.35770000
H	3.78449000	3.71767200	9.03950400
C	4.28028900	1.41819000	13.92457100
H	3.28992600	1.83501800	13.69180200
H	4.12343400	0.35841600	14.15918400
C	5.39365600	2.33860500	9.45830800
H	5.47577800	1.30448200	9.81629300
C	8.04462100	7.97625000	8.53022300
C	10.28331000	8.75928000	6.98678100
H	11.14937700	9.04109000	6.39613400
C	6.02741000	9.82338900	9.01040700
H	5.19787400	10.47737600	9.25137500
C	9.08838900	7.04352900	8.25381200
C	7.10984100	10.25298300	8.29601000
C	9.08042500	5.64024400	8.79889100
C	4.87024200	2.15539100	15.13506000
H	4.18385300	2.09250800	15.98773600
H	5.79887900	1.65500700	15.44452000
C	9.53545400	0.52402100	10.33466700
H	9.54796100	-0.57144700	10.28142800

H	9.85971500	0.88611300	9.34843000
C	6.08083100	3.74131300	13.55963900
H	7.04561100	3.27941300	13.79346500
H	6.29283400	4.78658800	13.33104500
C	10.51632500	1.02110800	11.40521400
H	10.27229600	0.54555900	12.36593500
H	11.53801700	0.71097700	11.15556200
C	10.16848200	7.45199200	7.48677400
H	10.95662200	6.73337900	7.27888500
C	3.79626100	1.78978500	6.99291200
H	3.65703900	2.79011900	6.55951200
H	3.28902900	1.08549400	6.32301100
C	2.94579400	6.05597000	10.19038000
H	2.93385200	5.93264700	9.10408000
H	2.23503200	6.85017600	10.44905200
H	2.58496500	5.12953700	10.64768500
C	5.17549200	3.62120700	14.79653500
H	5.65412400	4.12130100	15.64619000
H	4.23098000	4.15395100	14.61526500
C	8.99628300	3.01076800	11.83426600
H	8.96017500	4.09317000	11.96513000
H	8.66385700	2.56461500	12.77961500
C	4.48440000	6.91036600	12.67816900
H	4.18805800	6.02184000	13.24317700
H	3.70088100	7.66683800	12.81047700
H	5.41578300	7.30555000	13.08817900
C	8.93832000	6.90534600	14.06471400
H	9.84175600	5.02830900	8.30617300
H	8.11493600	5.13279100	8.60528000
H	9.29454200	5.64041400	9.87173700
H	5.72535000	5.31419300	8.86764100

TS1:

Rh	6.99507900	5.34864100	9.91999700
P	6.60858700	3.22333200	10.76705200
S	8.88271700	7.11175200	12.30164100
Si	5.00131000	6.38648300	10.31680300
O	5.64699600	7.99176900	10.56857000
O	8.33712800	5.85958400	11.64860300
O	7.86047500	7.92606300	12.97304900
O	9.88764700	7.81633200	11.48437500
N	7.27074500	7.45333000	9.04469000
F	10.77610600	5.47648700	13.19661100
F	10.51445200	7.28971400	14.37152500
C	8.91755600	9.15452800	8.42583500
C	8.07475800	0.67624600	10.73410600
H	7.53927900	0.30483400	11.61552400
H	7.48880900	0.37441600	9.86216600
C	8.22133800	2.21599900	10.81424900
H	8.72638700	2.56259600	9.90380900
C	5.14054800	1.07398200	7.40615400
H	5.52227200	1.02769900	6.37925300
H	5.23013900	0.05826900	7.81759700
C	6.72159100	8.34194000	9.87596900
C	6.00365600	2.04071200	8.23468200
H	5.98244000	3.03389600	7.77078300
H	7.04917200	1.71565800	8.21477400
F	9.06707400	5.67386300	14.52941600
C	3.13777100	1.64865700	8.84231300
H	3.11845300	0.66309500	9.32891000
H	2.10241400	2.00978700	8.83264000
C	9.06613800	11.32438900	9.78822500
H	10.07029300	11.13681900	10.18407700
H	8.50020300	11.86513500	10.54931600

H	9.16994900	11.97281900	8.91132700
C	5.55015800	1.71520900	13.02992300
H	5.08697200	1.05362200	12.28963700
H	6.51804700	1.27026300	13.29016600
C	10.49433200	1.89520900	11.93120400
H	11.04248300	2.25230900	11.04952100
H	11.09353500	2.17966600	12.80410600
C	5.77007400	3.13448300	12.45881500
H	4.77869100	3.54838400	12.23407600
C	10.01096900	9.53426900	7.61382200
H	10.52494000	10.46667900	7.81852100
C	4.00892700	2.60497100	9.67728500
H	3.60795100	2.65591100	10.69495600
H	3.94160100	3.61455800	9.25592400
C	4.67374200	1.76099900	14.29521400
H	3.66883600	2.11549700	14.02444700
H	4.54898300	0.74590300	14.69162300
C	5.48542000	2.15898000	9.68537200
H	5.51969900	1.15877800	10.13541700
C	8.27167200	7.90014300	8.19312000
C	10.41010500	8.74557800	6.55559000
H	11.24691400	9.04326200	5.93132400
C	7.25052200	9.64081900	10.09255600
H	6.77589800	10.25580300	10.84690100
C	8.61646600	7.14803600	7.02813400
C	8.39404600	10.02252100	9.44846300
C	7.78256900	5.98191000	6.55587300
C	5.26977400	2.68790100	15.36334900
H	4.60746500	2.73584900	16.23595000
H	6.22132500	2.26537700	15.71634500
C	9.44929800	-0.01505000	10.66383800
H	9.30637700	-1.10199700	10.62614600

H	9.94512800	0.26589400	9.72358100
C	6.39046800	4.05774900	13.53217700
H	7.38806700	3.70381800	13.80499200
H	6.53296600	5.06341900	13.13516900
C	10.34808300	0.37173400	11.84433500
H	9.90613700	-0.00782200	12.77685900
H	11.33056000	-0.10571100	11.74784100
C	9.68425300	7.58175200	6.25065800
H	9.94025300	7.01493700	5.35907800
C	3.66516900	1.49614900	7.40931600
H	3.56370800	2.45537300	6.88231000
H	3.05778500	0.77021600	6.85592500
C	3.81793200	6.57884900	8.84956700
H	4.36127100	6.88237100	7.95031900
H	3.06723300	7.34558000	9.07481200
H	3.28700500	5.64886600	8.62213500
C	5.51754600	4.09309400	14.79809100
H	5.99969900	4.72952500	15.54874400
H	4.55140900	4.56279300	14.56571200
C	9.12820800	2.59810300	12.00615900
H	9.26124500	3.68013300	12.05164000
H	8.64452600	2.29161500	12.94138900
C	3.95174100	6.17431600	11.86788200
H	3.34083100	5.26683600	11.84094400
H	3.26860600	7.03076300	11.91960100
H	4.55512300	6.17332000	12.77718400
C	9.86774900	6.34325600	13.68243500
H	8.15615300	5.61582100	5.59533200
H	6.73818200	6.28387900	6.42312000
H	7.77000400	5.15122700	7.26635300
H	6.18988000	4.88628400	8.70532000
C	11.09238000	5.11445100	9.74768500

C	10.49589000	3.75650800	7.94381500
C	11.97803300	4.14928200	7.67701800
C	12.31411400	5.16558400	8.80255400
H	10.89371100	6.05459900	10.26271300
H	11.22686700	4.33884100	10.50838600
H	10.45043800	2.76309100	8.40755400
H	9.88618900	3.71784500	7.03637000
H	12.63042300	3.27062100	7.70500800
H	12.09560800	4.60244300	6.68761900
H	13.25044700	4.93068200	9.31801100
H	12.41778500	6.17352100	8.38688100
N	9.94134200	4.74306300	8.89397800
H	9.70471200	5.57698700	8.36104200

INT2:

Rh	7.15888800	5.29075200	9.84970800
P	6.75124200	3.20986900	10.77190400
S	9.03856400	7.12716200	12.29031900
Si	5.12309000	6.30811700	10.28917200
O	5.74625500	7.91961000	10.61750000
O	8.37780000	5.90928500	11.68757900
O	8.11963900	8.01733700	13.01153800
O	10.04091900	7.75630000	11.40757000
N	7.29307900	7.50428200	8.98355300
F	10.92594200	5.43554600	13.09279900
F	10.74464600	7.23116000	14.30881500
C	8.94563600	9.23293000	8.45374800
C	8.21310400	0.64519500	10.95754600
H	7.64186400	0.29891400	11.82615000
H	7.67177100	0.30437900	10.07112200
C	8.34288100	2.18808100	10.98826000
H	8.91773300	2.48036200	10.10258800

C	5.46733800	0.90141800	7.43729300
H	5.90915500	0.80142900	6.43860100
H	5.52085300	-0.09362500	7.90210000
C	6.79107800	8.32000200	9.91312300
C	6.29145100	1.89942900	8.26865700
H	6.31406400	2.86846400	7.75641500
H	7.33058800	1.55931500	8.32926600
F	9.26660800	5.64428100	14.48502200
C	3.39025300	1.56064200	8.72190900
H	3.33325000	0.60009300	9.25358500
H	2.36084000	1.92785700	8.63367700
C	9.19422500	11.25773200	10.01095200
H	10.19819400	11.00297600	10.36806400
H	8.66017000	11.74402700	10.82956700
H	9.30291200	11.97877100	9.19335700
C	5.56714200	1.77677900	13.01701800
H	5.13826900	1.09750800	12.27236900
H	6.51825300	1.33538000	13.33735100
C	10.55519100	1.94734700	12.23161500
H	11.13915500	2.29782000	11.36914400
H	11.10419200	2.26662500	13.12481500
C	5.82052500	3.18109800	12.42094500
H	4.84484300	3.58866300	12.13129400
C	10.01584300	9.65869900	7.63465700
H	10.56459400	10.55499400	7.90172800
C	4.22017200	2.55207600	9.55757600
H	3.76068600	2.65865900	10.54572200
H	4.18935800	3.53928600	9.08261800
C	4.63056000	1.85377800	14.23665500
H	3.64195400	2.20788000	13.91123200
H	4.48120300	0.84745600	14.64683500
C	5.68905100	2.09628000	9.67722900

H	5.68804700	1.12033300	10.17884600
C	8.25747400	8.02233600	8.13244700
C	10.35086300	8.96065800	6.49211600
H	11.17432500	9.29206000	5.86699500
C	7.34772700	9.59241400	10.21817500
H	6.91643800	10.14104300	11.04591400
C	8.52034700	7.38608000	6.88056700
C	8.47697900	10.01118800	9.57247400
C	7.58463800	6.34171900	6.32058500
C	5.18183800	2.79942700	15.31162400
H	4.48213900	2.86675400	16.15321500
H	6.11583000	2.38202700	15.71436000
C	9.60114200	-0.02398100	10.97391800
H	9.47881700	-1.11397200	10.96998500
H	10.13259800	0.23160300	10.04581200
C	6.38552000	4.13141500	13.50283000
H	7.36968400	3.78905700	13.83198300
H	6.54442300	5.12804800	13.08949900
C	10.44397100	0.41877300	12.17700900
H	9.97334900	0.05553300	13.10180400
H	11.43905500	-0.03973700	12.13307000
C	9.57482900	7.85529300	6.10469200
H	9.77174200	7.37512300	5.14924000
C	3.99912300	1.33495400	7.33168000
H	3.93917100	2.26839700	6.75450300
H	3.41842800	0.58754600	6.77822600
C	3.98085600	6.53317500	8.78750100
H	4.54992000	6.81893100	7.89898100
H	3.24022000	7.31566500	8.99122600
H	3.43346200	5.61350000	8.55343300
C	5.45589300	4.19164300	14.72767600
H	5.90394100	4.84451500	15.48540000

H	4.50044200	4.65392600	14.44453600
C	9.16959100	2.61452700	12.22204200
H	9.26859000	3.70001900	12.26174400
H	8.64161100	2.30854700	13.13262500
C	3.96930500	6.11889800	11.77490400
H	3.35243400	5.21627600	11.71930900
H	3.29012800	6.97990700	11.76772100
H	4.50349800	6.12635600	12.72608300
C	10.05214600	6.31449700	13.62457200
H	8.00962200	5.88326500	5.42297600
H	6.63336800	6.80901400	6.03670100
H	7.33427700	5.56062400	7.04071000
H	6.38318600	4.78113600	8.63787200
C	10.63962200	4.98187800	9.64573100
C	9.67981000	3.85713700	7.81526400
C	11.13897900	4.10336500	7.39699100
C	11.81458100	4.58682500	8.70508200
H	10.72665200	5.98346800	10.06566600
H	10.56034100	4.28694400	10.48172500
H	9.57770400	2.86828900	8.27564200
H	8.96333500	3.91604800	6.99383800
H	11.61281800	3.21355700	6.97120900
H	11.17302300	4.88984900	6.63473500
H	12.41938400	3.79326000	9.15557500
H	12.48374100	5.43142200	8.51947400
N	9.38768700	4.89282200	8.83620500
H	9.33245100	5.77252400	8.32429900

TS2:

Rh	7.41740800	4.99880600	10.13592000
P	6.66612900	3.02259500	10.89115500
S	8.75900500	6.74326300	12.91264300
Si	5.54049600	6.29456000	10.54131300
O	6.36187100	7.82800300	10.64368500
O	8.54783600	5.58365900	11.96251700
O	7.63089500	6.97232100	13.82698600
O	9.39734900	7.91141100	12.28553600
N	7.40730100	7.65213400	8.61221700
F	11.15424300	5.64259800	13.24672600
F	10.47908500	6.85694300	14.92158500
C	8.90874800	9.44595800	7.87778900
C	7.92904200	0.44710400	11.45964300
H	7.62370000	0.43244000	12.51251900
H	7.11096500	-0.00035200	10.88583700
C	8.19627400	1.90698500	11.02617300
H	8.53141200	1.88050700	9.97943800
C	5.31845100	0.84785000	7.47433800
H	5.81195900	0.64818400	6.51569100
H	5.16564100	-0.12948100	7.95421300
C	7.18360900	8.33229600	9.71915500
C	6.24260700	1.70591200	8.35764900
H	6.49209400	2.63681200	7.83367700
H	7.18388500	1.16924600	8.51472500
F	9.62470600	4.88219400	14.59425500
C	3.29093000	1.87765500	8.58100000
H	3.03351300	0.95535400	9.12125000
H	2.34797900	2.40934900	8.40698100
C	9.35336200	11.44634100	9.42087500
H	10.44187100	11.32159300	9.43854100
H	9.04375000	11.82793100	10.39589400

H	9.12628600	12.20850900	8.66653500
C	4.92520700	1.68402200	12.85312100
H	4.28823500	1.36863000	12.02111100
H	5.62672200	0.86393200	13.04219500
C	10.63150800	1.62954300	11.69425200
H	10.97816200	1.66579100	10.65074500
H	11.43716200	2.05607800	12.30303400
C	5.69700100	2.98212100	12.51548300
H	4.94675500	3.76061200	12.34049400
C	9.78384200	9.93728800	6.87917900
H	10.31277500	10.87008200	7.04398500
C	4.21590000	2.74184900	9.45500400
H	3.71477800	2.97580700	10.40112700
H	4.40029100	3.69564400	8.95048400
C	4.04663300	1.88688400	14.10294700
H	3.26809500	2.62893700	13.87586700
H	3.52476300	0.95044500	14.33518100
C	5.57112400	2.04983000	9.70420000
H	5.37471700	1.10270900	10.22311100
C	8.22513700	8.20835600	7.65911800
C	9.96269300	9.24385000	5.70004600
H	10.63743200	9.62412600	4.93883000
C	7.78221500	9.59257300	10.00601900
H	7.56503300	10.05635600	10.96038700
C	8.37676400	7.52718300	6.41076000
C	8.66260300	10.14326900	9.11298300
C	7.54510900	6.30715000	6.09916800
C	4.86367200	2.36364200	15.31127400
H	4.20270600	2.53948800	16.16829500
H	5.55986500	1.56745100	15.61253600
C	9.19508800	-0.41511400	11.30251700
H	8.98317100	-1.43969600	11.63130800

H	9.45824000	-0.47803600	10.23685000
C	6.53876000	3.43585400	13.72890800
H	7.29026300	2.66832300	13.95284000
H	7.07623800	4.35734500	13.50369800
C	10.37792700	0.16810100	12.08689500
H	10.15823300	0.11572600	13.16257200
H	11.27801800	-0.43616000	11.92273300
C	9.24852500	8.05155700	5.46883000
H	9.36633000	7.53755300	4.51794100
C	3.95702800	1.51778600	7.24646200
H	4.09810800	2.43341500	6.65522900
H	3.30433800	0.86266600	6.65752600
C	4.25378900	6.47808300	9.15947100
H	4.70971200	6.42979900	8.16868900
H	3.76421100	7.45371100	9.26614400
H	3.47520600	5.71173800	9.22803200
C	5.65761500	3.63188100	14.97316500
H	6.28851100	3.93053600	15.81767600
H	4.96403900	4.46450400	14.79659400
C	9.36839500	2.49509200	11.84466700
H	9.57332600	3.52174800	11.54030400
H	9.09951200	2.54815800	12.90434100
C	4.62121100	6.35951700	12.18887500
H	3.86661000	5.57375000	12.29418400
H	4.09813800	7.32259100	12.23066600
H	5.31695500	6.32329400	13.03069300
C	10.08292600	5.99004300	13.98385400
H	7.87050400	5.84519500	5.16262300
H	6.48859500	6.58075400	5.99205900
H	7.57731700	5.55883800	6.89600900
H	6.74200000	4.58574500	8.82136300
C	10.70609100	5.77783200	9.75526100

C	10.13122600	4.09454300	8.23523800
C	11.52393200	4.58898400	7.76854500
C	11.86863400	5.75481000	8.73982000
H	10.45809400	6.76842300	10.13838200
H	10.91974600	5.14436500	10.61932300
H	10.23404100	3.22423600	8.89112700
H	9.46010400	3.82168700	7.41722200
H	12.26306400	3.78319100	7.80410500
H	11.48326600	4.94372300	6.73427100
H	12.83452100	5.61767700	9.23408800
H	11.91512600	6.70321400	8.19540400
N	9.54166300	5.19788200	9.03268600
H	9.22648300	5.90915200	8.37269900

INT3:

Rh	7.83267700	4.38813800	10.16265400
P	6.63468200	2.61875800	11.11404200
S	8.49561600	6.40522900	12.89936600
Si	6.16036300	5.90394700	9.70212800
O	6.84003600	7.47245000	9.88877700
O	8.75089900	5.16665400	12.05889400
O	7.16923500	6.41714700	13.53773800
O	8.97179700	7.65688000	12.28978800
N	7.93741200	7.96837600	7.92267500
F	10.94585400	5.96671400	13.80255600
F	9.67356400	7.09774600	15.15831700
C	8.95121400	10.19131100	7.67505300
C	7.63639200	-0.02780200	11.89506600
H	7.49444200	0.15782300	12.96612600
H	6.69192400	-0.43621900	11.51861100
C	7.98777000	1.29425700	11.18000900
H	8.12182400	1.06127800	10.11501600

C	4.65967000	0.47048900	7.99076100
H	5.01728600	0.16834200	6.99916300
H	4.45629600	-0.45767700	8.54381600
C	7.54585400	8.31784100	9.13270300
C	5.76582400	1.25858200	8.71597200
H	6.05647200	2.12685700	8.11196300
H	6.65427400	0.62467100	8.79791200
F	9.39449100	4.95144500	14.93995600
C	2.89902700	1.78408300	9.24099700
H	2.60669800	0.92755900	9.86519100
H	2.00659200	2.41255600	9.13767500
C	8.89164200	11.84712200	9.63385800
H	9.97572700	12.00502600	9.65907100
H	8.51775900	11.90811900	10.65799300
H	8.45799600	12.67563100	9.06195900
C	4.83476800	1.75787200	13.29396600
H	4.05220800	1.59243200	12.54806600
H	5.33553300	0.79311500	13.44177400
C	10.46123600	0.78918900	11.49800800
H	10.61696100	0.59796500	10.42618000
H	11.40640700	1.18747400	11.88510000
C	5.86154900	2.81679400	12.82928900
H	5.33215200	3.77329100	12.72698400
C	9.66256100	11.09428700	6.84717800
H	9.92244900	12.07680300	7.22688200
C	4.00791600	2.57719200	9.95351900
H	3.64394200	2.92201800	10.92612000
H	4.23299300	3.47518000	9.36891100
C	4.17674600	2.19447600	14.61784200
H	3.59038500	3.10633400	14.43727900
H	3.46473200	1.42567800	14.94269500
C	5.29676600	1.74136500	10.10590500

H	5.04608200	0.84947300	10.69638200
C	8.61569300	8.89627100	7.16966600
C	10.02075400	10.73474100	5.56549900
H	10.56446700	11.43096500	4.93377500
C	7.84161800	9.57972300	9.72639900
H	7.52556800	9.74549500	10.74928300
C	8.97981800	8.54245100	5.83373100
C	8.54816400	10.51562200	9.01738900
C	8.58393900	7.20263300	5.26753100
C	5.21191400	2.46804000	15.71848400
H	4.71135000	2.83480200	16.62263900
H	5.69975400	1.52178700	15.99509500
C	8.75564800	-1.06964500	11.71671900
H	8.49423700	-1.98768900	12.25655500
H	8.82951000	-1.34280100	10.65434100
C	6.92654400	3.01684900	13.93047300
H	7.45030600	2.06722900	14.10371100
H	7.66894700	3.74658300	13.61589700
C	10.11012000	-0.53112800	12.19817500
H	10.06847800	-0.36538700	13.28387300
H	10.89774700	-1.27419800	12.02619100
C	9.67237900	9.46343900	5.06575600
H	9.94633000	9.20006200	4.04710800
C	3.36244500	1.28007400	7.86821400
H	3.53340800	2.14069600	7.20618100
H	2.57831300	0.67540900	7.39746300
C	5.40290000	5.81438600	7.96931200
H	6.17696100	5.89313200	7.20369500
H	4.70251100	6.64666100	7.82995600
H	4.85560500	4.87959500	7.81481000
C	6.27585900	3.46776500	15.24686700
H	7.05240700	3.59331100	16.00994400

H	5.83014200	4.45864800	15.09621100
C	9.35044000	1.83958500	11.66907300
H	9.62109300	2.74876100	11.11631200
H	9.28759600	2.14096400	12.71920200
C	4.81825100	6.03952500	11.02139800
H	4.17259200	5.16045200	11.07301600
H	4.19115700	6.90692900	10.78211200
H	5.27664900	6.21048700	11.99961800
C	9.69896300	6.08580000	14.28410000
H	8.90124200	7.11346100	4.22489700
H	7.49948700	7.05928000	5.31302500
H	9.02938500	6.37431200	5.82889500
H	7.38468300	3.77728200	8.82077300
C	10.43820000	6.16907600	9.76756000
C	10.05908900	4.57306400	8.07943700
C	11.37737500	5.29412500	7.69190700
C	11.49016800	6.47616900	8.69177200
H	10.04702500	7.03824400	10.29490100
H	10.82372300	5.47680700	10.52088000
H	10.27030000	3.63314700	8.59915700
H	9.40812000	4.34271900	7.23361000
H	12.22616800	4.60895500	7.77419800
H	11.35065500	5.64934500	6.65790800
H	12.49240600	6.57462000	9.11778900
H	11.25197600	7.42112100	8.19341900
N	9.34015500	5.47666200	9.02864200
H	8.86470700	6.19366400	8.46684900

TS3:

Rh	7.83237900	4.29109800	10.00997600
P	6.66403600	2.60593100	11.14032000
S	9.24661600	6.26234200	12.47939200
Si	6.20309400	5.88560700	9.68308000
O	6.96076300	7.38305500	10.02530700
O	8.99317500	4.92793600	11.80290600
O	8.07470800	6.81808500	13.17017500
O	10.05936400	7.18766500	11.66923300
N	7.84035800	8.19244300	8.05268000
F	11.49048100	5.13254700	13.32072500
F	10.74301100	6.72149600	14.60704800
C	9.06705500	10.32012600	8.10444700
C	7.64711100	-0.14003400	11.65578600
H	7.07596700	-0.25850500	12.58368600
H	7.00036600	-0.48342200	10.84409800
C	8.04961500	1.34391200	11.47935100
H	8.63725800	1.40536700	10.54923700
C	5.04252300	0.19242400	8.02765600
H	5.48730200	-0.12931900	7.07841000
H	4.87033500	-0.72040000	8.61594800
C	7.65538200	8.31157200	9.35082200
C	6.02865000	1.10629800	8.77535400
H	6.26566900	1.97358300	8.14829100
H	6.97398100	0.57726700	8.93528300
F	9.77266500	4.77261900	14.60679200
C	3.10871700	1.43090100	9.08870300
H	2.83126100	0.58418800	9.73259000
H	2.18431800	1.98679400	8.89189500
C	9.45403900	11.51840800	10.33999400
H	10.54461000	11.56065300	10.24165100
H	9.21645500	11.39563000	11.39862500

H	9.06148500	12.48746400	10.01062800
C	5.20415800	1.68714100	13.49681200
H	4.68439000	0.98935700	12.83083600
H	6.06206100	1.14641500	13.91495000
C	10.23781600	0.87212200	12.68385400
H	10.82643200	1.02178500	11.76706900
H	10.87778100	1.18572300	13.51671100
C	5.70523100	2.93305000	12.73293800
H	4.81895700	3.45958000	12.35425200
C	9.76961500	11.31149700	7.37617300
H	10.20123700	12.15640700	7.90244300
C	4.10050000	2.33313200	9.84522300
H	3.63897300	2.66219900	10.78179400
H	4.29347700	3.23499600	9.25163500
C	4.26615100	2.09121200	14.64962500
H	3.35755300	2.54629200	14.23002000
H	3.94268300	1.19381200	15.19123100
C	5.43493400	1.60442800	10.11163800
H	5.20602600	0.72196700	10.72361600
C	8.51091000	9.20262700	7.40620200
C	9.90124400	11.21263300	6.00752700
H	10.43928600	11.97604800	5.45315900
C	8.15961100	9.38960600	10.13404800
H	7.99268800	9.36355900	11.20407400
C	8.62885400	9.12896500	5.98359800
C	8.87853100	10.38653500	9.52877900
C	7.97071600	8.00578500	5.22352800
C	4.93826200	3.08614500	15.60619900
H	4.23392000	3.39243300	16.38892400
H	5.77374800	2.58598100	16.11674200
C	8.89538500	-1.04111400	11.71280700
H	8.58665800	-2.08360400	11.85754000

H	9.40836400	-1.00139900	10.74105600
C	6.40768600	3.91237500	13.69757400
H	7.30731300	3.45018600	14.11703300
H	6.74516600	4.80415000	13.17000300
C	9.86918900	-0.61120500	12.81816100
H	9.39922500	-0.77816400	13.79787000
H	10.76963200	-1.23655500	12.79448700
C	9.32121900	10.12760500	5.31932500
H	9.40849900	10.07762900	4.23670300
C	3.70279400	0.89780000	7.77834200
H	3.86029700	1.73593600	7.08489600
H	2.99625100	0.21674700	7.28940600
C	5.43286100	5.93368100	7.95591000
H	6.19070100	5.98886100	7.17324100
H	4.79179700	6.81826100	7.86341900
H	4.81599900	5.04456100	7.78526000
C	5.46868200	4.31055900	14.84811400
H	5.99916900	4.98662200	15.52761200
H	4.62088500	4.88132600	14.44235600
C	8.98715900	1.76541300	12.63470200
H	9.27313500	2.81505500	12.54675000
H	8.44932100	1.66307600	13.58561900
C	4.81394900	5.96673200	10.95967400
H	4.14667700	5.10230700	10.91828100
H	4.21767500	6.86229900	10.74730600
H	5.21606200	6.06737900	11.97033100
C	10.38361400	5.68962900	13.83611800
H	8.08974300	8.14577800	4.14548100
H	6.90174500	7.95030900	5.45202000
H	8.39403900	7.03137700	5.48714800
H	7.18176700	3.72058400	8.74125300
C	10.68786800	5.18407900	9.19902300

C	9.33180600	4.80495700	7.31437700
C	10.63748700	5.37767200	6.74122600
C	11.58785400	5.45411400	7.96702900
H	10.84346100	5.87069900	10.02878600
H	10.82685800	4.16232000	9.56701200
H	9.36156200	3.71156400	7.34790300
H	8.43032400	5.10421600	6.77826900
H	11.03428300	4.75848800	5.93167500
H	10.46248600	6.37771000	6.33253000
H	12.39074000	4.71382900	7.90619400
H	12.06110700	6.43728000	8.03525900
N	9.27239800	5.30222300	8.71914500
H	9.04474000	6.29782500	8.66136500

INT4:

Rh	8.02734300	4.10573100	10.06699800
P	6.70687300	2.52039200	11.16998400
S	9.68082000	6.08767400	12.34559500
Si	6.48812500	5.73207800	9.52566600
O	7.34618700	7.17254200	9.85287100
O	9.04523300	4.77311000	11.93881700
O	8.83883800	6.92297500	13.20655400
O	10.39612100	6.75670400	11.23524500
N	7.64474700	8.36465600	7.89983000
F	11.87338000	4.65867100	12.72847800
F	11.74318600	6.44968600	13.96011600
C	8.69591300	10.58018700	8.02454300
C	7.48870900	-0.24039200	11.90354100
H	6.84594500	-0.27275700	12.79103900
H	6.88326500	-0.59432900	11.06493300
C	7.98993500	1.20607500	11.67621000
H	8.64280400	1.18102000	10.78935100

C	5.16660600	0.03879300	8.07821000
H	5.65455900	-0.34130100	7.17280600
H	4.91876700	-0.84014300	8.69063800
C	7.78297000	8.26626300	9.20210100
C	6.14399400	0.94211500	8.84962700
H	6.46103400	1.76974800	8.20457100
H	7.05174200	0.38014600	9.09384200
F	10.52863800	4.70675500	14.43870100
C	3.22685300	1.39934500	8.96427100
H	2.87624900	0.59293600	9.62404500
H	2.33997500	1.98173300	8.68767400
C	9.49514100	11.49040700	10.28611700
H	10.52050700	11.68986900	9.95455100
H	9.53199800	11.18919700	11.33512400
H	8.94581200	12.43684100	10.22132700
C	5.04832000	1.84499800	13.47683900
H	4.53031400	1.13840600	12.81869600
H	5.84047800	1.28126200	13.98498500
C	10.05763800	0.68291500	13.06069600
H	10.71535400	0.74413800	12.18164200
H	10.65678500	1.00952900	13.91859300
C	5.67657800	3.00609500	12.67431400
H	4.85172200	3.55847200	12.20560100
C	9.12689400	11.73690900	7.32830400
H	9.58990800	12.55063300	7.87679700
C	4.20813400	2.29458900	9.74250800
H	3.70554700	2.68407100	10.63385100
H	4.47466100	3.15967700	9.12362400
C	4.06375900	2.37793900	14.53443100
H	3.21490700	2.85639700	14.02534000
H	3.64993900	1.53902100	15.10725500
C	5.49092300	1.52522300	10.12243700

H	5.18717300	0.68346600	10.75855600
C	8.08685700	9.51222400	7.29346100
C	8.96016900	11.83346200	5.96355900
H	9.29053200	12.72257600	5.43425800
C	8.38695400	9.26404900	10.02091800
H	8.48274900	9.06781400	11.08263400
C	7.92069200	9.62636300	5.87836000
C	8.84687000	10.42072200	9.44513300
C	7.27537400	8.50659500	5.10362200
C	4.73228100	3.39308400	15.47194800
H	3.99853700	3.79210000	16.18242100
H	5.49880300	2.88004700	16.07025300
C	8.67310600	-1.20535100	12.10207200
H	8.29240500	-2.21830300	12.28093000
H	9.25431700	-1.25231000	11.16992900
C	6.37676500	4.00234700	13.62635000
H	7.21061800	3.50966500	14.13773400
H	6.81721700	4.83024600	13.06897400
C	9.59160700	-0.76644300	13.25030600
H	9.04534400	-0.84780000	14.20087100
H	10.45205100	-1.44177900	13.32706700
C	8.35744700	10.77804100	5.24733800
H	8.23044500	10.87093900	4.17145600
C	3.87667100	0.78698700	7.71647900
H	4.11260600	1.58759000	7.00135600
H	3.17269600	0.11491900	7.21169900
C	5.81670200	5.69058600	7.75913800
H	6.60579900	5.60527400	7.01092000
H	5.25918300	6.61022400	7.55392100
H	5.13862900	4.83761800	7.64115200
C	5.38881000	4.53181600	14.67930800
H	5.91227500	5.21824000	15.35414100

H	4.60786900	5.12296200	14.17945300
C	8.86914900	1.64042600	12.87240000
H	9.22405200	2.66551900	12.75120800
H	8.26145500	1.62311800	13.78604400
C	5.01368300	5.94469900	10.69176900
H	4.28938200	5.12975000	10.61376200
H	4.50279300	6.87381000	10.41042000
H	5.33097200	6.04368200	11.73232200
C	11.03934400	5.44068800	13.43933500
H	7.18276700	8.76864200	4.04585700
H	6.28013900	8.27444500	5.49527000
H	7.85765600	7.58154600	5.17819100
H	7.49897700	3.50836400	8.75381800
C	10.87023900	4.27266600	9.11497500
C	9.55339900	5.42580500	7.55354800
C	11.00891800	5.70996200	7.12733000
C	11.88294600	4.92894400	8.15002200
H	11.20313900	4.25438800	10.15262800
H	10.63156100	3.25087500	8.79757100
H	9.15194500	4.55169300	7.03206100
H	8.87824700	6.26870500	7.39900000
H	11.18931300	5.39602500	6.09535100
H	11.21921300	6.78208200	7.18096800
H	12.51814500	4.17805900	7.67195500
H	12.53742800	5.61054800	8.69977100
N	9.62470500	5.08889900	9.00225100
H	9.80086300	5.94602200	9.54044800

INT5:

Rh	8.53291400	3.79812800	9.99073300
P	6.91423200	2.53010100	11.10564000
S	9.90248300	6.08609500	12.14763100
Si	7.24205800	5.37994900	8.91832800
O	8.36794600	6.67890900	8.87302400
O	9.37013600	4.69701900	11.85634500
O	8.89284400	7.02155300	12.65125700
O	10.83400600	6.57994800	11.10641900
N	7.13813000	8.33156600	7.83710100
F	11.96305300	4.82496800	13.21891800
F	11.58408600	6.79892500	14.05669100
C	7.91029500	10.65625200	7.97709500
C	7.31085100	-0.13408500	12.33753600
H	6.55396000	0.02622100	13.11437600
H	6.79188200	-0.56863400	11.47884600
C	7.97997700	1.21310100	11.97559600
H	8.73554200	0.99573600	11.20404200
C	5.50168300	-0.25736000	8.22001000
H	6.04911600	-0.80339500	7.44251800
H	5.10716400	-1.01441300	8.91302400
C	8.16896100	7.98422000	8.57044200
C	6.46735900	0.67357400	8.97300400
H	6.93033700	1.36880100	8.26319000
H	7.28618500	0.08692000	9.40353300
F	10.29257000	5.12612800	14.58128000
C	3.60973300	1.36174400	8.66564800
H	3.11525200	0.68975800	9.38155600
H	2.81632400	1.96202700	8.20482700
C	10.02071900	11.22370400	9.31958400
H	10.52590200	11.75698300	8.50581300
H	10.78107400	10.72124900	9.92134100

H	9.53671900	11.97995400	9.94797200
C	4.91138700	2.38310400	13.22297600
H	4.41071300	1.62620400	12.60895900
H	5.56721200	1.84334500	13.91743200
C	9.78645600	0.75755500	13.70672700
H	10.55622600	0.62211700	12.93324300
H	10.29845200	1.16875800	14.58449300
C	5.75859500	3.34252600	12.35781800
H	5.06640700	3.88323400	11.69825200
C	7.69361600	12.00939300	7.61421600
H	8.38753200	12.77231500	7.95162200
C	4.57561200	2.28573700	9.42937200
H	4.01406100	2.83380800	10.19258600
H	4.98277300	3.03254500	8.73782000
C	3.85962400	3.15952100	14.03702600
H	3.13877200	3.61860800	13.34547500
H	3.28950100	2.46104500	14.66176500
C	5.73471200	1.48703900	10.06273100
H	5.28764300	0.77353900	10.76718200
C	6.98915400	9.65803500	7.52848000
C	6.60978500	12.35919500	6.83850200
H	6.44750600	13.39721300	6.56271800
C	9.13848200	8.89530300	9.07644500
H	9.93391700	8.50702400	9.70361500
C	5.86922800	10.03589600	6.72461000
C	9.01795600	10.23177000	8.78773800
C	4.89832000	8.98728300	6.24734100
C	4.50322300	4.25369300	14.90017200
H	3.72900000	4.82224600	15.42906500
H	5.12680900	3.78224900	15.67331600
C	8.35520500	-1.14458000	12.84924200
H	7.85408800	-2.08202100	13.11950200

H	9.04684800	-1.38738800	12.02976200
C	6.42922600	4.41716000	13.24252800
H	7.13440800	3.94760700	13.93678300
H	7.01807300	5.10688700	12.63744700
C	9.15351000	-0.59953300	14.04089800
H	8.48104500	-0.48094600	14.90247500
H	9.92273600	-1.32003500	14.34340100
C	5.70448700	11.37035200	6.39833100
H	4.85474400	11.66320600	5.78641900
C	4.33457200	0.52498500	7.60321300
H	4.72113400	1.19110400	6.81903700
H	3.63163900	-0.15887100	7.11300900
C	6.75542500	4.97479500	7.13612200
H	7.62331200	4.67617000	6.54147700
H	6.31689100	5.86903500	6.68238800
H	6.02619000	4.15949700	7.09070200
C	5.37307900	5.18988000	14.05008100
H	5.87343200	5.93166100	14.68239100
H	4.73080600	5.75436300	13.35886300
C	8.73662200	1.76406000	13.20705100
H	9.20674300	2.72450800	12.98508800
H	8.01720700	1.94533400	14.01561900
C	5.73636000	6.01713900	9.86431800
H	4.92133800	5.28835600	9.88430400
H	5.38012500	6.91573100	9.34967600
H	5.99254900	6.29234800	10.89064600
C	11.00272800	5.68879000	13.59324200
H	4.10368800	9.43785100	5.64589800
H	4.43811600	8.45824900	7.08848200
H	5.40214300	8.22631600	5.64198900
H	8.15859600	3.00870000	8.72821000
C	11.51863300	3.74100200	9.52459100

C	10.51900500	4.59805300	7.58562300
C	12.03827900	4.76938400	7.35829800
C	12.69835500	4.23568200	8.66194400
H	11.66211600	3.89221400	10.59413000
H	11.31245600	2.68037400	9.33787700
H	10.15927600	3.65752900	7.15850500
H	9.91838500	5.41479100	7.18588600
H	12.36745200	4.22289600	6.46999500
H	12.29017700	5.82212800	7.20161300
H	13.41978400	3.43609500	8.47247700
H	13.22654000	5.03888800	9.18292600
N	10.33774000	4.52500300	9.06035700
H	10.43870600	5.46963900	9.44945900

TS4:

Rh	0.90898600	0.38681500	-0.68432000
P	1.27038500	-1.42232000	0.59384400
S	2.29354300	3.11938200	-0.37118300
Si	-1.26041400	0.89728500	-0.02761000
O	-2.35582000	-0.39842300	-0.40150800
O	2.14083700	1.90566300	0.51849400
O	1.38014300	3.00686400	-1.54504000
O	3.67725600	3.52489400	-0.63736500
N	-4.36544000	0.65168900	0.05134600
F	2.24368300	4.59364900	1.81510900
F	1.65999800	5.65041200	0.00212800
C	-6.46172000	-0.48283300	-0.53424700
C	3.58654600	-3.25643300	0.59325800
H	3.44805400	-3.52477700	1.64754500
H	2.99313900	-3.96321400	0.00696400
C	3.12463900	-1.79834300	0.35343000
H	3.25092600	-1.59055900	-0.71764500

C	0.04461700	-4.77587300	-1.70060200
H	0.12346300	-4.95715800	-2.77918200
H	0.67791200	-5.52974800	-1.21093000
C	-3.70470600	-0.37676400	-0.42679600
C	0.57514700	-3.36946700	-1.37414200
H	0.00237500	-2.62361100	-1.93713100
H	1.61488100	-3.27838500	-1.70970500
F	0.28362500	4.24785600	0.93559000
C	-1.54638000	-4.61886200	0.26010500
H	-0.99681900	-5.36880400	0.84759700
H	-2.59561900	-4.69079500	0.57019500
C	-6.39440400	-2.78906200	-1.65167900
H	-7.05161000	-3.27715400	-0.92297700
H	-5.66480900	-3.52610800	-1.99564400
H	-7.01839000	-2.50547600	-2.50678300
C	1.33224300	-2.45903600	3.30660900
H	1.00218900	-3.41035200	2.87635200
H	2.42774500	-2.49485500	3.35106100
C	5.52132800	-1.00881800	0.74780700
H	5.67162900	-0.73490400	-0.30486300
H	6.13467500	-0.31617300	1.33568500
C	0.89180700	-1.25988400	2.43519700
H	-0.20685800	-1.24271400	2.42906600
C	-7.87875900	-0.44387900	-0.54328100
H	-8.43670300	-1.27934700	-0.95343600
C	-1.01110300	-3.21449800	0.59554300
H	-1.09646600	-3.05531400	1.67542400
H	-1.62856300	-2.45211500	0.11441300
C	0.78162100	-2.32678400	4.73882200
H	-0.31530900	-2.39060700	4.70831100
H	1.12588500	-3.17491000	5.34299200
C	0.45194200	-3.05690400	0.13253000

H	1.04417700	-3.80468400	0.67551300
C	-5.73366900	0.62257000	0.00846800
C	-8.54827100	0.64796100	-0.03563700
H	-9.63402500	0.67581700	-0.04417600
C	-4.33753500	-1.52216400	-0.99434400
H	-3.71630700	-2.32267600	-1.38163100
C	-6.44302300	1.74870200	0.53155200
C	-5.70683600	-1.58850100	-1.05273600
C	-5.68410700	2.91588100	1.10682500
C	1.19684500	-0.99941500	5.38749200
H	0.75133300	-0.91032900	6.38533900
H	2.28686900	-0.99352000	5.53067100
C	5.07019600	-3.43576700	0.21904200
H	5.37093500	-4.47137400	0.41889500
H	5.18591000	-3.28260500	-0.86352400
C	1.34836800	0.06733500	3.08132900
H	2.44078700	0.10775000	3.12404500
H	1.03710200	0.91536300	2.47096000
C	5.98034700	-2.45522800	0.96856300
H	5.95371100	-2.68551100	2.04332200
H	7.02124200	-2.58268600	0.64847400
C	-7.82618800	1.73672600	0.49790300
H	-8.37037300	2.59057200	0.89389100
C	-1.40601500	-4.94930800	-1.23179700
H	-2.05325300	-4.27953000	-1.81564800
H	-1.75234000	-5.97049300	-1.43086400
C	-1.63055700	1.44851200	1.74191300
H	-1.57422500	0.62661300	2.46099100
H	-2.64995400	1.84388200	1.76648000
H	-0.93597900	2.23242800	2.05621500
C	0.79470900	0.19472500	4.51086200
H	1.15020500	1.13133800	4.95590800

H	-0.30119400	0.26565800	4.47410300
C	4.04183500	-0.81890900	1.12339400
H	3.73624300	0.21590000	0.94940900
H	3.94033100	-1.00724100	2.19904700
C	-1.69700800	2.29048800	-1.23748100
H	-1.15300000	3.20538700	-0.99814000
H	-2.77392400	2.47336900	-1.15523700
H	-1.46912800	2.02483200	-2.27397200
C	1.57689900	4.49257500	0.66130100
H	-6.37021400	3.70007000	1.43873000
H	-4.99546200	3.34471200	0.37194000
H	-5.07209000	2.60881700	1.96156400
H	0.09767000	-0.55085200	-1.58135400
C	3.98670200	0.60960500	-2.58383400
C	2.18903200	-0.29672400	-3.76932300
C	3.26041300	0.02653100	-4.84600800
C	4.39308700	0.75062600	-4.06547400
H	4.32658800	1.43129100	-1.95086500
H	4.37169000	-0.32997700	-2.17175700
H	2.24956400	-1.34779400	-3.46732500
H	1.16385800	-0.10753500	-4.09594700
H	3.61498400	-0.88603100	-5.33469700
H	2.85080200	0.67080500	-5.62944100
H	5.38189800	0.32678500	-4.26421000
H	4.43327000	1.80977800	-4.33898500
N	2.50471000	0.54048700	-2.58709600
H	2.17066600	1.48992500	-2.76705000

INT6:

Rh	0.94941400	0.51667000	-0.62602600
P	1.25005900	-1.38343700	0.57686800
S	2.24971500	3.11536500	-0.55561900
Si	-1.25058600	1.04645400	-0.04766500
O	-2.32521700	-0.28721000	-0.39838900
O	2.33112300	2.07640700	0.52824000
O	1.21732700	2.64953000	-1.55847400
O	3.50825000	3.61195300	-1.10526000
N	-4.36009300	0.62936100	0.17665600
F	2.30849100	5.08805000	1.18878300
F	1.25260100	5.55581500	-0.64566000
C	-6.42715300	-0.53503200	-0.43754000
C	3.53361100	-3.25631100	0.57082000
H	3.35153200	-3.56441600	1.61430700
H	2.94391600	-3.93340800	-0.06365900
C	3.10291800	-1.78564100	0.37140200
H	3.25589300	-1.53583100	-0.69247000
C	-0.02591500	-4.60318000	-1.86322400
H	0.05253000	-4.73005700	-2.95614000
H	0.59351700	-5.40148100	-1.41335400
C	-3.66619100	-0.32332300	-0.39684000
C	0.53734100	-3.23257300	-1.46042600
H	-0.01085200	-2.43713900	-1.99210200
H	1.58680900	-3.15093500	-1.78622600
F	0.32339800	4.29225300	0.84753500
C	-1.61571000	-4.51620400	0.09945600
H	-1.08397900	-5.31495700	0.65002300
H	-2.67327400	-4.58193900	0.40548200
C	-6.30545100	-2.73748900	-1.74501400
H	-6.92667400	-3.31224800	-1.03825700
H	-5.55819400	-3.42032400	-2.17341800

H	-6.97148600	-2.40748300	-2.55934500
C	1.22988400	-2.46760800	3.27417000
H	0.88426000	-3.41018300	2.82181500
H	2.32940000	-2.53380900	3.33621200
C	5.50232300	-1.05811200	0.86595000
H	5.69792600	-0.75166500	-0.17653700
H	6.11260200	-0.39299000	1.49936500
C	0.83773000	-1.25010200	2.41021200
H	-0.26594500	-1.20850900	2.37470400
C	-7.84648300	-0.55227600	-0.40262200
H	-8.39053300	-1.37768800	-0.86522100
C	-1.04953300	-3.14777000	0.51301900
H	-1.13033200	-3.04870400	1.60639200
H	-1.65422800	-2.33916200	0.07966100
C	0.66024500	-2.34175600	4.69699400
H	-0.44346300	-2.38469800	4.64836200
H	0.97745200	-3.20892300	5.30074100
C	0.41341600	-2.98903700	0.05668400
H	0.99945400	-3.76975800	0.57004800
C	-5.72277900	0.55389000	0.17329900
C	-8.54190700	0.46905100	0.21221000
H	-9.63441800	0.45305800	0.23685800
C	-4.27559800	-1.44607000	-1.04028600
H	-3.63298400	-2.18968700	-1.51383600
C	-6.46014300	1.60681900	0.80819900
C	-5.64546800	-1.56511400	-1.06768600
C	-5.72452300	2.74927700	1.45511700
C	1.08822500	-1.03244300	5.36930200
H	0.62507000	-0.94602500	6.36655200
H	2.18146500	-1.05080700	5.53543900
C	5.02349700	-3.45377100	0.24324100
H	5.29990600	-4.50742900	0.41791300

H	5.18190100	-3.26748600	-0.83518900
C	1.30324800	0.05872800	3.08359500
H	2.40115900	0.07916100	3.14735500
H	1.02057400	0.92558400	2.47285000
C	5.92467800	-2.51788200	1.05385800
H	5.85657300	-2.78433600	2.12510500
H	6.98043800	-2.65708600	0.76647300
C	-7.84486100	1.54121900	0.81289200
H	-8.41228300	2.34210500	1.29569300
C	-1.47818300	-4.77085800	-1.40541700
H	-2.11470400	-4.05507000	-1.95727600
H	-1.84929900	-5.77764000	-1.66114000
C	-1.60173800	1.58179800	1.73100000
H	-1.55226400	0.74313600	2.44081700
H	-2.62382400	1.98768600	1.76385800
H	-0.89360800	2.35884700	2.05531600
C	0.72886100	0.18031000	4.50294600
H	1.09640000	1.10906600	4.97025700
H	-0.37053200	0.27723200	4.44828700
C	4.01599600	-0.84691900	1.19212900
H	3.73892900	0.20491300	1.03071400
H	3.87314000	-1.05450000	2.26551100
C	-1.81672300	2.40755600	-1.23310700
H	-1.31693200	3.36018400	-1.01094700
H	-2.90402200	2.53718300	-1.11248300
H	-1.60153000	2.15082800	-2.28124800
C	1.48025000	4.61565200	0.26396900
H	-6.42671600	3.48671300	1.87082800
H	-5.06492400	3.25925500	0.73547700
H	-5.07253000	2.39268900	2.26882300
H	0.09718100	-0.32368900	-1.57441300
C	4.23322000	0.58794700	-2.57320300

C	2.51667800	-0.57464400	-3.62421200
C	3.51064300	-0.22745200	-4.76376900
C	4.61023500	0.62332000	-4.07053300
H	4.50612500	1.49882800	-2.02417800
H	4.71760200	-0.27013800	-2.07822400
H	2.71034700	-1.59236400	-3.24589200
H	1.46012700	-0.53532000	-3.92461500
H	3.91616300	-1.13679400	-5.23376600
H	3.01155100	0.34705600	-5.55931900
H	5.62574900	0.23882100	-4.25157900
H	4.58885800	1.66119600	-4.43833500
N	2.77281600	0.38344100	-2.53498700
H	2.33266800	1.27269200	-2.78372200

INT7:

Rh	-0.48935700	0.40265800	0.07111900
P	-1.41644600	2.45010600	-0.28123900
S	-1.61938500	-2.16934800	0.36672300
Si	1.51522800	0.85392300	1.15676400
O	2.55538300	-0.28914100	0.42721600
O	-2.36225200	-0.86407000	0.52901400
O	-0.16793100	-1.81523300	0.11359200
O	-2.21396700	-3.17646800	-0.50390400
N	4.43461900	-0.01057800	1.73577200
F	-2.85819800	-3.30294100	2.39098900
F	-0.81134300	-3.98692400	2.08789300
C	6.25212800	-1.65028900	1.57447600
C	-4.17827100	3.09108800	-1.10070800
H	-4.19713200	4.07295200	-0.61290100
H	-3.79435100	3.24999500	-2.11265700
C	-3.28011700	2.11893900	-0.30141200
H	-3.32012100	1.14572600	-0.80987500

C	-1.13307600	2.52779100	-4.50476300
H	-1.33226700	1.72665700	-5.22588500
H	-1.85679000	3.32742700	-4.71913700
C	3.77335400	-0.71112200	0.84334200
C	-1.36334700	2.00139100	-3.07801800
H	-0.71332000	1.13549500	-2.89726300
H	-2.39312300	1.64083900	-2.98202600
F	-1.19197400	-2.03535700	2.97757700
C	0.61237000	4.13484200	-3.62807500
H	-0.02313600	5.01635700	-3.79498600
H	1.64939200	4.47544300	-3.73010400
C	6.00659500	-3.67249300	0.01101000
H	6.93042700	-3.50074200	-0.55410400
H	5.26588500	-4.10509400	-0.66486600
H	6.23779600	-4.41257700	0.78578100
C	-1.79307900	5.22061800	0.52034400
H	-1.73764600	5.47022900	-0.54462000
H	-2.85505700	5.08554600	0.75903800
C	-5.30041900	1.33595300	1.01673100
H	-5.26223100	0.34046700	0.55574800
H	-5.70696600	1.19829700	2.02563600
C	-1.02448500	3.91668800	0.82991000
H	0.03278100	4.09689300	0.59622700
C	7.54042600	-2.05088200	2.00925800
H	7.98572400	-2.95304200	1.60257400
C	0.38470300	3.60988800	-2.19929800
H	0.60310500	4.40898300	-1.48263000
H	1.09418900	2.79799300	-1.99647500
C	-1.24147700	6.39181700	1.35405200
H	-0.20623300	6.59593900	1.04649300
H	-1.81569900	7.30011500	1.13614200
C	-1.05476700	3.08309600	-2.01679400

H	-1.73984600	3.92624300	-2.17830000
C	5.67305300	-0.45880100	2.11398100
C	8.22720500	-1.30301300	2.94094900
H	9.21438600	-1.61340600	3.27119300
C	4.24977200	-1.91414200	0.25175600
H	3.61390900	-2.45228400	-0.44673200
C	6.39679000	0.30626300	3.08045800
C	5.48487900	-2.39239500	0.61312700
C	5.79341500	1.56356100	3.65088900
C	-1.27536100	6.08398400	2.85754400
H	-0.83335600	6.91166900	3.42420400
H	-2.32136000	6.00421900	3.18618100
C	-5.61554400	2.54299900	-1.18979200
H	-6.24426300	3.25417000	-1.73900000
H	-5.60621300	1.61511900	-1.77841000
C	-1.09650700	3.60071500	2.34021200
H	-2.13547000	3.41758600	2.63349000
H	-0.54682500	2.68248200	2.56313900
C	-6.21194800	2.25862500	0.19575100
H	-6.34445000	3.20863700	0.73351700
H	-7.21041000	1.81765800	0.09355100
C	7.65077500	-0.12930100	3.47104700
H	8.20551900	0.44966800	4.20549100
C	0.29127700	3.06924200	-4.68432300
H	1.00749500	2.24102100	-4.59234900
H	0.41824700	3.48234200	-5.69178400
C	2.34712800	2.54490700	1.04876900
H	2.42672500	2.91148000	0.02181900
H	3.35924300	2.41726700	1.44485100
H	1.83749700	3.30161600	1.65233100
C	-0.54519700	4.77075300	3.17341200
H	-0.62972400	4.53387100	4.24039000

H	0.52726300	4.89314700	2.96620500
C	-3.87083900	1.89448300	1.10911900
H	-3.24242500	1.21069000	1.68612900
H	-3.90101700	2.85034900	1.64750600
C	1.28611800	0.34909100	2.96618000
H	0.64505500	1.04540900	3.51530300
H	2.27295300	0.33311400	3.44129500
H	0.84816900	-0.64831900	3.04424400
C	-1.62055600	-2.92354400	2.07177300
H	6.46541200	2.01971000	4.38343600
H	4.83499900	1.35997400	4.13979200
H	5.58987800	2.29886800	2.86492100
H	0.64802000	0.89070300	-0.82675500
C	1.46208600	-4.49976100	-2.50693300
C	1.64854200	-5.13815600	-0.28100900
C	0.37819000	-5.87462800	-0.80057800
C	0.19369600	-5.35094000	-2.25186200
H	1.29637700	-3.66226600	-3.19139400
H	2.25818600	-5.12367900	-2.93633300
H	2.51259600	-5.81520100	-0.30517300
H	1.54521500	-4.77464100	0.74498400
H	0.50452900	-6.96171100	-0.76488900
H	-0.49394300	-5.62802200	-0.18891300
H	0.07923000	-6.15852900	-2.98243300
H	-0.69949500	-4.72185100	-2.31131300
N	1.90896600	-4.01079700	-1.19362100
H	1.30664400	-3.23602800	-0.91636000

TS5:

Rh	-0.47468200	0.45083300	-0.37401600
P	-1.52863500	2.44095900	-0.35119600
S	-1.65125100	-2.11612200	0.69468200
Si	1.51242300	0.91250300	0.74242500
O	2.60700600	-0.00368200	-0.23898400
O	-2.23187700	-0.77900000	0.28011200
O	-0.16962100	-2.06525000	0.62975000
O	-2.32695500	-3.29059000	0.13161100
N	4.53301800	0.10725200	1.01489900
F	-3.40018000	-2.20414000	2.67251300
F	-1.56332400	-3.28940400	3.06244500
C	6.51268200	-1.14510100	0.29656900
C	-4.27649000	3.06049300	-1.21547800
H	-4.34071000	3.99322400	-0.62950500
H	-3.86361600	3.33387600	-2.19673900
C	-3.37683100	2.03900300	-0.48349300
H	-3.35515600	1.11994500	-1.09537000
C	-1.15591100	3.51706300	-4.43348100
H	-1.25261400	2.88684300	-5.33328300
H	-1.97222600	4.26122800	-4.48770900
C	3.88380300	-0.35688300	-0.02444300
C	-1.33881900	2.65142600	-3.17811400
H	-0.58631100	1.84488800	-3.17286400
H	-2.31894900	2.15203700	-3.20907600
F	-1.58747900	-1.12426600	3.15679100
C	0.38313900	5.04801900	-3.13987600
H	-0.34714600	5.87811800	-3.11662200
H	1.38211300	5.51477800	-3.11959200
C	6.41479100	-2.56956600	-1.83436200
H	7.29971000	-2.10977500	-2.30474500
H	5.70819900	-2.84143300	-2.63095900

H	6.75647500	-3.49984000	-1.35121800
C	-2.08785400	4.93257200	1.02523800
H	-2.03117600	5.41642200	0.03753100
H	-3.14905900	4.68308000	1.19292600
C	-5.42289600	1.06722300	0.65615300
H	-5.33147500	0.12048100	0.09523900
H	-5.86468700	0.80732500	1.63266100
C	-1.24049700	3.64288800	1.06531800
H	-0.19149400	3.93589200	0.89210300
C	7.86582300	-1.49218900	0.54968400
H	8.38985700	-2.16318000	-0.13317600
C	0.19840000	4.17781800	-1.88622500
H	0.32657800	4.80346600	-0.98948300
H	0.99294100	3.41351300	-1.85623600
C	-1.63137400	5.92389700	2.10884500
H	-0.60615600	6.26600800	1.87606300
H	-2.27099500	6.82208000	2.08117900
C	-1.17375600	3.47690500	-1.88587500
H	-1.94789300	4.26369000	-1.87772200
C	5.83382800	-0.26442500	1.20122500
C	8.52208100	-0.98715400	1.65390500
H	9.56359100	-1.25723800	1.84548400
C	4.47009100	-1.23627000	-0.98536100
H	3.85829000	-1.57479400	-1.82284500
C	6.52918200	0.25154000	2.34325800
C	5.77660800	-1.63828700	-0.83769200
C	5.82006700	1.17574100	3.29639300
C	-1.65072900	5.29381900	3.50611100
H	-1.27259700	6.01080000	4.25377600
H	-2.69607400	5.07165600	3.79000500
C	-5.69118600	2.48956200	-1.41807400
H	-6.32261600	3.24332300	-1.91833300

H	-5.63104100	1.62779300	-2.10746400
C	-1.29311500	3.00356800	2.46807900
H	-2.32041700	2.68295000	2.69740900
H	-0.67804800	2.09313700	2.49527100
C	-6.33316200	2.03940100	-0.10147500
H	-6.52495200	2.92645400	0.53090100
H	-7.31662000	1.57922500	-0.29495100
C	7.85013900	-0.11900800	2.54287000
H	8.38515500	0.27143500	3.41341400
C	0.19394500	4.24203300	-4.42913600
H	1.00640900	3.49725700	-4.51396000
H	0.28187600	4.89982300	-5.30989800
C	2.26010700	2.63930700	0.89893400
H	2.29817100	3.18974300	-0.05133600
H	3.29297200	2.48859100	1.25108000
H	1.73617200	3.25550400	1.64536900
C	-0.83214600	3.99789000	3.54431300
H	-0.90345800	3.52657800	4.53857200
H	0.23656900	4.23629900	3.39131800
C	-4.02034900	1.65465800	0.86704900
H	-3.39350400	0.93026200	1.40130800
H	-4.10807500	2.55505600	1.49915200
C	1.48066500	0.09644500	2.44444900
H	0.72419000	0.53756000	3.11022900
H	2.47724300	0.22337800	2.89622800
H	1.26632000	-0.97601300	2.34506100
C	-2.07821900	-2.18972300	2.51724300
H	6.48475500	1.48215800	4.11742200
H	4.92876200	0.69486500	3.73023100
H	5.46067800	2.08174700	2.78234100
H	0.59945700	1.18850800	-1.19196500
Si	-1.58149300	-1.39021200	-3.61581300

H	-2.59393700	-0.41804900	-3.11965100
C	-0.76970200	-0.85188700	-5.21218700
H	-0.17505500	0.06545200	-5.07855300
H	-0.10540100	-1.64159700	-5.59778800
H	-1.53557400	-0.66113800	-5.98134000
O	-2.27806700	-2.85359900	-3.95438900
O	-0.46661300	-1.46092700	-2.35774900
C	0.68233700	-2.31779700	-2.39694700
H	1.35344000	-2.01685500	-1.58325400
H	0.39646000	-3.37144500	-2.24845600
H	1.21959000	-2.21387100	-3.35703100
C	-3.22748400	-3.57552900	-3.19032400
H	-4.25147000	-3.21184300	-3.39430400
H	-3.17968800	-4.63571200	-3.48662300
H	-3.03835400	-3.50642400	-2.10734200
C	-0.82619300	-6.77713800	-1.08183700
C	0.61762300	-5.57829300	0.27613100
C	-0.18854700	-6.45120700	1.26535200
C	-1.01571600	-7.38521700	0.34364600
H	-1.76526200	-6.70451000	-1.65190100
H	-0.14087000	-7.41346400	-1.67043500
H	1.56217000	-6.08450700	0.00087600
H	0.87228100	-4.58504300	0.67335300
H	0.44910800	-7.00106000	1.97598400
H	-0.85590800	-5.80321600	1.85567800
H	-0.65882200	-8.42700000	0.38360800
H	-2.07637400	-7.39876000	0.63915200
N	-0.22401700	-5.45240500	-0.91524900
H	-0.96184800	-4.77798600	-0.70194400

INT8:

Rh	-0.32357800	0.33428500	-0.64188400
P	-1.44849900	2.31446200	-0.43754000
S	-1.76369700	-2.22194700	1.05579600
Si	1.71371400	0.79353600	0.34401700
O	2.85233400	0.28963200	-0.85301300
O	-1.50398700	-0.73738800	0.90538600
O	-0.57254000	-3.01014200	1.41736500
O	-2.64455800	-2.77854700	0.01102500
N	4.79733500	0.40420300	0.38985900
F	-3.93695400	-1.52609100	2.40862100
F	-3.11584500	-3.46801200	2.94756700
C	6.92383100	-0.21226200	-0.66834100
C	-4.19420400	2.95149000	-1.32058200
H	-4.32006000	3.77283400	-0.60444300
H	-3.76350800	3.38697700	-2.22514200
C	-3.27858500	1.86324100	-0.70719200
H	-3.20405200	1.04785000	-1.44280700
C	-1.13068500	3.96405700	-4.33834500
H	-1.22866800	3.47914600	-5.31676700
H	-1.95807500	4.68434400	-4.26391600
C	4.19086600	0.14272400	-0.74388000
C	-1.26758000	2.91029100	-3.22609300
H	-0.49140300	2.14650300	-3.35150100
H	-2.22608900	2.39092100	-3.32435200
F	-2.12941900	-1.64659100	3.61481800
C	0.40195500	5.31687300	-2.84671800
H	-0.33924100	6.11313700	-2.68803500
H	1.38771900	5.79064500	-2.77013100
C	6.96010500	-0.95966900	-3.12060900
H	7.71729100	-0.23330900	-3.43687400
H	6.27043100	-1.11375500	-3.95376600

H	7.48099500	-1.90530600	-2.93258900
C	-2.21015000	4.56002900	1.25191700
H	-2.16959300	5.18078300	0.35000800
H	-3.24766500	4.22075500	1.35635700
C	-5.33160400	0.67349100	0.19944300
H	-5.18583900	-0.17839700	-0.47848100
H	-5.79065900	0.26764000	1.10785500
C	-1.27301000	3.33807200	1.12960100
H	-0.25040600	3.72447100	1.03290300
C	8.32630800	-0.36597800	-0.53022800
H	8.91533200	-0.70557100	-1.37580700
C	0.25292800	4.26018600	-1.73706700
H	0.38352200	4.74248800	-0.76279500
H	1.05214700	3.51594600	-1.83627900
C	-1.83786200	5.41186700	2.47969700
H	-0.83858400	5.84379500	2.32737900
H	-2.53207100	6.25642300	2.56474000
C	-1.11129200	3.54647200	-1.82577100
H	-1.88971500	4.31281300	-1.71339900
C	6.15529700	0.23510500	0.45225300
C	8.94226300	-0.08768300	0.67022800
H	10.01679300	-0.20706700	0.77360500
C	4.86494100	-0.30412300	-1.91709900
H	4.28229700	-0.49022300	-2.81309500
C	6.80860400	0.51971500	1.69158500
C	6.22522300	-0.48589700	-1.89275000
C	6.00575500	0.98925300	2.87664900
C	-1.84164500	4.58158700	3.77100800
H	-1.52947600	5.20109900	4.61986300
H	-2.86818800	4.25232400	3.98557800
C	-5.57617800	2.36373800	-1.66706300
H	-6.21228500	3.15464700	-2.08284600

H	-5.45265000	1.61413400	-2.46144100
C	-1.30645900	2.49281100	2.42313800
H	-2.30808700	2.07808600	2.57278000
H	-0.63562700	1.63383900	2.33515700
C	-6.25280300	1.71071800	-0.45454400
H	-6.50192100	2.48876500	0.28149900
H	-7.20194100	1.25077800	-0.75406600
C	8.17975700	0.35302900	1.77277100
H	8.68198100	0.56685600	2.71295600
C	0.20628600	4.71122700	-4.24292000
H	1.02677500	4.01008400	-4.45046900
H	0.26289300	5.49373800	-5.00866300
C	2.24865200	2.52883600	0.86121100
H	2.20697700	3.26201200	0.05387500
H	3.29112500	2.43711200	1.18332100
H	1.66452000	2.90006500	1.70789600
C	-0.93313700	3.35017400	3.64428000
H	-0.98974600	2.73892900	4.55232300
H	0.11225100	3.67945800	3.55546600
C	-3.96097200	1.27578700	0.54877800
H	-3.33141700	0.51630800	1.00887100
H	-4.10904300	2.07715600	1.28422200
C	1.81751900	-0.34758600	1.84581500
H	1.28396800	0.10784300	2.68706200
H	2.87092000	-0.46295700	2.12078800
H	1.37358600	-1.33070300	1.67822500
C	-2.79850400	-2.21197400	2.60295700
H	6.65248600	1.15488400	3.74269300
H	5.23838300	0.25864900	3.15222500
H	5.47859700	1.92335400	2.65569100
H	0.44466100	1.05673200	-1.75876300
Si	-0.32336400	-1.63455700	-3.12765800

H	-1.52106400	-0.76458200	-3.00746200
C	0.98577000	-1.00160800	-4.29835500
H	1.40947600	-0.05898700	-3.94100800
H	1.79588700	-1.73251900	-4.38966900
H	0.56757000	-0.84504800	-5.29781000
O	-0.72228300	-3.13919500	-3.64789100
O	0.25854400	-1.65755000	-1.52701900
C	1.30413400	-2.58946800	-1.15210600
H	1.15062000	-2.84939900	-0.10634600
H	1.21216400	-3.49350300	-1.76058800
H	2.28029200	-2.12530200	-1.30351900
C	-1.84321800	-3.92709800	-3.21488100
H	-2.29312900	-4.38154300	-4.10253800
H	-1.51443800	-4.71404700	-2.53021200
H	-2.59333200	-3.31563900	-2.70206000
C	-3.14792000	-6.63795600	-0.06081200
C	-0.96169700	-6.39395100	0.67059800
C	-1.78596200	-6.61504800	1.97202800
C	-3.25992600	-6.70019100	1.48431300
H	-3.99888000	-6.14275500	-0.53841500
H	-3.07973700	-7.65299900	-0.47646000
H	-0.52202400	-7.34336400	0.33584500
H	-0.15232800	-5.67205700	0.80015700
H	-1.47042900	-7.51847700	2.50448900
H	-1.65535000	-5.76979800	2.65321200
H	-3.77129000	-7.60470300	1.83038600
H	-3.82925800	-5.84088900	1.85244100
N	-1.89967300	-5.92256300	-0.36123900
H	-2.06624000	-4.92799000	-0.20701700

TS6:

Rh	-0.41326600	0.10442600	-0.38876600
P	-1.43267500	2.14759300	-0.47013500
S	-1.74358400	-2.10140400	1.88546800
Si	1.60076000	0.69532200	0.63763100
O	2.77732300	0.13576500	-0.51724200
O	-1.55018900	-0.67955100	1.41525200
O	-0.52365300	-2.77647600	2.35984700
O	-2.63391700	-2.90275000	1.01449500
N	4.72929300	0.36048600	0.70404100
F	-3.90039400	-1.16310600	3.09990900
F	-3.05890600	-2.95333400	4.00832300
C	6.86571000	-0.21526400	-0.35922500
C	-4.14062500	2.88416000	-1.39213700
H	-4.19278000	3.78172900	-0.76520600
H	-3.69627400	3.19086400	-2.34245300
C	-3.29183700	1.79681400	-0.68795300
H	-3.27515400	0.91972300	-1.34921000
C	-1.00027700	3.22655500	-4.55396600
H	-1.14134800	2.61961800	-5.45625300
H	-1.75818700	4.02238800	-4.59265300
C	4.11712200	0.04213300	-0.41324100
C	-1.23360200	2.35596700	-3.30758600
H	-0.53489900	1.51144100	-3.31627500
H	-2.23809300	1.92208700	-3.34356200
F	-2.07757100	-1.03018500	4.28220200
C	0.64171400	4.63838300	-3.24831600
H	-0.02907100	5.50902800	-3.21802700
H	1.66430400	5.03328800	-3.22499100
C	6.90204600	-1.03773800	-2.78735400
H	7.62802600	-0.29443500	-3.13603000
H	6.20902500	-1.24346300	-3.60661900

H	7.46043300	-1.95694700	-2.57662100
C	-2.07966500	4.64142100	0.89863800
H	-2.00615900	5.13059200	-0.07903200
H	-3.13431200	4.37906000	1.04340700
C	-5.41476700	0.85457000	0.35658000
H	-5.36529600	-0.06147700	-0.24688600
H	-5.88067100	0.57149800	1.30750900
C	-1.21176500	3.36436000	0.95073900
H	-0.17060400	3.68052800	0.81813500
C	8.27404800	-0.31456900	-0.23233500
H	8.86622800	-0.66001500	-1.07350100
C	0.39933800	3.76963900	-2.00085500
H	0.55939100	4.37923500	-1.10553200
H	1.13741900	2.95894300	-1.97315700
C	-1.66185100	5.63069100	2.00224800
H	-0.63646800	5.97560600	1.80768300
H	-2.30267000	6.51975600	1.96065700
C	-1.01825600	3.16179700	-2.00588700
H	-1.73296700	3.99548500	-2.00048900
C	6.09243000	0.24080000	0.75451300
C	8.89229800	0.02441600	0.95141900
H	9.97155400	-0.05295400	1.04611800
C	4.79826900	-0.41916400	-1.57923200
H	4.21338100	-0.65305000	-2.46270800
C	6.74858300	0.58849500	1.97657300
C	6.16366700	-0.55232800	-1.56586400
C	5.94093900	1.06706600	3.15470200
C	-1.72716100	4.98635000	3.39395000
H	-1.38572900	5.69607100	4.15671900
H	-2.77390900	4.75047100	3.63321300
C	-5.57072000	2.37494500	-1.65669000
H	-6.15467800	3.16754600	-2.14037200

H	-5.52601900	1.54151200	-2.37222000
C	-1.30166500	2.70616600	2.34711100
H	-2.32574800	2.37081000	2.53720400
H	-0.68149800	1.80689100	2.38771600
C	-6.26424300	1.90336100	-0.37182600
H	-6.42170500	2.76660100	0.29080400
H	-7.25866900	1.50291600	-0.60260800
C	8.12563500	0.47346200	2.04770100
H	8.62925300	0.73588000	2.97483800
C	0.39878600	3.85651400	-4.54580300
H	1.15275000	3.06195000	-4.63540000
H	0.52911100	4.51143300	-5.41549100
C	2.15899000	2.46613300	1.01293100
H	2.09433600	3.14587700	0.16117400
H	3.21216100	2.38726000	1.30196900
H	1.61287200	2.89836400	1.85542600
C	-0.89307400	3.69838700	3.44956300
H	-0.99660100	3.21844900	4.42961600
H	0.17038800	3.95390900	3.33960400
C	-3.98829200	1.36350000	0.62260600
H	-3.40686900	0.59532300	1.13403800
H	-4.05417100	2.22701200	1.29652800
C	1.77724100	-0.29548600	2.23553800
H	1.20603800	0.19642400	3.03082100
H	2.83352300	-0.31227700	2.52168700
H	1.39706400	-1.31553300	2.16080900
C	-2.75569200	-1.79316000	3.41507500
H	6.59029500	1.29034400	4.00586000
H	5.20772700	0.31581700	3.46560300
H	5.37167400	1.96907600	2.90721400
H	0.39125500	0.59363900	-1.59574200
Si	-1.18270900	-2.40753500	-1.94771600

H	-1.57346900	-0.92082600	-1.92722300
C	-0.28870900	-2.78769000	-3.55556000
H	0.55080500	-2.09676300	-3.68551500
H	0.09917200	-3.80857300	-3.60254500
H	-0.97734300	-2.65190800	-4.39499700
O	-2.76264000	-2.97705800	-2.03420000
O	0.01431000	-2.06555800	-0.74413300
C	1.28719800	-2.72320900	-0.64018100
H	1.43296200	-3.03893800	0.39430800
H	1.32089700	-3.59369000	-1.29725500
H	2.06582400	-2.01732900	-0.92780700
C	-3.93822800	-2.21895500	-1.80905200
H	-3.82806700	-1.18022200	-2.14772900
H	-4.75085900	-2.67939800	-2.38111000
H	-4.19320600	-2.22304100	-0.74502500
C	-1.58188900	-5.70340600	-1.97087600
C	-0.28048200	-5.36275000	-0.05669800
C	-1.00326800	-6.69835100	0.19898100
C	-1.71728500	-7.01034400	-1.14440900
H	-2.49932000	-5.39100500	-2.46875800
H	-0.80203900	-5.81318900	-2.73328300
H	0.69467000	-5.54153300	-0.52334100
H	-0.14133000	-4.75868100	0.84087400
H	-0.31391700	-7.48908700	0.50920100
H	-1.73522100	-6.56968600	1.00277800
H	-1.25550900	-7.85335200	-1.66745200
H	-2.76740400	-7.27029100	-0.98229000
N	-1.15108300	-4.64173200	-1.02351300
H	-1.97157800	-4.34810800	-0.48796500

INT9:

Rh	-0.43528400	-0.05868700	-0.32633600
P	-1.41826800	2.00569500	-0.46170100
S	-1.88633700	-2.07297500	2.04696200
Si	1.59406600	0.54553900	0.67893000
O	2.76724500	0.24653900	-0.57697900
O	-1.48075700	-0.68927300	1.62047600
O	-0.82250900	-2.88887300	2.65291200
O	-2.74231800	-2.76799200	1.04773200
N	4.73948000	0.35857900	0.62709000
F	-4.09887900	-0.96780200	2.99975900
F	-3.51451700	-2.81979600	3.98114900
C	6.86954500	0.07171000	-0.55974000
C	-4.04398300	2.86334600	-1.50924500
H	-4.07748100	3.75779800	-0.87735300
H	-3.54787400	3.15545300	-2.43853600
C	-3.27445800	1.73053000	-0.78661900
H	-3.25800400	0.86362300	-1.46070100
C	-0.72131200	3.11349500	-4.50440900
H	-0.84467900	2.52680700	-5.42264500
H	-1.43475200	3.94777800	-4.56988100
C	4.11045800	0.20173800	-0.51556800
C	-1.06339400	2.23945600	-3.28592600
H	-0.40599300	1.36246700	-3.27252000
H	-2.08439800	1.85573300	-3.38136600
F	-2.42582000	-0.98000800	4.39154300
C	0.91634400	4.42365800	-3.09236900
H	0.28951600	5.32702400	-3.08712400
H	1.95471800	4.76570000	-3.00856100
C	6.87535700	-0.33402700	-3.09138800
H	7.55735700	0.49124900	-3.32582000
H	6.16976900	-0.43487500	-3.91954800

H	7.48019700	-1.24700300	-3.04814200
C	-2.06092900	4.52402600	0.87498400
H	-1.92532200	5.00592000	-0.09958000
H	-3.12914300	4.29638400	0.97013500
C	-5.48591400	0.88302300	0.16536200
H	-5.47307000	-0.02482000	-0.45132800
H	-5.99824000	0.61546900	1.09680100
C	-1.23844900	3.21971300	0.97357100
H	-0.18235100	3.50191600	0.89618900
C	8.28383200	0.02112800	-0.47910100
H	8.86738200	-0.15057300	-1.37780600
C	0.56265800	3.55588500	-1.87097800
H	0.69769100	4.15210400	-0.96269200
H	1.26070600	2.71243100	-1.80667500
C	-1.66870800	5.50658100	1.99369000
H	-0.62350300	5.81622500	1.85254100
H	-2.27664600	6.41601300	1.91381400
C	-0.87994000	3.01608700	-1.96200800
H	-1.55488200	3.88203800	-1.98327300
C	6.10730900	0.29837400	0.62944300
C	8.91891300	0.18848000	0.73219200
H	10.00277500	0.14909300	0.79124700
C	4.78047300	-0.02681500	-1.75533100
H	4.18269400	-0.13973100	-2.65388900
C	6.78113200	0.47025100	1.87912900
C	6.15004300	-0.09404700	-1.79160100
C	5.98585300	0.70887700	3.13605400
C	-1.82951900	4.87274100	3.38223800
H	-1.50750000	5.57529200	4.16006900
H	-2.89471300	4.67109100	3.56507600
C	-5.48664800	2.42951900	-1.83170200
H	-6.01117100	3.25365800	-2.33061000

H	-5.45915600	1.59792500	-2.55022500
C	-1.41808500	2.57333500	2.36721700
H	-2.45898900	2.26612600	2.50773100
H	-0.82539700	1.65898600	2.44451100
C	-6.24853300	1.98848200	-0.57517500
H	-6.37593100	2.85327700	0.09196200
H	-7.25688500	1.64933700	-0.84117300
C	8.16341400	0.41188000	1.90289300
H	8.68015000	0.54160200	2.85067400
C	0.70549100	3.67017800	-4.41203900
H	1.42151800	2.83855500	-4.47184900
H	0.91649000	4.32734100	-5.26414100
C	2.08127600	2.26013500	1.32402600
H	2.02887700	3.04859800	0.56989500
H	3.12513500	2.17371200	1.64305800
H	1.48597900	2.55686100	2.19122400
C	-1.03996100	3.56036600	3.48562800
H	-1.20916400	3.08847700	4.46056500
H	0.03463400	3.78450100	3.43115700
C	-4.04189700	1.30406600	0.48503400
H	-3.51781100	0.49607000	0.99777200
H	-4.08286700	2.15119800	1.18068100
C	1.86982900	-0.66332700	2.10955400
H	1.35354800	-0.28167100	2.99731900
H	2.94046800	-0.72676000	2.32643400
H	1.46862800	-1.66088200	1.92433900
C	-3.05317600	-1.68527900	3.44291600
H	6.64765700	0.81660900	4.00002500
H	5.29223800	-0.11544600	3.33080700
H	5.37349800	1.61286200	3.05382900
H	0.31829700	0.36391100	-1.58426300
Si	-1.29945600	-2.59628700	-1.72541600

H	-1.71367900	-1.11757000	-1.47585900
C	-0.46947600	-2.80711100	-3.40976000
H	0.37684300	-2.12422800	-3.52804000
H	-0.10265000	-3.82456300	-3.58511600
H	-1.20103900	-2.59115900	-4.19352300
O	-2.93442000	-3.02762600	-2.04038600
O	0.09840600	-2.18469700	-0.67153800
C	1.43292500	-2.62923600	-0.94078700
H	1.92844800	-2.87534800	0.00241500
H	1.42898700	-3.51793400	-1.57838200
H	1.99529100	-1.83832300	-1.43936300
C	-4.01081500	-2.11858800	-2.03702300
H	-3.84187000	-1.25880200	-2.70304200
H	-4.90449300	-2.64372200	-2.39368600
H	-4.20812900	-1.74587700	-1.02478900
C	-1.83438900	-5.58575500	-1.61702600
C	-0.28244600	-5.00886900	0.06957300
C	-0.89110200	-6.33946000	0.52646500
C	-1.71009600	-6.82677600	-0.69769500
H	-2.84212600	-5.37077800	-1.96373500
H	-1.18292100	-5.67397800	-2.49101300
H	0.60926500	-5.17944300	-0.53824500
H	-0.05207200	-4.32271500	0.88336700
H	-0.12526000	-7.05373000	0.83998000
H	-1.54515500	-6.16191700	1.38605900
H	-1.20400300	-7.64173400	-1.22326100
H	-2.69287600	-7.19953400	-0.39682800
N	-1.34776700	-4.42029800	-0.80328100
H	-2.10456700	-4.15666100	-0.15502700

TS7:

Rh	-0.54012600	-0.16451300	-0.20439100
P	-1.36737200	1.92361400	-0.35419400
S	-2.01954200	-1.95116700	2.38754100
Si	1.54334000	0.36845500	0.65122200
O	2.61463300	0.54278400	-0.70521600
O	-1.35184200	-0.72045000	1.83813700
O	-1.11232300	-2.89962300	3.04660300
O	-3.02097900	-2.53923300	1.45791800
N	4.66937900	0.51683200	0.33355700
F	-3.98131800	-0.39883200	3.28176900
F	-3.67441000	-2.21329000	4.42448300
C	6.70318900	0.70962800	-1.02017900
C	-3.94191200	2.82772400	-1.49881400
H	-4.13689500	3.58443800	-0.72246700
H	-3.36279100	3.33025700	-2.28770800
C	-3.16255600	1.63833000	-0.89463900
H	-3.01236700	0.91071200	-1.71104900
C	-0.28988400	3.30671800	-4.23081100
H	-0.33576200	2.79078100	-5.20453500
H	-0.98676000	4.16276700	-4.30078500
C	3.95236700	0.59335400	-0.76110200
C	-0.76139100	2.35271200	-3.12270800
H	-0.12932300	1.44924600	-3.12416600
H	-1.78508900	2.01136800	-3.34133300
F	-2.28742300	-0.55967800	4.62197100
C	1.22315000	4.47402700	-2.57990800
H	0.60642400	5.39199200	-2.56269300
H	2.25870800	4.79274800	-2.37450400
C	6.52079700	0.93332200	-3.56877600
H	7.13193900	1.84861600	-3.63526500
H	5.75458500	0.97826700	-4.35545300

H	7.18991100	0.08621300	-3.79307900
C	-2.08914500	4.39669300	1.01641300
H	-1.83088100	4.91543300	0.07988500
H	-3.17304200	4.19903000	0.98011900
C	-5.36140400	0.46610800	-0.40225200
H	-5.17489600	-0.31573500	-1.16093300
H	-5.95651700	-0.01206900	0.39297800
C	-1.32807800	3.06082800	1.15341400
H	-0.25460700	3.30346200	1.21201200
C	8.12177300	0.75956800	-1.04765500
H	8.64220600	0.86511900	-2.00116300
C	0.75070100	3.52258400	-1.46892600
H	0.80886700	4.04523400	-0.50149200
H	1.43642800	2.66178000	-1.41082500
C	-1.80114600	5.32014100	2.21172900
H	-0.73152800	5.59980800	2.20489500
H	-2.36796400	6.25970900	2.09746800
C	-0.68080000	3.01350700	-1.73125800
H	-1.35035900	3.89143200	-1.73911900
C	6.03037900	0.57065300	0.23814900
C	8.84646200	0.67468400	0.12397400
H	9.93836300	0.71301200	0.09882300
C	4.52700900	0.73032500	-2.06309100
H	3.85700900	0.78022700	-2.92336400
C	6.79737500	0.48366700	1.44595200
C	5.89308900	0.79038900	-2.20711900
C	6.09497500	0.33766500	2.76896700
C	-2.14255300	4.64429400	3.54426700
H	-1.88622400	5.30762100	4.38738100
H	-3.23511300	4.48179400	3.59788900
C	-5.28589600	2.35695700	-2.08111800
H	-5.83435100	3.22338500	-2.48811200

H	-5.09282100	1.68342600	-2.93643000
C	-1.69505100	2.37298000	2.48451900
H	-2.76203100	2.09806100	2.48239100
H	-1.14493800	1.43090900	2.59735500
C	-6.13831500	1.62278000	-1.03974000
H	-6.44024700	2.33666100	-0.25088800
H	-7.07185800	1.25663400	-1.49920700
C	8.18023100	0.53718600	1.36206900
H	8.77034400	0.47071000	2.28063000
C	1.12643500	3.82705800	-3.96521700
H	1.83950200	2.98438900	-4.02234100
H	1.42694400	4.54443700	-4.74731500
C	1.92333200	1.83452800	1.78225900
H	1.86517400	2.80862700	1.27644100
H	2.96138800	1.70007300	2.12543800
H	1.26194100	1.84594300	2.66142100
C	-1.42401400	3.29769500	3.68091800
H	-1.72906600	2.78766400	4.60971600
H	-0.33594700	3.47375700	3.76714400
C	-4.01819200	0.93142200	0.17744000
H	-3.48417100	0.07556700	0.61011900
H	-4.21396800	1.63103100	1.00763700
C	1.96544400	-1.21227500	1.59546400
H	1.40932100	-1.23550400	2.54488400
H	3.04771800	-1.21205300	1.80095500
H	1.70203900	-2.11449300	1.02844100
C	-3.05810900	-1.23426300	3.76858700
H	6.81797600	0.27550100	3.59540500
H	5.46252900	-0.56402700	2.78906500
H	5.42064500	1.18801900	2.95930300
H	0.07026400	0.17380000	-1.56176500
Si	-0.73156100	-3.12427400	-1.83590500

H	-1.26278900	-1.84138700	-0.99647600
C	-0.10329000	-4.64115700	-2.82877300
H	0.53249300	-4.28052100	-3.65424000
H	0.50125600	-5.36220100	-2.25516900
H	-0.93583000	-5.18104400	-3.30401300
O	-1.77204200	-2.68939600	-3.11228900
O	0.79583400	-2.58193000	-1.24802100
C	2.08955100	-3.01924900	-1.60697000
H	2.82494800	-2.39112700	-1.08147500
H	2.27417400	-4.07076500	-1.32286300
H	2.27744100	-2.92052100	-2.69022600
C	-2.66263500	-1.62281700	-3.23408700
H	-2.42414400	-1.01842400	-4.12858200
H	-3.69976400	-1.99159400	-3.34369500
H	-2.63129300	-0.95455200	-2.35339700
C	-2.73450600	-5.15351600	-0.89730200
C	-0.84029900	-4.98075900	0.49188200
C	-1.84145500	-5.84573600	1.27984600
C	-3.10204700	-5.93738100	0.37587100
H	-3.57141000	-4.60711900	-1.35265300
H	-2.29546200	-5.80723300	-1.66313600
H	-0.14849200	-5.59149000	-0.10508900
H	-0.26303200	-4.30041400	1.12941000
H	-1.41376200	-6.83162600	1.51367000
H	-2.07811500	-5.34916300	2.23056300
H	-3.38106200	-6.97355400	0.13401900
H	-3.96374200	-5.47018700	0.87427000
N	-1.68661300	-4.18798600	-0.45452200
H	-2.18086200	-3.48929600	0.13280400

INT10:

Rh	-0.69182200	0.43013500	-0.37705400
P	-1.87414100	2.57671600	-0.25104900
S	-2.45289300	-2.12325600	0.78064300
Si	1.34479400	0.48271300	0.60183400
O	2.50063600	-0.48666100	-0.32103700
O	-2.43083300	-0.63563600	0.59986000
O	-1.61591700	-2.65816300	1.86528000
O	-2.37414200	-2.89325600	-0.50011800
N	4.36080000	-0.36750000	1.05207300
F	-5.05699500	-1.99850900	0.34847700
F	-4.42397500	-3.68390300	1.57162400
C	6.49741000	-1.25901200	0.23064700
C	-4.69430800	3.42617900	-0.63777100
H	-4.59508800	4.33735400	-0.03496300
H	-4.43323800	3.70181600	-1.66400800
C	-3.75323500	2.32904500	-0.09237300
H	-3.90234600	1.43345900	-0.71318100
C	-2.03930700	3.34952100	-4.40667700
H	-2.31784000	2.66549000	-5.21778700
H	-2.76515700	4.17530600	-4.43333600
C	3.80281300	-0.70358700	-0.09087000
C	-2.14364200	2.62193900	-3.05598200
H	-1.49390700	1.73640900	-3.06341700
H	-3.16675300	2.25463700	-2.91639100
F	-4.47134400	-1.67907700	2.42084000
C	-0.19924700	4.80935900	-3.47190000
H	-0.83804100	5.70431300	-3.45308000
H	0.82767300	5.16457000	-3.62172700
C	6.63955900	-2.26358400	-2.12341000
H	7.47091100	-1.63042500	-2.45351900
H	5.99846300	-2.45401200	-2.98792800

H	7.07272900	-3.21873100	-1.80459800
C	-2.06605500	5.18166200	1.09111700
H	-2.15588900	5.62642900	0.09367300
H	-3.08806800	5.01012900	1.45185400
C	-5.64526500	1.45658900	1.36696500
H	-5.72902700	0.53093100	0.78338600
H	-5.92954900	1.20032600	2.39482500
C	-1.30823700	3.83910400	1.03270700
H	-0.28778100	4.05370400	0.68496800
C	7.86904400	-1.50212700	0.49282600
H	8.48533300	-1.97698300	-0.26395400
C	-0.30181300	4.08388900	-2.11861600
H	-0.01639900	4.77724300	-1.32010800
H	0.41767200	3.25617100	-2.09020100
C	-1.36526400	6.17270900	2.03866100
H	-0.37941200	6.42906900	1.62510000
H	-1.93562400	7.10865100	2.08601900
C	-1.72092700	3.52686300	-1.87591500
H	-2.40981300	4.38111300	-1.83258200
C	5.69101400	-0.63144000	1.23217700
C	8.42231700	-1.13796600	1.70115000
H	9.47377200	-1.32479600	1.89968600
C	4.52766600	-1.33086700	-1.15274200
H	3.99867700	-1.56173300	-2.07190100
C	6.28098400	-0.25913200	2.48128600
C	5.86222400	-1.61090000	-1.00812600
C	5.43896200	0.40154800	3.54117800
C	-1.18705700	5.58278100	3.44506800
H	-0.64418900	6.28744000	4.08660300
H	-2.17619700	5.44145700	3.90405700
C	-6.16156500	2.95740600	-0.60632700
H	-6.81341800	3.75883500	-0.97611500

H	-6.28003300	2.11400600	-1.30157500
C	-1.16021300	3.24247300	2.45024500
H	-2.14671100	3.00236700	2.86151100
H	-0.60803600	2.29913100	2.40309100
C	-6.59703200	2.51756000	0.79832500
H	-6.60183200	3.39281900	1.46424600
H	-7.62637800	2.13916800	0.77520200
C	7.62439100	-0.51892500	2.68693200
H	8.07647800	-0.23730000	3.63485100
C	-0.62928400	3.90895800	-4.63760600
H	0.07992600	3.07414700	-4.72998300
H	-0.58681800	4.46241600	-5.58370400
C	2.21288500	2.16865400	0.72092300
H	2.33158400	2.62422600	-0.26741900
H	3.20399500	2.04849900	1.16905300
H	1.63841600	2.86207200	1.34333000
C	-0.45720800	4.23233300	3.39488300
H	-0.38714800	3.79964100	4.40013800
H	0.57516800	4.39145700	3.05207700
C	-4.18437100	1.93679700	1.33951800
H	-3.53144700	1.15528300	1.73414900
H	-4.09334800	2.81060700	1.99775900
C	1.24679800	-0.28660300	2.33489100
H	0.75186100	0.40492900	3.02625100
H	2.25501200	-0.48713100	2.71030900
H	0.66402300	-1.21170100	2.33242400
C	-4.21288100	-2.38671000	1.31589200
H	6.03806400	0.63041200	4.42714800
H	4.60395800	-0.23954400	3.84212100
H	4.99295300	1.33065800	3.17219900
H	0.28505000	1.05872600	-1.39000600
Si	-0.06365700	-4.07498900	-3.17842000

H	0.02033600	-0.96197300	-0.65616200
C	1.28751800	-5.21215900	-3.76771700
H	1.18295600	-5.34524900	-4.84892500
H	2.28645300	-4.80896100	-3.57646600
H	1.22626500	-6.20226200	-3.30652900
O	-1.51317600	-4.68217900	-3.60754000
O	0.06139200	-2.52571800	-3.65016500
C	1.14109900	-1.58571300	-3.63423200
H	1.18991800	-1.08291300	-2.66544500
H	2.09642600	-2.07659200	-3.85879500
H	0.94074200	-0.84554900	-4.41212400
C	-2.76958400	-3.98266400	-3.65172500
H	-2.70393000	-3.12522100	-4.32756200
H	-3.51458000	-4.68567000	-4.03050300
H	-3.05781200	-3.63736900	-2.65559800
C	-0.46942800	-5.45655200	-0.70475400
C	1.25334200	-3.71347000	-0.63320600
C	1.16133200	-4.37715000	0.73907100
C	0.54233800	-5.74686900	0.41819700
H	-1.48502800	-5.31396800	-0.33528900
H	-0.48661800	-6.22549800	-1.47865500
H	2.08523300	-4.12430300	-1.21385300
H	1.32529700	-2.62506500	-0.60932100
H	2.14013700	-4.44990900	1.21941200
H	0.49570800	-3.79685200	1.38466100
H	1.31169100	-6.44257300	0.06476900
H	0.05291300	-6.20228000	1.28223800
N	-0.04538200	-4.11531000	-1.31095500
H	-0.78697200	-3.44494400	-0.99594900

TS8:

Rh	-0.69486400	-0.08618300	-0.02107100
P	-1.76076400	2.03739600	-0.30179500
S	-2.40997600	-2.06402800	1.94964800
Si	1.40708000	0.29360600	0.84295500
O	2.56745200	-0.53721500	-0.16762900
O	-1.96557900	-0.62969500	1.71187500
O	-1.30831700	-3.00073100	2.22318400
O	-3.44608700	-2.50702900	0.99857800
N	4.47849100	-0.20963200	1.07790500
F	-4.31311700	-1.01130800	3.47127900
F	-3.78221800	-3.05175000	3.96892200
C	6.62643600	-1.03753200	0.23888000
C	-4.48175000	2.76156100	-1.20624700
H	-4.50581700	3.71804400	-0.65749800
H	-4.05226500	2.97965800	-2.19434600
C	-3.63299900	1.73445600	-0.42374300
H	-3.66353600	0.78782800	-0.99445000
C	-1.38985800	2.67301500	-4.48054500
H	-1.57810000	1.97396900	-5.31241000
H	-2.11207800	3.50208200	-4.60100900
C	3.89223800	-0.67395700	0.00194400
C	-1.64793300	1.95975700	-3.14436500
H	-1.00793600	1.06413600	-3.07888000
H	-2.68692200	1.59668600	-3.12222900
F	-2.46431100	-1.42435800	4.52280000
C	0.34351000	4.13078900	-3.36074000
H	-0.28614800	5.03831100	-3.41479300
H	1.38980000	4.47829900	-3.39683300
C	6.72929100	-2.24148500	-2.02251300
H	7.51353700	-1.59292800	-2.44668800
H	6.06453500	-2.55031000	-2.84165400

H	7.23474400	-3.14206700	-1.63638500
C	-2.22330200	4.67408900	0.86236000
H	-2.16478800	5.07725400	-0.16148400
H	-3.29001400	4.47758000	1.06286000
C	-5.73047900	0.92244500	0.75433900
H	-5.68808300	-0.06039900	0.25150800
H	-6.18261700	0.73787100	1.74276500
C	-1.42408900	3.36171400	0.98855400
H	-0.36711400	3.60228600	0.77973600
C	8.02300600	-1.17538700	0.45460100
H	8.64009900	-1.68003200	-0.29086200
C	0.08484500	3.41424200	-2.02602000
H	0.30147900	4.10488900	-1.19603900
H	0.78474500	2.56760800	-1.92655100
C	-1.71640600	5.72678100	1.86206900
H	-0.68208800	6.01151900	1.59409600
H	-2.32239100	6.64450300	1.77500100
C	-1.35812700	2.88068100	-1.93938100
H	-2.03717500	3.74919700	-1.99945700
C	5.82574600	-0.37344800	1.22515000
C	8.60438300	-0.67514100	1.60190900
H	9.67954800	-0.78313100	1.76528300
C	4.60346400	-1.34717100	-1.04007400
H	4.04874300	-1.69970200	-1.91099700
C	6.44478600	0.14020800	2.41168000
C	5.96189100	-1.53522000	-0.93588400
C	5.60805500	0.83457200	3.45216300
C	-1.74066200	5.20235900	3.30269400
H	-1.32973700	5.95901900	3.99198800
H	-2.78984500	5.03866100	3.61125400
C	-5.92090400	2.24908600	-1.39233800
H	-6.51399800	3.00567100	-1.93392600

H	-5.89905300	1.35256200	-2.03930500
C	-1.47311500	2.82833200	2.43658100
H	-2.50569700	2.55327300	2.70090700
H	-0.88841300	1.90062100	2.52184200
C	-6.59061800	1.89424400	-0.05989500
H	-6.74438800	2.82058700	0.52477000
H	-7.59374400	1.47137400	-0.23815600
C	7.81232600	-0.02130000	2.57202300
H	8.28996300	0.36787500	3.47578300
C	0.03544600	3.22940900	-4.56077300
H	0.75236400	2.38788700	-4.57556900
H	0.18278100	3.78095800	-5.50459800
C	2.04145700	2.07005400	0.93446200
H	2.04616500	2.57432200	-0.04177300
H	3.07693900	2.02146500	1.30613000
H	1.45115800	2.67158400	1.64133500
C	-0.96714700	3.88439000	3.43028700
H	-1.04214800	3.49237100	4.45822600
H	0.10723000	4.07449200	3.24938100
C	-4.29968500	1.44720000	0.93868900
H	-3.70885400	0.72420300	1.51181500
H	-4.33903200	2.38170200	1.52487900
C	1.44622200	-0.48693100	2.55991200
H	0.76158800	0.04991500	3.23554200
H	2.47048700	-0.42665400	2.95913200
H	1.12221100	-1.53756300	2.53783800
C	-3.29722000	-1.87321400	3.58489200
H	6.22793600	1.17527800	4.29431100
H	4.82374500	0.16743000	3.84407800
H	5.08465200	1.70655100	3.02863300
H	0.13127000	0.27792000	-1.27053100
Si	-0.62477700	-2.84713400	-3.24070300

H	0.02366600	-1.59367900	0.11783800
C	-0.17820000	-4.21785300	-4.42541300
H	-0.59904100	-3.98881500	-5.41735200
H	0.91125700	-4.33343400	-4.53933900
H	-0.59155300	-5.18507700	-4.09924400
O	-2.25379600	-2.64227000	-3.32068400
O	0.11593100	-1.42418700	-3.64893200
C	1.47465200	-1.07195800	-3.77795700
H	1.88551500	-0.72927900	-2.81368900
H	2.09296400	-1.91013600	-4.15055200
H	1.56004300	-0.24702600	-4.50392100
C	-3.09291900	-1.82429200	-2.52481300
H	-2.62304100	-0.84801000	-2.31331800
H	-4.02743200	-1.64180500	-3.07837000
H	-3.34045300	-2.30851300	-1.56583400
C	-1.10350100	-4.31967900	-0.92704500
C	1.20757300	-3.63495100	-1.23705700
C	1.10825300	-4.52994300	-0.00003100
C	-0.16321300	-5.33988300	-0.27022400
H	-1.75374000	-3.84485200	-0.18179300
H	-1.75673800	-4.77670200	-1.68682300
H	1.66783900	-4.18843700	-2.07661900
H	1.79694500	-2.72374300	-1.05833400
H	2.00694200	-5.14914400	0.14123800
H	0.97088100	-3.91195300	0.90098500
H	0.05361300	-6.17234300	-0.96142600
H	-0.60325800	-5.76240000	0.64418900
N	-0.20958300	-3.26981700	-1.54393500
H	-0.27267800	-2.01975800	-0.66537700

INT11:

Rh	-0.89916700	0.00477500	0.03003300
P	-1.08915300	2.32432800	-0.20705600
S	-2.75966900	-1.32054000	2.43889700
Si	1.30302900	-0.23462600	0.72311300
O	2.36521000	0.11069700	-0.58767700
O	-2.27315500	-0.05821400	1.72462600
O	-2.14722400	-2.54634300	1.89157500
O	-4.19930500	-1.30334100	2.69220000
N	4.35505600	-0.35199300	0.48644200
F	-2.32594600	0.07094000	4.64881800
F	-2.29232800	-2.08841500	4.92327600
C	6.43256300	-0.41044300	-0.81702200
C	-3.12258900	3.74092700	-1.80507500
H	-3.12458800	4.62308400	-1.15374300
H	-2.35339500	3.90997400	-2.56379400
C	-2.82435600	2.47368500	-0.96877500
H	-2.83449400	1.62128300	-1.66551000
C	0.85385700	3.26426500	-3.86365200
H	0.88972100	2.70974200	-4.80884000
H	0.31584100	4.20019100	-4.07243400
C	3.70592300	-0.07573800	-0.61863500
C	0.07530400	2.44734900	-2.81786200
H	0.56817800	1.47926800	-2.67457500
H	-0.93251000	2.23215500	-3.18984200
F	-0.61200100	-1.08517200	3.97016800
C	2.24644400	4.28234700	-2.01014400
H	1.78692800	5.27577100	-2.11526600
H	3.26730300	4.44914100	-1.64703700
C	6.36680300	0.01578000	-3.34418900
H	7.11850900	0.81315900	-3.33463600
H	5.64376100	0.24198400	-4.13131000

H	6.88488500	-0.91076500	-3.61595100
C	-1.36011300	4.89563600	1.11216100
H	-0.89669800	5.32361600	0.21662500
H	-2.44554100	4.94434700	0.96161100
C	-5.31979900	2.11281300	-0.65196100
H	-5.31664200	1.21492400	-1.28594500
H	-6.10513200	1.95892000	0.09653400
C	-0.93684000	3.42313500	1.31736900
H	0.14858700	3.41717700	1.48367900
C	7.83775000	-0.59886000	-0.81748100
H	8.39085100	-0.51476400	-1.74710900
C	1.45870800	3.46935600	-0.96582800
H	1.44579000	4.02709600	-0.02389800
H	1.97348500	2.52154000	-0.77696000
C	-0.98934200	5.74925900	2.33906900
H	0.10536800	5.78736200	2.43196500
H	-1.32391200	6.78156400	2.18086200
C	0.02396700	3.18358700	-1.46167900
H	-0.46124400	4.15453600	-1.62738500
C	5.71219600	-0.52401400	0.41397000
C	8.50140300	-0.88828600	0.35445200
H	9.57779200	-1.03275600	0.35135200
C	4.32655900	0.05289900	-1.89330000
H	3.70598400	0.26758600	-2.75661600
C	6.41475500	-0.82463500	1.62207300
C	5.68515800	-0.11079900	-2.00578600
C	5.66346000	-0.94367300	2.92232700
C	-1.59041100	5.17949500	3.63137700
H	-1.27111800	5.77780500	4.49278800
H	-2.68585300	5.25805300	3.58526800
C	-4.49068000	3.62362400	-2.50366000
H	-4.68886500	4.54212000	-3.06919600

H	-4.44521400	2.80883600	-3.24013900
C	-1.57259800	2.85081000	2.60246400
H	-2.66279000	2.82447600	2.50427900
H	-1.26198300	1.81785200	2.75535300
C	-5.62691400	3.34630200	-1.51059100
H	-5.75650800	4.21991100	-0.85577400
H	-6.57426600	3.21611400	-2.04676500
C	7.78612000	-0.99926700	1.56585800
H	8.32619900	-1.22846700	2.48103700
C	2.27319200	3.58920000	-3.37883600
H	2.84989100	2.65768300	-3.29757600
H	2.79287200	4.21680400	-4.11250900
C	1.71817400	0.89950600	2.17108500
H	1.74737000	1.95427000	1.88722500
H	2.71341000	0.62206100	2.53251200
H	0.99645900	0.77052700	2.98215700
C	-1.19655200	3.70858500	3.82196300
H	-1.67670200	3.29404400	4.71563200
H	-0.11220400	3.64164200	3.99178200
C	-3.95999000	2.23749000	0.05521200
H	-3.76746700	1.34934300	0.66129800
H	-4.00214500	3.08969300	0.74554700
C	1.52652300	-2.04591400	1.20056600
H	0.76403100	-2.35465600	1.92141200
H	2.51753000	-2.17853700	1.64541900
H	1.45379900	-2.69894200	0.32536000
C	-1.95360900	-1.09423900	4.10076000
H	6.34356000	-1.18635600	3.74333700
H	4.89498000	-1.72159900	2.86898600
H	5.14513300	-0.01090200	3.16849800
H	-0.13248600	0.08729600	-1.30106800
H	-1.12855400	-1.80591200	0.20702500

H	-0.81642400	-1.67619500	-0.52497200

TS9:

Rh	-0.88052800	0.10726800	0.22931500
P	-1.09577800	2.30869800	-0.16151400
S	-2.65729900	-1.37648100	2.39399400
Si	1.33974400	-0.06929700	0.79712400
O	2.40383900	0.25588900	-0.52353800
O	-2.46482600	-0.01502900	1.72688500
O	-1.74498000	-2.38159300	1.81076400
O	-4.05924300	-1.72775600	2.60462700
N	4.38837100	-0.30657900	0.51369500
F	-2.61304800	-0.01201200	4.66210800
F	-2.08188200	-2.11388200	4.86547300
C	6.44472000	-0.41975400	-0.81952600
C	-3.09128100	3.66455200	-1.85430900
H	-3.06049600	4.58922400	-1.26493100
H	-2.32229600	3.75421500	-2.62610900
C	-2.83170800	2.44690700	-0.93397800
H	-2.86249700	1.55022000	-1.57065800
C	0.87129500	3.14571000	-3.82405400
H	0.95127000	2.54706500	-4.73910700
H	0.28360400	4.03730000	-4.08636300
C	3.73511500	0.01848200	-0.57591600
C	0.12625700	2.33968500	-2.74579000
H	0.66778200	1.40826600	-2.54758700
H	-0.86496500	2.05235800	-3.11409600
F	-0.66077300	-0.70896000	4.00154900
C	2.18225800	4.33163800	-2.00902600
H	1.66779100	5.29087000	-2.16539300
H	3.18805600	4.57420500	-1.64665600
C	6.36132800	0.05586800	-3.33722500

H	7.14377800	0.82307400	-3.32431700
H	5.63706500	0.32446200	-4.10979300
H	6.83893300	-0.88501300	-3.63280500
C	-1.37953000	4.88982300	1.11455600
H	-0.91059100	5.30590700	0.21618300
H	-2.46355200	4.92731900	0.95217900
C	-5.33862700	2.18794300	-0.59994600
H	-5.36459300	1.25177300	-1.17501000
H	-6.12746200	2.10692900	0.15644200
C	-0.94344600	3.42459500	1.34804600
H	0.14241100	3.42777000	1.51059000
C	7.84132000	-0.66246900	-0.84329900
H	8.38508000	-0.58072700	-1.77864400
C	1.43164300	3.52542600	-0.93202800
H	1.37688500	4.12620400	-0.01869400
H	1.99725900	2.61905200	-0.69538100
C	-1.02750400	5.76723600	2.33009700
H	0.06589400	5.81986900	2.43031700
H	-1.37331800	6.79259100	2.15237600
C	0.02139400	3.14013000	-1.42965400
H	-0.50529600	4.07812000	-1.64992700
C	5.73648800	-0.53014800	0.41867800
C	8.50835600	-1.00175900	0.31324800
H	9.57814400	-1.18785400	0.29238200
C	4.34422900	0.14595900	-1.85664300
H	3.72147300	0.39880300	-2.70788300
C	6.44238000	-0.88340400	1.61057600
C	5.69358000	-0.06855100	-1.99161000
C	5.70405200	-0.99995500	2.91846900
C	-1.63116000	5.21085800	3.62679600
H	-1.32824200	5.82878100	4.48016000
H	-2.72725100	5.27159000	3.57044700

C	-4.46461900	3.54637300	-2.54275400
H	-4.63197100	4.43152700	-3.16822600
H	-4.44696900	2.68350600	-3.22341400
C	-1.57161200	2.86320700	2.64190600
H	-2.66032400	2.80941400	2.54253800
H	-1.23282900	1.84099300	2.81322900
C	-5.60780400	3.37318100	-1.53515500
H	-5.70719200	4.29110500	-0.93820100
H	-6.55967000	3.23999600	-2.06247400
C	7.80497900	-1.11024800	1.53183000
H	8.34742900	-1.37962700	2.43458900
C	2.26349700	3.57664800	-3.34255000
H	2.89298500	2.68626900	-3.21149500
H	2.75359800	4.19824700	-4.10124200
C	1.85897700	1.01541900	2.25092600
H	1.91229100	2.07369700	1.98007500
H	2.85709800	0.70209300	2.57133800
H	1.16251600	0.90244100	3.08613400
C	-1.21536700	3.75005900	3.84662500
H	-1.69376100	3.34534300	4.74574100
H	-0.13108200	3.70380000	4.02292400
C	-3.97484600	2.31326000	0.09987100
H	-3.81091000	1.45513600	0.75414800
H	-3.99008100	3.20729700	0.73649000
C	1.47084200	-1.91157200	1.19149400
H	0.78940800	-2.20095100	1.99393700
H	2.50439300	-2.11074400	1.49522300
H	1.24378300	-2.52797800	0.31711100
C	-1.96698200	-1.03505700	4.08914600
H	6.38327600	-1.29428700	3.72317100
H	4.89889700	-1.73914500	2.85756700
H	5.23373200	-0.05036300	3.19513900

H	-0.04616800	0.08927300	-1.06198800
H	-1.51379400	-2.35952000	-0.52681100
H	-1.27944700	-1.98482600	-1.13317300

INT12:

Rh	-0.97911700	0.21114500	0.52236700
P	-1.03499500	2.41301800	-0.04359700
S	-2.60504700	-1.49465200	2.09055900
Si	1.25616700	-0.06825000	1.00850400
O	2.25026900	0.27161600	-0.36614400
O	-2.64949400	0.01513800	2.13330200
O	-1.57501000	-1.86961300	1.04549100
O	-3.87436100	-2.20657900	2.05249000
N	4.32180100	-0.12302900	0.57376500
F	-2.67160600	-1.73524500	4.71176700
F	-1.47792100	-3.25201500	3.70091300
C	6.26377800	-0.43124500	-0.89434100
C	-2.97263200	3.75237600	-1.83207800
H	-2.96395900	4.68641700	-1.25704500
H	-2.17740700	3.83281200	-2.57837100
C	-2.73647200	2.55019100	-0.88790900
H	-2.74130400	1.64556100	-1.51425300
C	1.07875000	3.00578600	-3.66369500
H	1.18469800	2.34745900	-4.53430800
H	0.52176300	3.88895600	-4.00894100
C	3.57421400	0.02591200	-0.49390600
C	0.26914300	2.28741900	-2.56997300
H	0.77267500	1.35588800	-2.29002500
H	-0.71530100	2.00825400	-2.96275600
F	-0.70823500	-1.22843700	3.92533800
C	2.33677500	4.28672000	-1.87586100
H	1.84691600	5.24147200	-2.11673600

H	3.33144900	4.53703600	-1.48861000
C	5.96395500	-0.36012300	-3.43926900
H	6.73074900	0.40310800	-3.61402400
H	5.17337700	-0.22111500	-4.18033800
H	6.43239800	-1.33377200	-3.62187100
C	-1.33543700	5.05973300	1.12032900
H	-0.83429300	5.43933700	0.22300500
H	-2.41348500	5.10191800	0.92162000
C	-5.25415200	2.27750900	-0.63529200
H	-5.25626800	1.33554600	-1.20135900
H	-6.06842200	2.20166700	0.09447200
C	-0.92321600	3.60041200	1.41408400
H	0.15669900	3.59887400	1.61591900
C	7.65815300	-0.66670000	-0.99660200
H	8.12229600	-0.72844200	-1.97540300
C	1.52710900	3.56700600	-0.78085800
H	1.44316800	4.23017100	0.08620500
H	2.06477900	2.67092100	-0.45715500
C	-1.01393600	5.97390400	2.31671400
H	0.07610500	6.01945100	2.45128700
H	-1.34311300	6.99656200	2.09670400
C	0.13180600	3.17081300	-1.31077900
H	-0.36986800	4.10184900	-1.60626300
C	5.66091300	-0.35177600	0.40068400
C	8.42348900	-0.81778500	0.13879700
H	9.49135700	-0.99851500	0.05774400
C	4.07480800	-0.04641000	-1.82572200
H	3.37936300	0.06289800	-2.65072900
C	6.46839300	-0.51087000	1.56974600
C	5.41235300	-0.27247200	-2.03919700
C	5.83989000	-0.42553400	2.93612800
C	-1.66536800	5.46523300	3.61020600

H	-1.38362100	6.10671500	4.45345700
H	-2.75836300	5.53571500	3.51597500
C	-4.32153600	3.61882300	-2.56425300
H	-4.47433900	4.49490200	-3.20603200
H	-4.27794500	2.74741800	-3.23273900
C	-1.59999000	3.08655200	2.70391900
H	-2.68599000	3.04059500	2.57064700
H	-1.27621700	2.06531500	2.91954500
C	-5.49618900	3.45176900	-1.59203300
H	-5.61825500	4.37554700	-1.00838200
H	-6.43033100	3.30878500	-2.14777400
C	7.82400400	-0.73908600	1.41404300
H	8.44330100	-0.86090200	2.29921100
C	2.45842800	3.44318600	-3.15204900
H	3.06511500	2.55388200	-2.93660800
H	2.99072800	4.00579000	-3.92840100
C	1.79718600	1.03675500	2.44438900
H	1.82728500	2.09379100	2.16510500
H	2.80631300	0.74098200	2.74658800
H	1.12058700	0.92434600	3.29736700
C	-1.27490500	4.00756700	3.89223800
H	-1.78783200	3.64161700	4.78917300
H	-0.19773200	3.95509100	4.10584900
C	-3.91434600	2.41810600	0.10668800
H	-3.77094200	1.56737900	0.77766000
H	-3.95658300	3.31607600	0.73651200
C	1.54041800	-1.89159000	1.40099300
H	1.01879400	-2.18665300	2.31355100
H	2.61407600	-2.05774900	1.53661100
H	1.18133600	-2.53025400	0.58982900
C	-1.81531400	-1.96173400	3.71485900
H	6.58769700	-0.58111800	3.71859700

H	5.04965100	-1.17334800	3.05819500
H	5.37052900	0.55096500	3.09595500
H	-0.24962800	-0.01243900	-0.79932500

Siloxane:

Si	12.82732300	7.88605800	8.72715900
H	11.52404700	7.18468000	8.51273400
C	13.93793200	7.07399100	9.98087900
H	14.86991400	7.63767000	10.08583100
H	13.45530000	7.03246100	10.96180400
H	14.19167800	6.05449300	9.67571600
O	13.64330700	7.87372400	7.28139500
O	12.48653300	9.40691900	9.29909800
C	11.46017900	10.24904000	8.80111100
H	11.16304100	10.94222400	9.59394600
H	11.81241100	10.83790300	7.94441400
H	10.57180700	9.68064200	8.49132200
C	13.05595200	8.12980500	6.01647900
H	13.03642200	9.20563600	5.79975300
H	13.65744100	7.63653600	5.24688900
H	12.02933300	7.74258800	5.95037800

H2:

H	1.26425600	0.92857100	0.00000000
H	0.52145900	0.92857100	0.00000000

Pyrrolidine:

C	0.49536200	1.24024800	0.08209300
C	2.39591000	2.55914700	0.08692800
C	1.23019900	3.57627000	-0.05200900
C	-0.04833900	2.69068200	-0.04505100
H	-0.08677300	0.50432900	-0.47990400

H	0.48718900	0.92787500	1.13431500
H	2.68072400	2.45689000	1.14209800
H	3.29485600	2.84556000	-0.46642000
H	1.23289400	4.31588200	0.75464300
H	1.31209500	4.12965400	-0.99348600
H	-0.72928100	2.94478900	0.77315500
H	-0.60939400	2.81325500	-0.97742000
N	1.89260500	1.25746400	-0.37792500
H	1.89143500	1.26324700	-1.39752400

Siloxazane:

C	11.73412000	5.46850500	9.51356500
C	12.84761600	5.16221500	7.38248700
C	12.57592300	3.79179200	8.02439600
C	11.39093400	4.07999600	8.95577100
H	12.38015800	5.36769200	10.40104000
H	10.84153700	6.02046200	9.82838300
H	12.26019500	5.27460300	6.45842900
H	13.90186500	5.27998900	7.10408600
H	12.37061300	3.01376200	7.28330800
H	13.44431000	3.47335500	8.61317100
H	10.46036900	4.12752400	8.37776700
H	11.26076600	3.33104600	9.74227700
N	12.42549600	6.14787400	8.40042800
Si	13.13000800	7.72285000	8.54101100
C	14.77023000	7.82626900	9.42376600
H	15.12466200	8.86063700	9.46215100
H	14.68502500	7.46045000	10.45170200
H	15.52966900	7.22820000	8.91065400
O	13.42088500	8.27980400	7.00361700
O	12.09137900	8.66346900	9.43314000
C	10.72703500	8.90312800	9.13086800

H	10.18529600	9.07502600	10.06676000
H	10.61892200	9.79700500	8.50339300
H	10.26106600	8.05590700	8.61102800
C	12.45965400	8.39498700	5.96734100
H	12.01116100	9.39657900	5.96413300
H	12.95985200	8.24125200	5.00553400
H	11.65548200	7.65401200	6.06094700

INT6-H:

Rh	0.96631800	0.46303100	-0.92236900
P	1.17709600	-1.49688000	0.21296100
S	2.03748100	3.13760400	-0.79708000
Si	-1.23266800	1.01606800	-0.38554600
O	-2.18588500	-0.34086700	0.16067900
O	2.09918600	2.15970600	0.35333400
O	1.03396500	2.55617600	-1.79795200
O	3.33289100	3.49329000	-1.40089100
N	-4.14873200	0.81967800	0.51503900
C	-6.13189700	-0.38683200	1.31038800
C	3.48076800	-3.22137000	0.84925900
H	3.10512200	-3.47629200	1.84691100
H	3.06954200	-3.96487000	0.16002600
C	3.03399300	-1.79106100	0.46718200
H	3.42273200	-1.58463700	-0.53850100
C	0.92339500	-4.55727200	-2.72215100
H	1.40829700	-4.62482600	-3.70353600
H	1.30830600	-5.39772000	-2.12656900
C	-3.48333000	-0.31249600	0.52573900
C	1.31229800	-3.23158500	-2.04593300
H	1.01371700	-2.39432900	-2.68724600
H	2.40211100	-3.17741900	-1.94780500
C	-1.29069800	-4.52011500	-1.50694400

H	-1.01475700	-5.36038800	-0.85314500
H	-2.38005900	-4.56351400	-1.62542800
C	-6.00637700	-2.91359600	1.72157000
H	-6.37767800	-2.86895700	2.75175300
H	-5.28626200	-3.73287600	1.65713000
H	-6.86149200	-3.16339000	1.08325900
C	0.43212500	-2.89588000	2.68164900
H	0.27981200	-3.78315800	2.05777900
H	1.45005100	-2.96388600	3.08535700
C	5.21549000	-0.88270200	1.42517200
H	5.60663800	-0.60962000	0.43614500
H	5.63631600	-0.15944400	2.13366400
C	0.27843600	-1.59400800	1.86575900
H	-0.76287700	-1.53205500	1.52803000
C	-7.49419100	-0.32112800	1.69726800
H	-8.00867600	-1.22433600	2.00891400
C	-0.90201100	-3.19657100	-0.82549000
H	-1.38858400	-3.13757800	0.15181800
H	-1.28375200	-2.35110000	-1.40786500
C	-0.56840300	-2.91627300	3.85303300
H	-1.59028200	-2.94518300	3.44923800
H	-0.43652300	-3.83611900	4.43595300
C	0.62710000	-3.06814500	-0.67280300
H	0.96328100	-3.89220400	-0.02959400
C	-5.46162300	0.80843700	0.90036700
C	-8.16549700	0.88187600	1.67930800
H	-9.20909800	0.92983500	1.97654200
C	-4.06226400	-1.55693900	0.91619400
H	-3.44924300	-2.45106000	0.88985600
C	-6.17205000	2.04950200	0.88711700
C	-5.37666200	-1.60766700	1.30792300
C	-5.47083900	3.31112000	0.45608800

C	-0.41502800	-1.68383900	4.75631000
H	-1.17602200	-1.69638600	5.54572100
H	0.56006700	-1.72843000	5.26251700
C	5.01665600	-3.33734300	0.84999600
H	5.30634800	-4.35431300	1.14157000
H	5.38758300	-3.19045500	-0.17454300
C	0.50409400	-0.37175000	2.78383900
H	1.51791300	-0.39438000	3.19856900
H	0.42318400	0.55702600	2.21467300
C	5.67005400	-2.30499900	1.77812800
H	5.39109800	-2.52756300	2.81816700
H	6.76252900	-2.38410000	1.72522000
C	-7.49999200	2.05905700	1.27502200
H	-8.04467700	3.00000000	1.26766800
C	-0.59813300	-4.69102600	-2.86503600
H	-0.96514100	-3.92116300	-3.55817500
H	-0.85712800	-5.66028800	-3.30759200
C	-1.33261800	2.33335200	0.99008900
H	-1.93488100	1.97102500	1.82763000
H	-1.82575800	3.23365600	0.60615200
H	-0.34230300	2.60126200	1.36555400
C	-0.50283600	-0.38170800	3.94646900
H	-0.32511600	0.48275600	4.59721700
H	-1.51939000	-0.26841400	3.54548100
C	3.68186600	-0.75976900	1.42055300
H	3.38520400	0.25794400	1.15141100
H	3.32318500	-0.93973100	2.44110300
C	-2.07126100	1.63267900	-1.96622400
H	-1.47900600	2.43809400	-2.40929400
H	-3.07631500	2.00030300	-1.73957500
H	-2.15427500	0.82879600	-2.70461400
C	1.31349900	4.66671600	-0.18805400

H	-6.13964500	4.17310800	0.53178500
H	-5.11929100	3.23574800	-0.57836800
H	-4.58410600	3.50056000	1.06904300
H	0.21677100	-0.37293800	-1.95765100
C	4.46540100	0.51324800	-2.23339200
C	2.90984000	-0.28763900	-3.77067700
C	4.13059700	0.13529900	-4.63039200
C	5.13326500	0.74241500	-3.60772700
H	4.68326000	1.29490000	-1.50491800
H	4.77166900	-0.45415800	-1.81817800
H	2.95829500	-1.35659100	-3.54099200
H	1.94528500	-0.09193700	-4.24515100
H	4.55223800	-0.71829600	-5.17011500
H	3.84165100	0.87797500	-5.38015200
H	6.12525400	0.28416300	-3.66598300
H	5.25808600	1.81551600	-3.78132100
N	3.00935900	0.46584100	-2.49958600
H	2.72293000	1.42382100	-2.70356500
H	1.99106600	5.07791300	0.56166400
H	1.22609100	5.34938200	-1.03423500
H	0.33719000	4.45508900	0.24455600

TS6-H:

Rh	-0.42088700	0.10619200	-0.36933600
P	-1.43092300	2.15189300	-0.45942000
S	-1.73107700	-2.10708700	1.87934200
Si	1.59615400	0.69246600	0.64569500
O	2.77672500	0.14033800	-0.50969400
O	-1.54627100	-0.65708900	1.44096800
O	-0.46601900	-2.78944700	2.23624200
O	-2.61127500	-2.86807600	0.94778500
N	4.73075300	0.37478400	0.70661900

C	6.86465600	-0.21205000	-0.35609600
C	-4.13454900	2.89819400	-1.39372300
H	-4.19427400	3.78930200	-0.75835800
H	-3.68297800	3.21555600	-2.33703100
C	-3.28902100	1.80546100	-0.69458400
H	-3.26644000	0.93500300	-1.36452800
C	-0.98390600	3.23232300	-4.54268000
H	-1.12500400	2.62612700	-5.44548400
H	-1.73847100	4.03134500	-4.58331100
C	4.11527800	0.04642400	-0.40644800
C	-1.22397300	2.36223700	-3.29721700
H	-0.52921100	1.51460600	-3.30400000
H	-2.23020300	1.93279400	-3.33648800
C	0.65973100	4.63731300	-3.23246100
H	-0.00811400	5.51035600	-3.20376000
H	1.68368000	5.02861700	-3.20623600
C	6.89509900	-1.05758600	-2.77645500
H	7.62107300	-0.31837300	-3.13383400
H	6.20006400	-1.27051000	-3.59216500
H	7.45301700	-1.97534200	-2.55817200
C	-2.08810600	4.65439400	0.89746200
H	-2.01324700	5.13887600	-0.08233200
H	-3.14316500	4.39286700	1.04180100
C	-5.41744000	0.84955600	0.32551400
H	-5.36384700	-0.06140100	-0.28505300
H	-5.89567500	0.56077800	1.26940400
C	-1.22088700	3.37743800	0.95715900
H	-0.17899100	3.69313300	0.82854600
C	8.27314500	-0.31091500	-0.23114800
H	8.86331800	-0.66456900	-1.07035500
C	0.41097400	3.76877700	-1.98612200
H	0.57135900	4.37752600	-1.09025100

H	1.14574800	2.95529400	-1.95705600
C	-1.67221100	5.64914100	1.99685900
H	-0.64618100	5.99233100	1.80288600
H	-2.31221600	6.53857100	1.94994500
C	-1.00875900	3.16616500	-1.99433900
H	-1.71997900	4.00290100	-1.99045700
C	6.09376600	0.25468900	0.75481500
C	8.89425900	0.03866600	0.94810000
H	9.97369500	-0.03851300	1.04120500
C	4.79455500	-0.42593600	-1.56963600
H	4.20735400	-0.66816500	-2.44930000
C	6.75296000	0.61348400	1.97208700
C	6.15967500	-0.55991200	-1.55807900
C	5.94773100	1.10410900	3.14693000
C	-1.74081200	5.01193000	3.39171800
H	-1.39957100	5.72483100	4.15167700
H	-2.78857600	4.77917300	3.63014900
C	-5.56098600	2.38863600	-1.67508500
H	-6.14362300	3.18407100	-2.15571400
H	-5.50909300	1.56160700	-2.39740800
C	-1.31680300	2.72605100	2.35600800
H	-2.34354400	2.39561400	2.54280700
H	-0.70030200	1.82447600	2.40166300
C	-6.26262000	1.90463400	-0.39956700
H	-6.42525300	2.76166900	0.26992400
H	-7.25507700	1.50499300	-0.64018600
C	8.13008800	0.49837400	2.04167500
H	8.63568600	0.76925000	2.96534800
C	0.41752500	3.85681900	-4.53085800
H	1.16857600	3.05932900	-4.61843300
H	0.55278400	4.51144400	-5.40008900
C	2.15053200	2.46508600	1.02393900

H	2.09074300	3.14543500	0.17240700
H	3.20202400	2.38682200	1.31926900
H	1.59888900	2.89657500	1.86337800
C	-0.90930900	3.72257600	3.45509500
H	-1.01536000	3.24854700	4.43808600
H	0.15486600	3.97518200	3.34611200
C	-3.99200500	1.35500400	0.60664400
H	-3.40375200	0.58048200	1.10420100
H	-4.06022900	2.20911400	1.29252000
C	1.78266500	-0.29624300	2.24397900
H	1.24824900	0.22357500	3.04772400
H	2.84414300	-0.34441700	2.50651500
H	1.36334100	-1.30201600	2.18288400
C	-2.66648100	-1.92591100	3.41218900
H	6.59837500	1.33123800	3.99617300
H	5.21117400	0.35845500	3.46320700
H	5.38208400	2.00657200	2.89265500
H	0.38075400	0.58921700	-1.58484200
Si	-1.19610800	-2.38808200	-1.97002200
H	-1.59697100	-0.90781500	-1.94178700
C	-0.30009500	-2.78276200	-3.57376100
H	0.58742300	-2.15007200	-3.67705500
H	0.01487100	-3.82766000	-3.63764000
H	-0.96575800	-2.58222700	-4.41887300
O	-2.76625400	-2.97788700	-2.08589300
O	0.00416300	-2.04804300	-0.77385800
C	1.26885000	-2.71574800	-0.65259700
H	1.39976400	-3.01921700	0.38710200
H	1.29782100	-3.59390200	-1.29957300
H	2.05615100	-2.02047300	-0.94354100
C	-3.95019400	-2.24736700	-1.82080900
H	-3.86809700	-1.20169200	-2.14781700

H	-4.76678300	-2.71524300	-2.38160600
H	-4.17854800	-2.26989600	-0.75113300
C	-1.63660700	-5.70687800	-1.93539200
C	-0.27318000	-5.37210600	-0.06640400
C	-1.00897100	-6.69458100	0.22317000
C	-1.77446700	-7.00321900	-1.09222300
H	-2.56074500	-5.38222000	-2.41203600
H	-0.88120000	-5.84260500	-2.71868100
H	0.68628000	-5.57210100	-0.55780700
H	-0.10117700	-4.76120800	0.82074100
H	-0.32321200	-7.49554000	0.51554500
H	-1.71105500	-6.54830100	1.05048800
H	-1.35051800	-7.86235800	-1.62141200
H	-2.82455000	-7.23677700	-0.89255500
N	-1.15695900	-4.64532100	-1.01464300
H	-1.94972200	-4.31757800	-0.45569900
H	-3.60617000	-1.41650500	3.19543500
H	-2.85802000	-2.92422700	3.80813600
H	-2.06895000	-1.34657500	4.11684500

TS7-H:

Rh	-0.48996600	-0.18482900	-0.11482200
P	-1.35572600	1.87358200	-0.35881200
S	-1.78955500	-1.91237200	2.51365700
Si	1.60648900	0.38151700	0.67900100
O	2.64227400	0.60872900	-0.69010400
O	-1.19955900	-0.61140500	1.99163300
O	-0.77027100	-2.93367100	2.83355300
O	-2.89911400	-2.40580400	1.63679500
N	4.69441600	0.68110500	0.36911400
C	6.72689500	0.87572300	-0.99355600
C	-4.02786200	2.81234300	-1.11308000

H	-4.22959300	3.29177100	-0.14743900
H	-3.51847100	3.55571900	-1.73561200
C	-3.14758700	1.56205200	-0.89300300
H	-2.99905100	1.09366500	-1.87502100
C	-0.32194500	3.22814900	-4.27087700
H	-0.39107300	2.71931700	-5.23976700
H	-0.99453400	4.09633600	-4.32690300
C	3.98559600	0.69317100	-0.73622200
C	-0.80760700	2.27957500	-3.16009500
H	-0.20987300	1.36052100	-3.17464900
H	-1.84128700	1.98577600	-3.36987300
C	1.23805900	4.35764700	-2.63453700
H	0.64267200	5.28197500	-2.60649700
H	2.27691000	4.65030900	-2.44170400
C	6.54510600	0.99005800	-3.54872400
H	7.12536000	1.91406500	-3.65245600
H	5.78093100	0.98098200	-4.32952100
H	7.23199200	0.15715900	-3.73731100
C	-1.68065300	4.54123600	0.85712100
H	-1.08064200	4.94686000	0.03761600
H	-2.73024400	4.64563100	0.56052800
C	-5.24391200	0.14801400	-0.67336000
H	-5.05307300	-0.36994400	-1.62428900
H	-5.76756100	-0.56787900	-0.02935000
C	-1.34446400	3.05404900	1.12180200
H	-0.29330600	3.01490600	1.42445200
C	8.14134400	0.96397900	-1.02193000
H	8.65535000	1.04449100	-1.97423900
C	0.76031600	3.40409100	-1.52563800
H	0.85232600	3.90352800	-0.55510700
H	1.41677600	2.52835200	-1.49447900
C	-1.43083000	5.38566800	2.12153500

H	-0.35622200	5.37501400	2.35204000
H	-1.69506500	6.43107200	1.92067000
C	-0.69107500	2.93700100	-1.76823400
H	-1.33696400	3.82550200	-1.77155400
C	6.05696200	0.76958600	0.26606500
C	8.86426000	0.94687700	0.15096100
H	9.94808200	1.01386800	0.12607700
C	4.55774300	0.79363900	-2.03910200
H	3.89432600	0.78937700	-2.89751300
C	6.82108200	0.75324900	1.47459200
C	5.91910000	0.88548900	-2.18122600
C	6.12265500	0.64261600	2.80473900
C	-2.21329600	4.85799100	3.33155200
H	-1.97662000	5.45007300	4.22366500
H	-3.29012900	4.98536300	3.14897400
C	-5.37022800	2.43012900	-1.76369400
H	-5.98507200	3.32915400	-1.89333300
H	-5.18143600	2.03371000	-2.77144700
C	-2.17026700	2.52710100	2.31668900
H	-3.23885700	2.58250600	2.07111400
H	-1.93422100	1.47678900	2.50442400
C	-6.12223100	1.37925700	-0.93519900
H	-6.41844100	1.82354200	0.02593500
H	-7.04997400	1.08897200	-1.44272100
C	8.19944000	0.84139800	1.39113500
H	8.78488300	0.82852800	2.30722800
C	1.11138200	3.71475400	-4.02160600
H	1.79993300	2.86076600	-4.08691900
H	1.41464600	4.42325400	-4.80174600
C	1.92672300	1.84223100	1.84120000
H	1.86862800	2.81673500	1.34882700
H	2.94684800	1.72799200	2.22132200

H	1.23506300	1.83015300	2.68971000
C	-1.91583700	3.37262300	3.57644900
H	-2.52649500	2.99306100	4.40473900
H	-0.86732900	3.25592400	3.88460900
C	-3.89970900	0.52495600	-0.02757800
H	-3.30152400	-0.37594800	0.12688800
H	-4.08769300	0.93896600	0.96944900
C	2.10598000	-1.20139900	1.57956300
H	1.59211700	-1.25571500	2.54280800
H	3.18918900	-1.17630600	1.74014300
H	1.84164100	-2.09642300	1.01579300
C	-2.56295400	-1.42268100	4.06543900
H	6.84704300	0.62458900	3.62387500
H	5.51256000	-0.26458800	2.85999500
H	5.44008800	1.48380900	2.96585500
H	0.08070400	0.08625400	-1.50638600
Si	-0.66521700	-3.19967200	-1.68309300
H	-1.19361500	-1.89786800	-0.88640800
C	-0.10376700	-4.84158600	-2.51195500
H	0.40452000	-4.59654100	-3.45082000
H	0.59489600	-5.44510300	-1.92360100
H	-0.94963100	-5.48088800	-2.78426900
O	-1.54447400	-2.75686700	-3.06295200
O	0.86332800	-2.62069600	-1.18851900
C	2.14127100	-3.11103900	-1.57195100
H	2.88788100	-2.38286900	-1.24297300
H	2.36908600	-4.07630700	-1.10231100
H	2.22901400	-3.22619200	-2.65850200
C	-2.30582900	-1.59855300	-3.29675400
H	-1.93542600	-1.08506900	-4.19352100
H	-3.35808300	-1.86594500	-3.46836900
H	-2.25637800	-0.89651400	-2.45436800

C	-3.10384000	-4.67534000	-0.88485400
C	-1.21500800	-5.26227400	0.50005300
C	-2.34611000	-6.29909600	0.70993300
C	-3.62384800	-5.58673900	0.22828100
H	-3.75954000	-3.83584300	-1.12402700
H	-2.89871800	-5.23256100	-1.80239100
H	-0.36509200	-5.69148400	-0.02853400
H	-0.86618600	-4.81328900	1.43136600
H	-2.15924200	-7.19192300	0.10366800
H	-2.41347100	-6.61803900	1.75313200
H	-4.39570100	-6.27819000	-0.12189500
H	-4.04567100	-4.97232700	1.03004100
N	-1.81167800	-4.14885300	-0.33390300
H	-2.10332300	-3.42271500	0.34261100
H	-3.31835800	-0.66374500	3.85945600
H	-1.79002700	-1.02740300	4.72557100
H	-3.02145900	-2.30901400	4.50572200

TS-I:

Rh	9.26640200	3.68078500	10.59498000
P	7.54721900	2.33770700	11.64012700
S	9.05840800	6.55735500	12.65662400
Si	7.76744700	5.16856800	9.07703100
O	8.73531900	6.59623500	9.08326800
O	9.18271400	5.06460500	12.46204000
O	7.67558000	7.05762400	12.63950400
O	10.06614600	7.33383200	11.90252400
N	7.52044500	7.96380400	7.67169600
F	10.85861500	6.19850200	14.55770100
F	9.65364900	7.99698600	14.78788400
C	7.91463300	10.38435600	7.71495400
C	7.19564200	0.24157100	13.62954100

H	6.62697700	0.85900900	14.33422000
H	6.46710000	-0.18586300	12.93346700
C	8.24696300	1.10678800	12.89387000
H	8.82059400	0.45157200	12.22929000
C	6.03805100	-1.00956600	9.40501800
H	6.50283200	-1.95490200	9.09941600
H	5.23769600	-1.27247400	10.11173300
C	8.41673100	7.83593000	8.61940600
C	7.08207500	-0.13352800	10.12165100
H	7.92752900	0.05481200	9.45490100
H	7.48558400	-0.68263800	10.97556400
F	8.80131000	6.05084500	15.25335400
C	4.85298700	1.06707600	8.58739000
H	3.98142000	0.92052100	9.24105400
H	4.48985100	1.60302800	7.70229800
C	9.59062900	11.37352200	9.38414200
H	10.18159500	11.91884600	8.63896900
H	10.26418200	11.04295200	10.17770100
H	8.88149800	12.08770200	9.81767800
C	4.80637400	2.99485600	12.50289000
H	4.47416100	2.77672900	11.48465000
H	4.69556900	2.06560200	13.07840600
C	9.96079800	0.52559800	14.66139400
H	10.54256800	-0.07568500	13.94848600
H	10.67738300	0.93850600	15.38146000
C	6.28206700	3.45990800	12.53836500
H	6.35322500	4.39287100	11.97509600
C	7.59267200	11.65637500	7.17798400
H	8.09067500	12.54126700	7.56043100
C	5.89587700	1.92865200	9.32013200
H	5.45019100	2.89004700	9.58586700
H	6.72441000	2.14773900	8.63778500

C	3.87371600	4.06889500	13.09580600
H	3.90710300	4.95958100	12.45374400
H	2.83983300	3.70273400	13.06946500
C	6.46088100	1.21037900	10.56075600
H	5.61662000	0.96956800	11.21758600
C	7.25307500	9.22364000	7.20465600
C	6.65311800	11.77323100	6.17715300
H	6.40886500	12.74941400	5.76822000
C	9.11862600	8.92319500	9.20992600
H	9.79393500	8.71774500	10.03205600
C	6.27934400	9.36067500	6.16697000
C	8.87466400	10.19956800	8.76642900
C	5.58055000	8.14170400	5.62324800
C	4.27335900	4.45993800	14.52329600
H	3.62480400	5.26439400	14.89031200
H	4.11463100	3.60073700	15.19164900
C	7.88157700	-0.89984900	14.40480000
H	7.12555500	-1.48532100	14.94222500
H	8.35472500	-1.58509100	13.68701200
C	6.66922600	3.80123400	13.99694600
H	6.58480100	2.90331000	14.62044900
H	7.70066400	4.14377400	14.04457100
C	8.94488700	-0.37432900	15.37919400
H	8.45171700	0.20269100	16.17433200
H	9.45340000	-1.21174900	15.87200000
C	6.00201800	10.62492800	5.67826300
H	5.26291200	10.73506300	4.88849200
C	5.42787200	-0.29834900	8.19050900
H	6.20618600	-0.15437000	7.42769900
H	4.65194700	-0.92149900	7.73033600
C	7.51591100	4.70193900	7.25509700
H	8.46855100	4.49076600	6.75788200

H	7.05138600	5.55136100	6.74895200
H	6.87306300	3.82364300	7.13554700
C	5.74456000	4.88585600	14.57692000
H	6.04564600	5.09681800	15.60987500
H	5.89158300	5.81502400	14.01431100
C	9.26533000	1.67366000	13.90963900
H	10.00800500	2.29464100	13.40177900
H	8.76492300	2.31809200	14.63745800
C	6.11047200	5.73167300	9.79116400
H	5.37117800	4.93518200	9.90094400
H	5.70866000	6.47165900	9.09060200
H	6.25798600	6.22425900	10.75620200
C	9.62478000	6.71132900	14.42350000
H	4.86018200	8.41960300	4.84871400
H	5.04810800	7.60215000	6.41342900
H	6.29581200	7.43369800	5.19116900
H	8.66298600	3.44205600	9.17066700
C	12.05498600	5.17675300	10.95062900
C	11.48123500	5.10382000	8.63453000
C	12.84047600	5.84510600	8.76016200
C	12.96894900	6.19418200	10.26111800
H	11.70062500	5.49364100	11.92752600
H	12.54002000	4.20613900	11.04188600
H	11.60669800	4.08737000	8.26133100
H	10.77204100	5.61768000	7.98481600
H	13.65724600	5.18947400	8.44149200
H	12.87803700	6.73646900	8.12821200
H	13.99981300	6.14090900	10.62286000
H	12.59559100	7.20343600	10.46181600
N	10.89264000	5.05833300	10.01597900
H	10.37207200	5.93299400	10.12836200
Si	10.71962100	1.86478900	9.90439100

H	10.35715600	2.76054900	11.44052600
C	12.58774500	1.85151800	10.20141000
H	13.12123600	2.63987700	9.66542700
H	12.83748000	1.92028600	11.26479300
H	12.97620000	0.89944000	9.82284200
O	10.13256200	0.38432900	10.44802300
O	10.75683300	1.79455600	8.21793100
C	9.69834000	1.47490200	7.33865500
H	10.05676400	1.59681900	6.31143100
H	9.36517000	0.43577600	7.46594300
H	8.83274700	2.13347400	7.47828400
C	10.68304300	-0.85539200	10.01927000
H	11.01398100	-0.82296300	8.97422400
H	11.53745600	-1.14043200	10.64563600
H	9.91194900	-1.62708500	10.11496300

INT-I:

Rh	9.39516800	3.56448900	10.94293600
P	7.70236900	2.12591600	11.82825600
S	8.68754300	6.91531800	12.38144800
Si	7.46130500	5.44141000	8.64620200
O	8.59545200	6.70067100	8.70993700
O	8.42026200	5.46654300	12.08348600
O	7.47215600	7.73471500	12.44949300
O	9.83609900	7.46474600	11.61809600
N	7.41396100	8.12730500	7.35119000
F	10.33774600	5.97673600	14.24365900
F	9.82626600	8.08186000	14.46367600
C	7.99593300	10.50657600	7.29515400
C	7.19419900	-0.04202500	13.69605500
H	6.66947200	0.59053400	14.42219000
H	6.44190200	-0.38187800	12.97706600

C	8.30897500	0.77225900	12.99969700
H	8.82768100	0.11414500	12.29315600
C	6.03745400	-0.91269200	9.27011600
H	6.44923500	-1.84278300	8.85981000
H	5.22141400	-1.20678100	9.94606100
C	8.36339800	7.95727500	8.23777700
C	7.12717900	-0.17832300	10.07269600
H	7.98775100	0.02526000	9.43154600
H	7.49137800	-0.83307700	10.86942100
F	8.38394000	6.54904000	15.00363200
C	4.95740300	1.29914800	8.69065300
H	4.07991200	1.11973000	9.32805200
H	4.61692100	1.94410200	7.87109600
C	9.85125100	11.40349600	8.82058000
H	10.42954000	11.88344800	8.02250100
H	10.54995100	11.04171600	9.57779300
H	9.22789400	12.17932600	9.27888400
C	4.97195400	2.70342100	12.82258700
H	4.57946300	2.45995700	11.83158300
H	4.92708000	1.78078800	13.41526300
C	9.95357000	-0.01565400	14.76569200
H	10.49488400	-0.64307900	14.04352800
H	10.69138500	0.31656000	15.50584700
C	6.42959300	3.21760000	12.74375900
H	6.43017100	4.13043100	12.13750700
C	7.73713200	11.78688400	6.74374400
H	8.32788900	12.63816100	7.06517700
C	6.03771100	2.02507500	9.51066800
H	5.63216300	2.96246100	9.90382200
H	6.87173400	2.29578200	8.85282800
C	4.04393600	3.75598800	13.45850600
H	3.99568800	4.63280200	12.79795700

H	3.02555100	3.35204400	13.51688600
C	6.57899000	1.14197200	10.65185200
H	5.72781000	0.86843100	11.28586000
C	7.21044000	9.39176500	6.86364900
C	6.74165700	11.95419400	5.80625900
H	6.54638400	12.93669000	5.38652400
C	9.18694400	8.99296400	8.74999400
H	9.89458800	8.75420500	9.53470400
C	6.18009000	9.58000300	5.89191300
C	9.00946300	10.27365000	8.28575100
C	5.35262000	8.40752400	5.43313200
C	4.53284600	4.19623200	14.84251900
H	3.87796600	4.97809000	15.24520500
H	4.46965000	3.34601800	15.53765000
C	7.77790400	-1.26172900	14.43270300
H	6.97114800	-1.80912100	14.93528800
H	8.21253300	-1.95239100	13.69608700
C	6.91110600	3.63583000	14.15186100
H	6.92532400	2.75862400	14.81069100
H	7.92836700	4.02785800	14.10176400
C	8.85814700	-0.85128100	15.44186100
H	8.39597200	-0.26054100	16.24559200
H	9.29204200	-1.73899600	15.91754800
C	5.96904300	10.85043600	5.38655700
H	5.18722900	11.00085800	4.64623000
C	5.46965300	-0.03947000	8.14326200
H	6.25879500	0.15134300	7.40210000
H	4.66721900	-0.56798200	7.61502500
C	7.19908100	4.78982000	6.90575500
H	8.15324800	4.56670500	6.41852300
H	6.68438000	5.54907200	6.31199200
H	6.59530400	3.87600500	6.90792700

C	5.98054700	4.69353400	14.76997000
H	6.34562900	4.95820600	15.76868600
H	6.02776300	5.60984500	14.16950500
C	9.37167700	1.20662800	14.03380100
H	10.17801100	1.75446700	13.53826300
H	8.93609000	1.89053300	14.76979700
C	5.89736600	5.94716500	9.53911300
H	5.18268900	5.12289900	9.62201800
H	5.41727000	6.75796500	8.98319700
H	6.13606900	6.31512400	10.54127000
C	9.34277200	6.88208500	14.12848000
H	4.60108800	8.72336600	4.70433200
H	4.83899600	7.92899600	6.27352200
H	5.97890200	7.63741400	4.96983000
H	8.20789500	4.35933200	9.41347900
C	12.05867000	5.23659500	11.34216400
C	11.50262700	5.15905000	9.01098100
C	12.87550000	5.86686900	9.16673200
C	12.94552100	6.27662800	10.65219900
H	11.67195800	5.55693400	12.30780700
H	12.58180400	4.28768500	11.46693200
H	11.60492600	4.16801700	8.57024800
H	10.80122400	5.72890500	8.40067400
H	13.68662500	5.17232300	8.92307500
H	12.97179800	6.72350500	8.49396600
H	13.96701700	6.28538600	11.04371000
H	12.51519900	7.27164200	10.80433200
N	10.91533300	5.04731900	10.39491200
H	10.35293100	5.89511700	10.52755700
Si	10.66265500	1.89057000	9.97470700
H	10.26377700	3.07007300	12.08575300
C	12.51864000	1.89666700	10.35786600

H	13.04404800	2.75335100	9.93023500
H	12.70512800	1.88045400	11.43668600
H	12.97013500	0.99740200	9.92448200
O	10.13153500	0.34612800	10.41439700
O	10.71813000	1.93556000	8.28436600
C	9.64444500	1.71850400	7.39259900
H	10.00844000	1.86837100	6.37041500
H	9.25154300	0.69500800	7.46756100
H	8.81732200	2.41773600	7.56641600
C	10.77221700	-0.84111200	9.96752900
H	11.11819500	-0.75931800	8.92947600
H	11.63357800	-1.08901700	10.60104400
H	10.05359600	-1.66608800	10.02683400

TS-II:

Rh	-0.77994700	0.28667400	-1.01636500
P	-2.39557500	1.68198200	0.04072400
S	1.67220700	-2.71139800	0.80403000
Si	1.16666300	1.38061000	-0.29550200
O	2.43049400	0.27217300	-0.67164200
O	0.22617400	-2.38089100	1.11235100
O	2.59528100	-1.94007500	1.63848000
O	1.95633000	-2.80212300	-0.64743800
N	4.25275000	1.46125800	0.07365400
F	1.57523400	-5.37724000	0.47906900
F	3.16388400	-4.67207500	1.75837800
C	6.47763000	0.44167800	0.00764800
C	-5.19097000	2.22063500	-0.75805600
H	-5.44836300	2.58238300	0.25192900
H	-4.84079800	3.09365500	-1.32590100
C	-4.09134600	1.13757800	-0.65848100
H	-3.82978500	0.84398600	-1.69273500

C	-2.37335600	5.30235600	-2.19028200
H	-2.20099800	5.46657800	-3.26713300
H	-3.41885200	5.60487100	-1.99394300
C	3.75312200	0.35320000	-0.41302100
C	-2.20346900	3.81176300	-1.86104700
H	-1.19686900	3.48406000	-2.16650800
H	-2.90907800	3.21264200	-2.45944200
F	1.12408200	-4.70476700	2.48797400
C	-1.60069900	5.89841700	0.14157200
H	-2.60613200	6.23402400	0.45701100
H	-0.87977400	6.49154000	0.72864200
C	6.74474000	-1.97425400	-0.80998200
H	7.51891000	-1.74813400	-1.56226900
H	6.13123700	-2.80342000	-1.18869200
H	7.26890400	-2.32562200	0.09415900
C	-3.68061000	2.39526600	2.56752700
H	-3.82908900	3.39939600	2.13934100
H	-4.60046500	1.82587100	2.35461700
C	-5.92164000	-0.64045600	-0.64550400
H	-5.66546900	-0.98424500	-1.66305500
H	-6.28660500	-1.52814600	-0.10361900
C	-2.47015300	1.68808300	1.92358900
H	-1.57884200	2.29170500	2.16385600
C	7.86806900	0.58683700	0.25401300
H	8.54491900	-0.24164900	0.03796800
C	-1.44071200	4.40810400	0.48835000
H	-1.63809800	4.27497400	1.56215700
H	-0.39587700	4.10550700	0.32560200
C	-3.50890900	2.50610000	4.09163500
H	-2.65789900	3.17676000	4.31212800
H	-4.40099500	2.98351900	4.53119900
C	-2.39215400	3.53497900	-0.35550100

H	-3.41123300	3.85711900	-0.08852800
C	5.59866200	1.53563800	0.29587500
C	8.36882400	1.76658100	0.76703100
H	9.44007200	1.87300200	0.95598000
C	4.53586800	-0.79981000	-0.71661800
H	4.02204800	-1.69741600	-1.06009100
C	6.13267500	2.75286200	0.83064900
C	5.89469300	-0.76717400	-0.51253200
C	5.21209800	3.90362400	1.13849700
C	-3.25614200	1.13872200	4.73655300
H	-3.09291500	1.25242600	5.82126500
H	-4.15948000	0.51102500	4.62276200
C	-6.46314500	1.68329400	-1.43496700
H	-7.22538200	2.48036400	-1.46032900
H	-6.23436100	1.44060300	-2.48927600
C	-2.22507200	0.30906200	2.56424500
H	-3.06732200	-0.36569100	2.34476800
H	-1.32838500	-0.15595800	2.12883400
C	-7.00718700	0.43378100	-0.73814400
H	-7.35059500	0.70077000	0.27857600
H	-7.89126100	0.04898100	-1.27366700
C	7.49838400	2.84142200	1.05313800
H	7.91104500	3.76842000	1.46194200
C	-1.42808200	6.17351100	-1.35615700
H	-0.38371800	5.96298700	-1.65038900
H	-1.59953300	7.24169700	-1.57007400
C	1.62984400	2.94740400	-1.24961300
H	1.70767600	2.72927800	-2.32621100
H	2.62214800	3.26265700	-0.89266300
H	0.92359100	3.77811200	-1.12646400
C	-2.06219600	0.43017800	4.08688400
H	-1.92383400	-0.57216100	4.52433900

H	-1.13679500	0.99294600	4.30752700
C	-4.65408700	-0.11553500	0.04500000
H	-3.91181400	-0.92221500	0.09143800
H	-4.91027500	0.13802700	1.08689000
C	1.23471000	1.59257000	1.57506200
H	0.49456000	2.30002500	1.97379200
H	2.24433100	1.96593800	1.80970300
H	1.11667600	0.61438100	2.06559500
C	1.89086600	-4.48392200	1.41529700
H	5.76896500	4.75194500	1.56315300
H	4.42749700	3.60804600	1.85319600
H	4.68864900	4.25147900	0.23298800
H	-0.66374200	1.42973800	-2.01179500
C	-0.31196700	-2.03899800	-3.02668100
C	1.15996900	-0.28235800	-3.45206600
C	1.66485100	-1.43036200	-4.34440200
C	0.67054500	-2.59194700	-4.07984300
H	-0.61877300	-2.79678800	-2.29807000
H	-1.20660100	-1.60696000	-3.50493500
H	0.44897800	0.35698400	-3.99773700
H	1.96122100	0.34687400	-3.05101800
H	1.69999700	-1.12913000	-5.40224300
H	2.68625900	-1.71902500	-4.05430100
H	0.14247300	-2.91645400	-4.98910500
H	1.19635200	-3.46662000	-3.67068700
N	0.42243500	-0.94103400	-2.33898900
H	1.12023100	-1.39873800	-1.72799400
Si	-1.71532900	-2.96073700	0.36087800
H	-1.52146600	-1.54093200	-0.22103600
C	-2.09931800	-3.28984500	2.16605200
H	-1.54495600	-4.19271100	2.46754300
H	-1.75375600	-2.47985100	2.82026400

H	-3.17595500	-3.46012200	2.30793500
O	-3.30208600	-3.18635700	-0.27615100
O	-0.86295300	-4.12947100	-0.49876100
C	-1.30247100	-5.44713600	-0.74301900
H	-0.77416600	-6.15479700	-0.08181400
H	-2.38885600	-5.56688400	-0.58803400
H	-1.06814500	-5.72429100	-1.78562700
C	-3.61644800	-3.13609200	-1.63682600
H	-3.07539200	-3.89688100	-2.23181200
H	-4.69654000	-3.31488800	-1.77259100
H	-3.38441000	-2.14828600	-2.08929400

INT-II:

Rh	-0.80179600	0.28348100	-1.00974600
P	-2.42301600	1.68796200	0.03102400
S	1.82771400	-2.85860600	0.66871300
Si	1.14437600	1.33259400	-0.24049300
O	2.40530900	0.22626300	-0.64269900
O	0.43435500	-2.43300700	1.11890900
O	2.85789700	-2.22292100	1.48672900
O	1.99399900	-2.84795000	-0.80150000
N	4.22068300	1.38638100	0.16447100
F	1.77788000	-5.47891600	0.06576600
F	3.14528400	-4.93536000	1.64264600
C	6.46736300	0.44198600	-0.06815700
C	-5.19467900	2.20623400	-0.85923900
H	-5.47623800	2.61482900	0.12636300
H	-4.82246600	3.05029200	-1.45606700
C	-4.10686700	1.12155000	-0.68097900
H	-3.82423700	0.77713100	-1.69390100
C	-2.35245000	5.27815400	-2.25464800
H	-2.15212900	5.42741100	-3.32885300

H	-3.40370300	5.57988500	-2.09031700
C	3.73380000	0.32017500	-0.41843100
C	-2.18493900	3.79344100	-1.89929400
H	-1.16792900	3.46774000	-2.16996000
H	-2.86809900	3.18096000	-2.51016000
F	1.02126500	-5.01892900	2.04437200
C	-1.64493600	5.91027800	0.08749900
H	-2.65922000	6.24859900	0.37005000
H	-0.94153800	6.51340600	0.68562100
C	6.77025800	-1.86767900	-1.14015400
H	7.51809100	-1.54216000	-1.88249600
H	6.16745800	-2.66854900	-1.59031300
H	7.32682900	-2.29854700	-0.29146400
C	-3.75356600	2.47038500	2.51297100
H	-3.88696200	3.46391700	2.05647800
H	-4.67238900	1.90153200	2.29521400
C	-5.95013200	-0.64097500	-0.63180700
H	-5.67015000	-1.03497100	-1.62457100
H	-6.33971800	-1.49825600	-0.05851200
C	-2.53416800	1.73919000	1.91307600
H	-1.64426400	2.34441600	2.15600300
C	7.86060100	0.59991800	0.15326400
H	8.55113200	-0.18272400	-0.16579100
C	-1.49302000	4.42568300	0.46163400
H	-1.72224500	4.31043800	1.53090500
H	-0.44319300	4.12202300	0.33609700
C	-3.61432900	2.61980100	4.03705900
H	-2.76410700	3.29106300	4.25901100
H	-4.51276900	3.11362200	4.44439000
C	-2.41667800	3.53609100	-0.39641700
H	-3.44432100	3.85781400	-0.16428600
C	5.57043200	1.47540400	0.35589900

C	8.34695600	1.73424100	0.77163900
H	9.42043500	1.85070400	0.94093200
C	4.53618700	-0.77255400	-0.86178600
H	4.03707300	-1.64483700	-1.28311300
C	6.08966700	2.64537700	0.99959800
C	5.89943300	-0.72266300	-0.69458800
C	5.15023200	3.73056000	1.45379400
C	-3.38438700	1.26822600	4.72235200
H	-3.24508200	1.40866500	5.80728200
H	-4.28839100	0.64240500	4.60388400
C	-6.45224700	1.64666700	-1.54496600
H	-7.20745400	2.44649900	-1.62821900
H	-6.19700100	1.35345900	-2.58019000
C	-2.31016500	0.37761600	2.59593200
H	-3.14613600	-0.30397500	2.37495000
H	-1.40533200	-0.09635400	2.19080100
C	-7.02444400	0.43487700	-0.80551500
H	-7.39363600	0.75187900	0.18751500
H	-7.89631500	0.03053300	-1.34665200
C	7.45889500	2.74887900	1.19193400
H	7.86038300	3.63977500	1.68368800
C	-1.43180400	6.16393000	-1.40865400
H	-0.37954000	5.95082200	-1.67126500
H	-1.59908200	7.22856700	-1.64273400
C	1.63729800	2.92391800	-1.13706600
H	1.73777700	2.73515200	-2.21739800
H	2.62163200	3.22829400	-0.75025500
H	0.92986800	3.75234400	-1.00730200
C	-2.17994000	0.53772700	4.11799500
H	-2.05675700	-0.45373400	4.58361300
H	-1.25758000	1.10319700	4.34510900
C	-4.69711800	-0.09278000	0.06825800

H	-3.96066100	-0.89960300	0.18137000
H	-4.98332900	0.21632400	1.08695900
C	1.18663400	1.48870900	1.63627000
H	0.45597600	2.20495100	2.03821300
H	2.20079800	1.82831600	1.89999000
H	1.02807100	0.50262400	2.09867100
C	1.94008800	-4.69773700	1.12959400
H	5.69949700	4.54947100	1.94138700
H	4.40296700	3.34086800	2.16348800
H	4.58204800	4.14984000	0.60758400
H	-0.64421800	1.45000400	-1.98328200
C	-0.31169100	-2.05639000	-3.00450700
C	1.10741800	-0.26363600	-3.46684500
C	1.62395900	-1.40514900	-4.36226200
C	0.67422000	-2.59526200	-4.06099900
H	-0.58842100	-2.81237400	-2.26166500
H	-1.22443700	-1.65673800	-3.47646200
H	0.37631400	0.35975900	-4.00391300
H	1.90059700	0.38489500	-3.07907800
H	1.62000500	-1.11305700	-5.42324900
H	2.66148500	-1.65970400	-4.09906000
H	0.14587200	-2.95656900	-4.95604600
H	1.23605000	-3.44098700	-3.63969900
N	0.40124700	-0.93136000	-2.33973700
H	1.12043700	-1.36783300	-1.74014700
Si	-1.47353100	-2.87314100	0.52349400
H	-1.30729400	-1.45008600	-0.10365400
C	-1.85973900	-3.11419900	2.34455400
H	-1.47087000	-4.10459400	2.63261000
H	-1.34970500	-2.37974400	2.98066500
H	-2.94329000	-3.09244600	2.52578400
O	-3.09537000	-3.04542700	-0.06547500

O	-0.74585200	-4.13439800	-0.33547300
C	-1.30487500	-5.40617100	-0.57510900
H	-0.78171700	-6.16675200	0.02935200
H	-2.38045700	-5.44778200	-0.33327100
H	-1.17536100	-5.67609500	-1.63809200
C	-3.45620200	-3.02461500	-1.41320600
H	-2.93663400	-3.79533400	-2.01408500
H	-4.54006000	-3.20753800	-1.50860100
H	-3.24371900	-2.04539100	-1.89497000

TS-III:

Rh	9.48277100	3.69677700	10.04221200
P	7.38948400	2.58500700	10.96500000
S	10.73081200	0.16400400	10.15706600
Si	8.64518700	5.67035200	9.12657500
O	8.15946000	6.74754000	10.39598500
O	10.10551800	-0.83748400	11.03814800
O	10.67921500	1.56888900	10.67881400
O	10.38671100	0.07554300	8.71272000
N	7.35770000	8.53026000	9.15971100
F	12.77514500	-1.44421100	9.69692200
F	13.24052400	0.67171000	9.48445500
C	6.92052200	10.66401500	10.29200100
C	6.91845500	0.88236700	13.21576500
H	6.38533500	1.64773100	13.79461000
H	6.17930300	0.39392100	12.57398700
C	8.05318000	1.54060400	12.39180500
H	8.59021300	0.74923700	11.86092000
C	6.00360000	-0.90062300	8.94573600
H	6.54747700	-1.81425300	8.68219900
H	5.19780500	-1.20217900	9.63054000
C	7.75463700	8.03186000	10.30810400

C	6.96032500	0.05790600	9.67699700
H	7.82573400	0.25999700	9.03387700
H	7.35785300	-0.44147100	10.56353800
F	13.00436000	-0.20008200	11.46670800
C	4.70534600	1.06874600	8.03887100
H	3.83199000	0.87487000	8.67778600
H	4.32531200	1.55434400	7.13232700
C	7.39216000	10.89676300	12.80122000
H	8.03749400	11.77679200	12.70119600
H	7.76376400	10.29886400	13.63665200
H	6.39180500	11.26047400	13.06241800
C	4.77439000	3.50467700	11.82915700
H	4.34599000	2.98849100	10.96782600
H	4.76451000	2.79330000	12.66370200
C	9.66100300	1.15218300	14.29409000
H	10.21534400	0.40787700	13.70896900
H	10.38322100	1.63755200	14.96075800
C	6.22701800	3.96818500	11.55274500
H	6.20902500	4.66510700	10.70869700
C	6.47170800	12.00426900	10.18206100
H	6.45542900	12.63902200	11.06197700
C	5.66275800	2.02749200	8.76789100
H	5.14150600	2.96281200	8.99597700
H	6.48467600	2.29350000	8.09033100
C	3.87388700	4.70454900	12.17995700
H	3.80100900	5.36478100	11.30456200
H	2.85897800	4.34260300	12.38288900
C	6.25169400	1.38162000	10.04134200
H	5.42703400	1.15356800	10.72605500
C	6.93961900	9.83386400	9.12709600
C	6.05814100	12.50267900	8.96611200
H	5.71517400	13.52998400	8.88453400

C	7.77923600	8.77916600	11.52283400
H	8.13881400	8.29371300	12.42318200
C	6.50689500	10.36585000	7.87223100
C	7.36743000	10.08846200	11.52898900
C	6.52062200	9.49743100	6.64127800
C	4.41399400	5.50021100	13.37297900
H	3.77946100	6.37305100	13.56576000
H	4.37057000	4.87517400	14.27634500
C	7.50726000	-0.16219300	14.18445200
H	6.69410300	-0.60967700	14.76895000
H	7.96113100	-0.97098400	13.59728200
C	6.76683500	4.73912100	12.77682100
H	6.80258600	4.06690500	13.64187600
H	7.78301300	5.08896600	12.58680500
C	8.56637200	0.45106700	15.10863500
H	8.08611900	1.17743900	15.78067500
H	9.00186100	-0.32565700	15.74821300
C	6.07841000	11.68031700	7.81943600
H	5.74853200	12.08959400	6.86775000
C	5.39128000	-0.25926500	7.69451800
H	6.18469400	-0.07633500	6.95647100
H	4.67759500	-0.94457900	7.22249300
C	9.87801000	6.61487300	8.03847900
H	10.84488700	6.76327300	8.52324200
H	9.44983500	7.58796700	7.77897400
H	10.06133700	6.06663600	7.10825300
C	5.86222800	5.93466900	13.12053700
H	6.26685900	6.44783200	14.00103100
H	5.89301500	6.65774800	12.29646800
C	9.08038500	2.20571000	13.33312900
H	9.88984400	2.65840200	12.76297300
H	8.60497700	3.00041600	13.91853800

C	7.08588300	5.40781100	8.06558300
H	7.24498300	4.68416500	7.26198900
H	6.83330400	6.37660500	7.62412200
H	6.21918700	5.07794500	8.64456500
C	12.54882700	-0.23000800	10.20621700
H	6.17415400	10.05624000	5.76743300
H	5.87742300	8.62005300	6.76753400
H	7.52591700	9.11690800	6.43338900
H	8.17225400	2.67434200	9.57834700
C	9.49106400	2.96649200	6.76273000
C	11.66270400	3.21766600	7.58656000
C	11.83335500	2.41653300	6.28321500
C	10.40603700	2.35576200	5.67221600
H	8.54840200	2.42874800	6.89127000
H	9.25850800	4.00817100	6.53037500
H	11.75463800	4.29271800	7.42332700
H	12.36354500	2.94276800	8.37181700
H	12.56081800	2.88089000	5.61131500
H	12.18975800	1.40871900	6.51464000
H	10.32947600	2.91989900	4.73799600
H	10.11894000	1.32355300	5.45490000
N	10.26961900	2.92338000	8.03065200
H	10.28379600	1.93215400	8.29694800
Si	11.34804600	4.94724700	10.92862900
H	9.29724000	4.39933300	11.42572100
C	11.07501100	6.64413400	11.71460000
H	12.01481100	6.99427800	12.15530800
H	10.72079500	7.39708700	11.00680000
H	10.33669700	6.57083600	12.51858800
O	12.00873100	4.10117300	12.21767500
O	12.53998300	5.11109600	9.74688500
C	13.75856900	5.80646400	9.96932800

H	13.59166200	6.88379200	10.10096000
H	14.29358000	5.43089300	10.85136700
H	14.39947700	5.66349500	9.09298800
C	12.96765600	3.05508600	12.18203100
H	12.51123800	2.11195600	12.48926900
H	13.39405200	2.91672600	11.18262200
H	13.77986900	3.30249000	12.87705500

INT-III:

Rh	10.10764500	4.03880100	9.32805000
P	7.30192200	2.41615600	12.13934400
S	9.83953200	0.59614900	9.31276100
Si	9.13464200	5.69529600	8.11034100
O	7.87119300	6.49393900	9.02998600
O	8.54473700	-0.02812600	9.65416600
O	10.20256100	1.75910400	10.19750900
O	10.10101400	0.83840900	7.87926600
N	6.81885100	7.72435500	7.37740000
F	10.88647000	-1.82758200	9.15102400
F	12.34116300	-0.25740200	9.53572400
C	5.14306600	9.39660700	8.03281100
C	6.03640300	0.06862100	13.21382300
H	5.92311200	0.49606200	14.21831600
H	5.14400000	0.35185200	12.64467000
C	7.31699900	0.61261500	12.54561200
H	7.40605600	0.17982400	11.53949700
C	4.62005600	2.39753900	8.91840800
H	4.72370700	1.91768000	7.93898200
H	3.75300900	1.92874300	9.40627800
C	7.00301000	7.44126200	8.64888300
C	5.88361700	2.11802900	9.74808200
H	6.76205800	2.50274700	9.21663600

H	6.03779200	1.04044500	9.85100600
F	11.01902400	-0.92918500	11.12942900
C	4.30241400	4.60005100	10.12281200
H	3.41611100	4.24904900	10.67161600
H	4.18737400	5.68166900	9.99364600
C	4.62128000	9.77588300	10.51424200
H	4.80961600	10.85534700	10.49367100
H	4.92279000	9.39993200	11.49504400
H	3.53831900	9.63829300	10.41577900
C	6.38601900	3.45081600	14.63720300
H	5.38075800	3.44660700	14.19862700
H	6.51580500	2.48548000	15.14273400
C	8.64286500	-1.35257600	13.41203300
H	8.76982400	-1.76123800	12.40244600
H	9.53048400	-1.64741000	13.98321600
C	7.46742200	3.60645200	13.54620900
H	7.31048900	4.57082800	13.04292100
C	4.21264300	10.38097300	7.61527100
H	3.63255000	10.92098500	8.35675300
C	5.56063800	4.33258700	10.96845000
H	5.43946500	4.81015700	11.94716600
H	6.43037800	4.79374500	10.48458800
C	6.50197100	4.58021200	15.67563400
H	6.26544300	5.53789200	15.19141600
H	5.75084600	4.43339200	16.46032300
C	5.79738100	2.81232400	11.13013000
H	4.94971600	2.38766900	11.68674300
C	5.90508000	8.68904200	7.05011900
C	4.04266500	10.65551100	6.27562200
H	3.32942600	11.41082500	5.95832500
C	6.29353000	8.09716100	9.70336200
H	6.51887800	7.81696200	10.72713400

C	5.71707800	8.98620100	5.66350700
C	5.36935200	9.06818800	9.41257200
C	6.51467600	8.24512900	4.62248600
C	7.91124700	4.64861200	16.28205200
H	7.97757200	5.48855400	16.98300400
H	8.09560200	3.73748600	16.86909400
C	6.10963600	-1.46579400	13.32051500
H	5.21175500	-1.83879700	13.82680000
H	6.10135700	-1.89267100	12.30878100
C	8.89088600	3.64927900	14.15003200
H	9.12057700	2.69290900	14.63419400
H	9.63719600	3.81843100	13.36753200
C	7.37482300	-1.92823000	14.05790400
H	7.32116400	-1.60395000	15.10730400
H	7.42020600	-3.02313300	14.07297500
C	4.79633600	9.95632100	5.30895000
H	4.65097500	10.18527900	4.25599000
C	4.36786800	3.90243600	8.75799400
H	5.17664800	4.34689900	8.16390100
H	3.43903000	4.07499800	8.20233100
C	10.13596600	7.12363200	7.35964200
H	10.70344000	7.66870700	8.11957600
H	9.46419700	7.82334300	6.85310500
H	10.85430500	6.74510300	6.62574900
C	8.98454200	4.77736400	15.19179800
H	9.98614500	4.77432300	15.63513300
H	8.87837100	5.74376300	14.68070200
C	8.58474200	0.18149500	13.31808600
H	9.48136000	0.55690400	12.81597600
H	8.56765100	0.60382400	14.33093800
C	8.25307300	4.76263300	6.69766600
H	8.97508900	4.34267500	5.98858400

H	7.60000600	5.45798500	6.16120100
H	7.64264600	3.93344500	7.07120000
C	11.09672500	-0.68389200	9.80821900
H	6.26976800	8.60186100	3.61798800
H	6.31947000	7.16839100	4.66496100
H	7.59030700	8.36810200	4.78400600
H	8.42381500	2.64218100	11.32059400
C	12.23494700	4.12125700	6.80632400
C	13.04323000	2.96169000	8.65258700
C	14.00458600	2.57462400	7.50985800
C	13.50557300	3.40158800	6.29054300
H	11.43832400	4.19144900	6.06242800
H	12.47157300	5.12807100	7.15799900
H	13.39938100	3.84564000	9.18749000
H	12.86209200	2.15988700	9.36775100
H	15.04599800	2.78979600	7.76659500
H	13.93431300	1.50176100	7.30369500
H	14.25193400	4.12139600	5.94201500
H	13.27012600	2.74634000	5.44628100
N	11.76859600	3.34279400	7.98186100
H	11.34331700	2.47372500	7.64027500
Si	11.08078000	5.54058800	10.69063000
H	8.90399000	4.37764100	10.27777100
C	10.18624000	7.07394200	11.34257100
H	10.80361400	7.59209300	12.08454200
H	9.93742100	7.77696000	10.54341600
H	9.24734300	6.78163500	11.82235100
O	11.48681100	4.78752800	12.16550200
O	12.54944500	6.03511700	9.99585200
C	13.44829200	6.91042800	10.65945700
H	13.04981600	7.93272700	10.71719200
H	13.67464700	6.57372800	11.68029700

H	14.38427200	6.94294700	10.09113300
C	12.36939000	3.68228800	12.27458600
H	11.95365000	2.78196400	11.80567200
H	13.34488000	3.89356700	11.81609200
H	12.53350800	3.47756800	13.33911600
